# NXPH4 Used as a New Prognostic and Immunotherapeutic Marker for Muscle-Invasive Bladder Cancer

**DOI:** 10.1155/2022/4271409

**Published:** 2022-10-04

**Authors:** Zhiming Gui, Xiaoling Ying, Chunxiao Liu

**Affiliations:** ^1^Department of Urology, Zhujiang Hospital, Southern Medical University, Guangzhou 510280, China; ^2^Department of Urology, Affiliated Hospital of Guangdong Medical University, Zhanjiang 524000, China; ^3^Laboratory of Translational Medicine, The First Affiliated Hospital of Sun Yat sen University, 510000, China

## Abstract

**Background:**

One of the most common malignant tumors of the urinary system is muscle-invasive bladder cancer (MIBC). With the increased use of immunotherapy, its importance in the field of cancer is becoming abundantly evident. This study classifies MIBC according to GSVA score from the perspective of the GSEA immune gene set.

**Methods:**

This study integrated the sequencing and clinical data of MIBC patients in TCGA and GEO databases, then scored the data using the GSVA algorithm, the CNMF algorithm was implemented to divide the subtypes of GEO and TCGA datasets, respectively, and finally screened and determined the key pathways in combination with clinical data. Simultaneously, LASSO Cox regression model was constructed based on key pathway genes to assess the model's predictive ability (ROC) and describe the immune landscape differences between high- and low-risk groups; key genes were further analyzed and verified in patient tissues.

**Results:**

404 TCGA and 297 GEO datasets were divided into C1-3 groups (TCGA-C1:120/C2:152/C3:132; GEO- C1:112/C2:101/C3:84), of which TCGA-C2 (*n* = 152) subtype and GEO-C1 (*n* = 112) subtype had the worst prognosis. LASSO Cox regression model with ROC (train set = 0.718, test set = 0.667) could be constructed. When combined with the Cancer Immunome Atlas database, it was found that patients with high-risk scores were more sensitive to PD-1 inhibitor and PD-1 inhibitor combined with CTLA-4. NXPH4, as a key gene, plays a role in MIBC with tissue validation results show that nxph4 is highly expressed in tumor.

**Conclusion:**

The immune gene score of MIBC data in TCGA and GEO databases was successfully evaluated using GSVA in this research. The lasso Cox expression model was successfully constructed by screening immune genes, the high-risk group had a worse prognosis and higher sensitivity to immunotherapy, PD-1 inhibitors or PD-1 combined with CTLA-4 inhibitors can be preferentially used in high-risk patients who are sensitive to immunotherapy, and NXPH4 may be a molecular target to adjust the effect of immunotherapy.

## 1. Introduction

According to the American Cancer Association, the United States is predicted to have 1898160 new cancer cases and 608570 death cancer cases in 2021, with the bladder cancer (BC) incidence and mortality rates expected to be 7% and4%, respectively [1]. In patients with BC, accounts for approximately 25% of all cases were MIBC [2]. The disease is characterized by the onset of concealment and always has been progressed to muscle-invasive bladder cancer (MIBC) at the time of clinical discovery with a poor prognosis [3]. It is crucial to predict the survival rate early, further refine the classification of MIBC patients, and provide the basis for individualized treatment. At present, researchers have already tried to build prediction models from different levels, including DNA, RNA (mRNA, miRNA, lncRNA...), protein, gene mutation (TP53), and modification omics (DNA methylation) [4–9]; the prognosis prediction of MIBC patients still needs a more comprehensive and unique method.

GSVA is a nonparametric and unsupervised analysis method that assesses potential pathway activity variation in each sample by inputting the preselected gene set [10]. At present, several studies have shown that the tumor malignant phenotype is related to tumor immune microenvironment [11]. As an immune-sensitive malignant tumor, a variety of invasive immune cells are also related to MIBC, such as regulatory natural killer (NK) cells, T cells (Treg), CD8+ T cells, CD4+ T cells, and macrophages [12]. However, the current research is mostly based on some special immune genes to analyze their mediated downstream pathways, while it is more direct to integrate immune gene sets into the overall analysis based on GSVA algorithm to obtain key immune-related pathways, and it is no longer necessary to speculate on immune pathways through some genes. Recently, research employing GSVA based on mRNA and miRNA sets established and validated the pathway prognosis characteristics of pancreatic cancer [13], respectively. This method performs well in identifying prognostic factors of multiple cancers [14–16]. Therefore, the application of the GSVA algorithm to evaluate the expression score of the GSEA immune gene set in MIBC patients may have incredible potential for typing and risk model construction.

In this study, the GSVA algorithm was used to evaluate the pathway activity variation score of the GSEA immune gene set in each MIBC patient for the first time, and then, the MIBC patients from TCGA and GEO were typed, and the key pathways were obtained. More importantly, we successfully constructed a risk regression model based on the genes of essential pathways, and MIBC patients who were more sensitive to PD-1 inhibitor and CTLA-4 were distinguished using a cancer immunohistochemical database. Our results not only provide a selection basis for clinical personalized therapy, especially immunotherapy, but also provide a new molecular target for molecular therapy and further analyzed and verified the gene NXPH4 that may play a role in tumor immune progression. Neurexophilin 4 (NXPH4) is a secreted protein of synapses, belonging to the neurexophilin (NXPH) family. Its family of proteins was first thought to be a kind of neuroglycoprotein, and its mature peptide molecular weight is 29 KD, because it is related to neurexin I of neuronal membrane proteins *α*. It is named after its close combination and is widely expressed in the nervous system. At present, nxph4 is found to be highly expressed in liver cancer, non-small-cell lung cancer, and many other cancer cells [17]. The roadmap of the whole article is described in Figure S1.

## 2. Materials and Methods

### 2.1. Dataset Acquisition and Preparation

In this study, RNA sequencing data (HTSeq-Counts) of 297 MIBC tissue samples with prognosis were included in three datasets GSE13507 (*n* = 62), GSE31684 (*n* = 74), and GSE32894 (*n* = 161) in the GEO, and TCGA website was used to download the bladder cancer (BC) (*n* = 414) and para-cancerous tissue (*n* = 19) and was screened in combination with their corresponding clinical data. A total of 404 MIBC tissue samples with prognostic information (*T* ≥ *T*2) were obtained; through this method, the sequencing data of MIBC patients were obtained from TCGA database. The original count of RNA sequencing data was transformed into one-millionth transcript (TPM) value, and further log2 transformation (log2(TPM+1)) for subsequent analysis, the infiltration estimation for TCGA was downloaded from the TIMER2.0 database (http://timer.comp-genomics.org/); the Copy Number Variation (CNV) of MIBC also came from TCGA.

### 2.2. Gene Set Variation Analysis (GSVA)

The GSVA technique is a nonparametric estimation method that is often used to investigate the variation of biological pathways among different molecular clusters. In this study, the “c7.immunesigdb_HALLMARK.gmt” gene set was used as the reference gene set for performing GSVA to identify the differences of immune-related pathways between different clusters, and the default parameters were selected to refer to the “GSVA” R package. FDR < 0.05 and *t* value > 2 are set as the cut-off standard.

### 2.3. Unsupervised Clustering Analysis

Based on the GSVA result, the “CancerSubtypes” R package [19] was used to perform the consistent nonnegative matrix decomposition (CNMF) algorithm, selected the default parameters, and constructed the molecular classification in TCGA queue and GEO queue datasets, respectively. Silhouette coefficients are used to estimate clustering results, ranging from -1 to 1. Silhouette coefficients close to 1 show that samples are different from adjacent clusters.

### 2.4. Screening of Differential Pathways among Molecular Typing

After the molecular typing of TCGA queue and GEO queue was obtained based on the CNMF algorithm, the differential expression pathway between each molecular subtype was calculated by the “Limma” algorithm [20], and the coincidence analysis was carried out to obtain the common differential expression pathway between each subtype in TCGA queue and geo queue.

### 2.5. Prognosis Analysis of the Differential Expression Pathway

The “survival” R package was used to select pathways with *P* < 0.05 for analysis using Kaplan-Meier analysis.

### 2.6. Establishment and Validation of the Risk Signature

After acquiring the key pathway, the pathway gene was obtained and extracted from TCGA and Geodata queues. Then, Kaplan Meier analysis and univariate Cox regression analysis were carried out through the “survival” R package to select the gene with *P* < 0.01 for the construction of the lasso Cox expression model. The entire dataset, a total of 701 samples, was randomly divided into training and test sets, and the baseline and clinical features were consistent between the two groups. The test set was used to verify the established model construct by training set. Based on the 29 previously obtained genes with prognostic value, the R software package “Glmnet” was used to carry out LASSO Cox regression analysis. The ROC analyses in the high- and low-risk groups were compared by calculating the area under the curve (AUC). The risk score was calculated using the following formula: risk score = coef (RNA1) x expr (RNA1) ⋯ ..+coef (RNA12) × expr (RNA12); coef (RNA) represents the coefficient of RNA correlated with survival, and expr (RNA) represents the expression of RNA [21].

### 2.7. Acquisition of Immunogenomic Signatures

First, the infiltration estimation for TCGA was downloaded from the TIMER2.0 database, and the differences in immune-related function then of two groups used the R packages “GSEABASE” and “GSVA” combined with the immune gene set's annotation file “immune.gmt” [22] (http://www.gsea-msigdb.org/gsea/index.jsp). The treatment response data of MIBC patients to PD-1 inhibitors and CTLA-4 inhibitors were downloaded from the cancer-immune atlas database (TCIA) (https://tcia.at/). Then, analyze whether there are differences in immunosuppressive therapy between high-risk and low-risk groups.

### 2.8. Immunohistochemical (IHC) Analysis

The expression level of the key gene NXPH4 was detected in paraffin sections of MIBC patients by immunohistochemistry; the histological section' antigen retrieval was conducted with antigen unmasking solution at high pressure with pressure cooker for 20 min after sections were deparaffinized and rehydrated; then, the tumor sections were incubated with specific antibodies (ab74999; suitable for: WB, IHC-P, reacts with: human; diluted 1 : 200) overnight at 4°C after blocked with 10% normal goat serum for 1 h, and horseradish peroxidase-conjugated antibodies were used at room temperature for 1 h; DAB basic kit (Ventana Medical Systems) was used to checked the antigen-antibody reaction sites. And images were acquired with a PathScope™ 4S scanner (DigiPath, USA) by experienced laboratory technicians independently without any information concerning the group in a blinded fashion.

### 2.9. Statistical Analyses

All analyses used R software v4.2.0, SPSS v13.0, and GraphPad Prism 8 to carry out. Bilateral *P* values < 0.05 were considered statistically significant.

## 3. Results

### 3.1. Identification of Three MIBC Molecular Subtypes Based on CNMF

**Figure 1:**
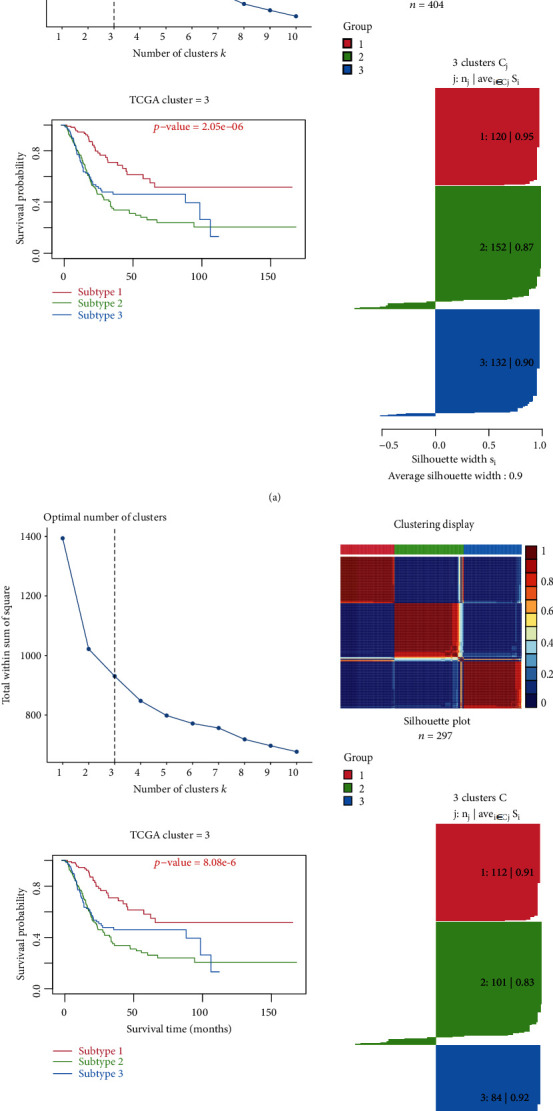
Unsupervised cluster analysis of the muscle invasive bladder cancer (MIBC) GSVA data in TCGA and GEO databases. (a) TCGA: the optimal cluster number was 3; 3 clusters plot of the model; overall survival for the 3 subtypes, the best number of clusters was C1; the C3 cluster also showed a better OS than C2 clusters (log-rank *P* < 0.001); clustering display of the model; silhouette plot of the 3 clusters (C1:120/0.95; C2:152/0.87; C3:132/0.90), average silhouette width = 0.9; (b) GEO: the optimal cluster number was 3; 3 clusters plot of the model; overall survival for the 3 subtypes, the best number of clusters was C3; the C2 cluster also showed a better OS than C1 clusters (log-rank *P* < 0.001); clustering display of the model; silhouette plot of the 3 clusters (C1:112/0.91; C2:101/0.83; C3:84/0.92), average silhouette width = 0.89.

**Figure 2:**
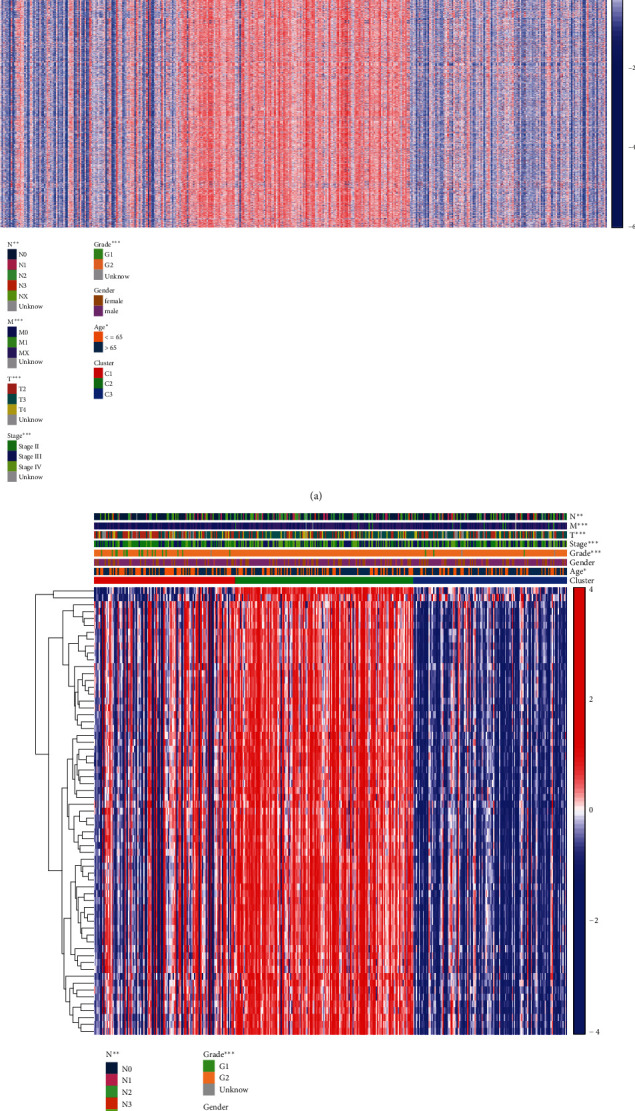
(a) Analysis heatmap of 3 molecular subtypes with clinical data (TCGA). (b) Analysis heatmap of 65 common difference pathways with clinical data and molecular subtypes (^∗^*P* < 0.05, ^∗∗^*P* < 0.01, ^∗∗∗^*P* < 0.001).

Firstly, a total of 404 TCGA MIBC cohorts and 297 GEO cohorts were analyzed for GSVA based on the “c7.immunesigdb_HALLMARK.gmt” gene set, and 4922 immune-related pathways were obtained (Figures 2A); then, molecular classification was performed by CNMF algorithm to characterize three distinct MIBC molecular clusters based on these pathways. The results showed that it is most appropriate for the two groups of data to be divided into three subtypes (Figure 1(a)), TCGA-C1 cluster (*N* = 120), C2 cluster (*N* = 152), and C3 cluster (*N* = 132). And the C1 cluster also showed a better OS than C3 and C2 clusters (log-rank *P* < 0.001). Combined with clinical data, there were significant differences in clinical characteristics such as N, M, T, tumor grade, and stage, including age among the three groups (Figure 2(a), supplement table 1, *P* < 0.05), GEO-C1 cluster (*N* = 112), C2 cluster (*N* = 101), and C3 cluster (*N* = 84). C3 cluster showed a better OS than C1 and C2 cluster (log-rank *P* < 0.001, Figure 1(b), supplement table 2). Overall, molecular classification had an excellent performance to predict the OS of MIBC.

### 3.2. Identification of Differential Pathways among Molecular Typing: GSE21670_STAT3_KO_VS_WT_CD4_TCELL_TGFB_IL6_TREATED_DN

**Figure 3:**
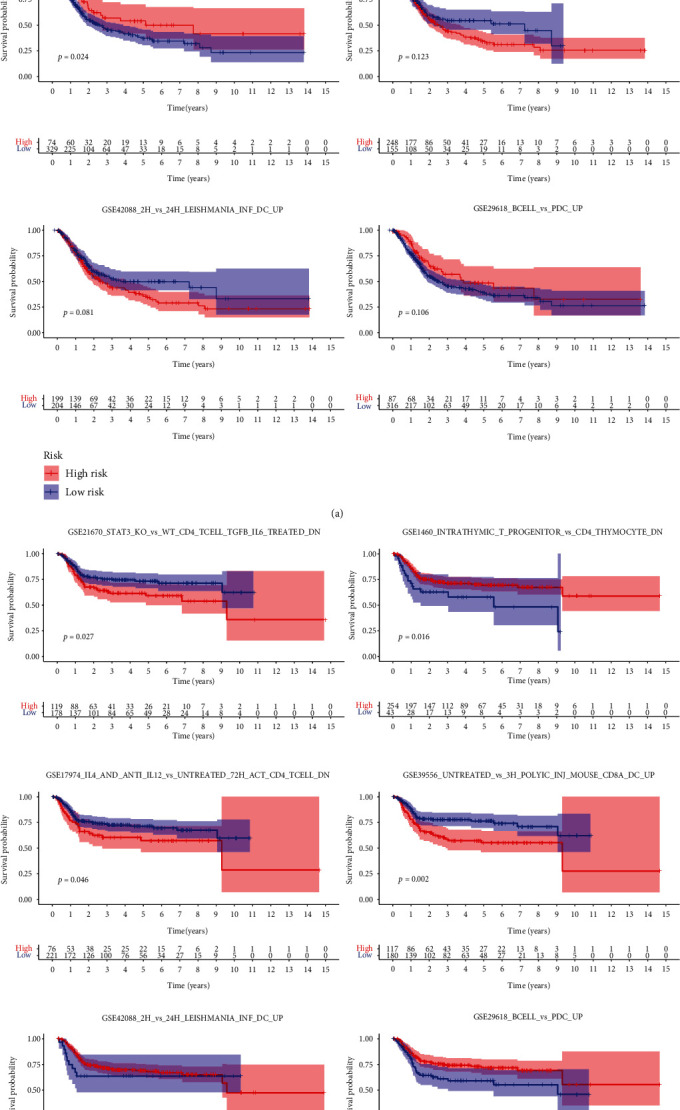
The survival of 6 pathways obtained by coincidence of (a) TCGA and (b) GEO database.

To begin, the data from three TCGA and GEO queues' molecular subtypes were read, and then, the differential enrichment pathways were filtered and overlapped using the “Limma” R package to obtain the pathways with different expressions in the three subtypes. Among them, the molecular subtypes of TCGA queue obtained 65 differential pathways (Figures 2B, supplement table 3, *P* < 0.05), the correlation between them and clinical data was analyzed (Figure 2(b)), while the molecular subtypes of the GEO queue obtained 463 differential pathways (Figures 2C, *P* < 0.05). Finally, six pathways were obtained (Figures 2D, supplement table 4) and then screened in combination with their prognostic information. Among them, there was a prognostic value (*P* < 0.05) with the same prognostic trend which was only one: “GSE21670_STAT3_KO_VS_WT_CD4_TCELL_TGFB_IL6_TREATED_DN,” the high expression of this pathway means a worse prognosis in MIBC patients included in TCGA database or GEO database (Figure 3, supplement Table 5).

### 3.3. Construction and Evaluation of a Risk Model Based on Pathway Genes

**Figure 4:**
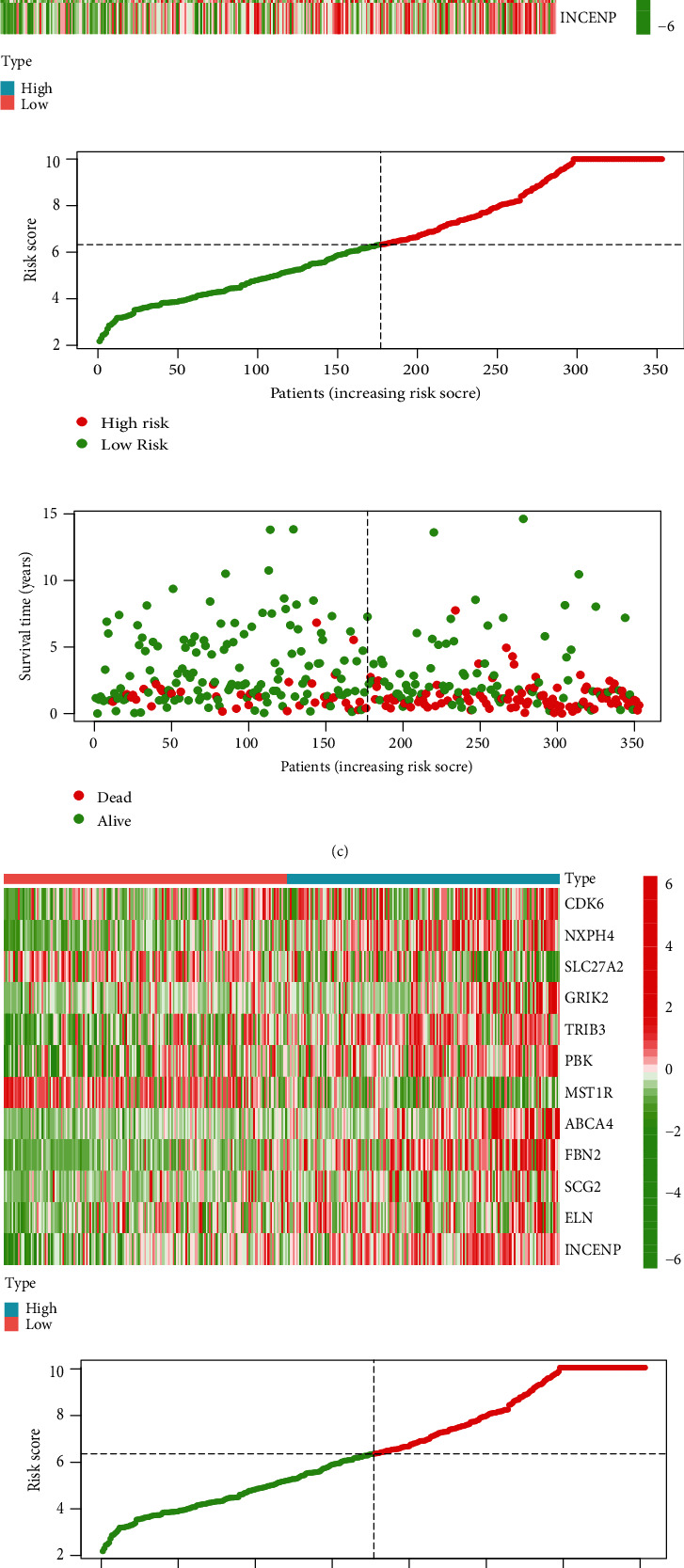
Prognostic value of the risk model based on the 12 prognostic genes in TCGA and GEO database. (a, b) Kaplan-Meier survival analysis of muscle invasive bladder cancer (MIBC) in the high- and low-risk groups for the training set and test set. (c, d) Clustering analysis heatmap shows the levels of the 12 genes for each patient in the training set and test set; distribution of the m6a-related lncRNA model-based risk score in the training set and test set; patterns of the survival status and survival time between the high- and low-risk groups in the training set and test set; (e, f) receiver-operating characteristic (ROC) curves of the risk score in the training set and test set. (g) The CNV variation frequency of 12 genes in TCGA cohort. The height of the column represented the alteration frequency. The deletion frequency, blue dot; the amplification frequency, red dot. (h) The location of CNV alteration of 12 genes on 23 chromosomes using TCGA cohort.

Firstly, this study obtained 200 genes of the “GSE21670_STAT3_KO_VS_WT_CD4_TCELL_TGFB_IL6_TREATED_DN” pathway based on the “c7.immunesigdb_HALLMARK.gmt” gene set. Finally, in the 701 cohorts of TCGA and GEO datasets, the expression matrix of 128 genes was obtained. Then, based on univariate Cox regression analysis, 28 genes with prognostic value were selected from 128 genes (Figures 3A), and 12 genes were screened for later multivariate analysis (Figures 3B, C). We randomly separated 701 MIBC datasets into a training set and a test set. The operation is to randomly allocate them by using R software. There are 351 samples in the training set and 350 samples in the test set. Then, based on 12 genes independently related to OS, a risk model was constructed to evaluate the prognostic risk of MIBC patients in the training set, verified it in the test set, and divided the set into high- and low-risk groups according to the median prognostic risk value. The survival and prognosis of patients in the high-risk group were worse (Figures 4(a) and 4(b)). Figures 4(c) and 4(d), respectively, demonstrate the risk level distribution, survival time, survival status, and expression criteria of 12 genes in the training and test sets for the high-risk and low-risk groups. From them, it can be found that except SLC7A2 and MST1R are protective genes (low expression in the high-risk group), the other 10 genes such as CDK6, NXPH4, GRIK2, TRIB3, PBK, ABCA4, FBN2, SCG2 AND ELN, and INCENP were pathogenic gene genes. Time-dependent ROC analysis showed that in the training cohort, the AUC of OS predicted by risk score was 0.718 at 1 year, 0.716 at 2 years, and 0.733 at 3 years (Figure 4(e)), and the AUC of the test cohort was 0.667 at 1 year, 0.642 at 2 years, 0.636 at 3 years, and 0.660 (Figure 4(f)). This finding suggests that the model may be more useful in determining the one-year survival rate of MIBC patients. Simultaneously, we calculated each patient's risk score to further verify the model's prognostic ability based on the unified formula (supplement table 6).

### 3.4. Landscape of Genetic Variation of Risk Factors

A total of 12 genes were identified, and a risk regression model was successfully built. The copy number variation of the risk factors, as well as the incidence of somatic mutations in BC, was then summarized. Among 412 samples, 88 genes had gene mutations, with a frequency of 21.33%. The investigation of CNV alternation frequency shows that CNV changes are common in 12 genes, most of which focus on the deletion of copy number, while INCENP, NXPH4, CDK6, and SLC27A2 all have a wide range of CNV amplification frequencies (Figure 4(g)). The location of CNV changes in the 12 genes on the chromosome is depicted in Figure 4(h).

### 3.5. Evaluation of MIBC Immune Microenvironment Distribution and Immunotherapy Response Based on the Risk Model

**Figure 5:**
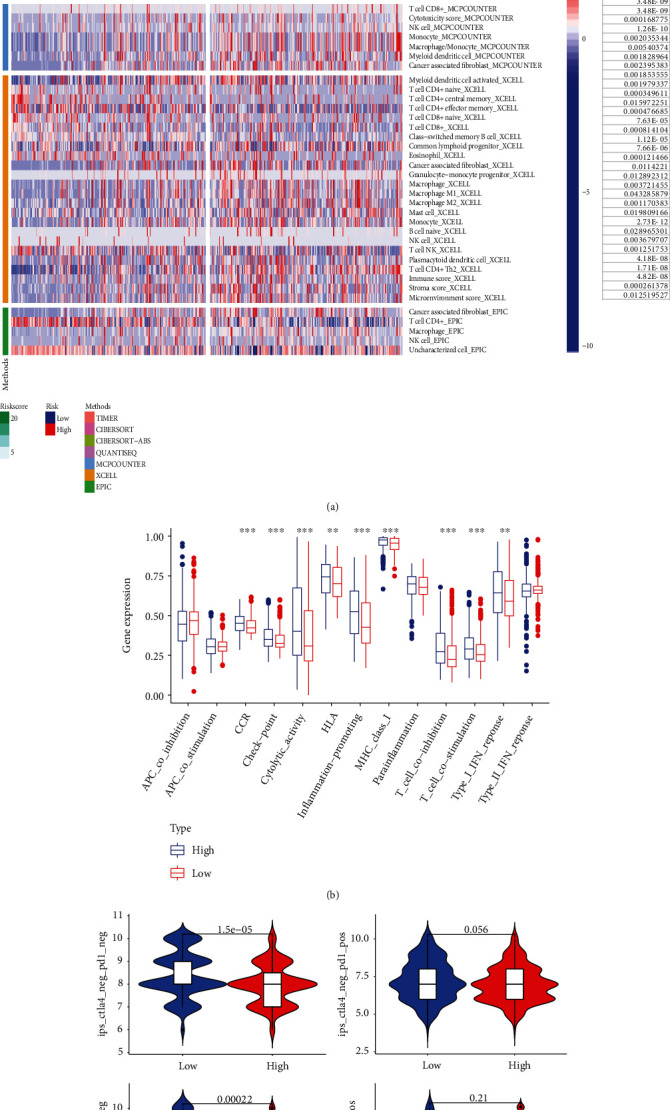
Immune landscape between the high- and low-risk patients with muscle invasive bladder cancer (MIBC). (a) Correlation between immune cells predicted using seven software programs and risk scores (TIMER, CIBERSORT, CIBERSORT-ABS, QUANTISEQ, MCPCOUNTER, XCELL, and EPIC). (b) Analysis of immune function between the high- and low-risk groups. (c) Evaluation of immune response to CTLA4 and PD1 immunosuppressants in MIBC patients in high-risk group and low-risk group.

Firstly, the infection of 404 TCGA queues was estimated in the TIMER2.0 database. The evaluation was based on the use of seven different software programs (CIBERSORT, CIBERSORT-ABS, TIMER, MCPCOUNTER, QUANTISEQ, EPIC, and XCELL). Further investigation was conducted to determine the variations in immune characteristics between the two groups. Compared with the low-risk group, the scores of macrophage (M0, M1, M2) Treg, tumor-associated fibroblasts, immunity, matrix, and microenvironment in the high-risk group were significantly higher, while in the low-risk group, neutrophils, memory CD4 + T cells, and T cell follicular helper cells were the main immune cell types (Figure 5(a)). Then, the immune function distribution (701-GEO-TCGA) of 701 MIBC patients was further analyzed and summarized by GEO and TCHA. The results showed that the immune activity was significantly higher in high-risk groups (Figure 5(b), supplementary table 7). Based on the results of this study, the further analysis combined with the TCIA database revealed that the negative rate of PD-1 inhibitor or PD-1 combined with CTLA-4 inhibitors was low in a high-risk population of invasive bladder cancer, which provided a theoretical basis for clinical drug selection (Figure 5(c), supplementary table 8).

### 3.6. NXPH4 Is a Key Gene in MIBC Occurrence and Immune Regulation

**Figure 6:**
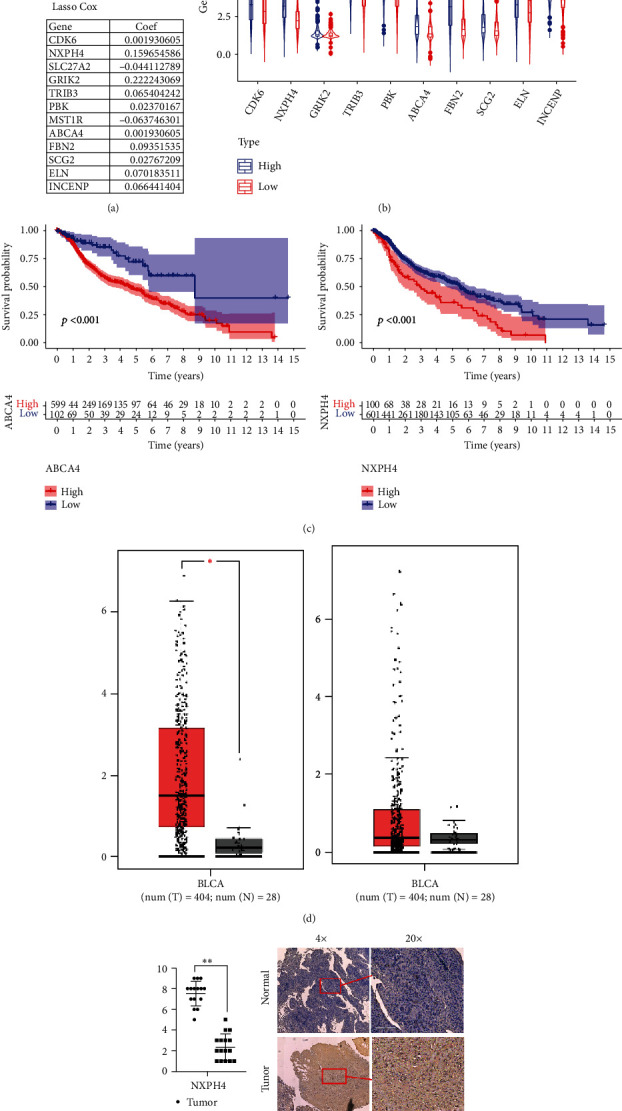
(a) Regression coefficient of 12 genes in risk regression model. (b) NXPH4 and ABCA4 were highly expressed in high-risk groups. (c) NXPH4 and ABCA4 were highly expressed with the worse prognosis. (d) NXPH4 was highly expressed in the MIBC tumor tissues. (e, f) Immunohistochemical results showed that NXPH4 was highly expressed in tumor tissues.

Based on the risk regression coefficient, we further screened out the genes NXPH4 (coef = 0.1597) and ABCA4 (coef = 0.1943) (Figure 6(a)), which were highly expressed in the high-risk group (Figure 6(b)). The survival analysis showed that the high expression of both revealed a poor prognosis (*P* < 0.001, Figure 6(c)). At the same time, NXPH4 increased more significantly in cancer compared to adjacent tissues (*P* < 0.05, Figure 6(d)); NXPH4 was further verified in human paraffin sections with results showing that its expression was significantly increased in tumor tissues (Figure 6(e)).

## 4. Discussion

MIBC is an immunosuppressive urinary malignancy that develops rapidly, has a high metastatic potential, and has a poor prognosis [23]. Numerous research has now thoroughly examined its occurrence, development, and treatment response based on gene, protein, or modification omics [24–26]. The results show that different MIBC molecular subtypes often have different clinical characteristics and prognoses, especially the immune characteristics are often directly related to the treatment or prognosis of patients [27]. Few studies have further identified the molecular subtypes of MIBC patients according to the characteristics of TME, immune checkpoints, and some specific protein molecules or specific modifications, which provide a certain reference for clinical treatment [28–30]. However, most research at the present is focused on the expression of single or multiple genes and proteins to build models and, ultimately, identify molecular subtypes. The level of expression of each gene has a significant impact on the models, and the resulting errors are often larger. Therefore, it may be more accurate to use new algorithms to reduce these errors and establish models from the perspective of overall pathway correlation [31, 32]; however, this study has some limitations, such as the number of randomly assigned patients, and the mechanism of the selected immune pathway in the prognosis and treatment of MIBC is still unclear. However, exploring the role of immune-related pathways in the identification of MIBC molecular subtypes has important guiding significance for follow-up research and the development of MIBC treatment.

In this study, GSVA was performed on the screened MIBC patient dataset based on the immune gene set (GSEA) and obtained the immune-related pathway matrix of 701 MIBC patients, then successfully constructed molecular subtypes based on the matrix by using the CNMF algorithm, and then, analyzed the differential expression pathways among molecular subtypes, screened the key pathways in combination with clinical information, and then, analyzed the pathway genes. A risk regression model was constructed to analyze the differences in immune landscape and immune treatment response between high- and low-risk groups. This study obtained the key pathway: “GSE21670_STAT3_KO_VS_WT_CD4_TCELL_TGFB_IL6_TREATED_DN,” the activation of this pathway means that MIBC patients have a worse prognosis. This pathway comprises 200 genes, 28 of which are prognostic. We successfully built a risk regression model to predict patient survival based on 12 genes. The AUC of OS predicted by the training set risk score is 0.718 at 1 year and that of the test cohort is 0.667 at 1 year, which is higher than other model prediction abilities. Based on this model, it was found that a large number of immune cells are distributed in patients in the high-risk group, and they may be more sensitive to the treatment of PD-1 or CTLA4 immunosuppressants. However, the potential mechanism of action is unclear and will need to be investigated further more in subsequent experiments. Simultaneously, based on this model, we effectively obtained some high-risk genes that may contribute to the progression of MIBC for additional research. Some molecular targets have been discovered. At present, oncology research is not limited to the division between cancer and adjacent cancer [33]. With the continuous progress of sequencing technology, the identification of new and more accurate molecular subtypes by using technologies such as RNA sequencing, protein mass spectrometry, DNA sequencing, single-cell sequencing, and modified omics sequencing is developing rapidly [34, 35]. In MIBC, there are a large number of studies on the division of subtypes based on different levels. Gordon Robertson used the nonnegative matrix factorization approach to cluster somatic mutations and site copy number changes in 125 MIBC samples [36]. The results showed that there were three different subtypes of high-grade myometrial invasive bladder tumors: subtype A was “centralized amplification,” subtype B was “mastoid CDKN2A deletion FGFR3 mutation,” and subtype C was “TP53/cell cycle mutant.” Wu et al. went on to identify 99 DEIGs based on TP53 mutation status, design and validate TIPS such as ORM1, PTHLH, and CTSE, and effectively construct prediction models in TCGA and GEO databases to identify poor high-risk prognosis groups [37]. However, the identification of MIBC subtypes is still based on RNA sequencing data. Among them, Eur Urol magazine published a large-scale META analysis of the National University of Singapore research team, which included 2411 specific tumors, including nonmuscle invasive (NMIBC) and myometrial invasive bladder cancer (MIBC); six molecular subtypes with different overall survival (OS) and molecular characteristics were determined in this study: subtype neural-like (median OS, 87 Mo), HER2-like (107.7 Mo), papillary-like (>135 Mo), lunar-like (91.7 Mo), mesenchymal-like (MES; 86.6 Mo), and squamous cell carcinoma-like (SCC; 20.6 Mo). It is also suggested that NMIBC with a high risk of progression can show the molecular characteristics of MIBC, which provides an important reference for the subtype analysis of MIBC [38]. Kamoun et al. published an article in the European Urology magazine that study according to the sequencing data of 1750 MIBC transcripts from 16 published datasets and two other queues; six molecular subtypes can be identified: luminal papillary (24%), luminal nonspecific (8%), luminal unstable (15%), stroma rich (15%), basic/square (35%), and neuroendocrine like (3%). Simultaneously, the study discovered that these consensus categories varied in terms of potential carcinogenic mechanism, immune and stromal cell infiltration, and histological and clinical features, which has made great progress compared with the previous simple classification of lumen type of basal type [39]. With the deepening of the research on various pathways, their important functions in the process of tumorigenesis and development are increasingly reflected. Therefore, several studies on distinguishing tumor subtypes based on classical pathways have begun to emerge. Studies based on autophagy, apoptosis, ferroptosis, necrosis, and even m6A methylation modification have been reported [40–44], which are of certain research significance. It is also common to classify based on immune genes, but these studies are based on single or multiple genes [45], which are very dependent on the expression of a gene, and there are some limitations. Under such conditions, our study used GSVA to assess the immune pathway score in the entire dataset and then identified the subtype based on the pathway score, reducing the effect of a single gene on the typing results and obtaining the key pathways and key genes. Although the potential mechanism cannot be further analyzed in this study, this method is still used in the identification of MIBC subtypes for the first time, and this method can be used to further analyze the potential functions of other pathways in the identification of MIBC subtypes, it is considered that establishing the model from the perspective of the overall pathway has a strong research prospect.

In addition, based on the risk regression model, this study obtained 10 high-risk genes that may cause disease, CDK6, NXPH4, GRIK2, TRIB3, PBK, ABCA4, FBN2, SCG2, ELN, and INCENP (highly expressed in the high-risk group). CDK6 and PBK were reported in the literature, which was associated with the progress of MIBC. Steele et al.'s study showed obatoclax (BH3 simulant) can reduce the expression levels of cyclin D1 and CDK4/6, inhibit cell proliferation, and promote apoptosis, and may significantly enhance the therapeutic efficacy of cisplatin in MIBC cells through this mechanism [46]. According to Singh et al.'s study, immunohistochemical (IHC) expression of PBK/TOPK (*P* < 0.001) is significantly related to MIBC grade, and the protein is specific for human BC, suggesting that it might be used as a potential target for the development of cancer immunotherapy and diagnostic biomarkers [47]. The genes GRIK2 and ELN have been linked to BLCA. Inoue et al. studies have shown that overexpression of GRIK2 can increase the ability of urothelial carcinogenesis, invasion, and tumorigenicity and play a role in the maintenance of CSCs/CICS; immunohistochemical staining further showed that higher levels of GRIK2 and ALDH1 expression were associated with poor prognosis of urinary tract cancer cases [48]. Jiang et al.'s results showed that SATB1, TTLL7, SREBF1, ELN, DSC2, DIP2C, hsa-mir-29c-3p, and hsa-mir-20A-5P were identified as independent prognostic factors of BLCA [49]. The remaining genes have been identified in other tumors and are critical in tumor progression. By suppressing FOXO1 degradation and increasing SOX2 transcription, TRIB3 can help breast cancer [50]. FBN2 exposure in tumor endothelium can affect TGF-*β* by affecting microfibril isolation which directly stimulates tumor angiogenesis, resulting in higher local activity of TGF in the tumor microenvironment concentration [51]. Few other genes have also been reported to varying degrees, fully reflecting their potential as a new molecular target in MIBC screening or molecular therapy. Among them, NXPH4 and ABCA4, as the genes with the heaviest regression coefficient, both suggest a worse prognosis, and NXPH4 is also significantly increased in MIBC tumor tissues. NXPH4 (neurexophilin 4) is a protein coding gene, which may be a signal molecule similar to binding to alpha-neurexins and possibly other receptors [52]. Current studies show that EZH2/NXPH4/CDKN2A axis can participate in regulating the proliferation and migration of non-small-cell lung cancer cells [18] revealing a poor prognosis in the prognosis model of breast cancer [53]. It is consistent with the results of this study, and this study shows that NXPH4 not only has the ability to promote tumorigenesis but also may have certain value in tumor immune regulation. However, this study does not deeply reveal the mechanism, but only preliminarily proves the expression level of this gene in MIBC, which still needs further exploration at the cellular and animal levels.

## 5. Conclusion

This study used the GSVA algorithm to evaluate the MIBC sequencing matrix in GEO and TCGA databases for the first time, successfully constructed a new molecular subtype and risk regression model, and obtained key pathways and potential molecular therapeutic targets. This can provide a selection basis for clinical treatment, especially PD-1 and CTLA4 immunosuppressant treatment, and might aid in the subsequent establishment of the model from the perspective of overall pathways.

## Data Availability

The datasets generated and/or analysed during the current study are available in the [GSE13507] repository [https://www.ncbi.nlm.nih.gov/geo/query/acc.cgi?acc=GSE13507]; in [GSE31684] repository [https://www.ncbi.nlm.nih.gov/geo/query/acc.cgi?acc=GSE31684]; in [GSE32894] repository [https://www.ncbi.nlm.nih.gov/geo/query/acc.cgi?acc=GSE32894]; and in [GSE21670] repository [https://www.ncbi.nlm.nih.gov/geo/query/acc.cgi?acc=GSE21670].

## Supplementary Materials

Supplementary Materials Figure s1: article roadmap of the whole research. Figure s2: (A) GSVA results heatmap of invasive bladder cancer in TCGA database (normal =19, tumor =404); Wayne diagram of differential pathways between clusters. (B) Wayne diagram in TCGA clusters (*n* = 65). (C) Wayne diagram in GEO clusters (*n* = 463). (D) Wayne diagram in TCGA clusters and GEO clusters (*n* = 6). Figure s3: (A) the 28 prognostic key pathway genes (*P* <0.01). Risk model for patients with muscle invasive bladder cancer (MIBC) based on 12 genes (SLC7A2, MST1R, CDK6, NXPH4, GRIK2, TRIB3, PBK, ABCA4, FBN2, SCG2, ELN, and INCENP). (B) LASSO regression with 10-fold crossvalidation was used to obtain 12 prognostic genes with an error within one standard error of the minimum (lambda.1se). (C) LASSO coefficient profiles of 28 key pathway genes. Supplement Table 1: clinical characteristics such as N, M, T, tumor grade, and stage, including age among the three groups (TCGA). Supplement Table 2: survival and prognosis information of three groups based on GEO. Supplement Table 3: 65 differential pathways were obtained from the molecular subtypes of TCGA queue. Supplement Table 4: 6 common differential pathways were obtained based on 65 TCGA, differential pathways, and 463 GEO, differential pathways. Supplement Table 5: 6 common differential pathways with prognosis. Supplement Table 6: the risk model based on the 12 prognostic genes in TCGA and GEO databases. Supplement Table 7: immune landscape between the high- and low-risk patients with muscle invasive bladder cancer (MIBC). Supplement Table 8: evaluation of immune response to CTLA4 and PD1 immunosuppressants in MIBC patients.
