# Supplementary material for: NXPH4 Used as a New Prognostic and Immunotherapeutic Marker for Muscle-Invasive Bladder Cancer
Source: J Oncol. 2022 Oct 4;2022:4271409. doi: 10.1155/2022/4271409 (PMC9553512; doi:10.1155/2022/4271409)
Supplement: Supplementary Materials — Figure s1: article roadmap of the whole research. Figure s2: (A) GSVA results heatmap of invasive bladder cancer in TCGA database (normal =19, tumor =404); Wayne diagram of differential pathways between clusters. (B) Wayne diagram in TCGA clusters (n = 65). (C) Wayne diagram in GEO clusters (n = 463). (D) Wayne diagram in TCGA clusters and GEO clusters (n = 6). Figure s3: (A) the 28 prognostic key pathway genes (P <0.01). Risk model for patients with muscle invasive bladder cancer (MIBC) based on 12 genes (SLC7A2, MST1R, CDK6, NXPH4, GRIK2, TRIB3, PBK, ABCA4, FBN2, SCG2, ELN, and INCENP). (B) LASSO regression with 10-fold crossvalidation was used to obtain 12 prognostic genes with an error within one standard error of the minimum (lambda.1se). (C) LASSO coefficient profiles of 28 key pathway genes. Supplement Table 1: clinical characteristics such as N, M, T, tumor grade, and stage, including age among the three groups (TCGA). Supplement Table 2: survival and prognosis information of three groups based on GEO. Supplement Table 3: 65 differential pathways were obtained from the molecular subtypes of TCGA queue. Supplement Table 4: 6 common differential pathways were obtained based on 65 TCGA, differential pathways, and 463 GEO, differential pathways. Supplement Table 5: 6 common differential pathways with prognosis. Supplement Table 6: the risk model based on the 12 prognostic genes in TCGA and GEO databases. Supplement Table 7: immune landscape between the high- and low-risk patients with muscle invasive bladder cancer (MIBC). Supplement Table 8: evaluation of immune response to CTLA4 and PD1 immunosuppressants in MIBC patients. [file 4271409.f1.zip › supplement table1.docx]

**Supplement Table1** Clinical characteristics such as N, M, T, tumor grade, and stage, including age among the three groups (TCGA)

| cluster-404(MIBC-TCGA) | | clinical-MIBC(TCGA) | | | | | | | |
| --- | --- | --- | --- | --- | --- | --- | --- | --- | --- |
| ID | Cluster | Id | Age | Gender | Grade | Stage | T | M | N |
| TCGA-FD-A6TF | C2 | TCGA-CF-A3MF | <=65 | MALE | G1 | Stage III | T3 | M0 | N0 |
| TCGA-XF-AAMR | C2 | TCGA-CF-A47S | <=65 | MALE | G1 | Stage II | T2 | M0 | N0 |
| TCGA-XF-A8HF | C3 | TCGA-CF-A47W | <=65 | MALE | G1 | Stage II | T2 | M0 | N0 |
| TCGA-ZF-AA4U | C3 | TCGA-CF-A3MG | <=65 | MALE | G1 | Stage II | T2 | M0 | N0 |
| TCGA-ZF-AA54 | C2 | TCGA-E7-A8O7 | <=65 | MALE | G1 | Stage II | T2 | M0 | N0 |
| TCGA-GU-A766 | C2 | TCGA-CF-A7I0 | <=65 | MALE | G1 | Stage II | T2 | M0 | N0 |
| TCGA-BL-A13I | C2 | TCGA-CF-A47Y | <=65 | MALE | G1 | Stage II | T2 | M0 | N0 |
| TCGA-XF-AAML | C1 | TCGA-CF-A47T | <=65 | FEMALE | G1 | Stage II | T2 | M0 | N0 |
| TCGA-ZF-AA58 | C2 | TCGA-CF-A5U8 | <=65 | MALE | G1 | Stage II | T2 | M0 | N0 |
| TCGA-G2-AA3B | C1 | TCGA-CF-A47X | <=65 | MALE | G1 | Stage II | T2 | M0 | N0 |
| TCGA-K4-A54R | C2 | TCGA-CF-A3MI | <=65 | MALE | G1 | Stage II | T2 | M0 | N0 |
| TCGA-DK-A1AA | C1 | TCGA-CF-A5UA | >65 | MALE | G1 | Stage II | T2 | M0 | N0 |
| TCGA-CU-A5W6 | C3 | TCGA-E7-A3X6 | >65 | MALE | G1 | Stage II | T2 | M0 | NX |
| TCGA-DK-AA6R | C2 | TCGA-CF-A3MH | >65 | MALE | G1 | Stage II | T2 | M0 | N0 |
| TCGA-BT-A3PH | C3 | TCGA-CF-A9FH | >65 | MALE | G1 | Stage II | T2 | M0 | N0 |
| TCGA-DK-A3IQ | C2 | TCGA-CF-A47V | <=65 | MALE | G2 | Stage II | T2 | M0 | N0 |
| TCGA-E7-A4XJ | C3 | TCGA-CF-A9FF | <=65 | MALE | G2 | Stage II | T2 | M0 | N0 |
| TCGA-XF-A9T6 | C3 | TCGA-ZF-A9R3 | <=65 | FEMALE | G2 | Stage II | T2 | M0 | NX |
| TCGA-ZF-AA5H | C2 | TCGA-CF-A8HX | <=65 | FEMALE | G2 | Stage II | T2 | M0 | N0 |
| TCGA-DK-A3WX | C2 | TCGA-ZF-AA4X | <=65 | MALE | G2 | Stage II | T2 | M0 | NX |
| TCGA-YF-AA3L | C1 | TCGA-CF-A8HY | <=65 | MALE | G2 | Stage II | T2 | M0 | N0 |
| TCGA-BT-A2LA | C3 | TCGA-E7-A8O8 | <=65 | MALE | G2 | Stage II | T2 | M0 | N0 |
| TCGA-4Z-AA87 | C1 | TCGA-ZF-AA53 | <=65 | MALE | G2 | Stage II | T2 | M0 | NX |
| TCGA-BT-A2LB | C2 | TCGA-DK-AA6M | <=65 | FEMALE | G2 | Stage II | T2 | M0 | N0 |
| TCGA-XF-A8HB | C3 | TCGA-E7-A4XJ | >65 | MALE | G2 | Stage II | T2 | M0 | N0 |
| TCGA-DK-A2I6 | C3 | TCGA-ZF-AA51 | >65 | FEMALE | G2 | Stage II | T2 | M0 | NX |
| TCGA-ZF-AA4X | C1 | TCGA-E7-A97P | >65 | MALE | G2 | Stage II | T2 | M0 | NX |
| TCGA-BT-A20P | C3 | TCGA-GV-A3JW | >65 | MALE | G2 | Stage II | T2 | MX | NX |
| TCGA-CF-A3MF | C1 | TCGA-G2-AA3B | >65 | FEMALE | G2 | Stage II | T2 | M0 | N0 |
| TCGA-CF-A47T | C3 | TCGA-UY-A78O | >65 | FEMALE | G2 | Stage II | T2 | MX | N0 |
| TCGA-YC-A8S6 | C2 | TCGA-DK-AA6W | >65 | MALE | G2 | Stage II | T2 | M0 | N0 |
| TCGA-DK-A3IS | C3 | TCGA-UY-A78P | >65 | FEMALE | G2 | Stage II | T2 | MX | N0 |
| TCGA-XF-A9SJ | C2 | TCGA-KQ-A41R | >65 | FEMALE | G2 | Stage II | T2 | MX | N0 |
| TCGA-DK-AA6T | C1 | TCGA-ZF-A9R4 | >65 | MALE | G2 | Stage II | T2 | M0 | NX |
| TCGA-E7-A5KE | C3 | TCGA-ZF-AA5N | <=65 | FEMALE | G2 | Stage IV | T2 | M1 | NX |
| TCGA-ZF-AA4V | C3 | TCGA-GU-AATP | >65 | MALE | G2 | Stage IV | T2 | MX | N2 |
| TCGA-ZF-AA53 | C2 | TCGA-UY-A78N | >65 | MALE | G2 | Stage IV | T2 | MX | N1 |
| TCGA-CU-A72E | C3 | TCGA-UY-A78M | >65 | FEMALE | G2 | Stage IV | T2 | MX | N2 |
| TCGA-FD-A62O | C3 | TCGA-CU-A0YR | >65 | MALE | G2 | Stage IV | T2 | M0 | N2 |
| TCGA-BL-A3JM | C3 | TCGA-E7-A6MF | <=65 | MALE | G1 | Stage II | T2 | M0 | NX |
| TCGA-FJ-A3ZF | C1 | TCGA-E7-A5KF | >65 | MALE | G1 | Stage II | T2 | M0 | N0 |
| TCGA-CU-A0YO | C2 | TCGA-4Z-AA83 | <=65 | MALE | G2 | Stage II | T2 | M0 | N0 |
| TCGA-CF-A5UA | C3 | TCGA-4Z-AA7W | <=65 | MALE | G2 | Stage II | T2 | M0 | N0 |
| TCGA-K4-AAQO | C2 | TCGA-YF-AA3M | <=65 | MALE | G2 | Stage II | T2 | MX | NX |
| TCGA-ZF-A9R7 | C1 | TCGA-XF-AAMQ | <=65 | FEMALE | G2 | Stage II | T2 | MX | N0 |
| TCGA-FD-A3SO | C2 | TCGA-4Z-AA7Y | <=65 | MALE | G2 | Stage II | T2 | M0 | N0 |
| TCGA-E7-A97P | C2 | TCGA-DK-AA77 | <=65 | MALE | G2 | Stage II | T2 | M0 | N0 |
| TCGA-GU-A767 | C3 | TCGA-GU-A766 | <=65 | MALE | G2 | Stage II | T2 | MX | N0 |
| TCGA-4Z-AA81 | C1 | TCGA-4Z-AA7O | <=65 | MALE | G2 | Stage II | T2 | M0 | N0 |
| TCGA-2F-A9KQ | C3 | TCGA-DK-A6B1 | >65 | MALE | G2 | Stage II | T2 | M0 | N0 |
| TCGA-SY-A9G0 | C2 | TCGA-DK-A3IS | >65 | MALE | G2 | Stage II | T2 | M0 | N0 |
| TCGA-GV-A40G | C3 | TCGA-LT-A8JT | >65 | FEMALE | G2 | Stage II | T2 | M0 | N0 |
| TCGA-XF-A9SL | C2 | TCGA-PQ-A6FI | >65 | MALE | G2 | Stage II | T2 | MX | N0 |
| TCGA-XF-AAN2 | C1 | TCGA-DK-A6AW | >65 | MALE | G2 | Stage II | T2 | M0 | N0 |
| TCGA-FD-A6TD | C2 | TCGA-YC-A8S6 | >65 | MALE | G2 | Stage II | T2 | MX | N0 |
| TCGA-G2-AA3F | C3 | TCGA-4Z-AA80 | >65 | MALE | G2 | Stage II | T2 | M0 | N0 |
| TCGA-FJ-A871 | C3 | TCGA-FD-A43P | >65 | MALE | G2 | Stage II | T2 | MX | N0 |
| TCGA-ZF-A9RN | C1 | TCGA-GV-A40G | >65 | MALE | G2 | Stage II | T2 | MX | N0 |
| TCGA-GC-A3RB | C3 | TCGA-E7-A5KE | >65 | FEMALE | G2 | Stage II | T2 | M0 | N0 |
| TCGA-UY-A9PF | C2 | TCGA-XF-A9SG | >65 | MALE | G2 | Stage II | T2 | MX | N0 |
| TCGA-YC-A89H | C3 | TCGA-DK-A6AV | >65 | FEMALE | G2 | Stage II | T2 | M0 | N0 |
| TCGA-XF-AAMQ | C1 | TCGA-FD-A43X | >65 | MALE | G2 | Stage II | T2 | MX | N0 |
| TCGA-CF-A1HR | C1 | TCGA-DK-A1A6 | <=65 | MALE | G2 | Stage IV | T2 | M0 | N1 |
| TCGA-E7-A3X6 | C1 | TCGA-4Z-AA82 | <=65 | MALE | G2 | Stage IV | T2 | M0 | N1 |
| TCGA-E7-A97Q | C1 | TCGA-HQ-A2OE | >65 | MALE | unknow | unknow | T2 | MX | N2 |
| TCGA-E7-A7DV | C3 | TCGA-E7-A677 | >65 | MALE | G1 | Stage II | T2 | M0 | N0 |
| TCGA-HQ-A2OF | C3 | TCGA-FD-A3N6 | <=65 | FEMALE | G2 | Stage II | T2 | MX | N0 |
| TCGA-XF-AAN1 | C1 | TCGA-YF-AA3L | <=65 | FEMALE | G2 | Stage II | T2 | MX | N0 |
| TCGA-DK-AA6P | C3 | TCGA-XF-A8HB | <=65 | FEMALE | G2 | Stage II | T2 | MX | N0 |
| TCGA-CF-A47Y | C1 | TCGA-GV-A6ZA | <=65 | MALE | G2 | Stage II | T2 | MX | N0 |
| TCGA-CF-A3MI | C1 | TCGA-E7-A4IJ | <=65 | MALE | G2 | Stage II | T2 | M0 | NX |
| TCGA-G2-A2EL | C3 | TCGA-FD-A3B8 | <=65 | MALE | G2 | Stage II | T2 | MX | N0 |
| TCGA-XF-AAMZ | C1 | TCGA-FD-A5BR | <=65 | MALE | G2 | Stage II | T2 | MX | N0 |
| TCGA-DK-AA6L | C1 | TCGA-DK-A3IU | <=65 | MALE | G2 | Stage II | T2 | M0 | N0 |
| TCGA-XF-A9T4 | C2 | TCGA-K4-A54R | <=65 | MALE | G2 | Stage II | T2 | MX | N0 |
| TCGA-K4-A6MB | C3 | TCGA-GC-A3RC | <=65 | MALE | G2 | Stage II | T2 | M0 | N0 |
| TCGA-UY-A8OB | C3 | TCGA-FD-A3NA | <=65 | MALE | G2 | Stage II | T2 | MX | N0 |
| TCGA-ZF-A9R5 | C1 | TCGA-C4-A0F0 | <=65 | MALE | G2 | Stage II | T2 | M0 | N0 |
| TCGA-CU-A3KJ | C3 | TCGA-DK-A6B0 | <=65 | MALE | G2 | Stage II | T2 | M0 | N0 |
| TCGA-DK-AA6S | C2 | TCGA-XF-AAME | <=65 | FEMALE | G2 | Stage II | T2 | MX | N0 |
| TCGA-E7-A85H | C1 | TCGA-XF-A9SH | <=65 | FEMALE | G2 | Stage II | T2 | M0 | N0 |
| TCGA-G2-AA3C | C3 | TCGA-E7-A541 | >65 | MALE | G2 | Stage II | T2 | MX | N0 |
| TCGA-E7-A8O8 | C1 | TCGA-GU-A764 | >65 | MALE | G2 | Stage II | T2 | MX | N0 |
| TCGA-XF-A9SM | C2 | TCGA-UY-A8OD | >65 | FEMALE | G2 | Stage II | T2 | MX | N0 |
| TCGA-DK-A3IN | C2 | TCGA-FD-A3N5 | >65 | MALE | G2 | Stage II | T2 | MX | N0 |
| TCGA-CF-A47W | C1 | TCGA-GC-A3BM | >65 | MALE | G2 | Stage II | T2 | M0 | N0 |
| TCGA-GC-A3I6 | C2 | TCGA-DK-AA71 | >65 | MALE | G2 | Stage II | T2 | M0 | N0 |
| TCGA-FD-A6TA | C2 | TCGA-BT-A20W | >65 | MALE | G2 | Stage II | T2 | M0 | N0 |
| TCGA-KQ-A41P | C3 | TCGA-E5-A4U1 | >65 | MALE | G2 | Stage II | T2 | M0 | N0 |
| TCGA-UY-A9PH | C1 | TCGA-E7-A519 | >65 | MALE | G2 | Stage II | T2 | M0 | NX |
| TCGA-FD-A5BY | C2 | TCGA-XF-AAN2 | >65 | MALE | G2 | Stage II | T2 | MX | N0 |
| TCGA-K4-A3WU | C2 | TCGA-XF-A9SI | >65 | FEMALE | G2 | Stage II | T2 | MX | N0 |
| TCGA-FD-A3SJ | C3 | TCGA-DK-A2I1 | >65 | FEMALE | G2 | Stage II | T2 | M0 | N0 |
| TCGA-HQ-A5NE | C2 | TCGA-UY-A9PH | >65 | MALE | G2 | Stage II | T2 | MX | N0 |
| TCGA-XF-AAMT | C2 | TCGA-E7-A6ME | >65 | MALE | G2 | Stage II | T2 | M0 | NX |
| TCGA-XF-A9SU | C2 | TCGA-ZF-A9R2 | >65 | MALE | G2 | Stage II | T2 | M0 | N0 |
| TCGA-CF-A47S | C1 | TCGA-FD-A3B6 | >65 | MALE | G2 | Stage II | T2 | MX | N0 |
| TCGA-FD-A6TK | C2 | TCGA-K4-A5RJ | >65 | MALE | G2 | Stage II | T2 | MX | N0 |
| TCGA-E7-A5KF | C1 | TCGA-XF-AAML | >65 | MALE | G2 | Stage II | T2 | MX | N0 |
| TCGA-UY-A78L | C3 | TCGA-BT-A20J | >65 | MALE | G2 | Stage II | T2 | MX | N0 |
| TCGA-S5-A6DX | C1 | TCGA-FD-A5BU | >65 | FEMALE | G2 | Stage II | T2 | MX | N0 |
| TCGA-XF-A8HI | C1 | TCGA-FD-A62P | >65 | MALE | G2 | Stage II | T2 | MX | N0 |
| TCGA-XF-A9T5 | C1 | TCGA-K4-A3WV | >65 | FEMALE | G2 | Stage II | T2 | MX | N0 |
| TCGA-XF-A9SX | C2 | TCGA-DK-A1A5 | >65 | MALE | G2 | Stage II | T2 | M0 | N0 |
| TCGA-G2-A2EK | C1 | TCGA-GC-A3OO | >65 | MALE | G2 | Stage II | T2 | M0 | N0 |
| TCGA-DK-A6AW | C1 | TCGA-4Z-AA81 | >65 | MALE | G2 | Stage II | T2 | M0 | N0 |
| TCGA-GC-A3BM | C1 | TCGA-BT-A3PK | >65 | MALE | G2 | Stage II | T2 | MX | N0 |
| TCGA-GV-A6ZA | C1 | TCGA-K4-A4AC | >65 | MALE | G2 | Stage II | T2 | MX | N0 |
| TCGA-G2-A2EJ | C3 | TCGA-2F-A9KT | >65 | MALE | G2 | Stage II | T2 | M0 | N0 |
| TCGA-FD-A43N | C2 | TCGA-GD-A76B | >65 | FEMALE | G2 | Stage II | T2 | MX | N0 |
| TCGA-GV-A3QH | C3 | TCGA-GC-A6I1 | >65 | FEMALE | G2 | Stage II | T2 | MX | N0 |
| TCGA-BT-A0YX | C3 | TCGA-XF-A9T4 | <=65 | MALE | G2 | Stage IV | T2 | MX | N1 |
| TCGA-FT-A3EE | C3 | TCGA-XF-A8HI | <=65 | FEMALE | G2 | Stage IV | T2 | MX | N2 |
| TCGA-ZF-A9RL | C1 | TCGA-CU-A3QU | <=65 | MALE | G2 | Stage IV | T2 | M0 | N1 |
| TCGA-ZF-A9R9 | C2 | TCGA-FD-A3SJ | <=65 | MALE | G2 | Stage IV | T2 | MX | N2 |
| TCGA-FD-A5BX | C2 | TCGA-E5-A2PC | <=65 | FEMALE | G2 | Stage IV | T2 | MX | N1 |
| TCGA-BL-A13J | C2 | TCGA-ZF-AA5P | <=65 | MALE | G2 | Stage IV | T2 | M0 | N2 |
| TCGA-FT-A61P | C2 | TCGA-XF-AAMW | >65 | FEMALE | G2 | Stage IV | T2 | unknow | N0 |
| TCGA-DK-AA77 | C1 | TCGA-DK-A2I6 | >65 | MALE | G2 | Stage IV | T2 | M0 | N1 |
| TCGA-ZF-AA5N | C3 | TCGA-FD-A3B5 | >65 | MALE | G2 | Stage IV | T2 | MX | N1 |
| TCGA-DK-AA6U | C3 | TCGA-UY-A9PE | >65 | MALE | G2 | Stage IV | T2 | MX | N2 |
| TCGA-BT-A20O | C2 | TCGA-E7-A678 | <=65 | MALE | G1 | Stage III | T3 | M0 | N0 |
| TCGA-GD-A3OS | C2 | TCGA-E7-A7DU | >65 | MALE | G1 | Stage III | T3 | M0 | N0 |
| TCGA-XF-A9SZ | C2 | TCGA-CF-A27C | <=65 | MALE | G2 | Stage III | T3 | M0 | N0 |
| TCGA-4Z-AA82 | C2 | TCGA-DK-A3WW | <=65 | MALE | G2 | Stage III | T3 | M0 | N0 |
| TCGA-DK-AA76 | C3 | TCGA-BL-A13I | <=65 | FEMALE | G2 | Stage III | T3 | M0 | N0 |
| TCGA-DK-A3IL | C3 | TCGA-DK-A1AA | <=65 | MALE | G2 | Stage III | T3 | M0 | N0 |
| TCGA-E7-A678 | C1 | TCGA-HQ-A5NE | <=65 | MALE | G2 | Stage III | T3 | M0 | N0 |
| TCGA-ZF-A9R1 | C3 | TCGA-ZF-A9R5 | <=65 | MALE | G2 | Stage III | T3 | M0 | N0 |
| TCGA-XF-A9SH | C1 | TCGA-CF-A1HR | <=65 | MALE | G2 | Stage III | T3 | M0 | N0 |
| TCGA-FD-A5BU | C2 | TCGA-BL-A3JM | <=65 | MALE | G2 | Stage III | T3 | M0 | N0 |
| TCGA-C4-A0F7 | C2 | TCGA-DK-A3IT | <=65 | MALE | G2 | Stage III | T3 | M0 | N0 |
| TCGA-C4-A0F0 | C2 | TCGA-E7-A85H | <=65 | MALE | G2 | Stage III | T3 | M0 | N0 |
| TCGA-FD-A3N5 | C2 | TCGA-DK-A1AG | <=65 | MALE | G2 | Stage III | T3 | M0 | N0 |
| TCGA-4Z-AA7S | C3 | TCGA-E7-A7XN | >65 | MALE | G2 | Stage III | T3 | M0 | N0 |
| TCGA-CF-A3MH | C1 | TCGA-DK-A3WX | >65 | FEMALE | G2 | Stage III | T3 | M0 | N0 |
| TCGA-DK-A6B2 | C2 | TCGA-DK-A3WY | >65 | FEMALE | G2 | Stage III | T3 | M0 | N0 |
| TCGA-4Z-AA86 | C2 | TCGA-ZF-AA54 | >65 | MALE | G2 | Stage III | T3 | MX | NX |
| TCGA-5N-A9KI | C2 | TCGA-DK-A3IQ | >65 | MALE | G2 | Stage III | T3 | M0 | N0 |
| TCGA-GU-AATO | C3 | TCGA-FD-A3B3 | >65 | FEMALE | G2 | Stage III | T3 | MX | N0 |
| TCGA-CF-A9FH | C1 | TCGA-DK-AA74 | >65 | MALE | G2 | Stage III | T3 | M0 | N0 |
| TCGA-FD-A6TG | C2 | TCGA-CF-A1HS | >65 | FEMALE | G2 | Stage III | T3 | M0 | N0 |
| TCGA-4Z-AA7O | C1 | TCGA-DK-A3IM | >65 | MALE | G2 | Stage III | T3 | M0 | N0 |
| TCGA-GV-A3QI | C3 | TCGA-DK-A3X1 | >65 | FEMALE | G2 | Stage III | T3 | M0 | N0 |
| TCGA-DK-A6AV | C3 | TCGA-GC-A3WC | >65 | FEMALE | G2 | Stage III | T3 | MX | N0 |
| TCGA-DK-A1A3 | C2 | TCGA-DK-AA75 | >65 | MALE | G2 | Stage III | T3 | M0 | N0 |
| TCGA-GD-A6C6 | C3 | TCGA-DK-A1AE | >65 | MALE | G2 | Stage III | T3 | M0 | N0 |
| TCGA-GC-A3RD | C3 | TCGA-DK-AA6L | <=65 | MALE | G2 | Stage IV | T3 | MX | N1 |
| TCGA-BT-A20R | C2 | TCGA-DK-A1A3 | <=65 | MALE | G2 | Stage IV | T3 | M0 | N2 |
| TCGA-CF-A8HX | C1 | TCGA-G2-AA3D | <=65 | MALE | G2 | Stage IV | T3 | M0 | N2 |
| TCGA-PQ-A6FI | C3 | TCGA-DK-AA6Q | <=65 | FEMALE | G2 | Stage IV | T3 | M0 | N1 |
| TCGA-FJ-A3Z7 | C2 | TCGA-DK-A2I2 | <=65 | FEMALE | G2 | Stage IV | T3 | M0 | N3 |
| TCGA-FD-A3SP | C2 | TCGA-2F-A9KO | <=65 | MALE | G2 | Stage IV | T3 | M0 | N1 |
| TCGA-CF-A1HS | C3 | TCGA-FD-A6TH | <=65 | MALE | G2 | Stage IV | T3 | MX | N2 |
| TCGA-DK-AA71 | C3 | TCGA-DK-A1A7 | >65 | FEMALE | G2 | Stage IV | T3 | M0 | N2 |
| TCGA-KQ-A41O | C1 | TCGA-DK-A6B2 | >65 | MALE | G2 | Stage IV | T3 | M0 | N1 |
| TCGA-E7-A677 | C1 | TCGA-G2-AA3F | >65 | MALE | G2 | Stage IV | T3 | M0 | N1 |
| TCGA-DK-A1A5 | C2 | TCGA-DK-A3IL | >65 | FEMALE | G2 | Stage IV | T3 | M0 | N2 |
| TCGA-FD-A3B4 | C2 | TCGA-DK-A2HX | >65 | FEMALE | G2 | Stage IV | T3 | M0 | N2 |
| TCGA-DK-A1AB | C2 | TCGA-KQ-A41O | >65 | MALE | G2 | Stage IV | T3 | M0 | N1 |
| TCGA-ZF-AA4N | C2 | TCGA-DK-A1AF | >65 | FEMALE | G2 | Stage IV | T3 | M0 | N2 |
| TCGA-BT-A20X | C2 | TCGA-DK-A3X2 | >65 | MALE | G2 | Stage IV | T3 | M0 | N2 |
| TCGA-GV-A3JW | C3 | TCGA-DK-A3IK | >65 | MALE | G2 | Stage IV | T3 | M0 | N2 |
| TCGA-UY-A78P | C2 | TCGA-GC-A3I6 | <=65 | MALE | G2 | Stage III | T3 | M0 | N0 |
| TCGA-BL-A0C8 | C3 | TCGA-S5-AA26 | <=65 | MALE | G2 | Stage III | T3 | MX | N0 |
| TCGA-BT-A42C | C3 | TCGA-UY-A9PA | <=65 | MALE | G2 | Stage III | T3 | MX | N0 |
| TCGA-G2-A3IB | C3 | TCGA-UY-A9PB | <=65 | MALE | G2 | Stage III | T3 | MX | N0 |
| TCGA-2F-A9KP | C3 | TCGA-BT-A2LA | <=65 | MALE | G2 | Stage III | T3 | M0 | N0 |
| TCGA-DK-A3IT | C2 | TCGA-K4-AAQO | <=65 | MALE | G2 | Stage III | T3 | MX | N0 |
| TCGA-FD-A43P | C1 | TCGA-2F-A9KR | <=65 | FEMALE | G2 | Stage III | T3 | M0 | N0 |
| TCGA-DK-A3IV | C1 | TCGA-CU-A0YN | <=65 | MALE | G2 | Stage III | T3 | M0 | N0 |
| TCGA-2F-A9KW | C3 | TCGA-FD-A6TK | <=65 | MALE | G2 | Stage III | T3 | MX | N0 |
| TCGA-GU-A762 | C2 | TCGA-FD-A5BY | <=65 | FEMALE | G2 | Stage III | T3 | MX | N0 |
| TCGA-XF-AAME | C2 | TCGA-E7-A7PW | <=65 | MALE | G2 | Stage III | T3 | M0 | NX |
| TCGA-XF-AAN0 | C1 | TCGA-GD-A6C6 | <=65 | MALE | G2 | Stage III | T3 | MX | N0 |
| TCGA-DK-A6B1 | C3 | TCGA-4Z-AA7N | <=65 | MALE | G2 | Stage III | T3 | M0 | N0 |
| TCGA-CF-A5U8 | C1 | TCGA-4Z-AA7M | <=65 | MALE | G2 | Stage III | T3 | M0 | N0 |
| TCGA-XF-AAMW | C2 | TCGA-FD-A3B7 | >65 | MALE | G2 | Stage III | T3 | MX | N0 |
| TCGA-BT-A3PJ | C2 | TCGA-K4-A3WS | >65 | MALE | G2 | Stage III | T3 | MX | N0 |
| TCGA-YC-A9TC | C3 | TCGA-K4-A5RI | >65 | FEMALE | G2 | Stage III | T3 | MX | N0 |
| TCGA-GV-A3QG | C2 | TCGA-2F-A9KQ | >65 | MALE | G2 | Stage III | T3 | M0 | N0 |
| TCGA-K4-A6FZ | C2 | TCGA-G2-A2EO | >65 | MALE | G2 | Stage III | T3 | M0 | N0 |
| TCGA-KQ-A41R | C1 | TCGA-K4-A5RH | >65 | MALE | G2 | Stage III | T3 | MX | N0 |
| TCGA-DK-AA6M | C2 | TCGA-R3-A69X | >65 | MALE | G2 | Stage III | T3 | M0 | N0 |
| TCGA-ZF-AA52 | C2 | TCGA-BT-A20U | >65 | FEMALE | G2 | Stage III | T3 | M0 | N0 |
| TCGA-K4-A5RJ | C1 | TCGA-ZF-AA52 | >65 | MALE | G2 | Stage III | T3 | MX | NX |
| TCGA-HQ-A5ND | C3 | TCGA-GC-A4ZW | >65 | MALE | G2 | Stage III | T3 | M0 | N0 |
| TCGA-BT-A3PK | C2 | TCGA-BT-A20N | >65 | MALE | G2 | Stage III | T3 | MX | N0 |
| TCGA-FD-A3N6 | C3 | TCGA-BT-A2LB | >65 | FEMALE | G2 | Stage III | T3 | M0 | N0 |
| TCGA-CF-A47X | C1 | TCGA-BT-A42E | >65 | MALE | G2 | Stage III | T3 | M0 | N0 |
| TCGA-ZF-AA51 | C1 | TCGA-MV-A51V | >65 | MALE | G2 | Stage III | T3 | M0 | N0 |
| TCGA-UY-A78O | C1 | TCGA-BT-A20O | >65 | MALE | G2 | Stage III | T3 | MX | N0 |
| TCGA-GC-A3OO | C2 | TCGA-K4-A6FZ | >65 | FEMALE | G2 | Stage III | T3 | MX | N0 |
| TCGA-FD-A43Y | C2 | TCGA-FD-A43N | >65 | MALE | G2 | Stage III | T3 | MX | N0 |
| TCGA-S5-AA26 | C1 | TCGA-ZF-A9RC | >65 | MALE | G2 | Stage III | T3 | MX | N0 |
| TCGA-BT-A42E | C1 | TCGA-XF-A8HD | >65 | MALE | G2 | Stage III | T3 | MX | N0 |
| TCGA-FD-A43X | C1 | TCGA-FD-A6TD | >65 | MALE | G2 | Stage III | T3 | MX | N0 |
| TCGA-ZF-AA4R | C2 | TCGA-PQ-A6FN | >65 | FEMALE | G2 | Stage III | T3 | MX | N0 |
| TCGA-BT-A20T | C2 | TCGA-4Z-AA7Q | >65 | MALE | G2 | Stage III | T3 | M0 | NX |
| TCGA-E7-A7DU | C1 | TCGA-UY-A9PD | >65 | MALE | G2 | Stage III | T3 | MX | N0 |
| TCGA-DK-A3WY | C1 | TCGA-XF-A8HF | >65 | MALE | G2 | Stage III | T3 | M0 | N0 |
| TCGA-G2-A2EF | C1 | TCGA-BT-A20P | >65 | FEMALE | G2 | Stage III | T3 | M0 | N0 |
| TCGA-C4-A0F6 | C3 | TCGA-FD-A6TB | >65 | MALE | G2 | Stage III | T3 | MX | N0 |
| TCGA-CF-A7I0 | C1 | TCGA-GC-A3RD | >65 | FEMALE | G2 | Stage III | T3 | M0 | N0 |
| TCGA-FD-A3B6 | C2 | TCGA-GD-A2C5 | <=65 | FEMALE | G2 | Stage IV | T3 | MX | N2 |
| TCGA-GC-A3YS | C2 | TCGA-FD-A6TE | <=65 | MALE | G2 | Stage IV | T3 | MX | N2 |
| TCGA-XF-A9T2 | C3 | TCGA-BT-A42B | <=65 | MALE | G2 | Stage IV | T3 | MX | N1 |
| TCGA-XF-A9T0 | C3 | TCGA-XF-AAN7 | <=65 | MALE | G2 | Stage IV | T3 | MX | N1 |
| TCGA-YF-AA3M | C3 | TCGA-FD-A5C0 | <=65 | MALE | G2 | Stage IV | T3 | MX | N2 |
| TCGA-4Z-AA7Y | C3 | TCGA-GC-A3YS | <=65 | MALE | G2 | Stage IV | T3 | MX | N1 |
| TCGA-E7-A6ME | C3 | TCGA-ZF-AA58 | <=65 | FEMALE | G2 | Stage IV | T3 | MX | N2 |
| TCGA-XF-AAN5 | C2 | TCGA-4Z-AA84 | <=65 | MALE | G2 | Stage IV | T3 | M1 | N2 |
| TCGA-FD-A3SN | C2 | TCGA-FD-A3SQ | <=65 | MALE | G2 | Stage IV | T3 | MX | N2 |
| TCGA-ZF-A9R2 | C3 | TCGA-UY-A8OB | <=65 | MALE | G2 | Stage IV | T3 | MX | N1 |
| TCGA-ZF-AA4T | C3 | TCGA-GV-A3QG | <=65 | MALE | G2 | Stage IV | T3 | MX | N3 |
| TCGA-E7-A4IJ | C1 | TCGA-4Z-AA86 | >65 | MALE | G2 | Stage IV | T3 | M0 | N1 |
| TCGA-FD-A6TE | C1 | TCGA-2F-A9KP | >65 | MALE | G2 | Stage IV | T3 | MX | N2 |
| TCGA-H4-A2HO | C1 | TCGA-ZF-AA4R | >65 | MALE | G2 | Stage IV | T3 | MX | N1 |
| TCGA-FD-A6TB | C2 | TCGA-FD-A3SO | >65 | MALE | G2 | Stage IV | T3 | MX | N1 |
| TCGA-XF-AAN3 | C2 | TCGA-C4-A0EZ | >65 | FEMALE | G2 | Stage IV | T3 | M1 | N1 |
| TCGA-XF-AAMH | C3 | TCGA-XF-A9SL | >65 | MALE | G2 | Stage IV | T3 | MX | N2 |
| TCGA-FD-A3B8 | C2 | TCGA-FD-A3SM | >65 | MALE | G2 | Stage IV | T3 | M1 | N2 |
| TCGA-XF-A9SY | C2 | TCGA-GU-A42P | >65 | MALE | G2 | Stage IV | T3 | M0 | N1 |
| TCGA-CF-A9FF | C1 | TCGA-FD-A6TG | >65 | MALE | G2 | Stage IV | T3 | MX | N2 |
| TCGA-XF-A9ST | C3 | TCGA-FD-A62O | >65 | MALE | G2 | Stage IV | T3 | MX | N2 |
| TCGA-DK-A6B5 | C3 | TCGA-ZF-A9RD | >65 | FEMALE | G2 | Stage IV | T3 | MX | N2 |
| TCGA-GC-A3RC | C2 | TCGA-UY-A9PF | >65 | MALE | G2 | Stage IV | T3 | MX | N2 |
| TCGA-GV-A3QK | C1 | TCGA-FD-A5BZ | >65 | FEMALE | G2 | Stage IV | T3 | MX | N1 |
| TCGA-GU-A764 | C2 | TCGA-BT-A2LD | >65 | FEMALE | G2 | Stage IV | T3 | M0 | N1 |
| TCGA-GU-A42R | C3 | TCGA-XF-A9T5 | >65 | FEMALE | G2 | Stage IV | T3 | MX | N1 |
| TCGA-DK-A6B6 | C1 | TCGA-XF-A8HC | >65 | MALE | G2 | Stage IV | T3 | MX | N2 |
| TCGA-R3-A69X | C2 | TCGA-XF-AAMZ | >65 | FEMALE | G2 | Stage IV | T3 | MX | N2 |
| TCGA-5N-A9KM | C1 | TCGA-CU-A0YO | >65 | MALE | G2 | Stage IV | T3 | MX | N2 |
| TCGA-4Z-AA7M | C3 | TCGA-GC-A6I3 | >65 | FEMALE | G2 | Stage IV | T3 | MX | N1 |
| TCGA-ZF-AA56 | C2 | TCGA-G2-A2ES | >65 | MALE | G2 | Stage II | T3 | M0 | N0 |
| TCGA-XF-A8HH | C2 | TCGA-GV-A3QI | <=65 | MALE | G2 | Stage III | T3 | MX | N0 |
| TCGA-C4-A0EZ | C3 | TCGA-XF-A8HE | <=65 | MALE | G2 | Stage III | T3 | MX | N0 |
| TCGA-UY-A8OD | C2 | TCGA-FD-A5BV | <=65 | FEMALE | G2 | Stage III | T3 | MX | N0 |
| TCGA-2F-A9KT | C3 | TCGA-XF-AAMR | <=65 | MALE | G2 | Stage III | T3 | MX | N0 |
| TCGA-BT-A20Q | C2 | TCGA-FJ-A871 | <=65 | MALE | G2 | Stage III | T3 | MX | NX |
| TCGA-CU-A0YR | C2 | TCGA-HQ-A2OF | <=65 | FEMALE | G2 | Stage III | T3 | M0 | N0 |
| TCGA-SY-A9G5 | C2 | TCGA-XF-A9T2 | <=65 | MALE | G2 | Stage III | T3 | MX | N0 |
| TCGA-4Z-AA84 | C3 | TCGA-GC-A3RB | <=65 | MALE | G2 | Stage III | T3 | M0 | N0 |
| TCGA-4Z-AA7Q | C1 | TCGA-ZF-AA4W | <=65 | MALE | G2 | Stage III | T3 | MX | N0 |
| TCGA-FD-A5BT | C2 | TCGA-GV-A3JX | <=65 | MALE | G2 | Stage III | T3 | MX | N0 |
| TCGA-CF-A27C | C1 | TCGA-XF-A9SP | <=65 | MALE | G2 | Stage III | T3 | MX | N0 |
| TCGA-E7-A6MF | C1 | TCGA-FD-A62S | <=65 | FEMALE | G2 | Stage III | T3 | MX | N0 |
| TCGA-DK-A3WW | C1 | TCGA-DK-AA6S | <=65 | MALE | G2 | Stage III | T3 | M0 | N0 |
| TCGA-XF-AAMY | C2 | TCGA-FD-A3SP | <=65 | MALE | G2 | Stage III | T3 | MX | N0 |
| TCGA-K4-A3WS | C2 | TCGA-XF-AAN5 | <=65 | FEMALE | G2 | Stage III | T3 | MX | N0 |
| TCGA-UY-A9PA | C1 | TCGA-FD-A5C1 | <=65 | FEMALE | G2 | Stage III | T3 | MX | N0 |
| TCGA-2F-A9KR | C3 | TCGA-XF-A9T8 | <=65 | MALE | G2 | Stage III | T3 | MX | N0 |
| TCGA-E5-A4U1 | C1 | TCGA-ZF-AA4V | >65 | MALE | G2 | Stage III | T3 | M0 | N0 |
| TCGA-E7-A3Y1 | C1 | TCGA-ZF-A9RN | >65 | FEMALE | G2 | Stage III | T3 | M0 | NX |
| TCGA-FD-A6TC | C2 | TCGA-GU-A42Q | >65 | MALE | G2 | Stage III | T3 | M0 | N0 |
| TCGA-FD-A5BV | C3 | TCGA-2F-A9KW | >65 | FEMALE | G2 | Stage III | T3 | MX | N0 |
| TCGA-DK-A2I4 | C2 | TCGA-FD-A5BS | >65 | MALE | G2 | Stage III | T3 | MX | N0 |
| TCGA-UY-A78M | C3 | TCGA-XF-A9ST | >65 | MALE | G2 | Stage III | T3 | MX | N0 |
| TCGA-4Z-AA7R | C3 | TCGA-GU-AATQ | >65 | MALE | G2 | Stage III | T3 | MX | N0 |
| TCGA-FD-A62P | C2 | TCGA-XF-A9T0 | >65 | MALE | G2 | Stage III | T3 | MX | N0 |
| TCGA-CF-A47V | C1 | TCGA-XF-A8HG | >65 | MALE | G2 | Stage III | T3 | MX | N0 |
| TCGA-XF-A9SI | C1 | TCGA-FD-A62N | >65 | MALE | G2 | Stage III | T3 | MX | N0 |
| TCGA-GU-A42Q | C3 | TCGA-ZF-A9RG | >65 | FEMALE | G2 | Stage III | T3 | MX | N0 |
| TCGA-BT-A42F | C1 | TCGA-BT-A0YX | >65 | FEMALE | G2 | Stage III | T3 | M0 | N0 |
| TCGA-FD-A3B5 | C2 | TCGA-XF-AAMJ | >65 | MALE | G2 | Stage III | T3 | M0 | N0 |
| TCGA-DK-A1A7 | C1 | TCGA-C4-A0F1 | >65 | MALE | G2 | Stage III | T3 | M0 | N0 |
| TCGA-FD-A3SR | C2 | TCGA-FD-A43S | >65 | FEMALE | G2 | Stage III | T3 | MX | N0 |
| TCGA-GU-AATP | C2 | TCGA-DK-A1AC | >65 | MALE | G2 | Stage III | T3 | M0 | N0 |
| TCGA-DK-AA6X | C1 | TCGA-KQ-A41N | >65 | MALE | G2 | Stage III | T3 | M0 | N0 |
| TCGA-XF-A9T3 | C2 | TCGA-XF-AAN8 | >65 | FEMALE | G2 | Stage III | T3 | MX | N0 |
| TCGA-PQ-A6FN | C3 | TCGA-CU-A3KJ | >65 | MALE | G2 | Stage III | T3 | M0 | N0 |
| TCGA-E7-A519 | C1 | TCGA-BT-A3PJ | >65 | MALE | G2 | Stage III | T3 | M0 | N0 |
| TCGA-DK-A3IU | C2 | TCGA-XF-A9SM | >65 | MALE | G2 | Stage III | T3 | MX | N0 |
| TCGA-FD-A3SM | C2 | TCGA-XF-AAN4 | >65 | FEMALE | G2 | Stage III | T3 | MX | N0 |
| TCGA-BL-A5ZZ | C2 | TCGA-XF-AAMY | >65 | MALE | G2 | Stage III | T3 | MX | N0 |
| TCGA-FD-A62N | C2 | TCGA-FD-A3SN | >65 | MALE | G2 | Stage III | T3 | MX | N0 |
| TCGA-BT-A20J | C2 | TCGA-DK-A2I4 | >65 | MALE | G2 | Stage III | T3 | M0 | N0 |
| TCGA-K4-A3WV | C3 | TCGA-ZF-A9R0 | >65 | MALE | G2 | Stage III | T3 | M0 | NX |
| TCGA-GU-AATQ | C3 | TCGA-C4-A0F6 | >65 | FEMALE | G2 | Stage III | T3 | M0 | N0 |
| TCGA-G2-A3VY | C3 | TCGA-FD-A5BT | >65 | MALE | G2 | Stage III | T3 | MX | N0 |
| TCGA-XF-AAN8 | C2 | TCGA-CF-A9FL | >65 | MALE | G2 | Stage III | T3 | M0 | N0 |
| TCGA-CU-A3QU | C3 | TCGA-XF-AAMX | >65 | FEMALE | G2 | Stage III | T3 | MX | N0 |
| TCGA-KQ-A41Q | C3 | TCGA-XF-A9T6 | >65 | FEMALE | G2 | Stage III | T3 | MX | N0 |
| TCGA-DK-A1AF | C2 | TCGA-KQ-A41Q | >65 | MALE | G2 | Stage III | T3 | MX | N0 |
| TCGA-LT-A5Z6 | C1 | TCGA-ZF-A9R9 | <=65 | MALE | G2 | Stage IV | T3 | MX | N2 |
| TCGA-GC-A4ZW | C3 | TCGA-FD-A6TA | <=65 | MALE | G2 | Stage IV | T3 | MX | N2 |
| TCGA-K4-A4AB | C2 | TCGA-ZF-AA5H | <=65 | FEMALE | G2 | Stage IV | T3 | M0 | N2 |
| TCGA-DK-AA74 | C2 | TCGA-XF-A9SY | <=65 | FEMALE | G2 | Stage IV | T3 | MX | N2 |
| TCGA-G2-A2EO | C2 | TCGA-XF-A8HH | <=65 | FEMALE | G2 | Stage IV | T3 | M0 | N2 |
| TCGA-DK-A1AG | C1 | TCGA-XF-A9SX | <=65 | FEMALE | G2 | Stage IV | T3 | MX | N2 |
| TCGA-GV-A3JZ | C3 | TCGA-BT-A20T | <=65 | MALE | G2 | Stage IV | T3 | M0 | N1 |
| TCGA-XF-A9SV | C2 | TCGA-K4-A6MB | <=65 | MALE | G2 | Stage IV | T3 | MX | N1 |
| TCGA-UY-A78K | C1 | TCGA-XF-A9SK | <=65 | MALE | G2 | Stage IV | T3 | MX | N1 |
| TCGA-FD-A6TH | C3 | TCGA-G2-AA3C | >65 | MALE | G2 | Stage IV | T3 | M0 | N1 |
| TCGA-DK-AA6W | C3 | TCGA-GV-A3JV | >65 | MALE | G2 | Stage IV | T3 | MX | N1 |
| TCGA-CF-A3MG | C3 | TCGA-DK-A1AD | >65 | MALE | G2 | Stage IV | T3 | M0 | N2 |
| TCGA-DK-A3X1 | C3 | TCGA-XF-A9T3 | >65 | FEMALE | G2 | Stage IV | T3 | MX | N2 |
| TCGA-DK-A1A6 | C1 | TCGA-BT-A20Q | >65 | MALE | G2 | Stage IV | T3 | M0 | N2 |
| TCGA-XF-AAMG | C3 | TCGA-XF-A9SU | >65 | FEMALE | G2 | Stage IV | T3 | MX | N1 |
| TCGA-E7-A541 | C1 | TCGA-XF-AAMT | >65 | FEMALE | G2 | Stage IV | T3 | MX | N2 |
| TCGA-XF-A8HE | C2 | TCGA-CU-A72E | >65 | MALE | G2 | Stage IV | T3 | M0 | N2 |
| TCGA-DK-AA6Q | C3 | TCGA-BT-A3PH | >65 | MALE | G2 | Stage IV | T3 | MX | N2 |
| TCGA-UY-A78N | C3 | TCGA-FT-A61P | >65 | MALE | G2 | Stage IV | T3 | MX | N2 |
| TCGA-DK-AA75 | C3 | TCGA-KQ-A41P | >65 | MALE | G2 | Stage IV | T3 | M1 | N3 |
| TCGA-ZF-AA5P | C2 | TCGA-XF-AAN3 | >65 | MALE | G2 | Stage IV | T3 | MX | N2 |
| TCGA-GC-A3WC | C1 | TCGA-HQ-A5ND | >65 | MALE | G2 | Stage IV | T3 | M0 | N1 |
| TCGA-K4-A4AC | C3 | TCGA-GV-A3QF | >65 | MALE | G2 | Stage IV | T3 | MX | N2 |
| TCGA-FD-A3SL | C2 | TCGA-BT-A20R | >65 | FEMALE | G2 | Stage IV | T3 | M0 | N1 |
| TCGA-K4-A5RH | C2 | TCGA-XF-A9SZ | >65 | MALE | G2 | Stage IV | T3 | MX | N2 |
| TCGA-FD-A5C1 | C2 | TCGA-XF-AAMH | >65 | MALE | G2 | Stage IV | T3 | MX | N1 |
| TCGA-XF-AAN7 | C2 | TCGA-FD-A6TF | >65 | FEMALE | G2 | Stage IV | T3 | M1 | N2 |
| TCGA-2F-A9KO | C1 | TCGA-GU-A767 | >65 | MALE | G2 | Stage IV | T3 | MX | N2 |
| TCGA-DK-A3IM | C3 | TCGA-ZF-A9R1 | >65 | MALE | G2 | Stage IV | T3 | M0 | N1 |
| TCGA-FD-A3SS | C3 | TCGA-FD-A5BX | >65 | MALE | G2 | Stage IV | T3 | MX | N1 |
| TCGA-UY-A9PB | C2 | TCGA-XF-A9SJ | >65 | MALE | G2 | Stage IV | T3 | MX | N2 |
| TCGA-FD-A6TI | C2 | TCGA-XF-A9SW | >65 | MALE | G2 | Stage IV | T3 | MX | N2 |
| TCGA-XF-AAMJ | C2 | TCGA-FD-A43Y | <=65 | MALE | G2 | Stage III | T4 | MX | N0 |
| TCGA-GU-A763 | C1 | TCGA-GU-A763 | >65 | MALE | G2 | Stage III | T4 | M0 | N0 |
| TCGA-G2-A2EC | C2 | TCGA-5N-A9KI | >65 | FEMALE | G2 | Stage III | T4 | MX | NX |
| TCGA-FD-A3B7 | C2 | TCGA-E7-A7DV | <=65 | MALE | G2 | Stage IV | T4 | MX | N3 |
| TCGA-FD-A5C0 | C2 | TCGA-ZF-AA4T | <=65 | MALE | G2 | Stage IV | T4 | MX | N2 |
| TCGA-FD-A5BS | C1 | TCGA-BL-A13J | <=65 | MALE | G2 | Stage IV | T4 | M0 | N2 |
| TCGA-CU-A0YN | C2 | TCGA-FD-A3SS | >65 | MALE | G2 | Stage IV | T4 | MX | N3 |
| TCGA-FD-A5BZ | C2 | TCGA-XF-AAMF | >65 | MALE | G2 | Stage IV | T4 | MX | N2 |
| TCGA-UY-A9PE | C3 | TCGA-DK-AA6R | >65 | MALE | G2 | Stage IV | T4 | M0 | N1 |
| TCGA-GV-A40E | C3 | TCGA-SY-A9G0 | >65 | MALE | G2 | Stage IV | T4 | M0 | N1 |
| TCGA-BT-A2LD | C3 | TCGA-UY-A8OC | >65 | MALE | G2 | Stage IV | T4 | MX | N1 |
| TCGA-UY-A9PD | C3 | TCGA-XF-AAMG | <=65 | MALE | G2 | Stage III | T4 | MX | N0 |
| TCGA-KQ-A41N | C3 | TCGA-H4-A2HO | <=65 | MALE | G2 | Stage III | T4 | MX | N0 |
| TCGA-GD-A3OQ | C2 | TCGA-FD-A3B4 | <=65 | FEMALE | G2 | Stage III | T4 | MX | N0 |
| TCGA-BT-A20U | C2 | TCGA-4Z-AA7S | >65 | MALE | G2 | Stage III | T4 | M0 | N0 |
| TCGA-XF-A9SW | C2 | TCGA-SY-A9G5 | >65 | MALE | G2 | Stage III | T4 | M0 | N0 |
| TCGA-FD-A3B3 | C2 | TCGA-CU-A3YL | >65 | MALE | G2 | Stage III | T4 | M0 | N0 |
| TCGA-ZF-A9RC | C3 | TCGA-CU-A5W6 | >65 | MALE | G2 | Stage III | T4 | M0 | N0 |
| TCGA-E7-A6MD | C1 | TCGA-ZF-AA4U | >65 | MALE | G2 | Stage III | T4 | MX | NX |
| TCGA-E5-A4TZ | C3 | TCGA-4Z-AA87 | >65 | MALE | G2 | Stage III | T4 | M0 | N0 |
| TCGA-ZF-A9R4 | C1 | TCGA-DK-A3IN | >65 | MALE | G2 | Stage III | T4 | M0 | N0 |
| TCGA-ZF-A9RE | C3 | TCGA-5N-A9KM | >65 | FEMALE | G2 | Stage III | T4 | MX | N0 |
| TCGA-BT-A20W | C1 | TCGA-XF-AAN1 | >65 | FEMALE | G2 | Stage III | T4 | M0 | N0 |
| TCGA-GC-A6I3 | C1 | TCGA-BT-A0S7 | >65 | MALE | G2 | Stage III | T4 | MX | N0 |
| TCGA-4Z-AA80 | C3 | TCGA-K4-A4AB | >65 | MALE | G2 | Stage III | T4 | MX | N0 |
| TCGA-G2-A3IE | C1 | TCGA-ZF-AA56 | >65 | FEMALE | G2 | Stage III | T4 | MX | N0 |
| TCGA-ZF-A9R0 | C1 | TCGA-FD-A6TC | >65 | FEMALE | G2 | Stage III | T4 | MX | N0 |
| TCGA-FD-A43S | C1 | TCGA-BL-A5ZZ | >65 | FEMALE | G2 | Stage III | T4 | MX | N0 |
| TCGA-XF-A8HG | C1 | TCGA-FT-A3EE | >65 | FEMALE | G2 | Stage III | T4 | MX | N0 |
| TCGA-K4-A83P | C1 | TCGA-K4-A3WU | >65 | MALE | G2 | Stage III | T4 | MX | N0 |
| TCGA-GD-A2C5 | C3 | TCGA-KQ-A41S | >65 | FEMALE | G2 | Stage III | T4 | MX | NX |
| TCGA-XF-AAMX | C3 | TCGA-K4-A83P | >65 | MALE | unknow | Stage IV | T4 | MX | N1 |
| TCGA-DK-A1AE | C3 | TCGA-DK-A6B5 | <=65 | MALE | G2 | Stage IV | T4 | M0 | N2 |
| TCGA-FJ-A3ZE | C3 | TCGA-GD-A3OQ | <=65 | MALE | G2 | Stage IV | T4 | MX | N1 |
| TCGA-DK-A2I1 | C2 | TCGA-GV-A3JZ | <=65 | MALE | G2 | Stage IV | T4 | MX | N3 |
| TCGA-DK-A6B0 | C1 | TCGA-GV-A3QK | <=65 | FEMALE | G2 | Stage IV | T4 | M0 | N2 |
| TCGA-FD-A5BR | C2 | TCGA-BT-A20X | <=65 | MALE | G2 | Stage IV | T4 | M0 | N2 |
| TCGA-E7-A7PW | C1 | TCGA-BT-A20V | <=65 | FEMALE | G2 | Stage IV | T4 | M0 | N2 |
| TCGA-LT-A8JT | C1 | TCGA-FD-A3SL | <=65 | MALE | G2 | Stage IV | T4 | M1 | N2 |
| TCGA-FJ-A3Z9 | C3 | TCGA-E7-A97Q | <=65 | FEMALE | G2 | Stage IV | T4 | MX | N3 |
| TCGA-DK-A1AC | C1 | TCGA-BT-A42F | <=65 | MALE | G2 | Stage IV | T4 | MX | N1 |
| TCGA-C4-A0F1 | C2 | TCGA-E7-A6MD | >65 | MALE | G2 | Stage IV | T4 | M0 | N1 |
| TCGA-G2-A2ES | C2 | TCGA-FD-A3SR | >65 | MALE | G2 | Stage IV | T4 | MX | N2 |
| TCGA-XF-AAN4 | C2 | TCGA-GU-A42R | >65 | MALE | G2 | Stage IV | T4 | MX | N2 |
| TCGA-4Z-AA83 | C1 | TCGA-XF-AAN0 | >65 | MALE | G2 | Stage IV | T4 | M0 | N2 |
| TCGA-BT-A20V | C3 | TCGA-FD-A43U | >65 | MALE | G2 | Stage IV | T4 | MX | N2 |
| TCGA-XF-A8HD | C1 | TCGA-DK-A1AB | >65 | FEMALE | G2 | Stage IV | T4 | M0 | N2 |
| TCGA-GV-A3JX | C3 | TCGA-GU-AATO | >65 | MALE | G2 | Stage IV | T4 | MX | N2 |
| TCGA-H4-A2HQ | C3 | TCGA-FJ-A3Z7 | >65 | MALE | G2 | Stage IV | T4 | MX | N2 |
| TCGA-XF-A9SP | C2 | TCGA-LC-A66R | >65 | MALE | G2 | Stage IV | T4 | MX | N2 |
| TCGA-DK-A3IK | C1 | TCGA-XF-A9SV | >65 | MALE | G2 | Stage IV | T4 | M1 | N2 |
| TCGA-4Z-AA7N | C1 | TCGA-S5-A6DX | >65 | MALE | G2 | Stage IV | T4 | MX | N2 |
| TCGA-HQ-A2OE | C3 | TCGA-GD-A3OP | >65 | FEMALE | G2 | Stage IV | T4 | MX | N2 |
| TCGA-UY-A8OC | C2 | TCGA-GU-A762 | >65 | MALE | G2 | Stage IV | T4 | MX | N2 |
| TCGA-GV-A3JV | C1 | TCGA-4Z-AA89 | <=65 | MALE | G2 | Stage IV | T4 | M0 | N0 |
| TCGA-XF-A9T8 | C2 | TCGA-E5-A4TZ | <=65 | MALE | G2 | Stage IV | T4 | MX | N2 |
| TCGA-KQ-A41S | C2 | TCGA-4Z-AA7R | >65 | MALE | G2 | Stage IV | T4 | M0 | N0 |
| TCGA-CU-A3YL | C1 | TCGA-FD-A6TI | >65 | MALE | G2 | Stage IV | T4 | MX | N1 |
| TCGA-E7-A7XN | C1 | TCGA-C4-A0F7 | >65 | MALE | G2 | Stage IV | T4 | M0 | N2 |
| TCGA-DK-A2HX | C2 | TCGA-FJ-A3Z9 | >65 | MALE | G2 | unknow | T4 | M0 | N0 |
| TCGA-CF-A8HY | C3 | TCGA-DK-AA6U | <=65 | MALE | unknow | Stage II | unknow | M0 | N0 |
| TCGA-4Z-AA7W | C1 | TCGA-E7-A3Y1 | <=65 | MALE | G1 | Stage II | unknow | M0 | N0 |
| TCGA-FD-A43U | C1 | TCGA-G2-A2EF | <=65 | MALE | G2 | Stage II | unknow | M0 | N0 |
| TCGA-BT-A0S7 | C2 | TCGA-G2-A3IE | <=65 | MALE | G2 | Stage II | unknow | MX | NX |
| TCGA-ZF-A9R3 | C1 | TCGA-GD-A3OS | <=65 | FEMALE | G2 | Stage II | unknow | MX | NX |
| TCGA-4Z-AA89 | C1 | TCGA-LT-A5Z6 | <=65 | MALE | G2 | Stage II | unknow | MX | NX |
| TCGA-GU-A42P | C3 | TCGA-G2-A2EJ | <=65 | FEMALE | G2 | Stage II | unknow | M0 | N0 |
| TCGA-FD-A3NA | C2 | TCGA-DK-A6B6 | <=65 | MALE | G2 | Stage II | unknow | MX | NX |
| TCGA-BT-A20N | C3 | TCGA-G2-A2EK | <=65 | MALE | G2 | Stage II | unknow | M0 | N0 |
| TCGA-XF-A8HC | C3 | TCGA-G2-A2EC | <=65 | FEMALE | G2 | Stage II | unknow | M0 | N0 |
| TCGA-ZF-AA4W | C3 | TCGA-DK-A3IV | <=65 | MALE | G2 | Stage II | unknow | M0 | NX |
| TCGA-LC-A66R | C2 | TCGA-ZF-A9RL | <=65 | MALE | G2 | Stage II | unknow | MX | unknow |
| TCGA-E7-A8O7 | C1 | TCGA-DK-AA6T | <=65 | MALE | G2 | Stage II | unknow | M0 | N0 |
| TCGA-CF-A9FL | C2 | TCGA-DK-AA6X | <=65 | MALE | G2 | Stage II | unknow | M0 | N0 |
| TCGA-ZF-A9RD | C2 | TCGA-BT-A42C | <=65 | MALE | G2 | Stage II | unknow | M0 | N0 |
| TCGA-G2-AA3D | C1 | TCGA-DK-AA76 | <=65 | FEMALE | G2 | Stage II | unknow | M0 | N0 |
| TCGA-DK-A2I2 | C2 | TCGA-G2-A3IB | >65 | MALE | G2 | Stage II | unknow | MX | NX |
| TCGA-FD-A62S | C2 | TCGA-G2-A3VY | >65 | MALE | G2 | Stage II | unknow | unknow | unknow |
| TCGA-MV-A51V | C3 | TCGA-GV-A3QH | >65 | MALE | G2 | Stage II | unknow | MX | NX |
| TCGA-E5-A2PC | C3 | TCGA-DK-AA6P | >65 | FEMALE | G2 | Stage II | unknow | M0 | N0 |
| TCGA-DK-A3X2 | C3 | TCGA-ZF-AA4N | >65 | MALE | G2 | Stage II | unknow | unknow | unknow |
| TCGA-GD-A76B | C1 | TCGA-GV-A40E | >65 | MALE | G2 | Stage II | unknow | MX | NX |
| TCGA-GC-A6I1 | C1 | TCGA-ZF-A9R7 | >65 | FEMALE | G2 | Stage II | unknow | M0 | NX |
| TCGA-XF-A9SK | C2 | TCGA-G2-A2EL | >65 | MALE | G2 | Stage II | unknow | M0 | N0 |
| TCGA-DK-A1AD | C1 | TCGA-ZF-A9RE | >65 | FEMALE | G2 | Stage II | unknow | MX | unknow |
| TCGA-K4-A5RI | C3 | TCGA-YC-A89H | >65 | FEMALE | G2 | Stage II | unknow | MX | unknow |
| TCGA-FD-A3SQ | C2 | TCGA-FJ-A3ZF | >65 | MALE | G2 | Stage III | unknow | M0 | N0 |
| TCGA-GV-A3QF | C3 | TCGA-UY-A78K | <=65 | MALE | G2 | Stage IV | unknow | MX | N2 |
|  |  | TCGA-UY-A78L | <=65 | MALE | G2 | Stage IV | unknow | MX | N1 |
|  |  | TCGA-H4-A2HQ | <=65 | FEMALE | G2 | Stage IV | unknow | M1 | NX |
|  |  | TCGA-FJ-A3ZE | <=65 | MALE | G2 | Stage IV | unknow | M1 | N3 |
|  |  | TCGA-YC-A9TC | >65 | MALE | G2 | Stage IV | unknow | M1 | unknow |
