# Supplementary material for: NXPH4 Used as a New Prognostic and Immunotherapeutic Marker for Muscle-Invasive Bladder Cancer
Source: J Oncol. 2022 Oct 4;2022:4271409. doi: 10.1155/2022/4271409 (PMC9553512; doi:10.1155/2022/4271409)
Supplement: Supplementary Materials — Figure s1: article roadmap of the whole research. Figure s2: (A) GSVA results heatmap of invasive bladder cancer in TCGA database (normal =19, tumor =404); Wayne diagram of differential pathways between clusters. (B) Wayne diagram in TCGA clusters (n = 65). (C) Wayne diagram in GEO clusters (n = 463). (D) Wayne diagram in TCGA clusters and GEO clusters (n = 6). Figure s3: (A) the 28 prognostic key pathway genes (P <0.01). Risk model for patients with muscle invasive bladder cancer (MIBC) based on 12 genes (SLC7A2, MST1R, CDK6, NXPH4, GRIK2, TRIB3, PBK, ABCA4, FBN2, SCG2, ELN, and INCENP). (B) LASSO regression with 10-fold crossvalidation was used to obtain 12 prognostic genes with an error within one standard error of the minimum (lambda.1se). (C) LASSO coefficient profiles of 28 key pathway genes. Supplement Table 1: clinical characteristics such as N, M, T, tumor grade, and stage, including age among the three groups (TCGA). Supplement Table 2: survival and prognosis information of three groups based on GEO. Supplement Table 3: 65 differential pathways were obtained from the molecular subtypes of TCGA queue. Supplement Table 4: 6 common differential pathways were obtained based on 65 TCGA, differential pathways, and 463 GEO, differential pathways. Supplement Table 5: 6 common differential pathways with prognosis. Supplement Table 6: the risk model based on the 12 prognostic genes in TCGA and GEO databases. Supplement Table 7: immune landscape between the high- and low-risk patients with muscle invasive bladder cancer (MIBC). Supplement Table 8: evaluation of immune response to CTLA4 and PD1 immunosuppressants in MIBC patients. [file 4271409.f1.zip › supplement table4.docx]

**Supplement Table4** 6 common differential pathways were obtained based on 65 TCGA - differential pathways and 463 GEO - differential pathways

| GEO-different GSVA | TCGA-different GSVA | TCGA-GEO-6 common dif-GSVA | |
| --- | --- | --- | --- |
| id | HALLMARK_EPITHELIAL_MESENCHYMAL_TRANSITION | GSE21670_STAT3_KO_VS_WT_CD4_TCELL_TGFB_IL6_TREATED_DN | |
| GSE14415_INDUCED_TREG_VS_TCONV_UP | HALLMARK_HEDGEHOG_SIGNALING | GSE1460_INTRATHYMIC_T_PROGENITOR_VS_CD4_THYMOCYTE_DN | |
| GSE14415_INDUCED_VS_NATURAL_TREG_DN | GSE11057_PBMC_VS_MEM_CD4_TCELL_UP | GSE17974_IL4_AND_ANTI_IL12_VS_UNTREATED_72H_ACT_CD4_TCELL_DN | |
| GSE16451_CTRL_VS_WEST_EQUINE_ENC_VIRUS_IMMATURE_NEURON_CELL_LINE_DN | GSE29618_MONOCYTE_VS_PDC_UP | GSE39556_UNTREATED_VS_3H_POLYIC_INJ_MOUSE_CD8A_DC_UP | |
| GSE14415_TCONV_VS_FOXP3_KO_INDUCED_TREG_DN | GSE21670_UNTREATED_VS_IL6_TREATED_STAT3_KO_CD4_TCELL_UP | GSE42088_2H_VS_24H_LEISHMANIA_INF_DC_UP | |
| GSE45365_WT_VS_IFNAR_KO_BCELL_MCMV_INFECTION_DN | GSE21670_STAT3_KO_VS_WT_CD4_TCELL_TGFB_IL6_TREATED_DN | GSE29618_BCELL_VS_PDC_UP | |
| GSE45365_HEALTHY_VS_MCMV_INFECTION_CD11B_DC_DN | GSE2405_S_AUREUS_VS_A_PHAGOCYTOPHILUM_NEUTROPHIL_UP | | |
| GSE39110_DAY3_VS_DAY6_POST_IMMUNIZATION_CD8_TCELL_DN | GSE40274_CTRL_VS_FOXP3_AND_LEF1_TRANSDUCED_ACTIVATED_CD4_TCELL_UP | | |
| GSE13547_CTRL_VS_ANTI_IGM_STIM_BCELL_2H_UP | GSE24634_TEFF_VS_TCONV_DAY3_IN_CULTURE_DN | |  |
| GSE13547_CTRL_VS_ANTI_IGM_STIM_BCELL_12H_UP | GSE24634_TREG_VS_TCONV_POST_DAY5_IL4_CONVERSION_DN | | |
| GSE3982_BCELL_VS_TH1_DN | GSE29618_PDC_VS_MDC_DAY7_FLU_VACCINE_DN | |  |
| GSE24671_BAKIMULC_VS_SENDAI_VIRUS_INFECTED_MOUSE_SPLENOCYTES_UP | GSE24671_CTRL_VS_SENDAI_VIRUS_INFECTED_MOUSE_SPLENOCYTES_UP | | |
| GSE28726_NAIVE_VS_ACTIVATED_CD4_TCELL_DN | GSE40274_CTRL_VS_FOXP3_AND_GATA1_TRANSDUCED_ACTIVATED_CD4_TCELL_UP | | |
| GSE3982_BCELL_VS_TH2_DN | GSE3565_CTRL_VS_LPS_INJECTED_SPLENOCYTES_UP | |  |
| GSE3982_CENT_MEMORY_CD4_TCELL_VS_TH1_DN | GSE3565_CTRL_VS_LPS_INJECTED_DUSP1_KO_SPLENOCYTES_UP | | |
| GSE40666_WT_VS_STAT1_KO_CD8_TCELL_WITH_IFNA_STIM_90MIN_DN | GSE30083_SP2_VS_SP4_THYMOCYTE_DN |  |  |
| GSE24634_IL4_VS_CTRL_TREATED_NAIVE_CD4_TCELL_DAY7_UP | GSE40274_CTRL_VS_XBP1_TRANSDUCED_ACTIVATED_CD4_TCELL_UP | | |
| GSE32164_RESTING_DIFFERENTIATED_VS_ALTERNATIVELY_ACT_M2_MACROPHAGE_UP | GSE22886_DC_VS_MONOCYTE_DN |  |  |
| GSE20366_EX_VIVO_VS_HOMEOSTATIC_CONVERSION_TREG_DN | GSE26495_NAIVE_VS_PD1LOW_CD8_TCELL_DN | |  |
| GSE28726_NAIVE_CD4_TCELL_VS_NAIVE_VA24NEG_NKTCELL_UP | GSE45739_UNSTIM_VS_ACD3_ACD28_STIM_NRAS_KO_CD4_TCELL_DN | | |
| GSE23568_CTRL_VS_ID3_TRANSDUCED_CD8_TCELL_DN | GOLDRATH_NAIVE_VS_MEMORY_CD8_TCELL_DN | |  |
| GSE2770_UNTREATED_VS_IL4_TREATED_ACT_CD4_TCELL_2H_UP | GSE30083_SP3_VS_SP4_THYMOCYTE_DN |  |  |
| GOLDRATH_EFF_VS_MEMORY_CD8_TCELL_UP | GSE26495_NAIVE_VS_PD1HIGH_CD8_TCELL_DN | |  |
| GSE12845_IGD_POS_BLOOD_VS_PRE_GC_TONSIL_BCELL_DN | GSE2935_UV_INACTIVATED_VS_LIVE_SENDAI_VIRUS_INF_MACROPHAGE_DN | | |
| GSE15750_DAY6_VS_DAY10_TRAF6KO_EFF_CD8_TCELL_UP | GSE29618_PDC_VS_MDC_DN |  |  |
| GSE13547_2H_VS_12_H_ANTI_IGM_STIM_ZFX_KO_BCELL_DN | GSE1460_INTRATHYMIC_T_PROGENITOR_VS_CD4_THYMOCYTE_DN | | |
| GSE24634_TREG_VS_TCONV_POST_DAY7_IL4_CONVERSION_UP | GSE17974_IL4_AND_ANTI_IL12_VS_UNTREATED_48H_ACT_CD4_TCELL_DN | | |
| GSE30962_ACUTE_VS_CHRONIC_LCMV_PRIMARY_INF_CD8_TCELL_DN | GSE17974_IL4_AND_ANTI_IL12_VS_UNTREATED_72H_ACT_CD4_TCELL_DN | | |
| GSE26156_DOUBLE_POSITIVE_VS_CD4_SINGLE_POSITIVE_THYMOCYTE_DN | GSE39556_UNTREATED_VS_3H_POLYIC_INJ_MOUSE_CD8A_DC_UP | | |
| GSE24634_TEFF_VS_TCONV_DAY10_IN_CULTURE_UP | GSE7219_UNSTIM_VS_LPS_AND_ANTI_CD40_STIM_NIK_NFKB2_KO_DC_UP | | |
| GSE32986_UNSTIM_VS_GMCSF_AND_CURDLAN_HIGHDOSE_STIM_DC_UP | GSE10325_CD4_TCELL_VS_BCELL_UP |  |  |
| GSE13547_2H_VS_12_H_ANTI_IGM_STIM_BCELL_DN | GSE12845_PRE_GC_VS_DARKZONE_GC_TONSIL_BCELL_UP | |  |
| GSE43863_TH1_VS_LY6C_LOW_CXCR5NEG_EFFECTOR_CD4_TCELL_UP | GSE13522_CTRL_VS_T_CRUZI_Y_STRAIN_INF_SKIN_IFNAR_KO_UP | | |
| GSE14415_NATURAL_TREG_VS_TCONV_DN | GSE2405_0H_VS_12H_A_PHAGOCYTOPHILUM_STIM_NEUTROPHIL_UP | | |
| GSE2405_S_AUREUS_VS_UNTREATED_NEUTROPHIL_DN | GSE23505_UNTREATED_VS_4DAY_IL6_IL1_IL23_TREATED_CD4_TCELL_DN | | |
| GOLDRATH_NAIVE_VS_EFF_CD8_TCELL_DN | GSE23568_ID3_KO_VS_WT_CD8_TCELL_DN |  |  |
| GSE25088_WT_VS_STAT6_KO_MACROPHAGE_DN | GSE23568_ID3_TRANSDUCED_VS_ID3_KO_CD8_TCELL_DN | |  |
| GSE12392_CD8A_POS_VS_NEG_SPLEEN_DC_DN | GSE42088_2H_VS_24H_LEISHMANIA_INF_DC_UP | |  |
| GSE23568_ID3_KO_VS_WT_CD8_TCELL_UP | GSE21670_STAT3_KO_VS_WT_CD4_TCELL_UP |  |  |
| GSE23502_WT_VS_HDC_KO_MYELOID_DERIVED_SUPPRESSOR_CELL_COLON_TUMOR_UP | GSE10325_LUPUS_CD4_TCELL_VS_LUPUS_BCELL_UP | |  |
| GSE10239_NAIVE_VS_DAY4.5_EFF_CD8_TCELL_DN | GSE32164_ALTERNATIVELY_ACT_M2_VS_CMYC_INHIBITED_MACROPHAGE_UP | | |
| GSE11386_NAIVE_VS_MEMORY_BCELL_UP | GSE41867_MEMORY_VS_EXHAUSTED_CD8_TCELL_DAY30_LCMV_UP | | |
| GSE13547_CTRL_VS_ANTI_IGM_STIM_ZFX_KO_BCELL_2H_UP | GSE33292_DN3_THYMOCYTE_VS_TCF1_KO_TCELL_LYMPHOMA_UP | | |
| GSE10273_HIGH_IL7_VS_HIGH_IL7_AND_IRF4_IN_IRF4_8_NULL_PRE_BCELL_UP | GSE3982_MEMORY_CD4_TCELL_VS_BCELL_UP |  |  |
| GSE15750_DAY6_VS_DAY10_EFF_CD8_TCELL_UP | GSE3039_ALPHAALPHA_VS_ALPHABETA_CD8_TCELL_DN | |  |
| GSE40274_CTRL_VS_EOS_TRANSDUCED_ACTIVATED_CD4_TCELL_UP | GSE45739_UNSTIM_VS_ACD3_ACD28_STIM_WT_CD4_TCELL_DN | | |
| GSE13411_PLASMA_CELL_VS_MEMORY_BCELL_UP | GSE43863_DAY6_EFF_VS_DAY150_MEM_LY6C_INT_CXCR5POS_CD4_TCELL_DN | | |
| GSE21670_UNTREATED_VS_TGFB_IL6_TREATED_STAT3_KO_CD4_TCELL_UP | GSE22886_DAY0_VS_DAY1_MONOCYTE_IN_CULTURE_UP | |  |
| GSE17301_IFNA2_VS_IFNA5_STIM_ACD3_ACD28_ACT_CD8_TCELL_DN | GSE3982_BCELL_VS_CENT_MEMORY_CD4_TCELL_UP | |  |
| GSE36826_WT_VS_IL1R_KO_SKIN_STAPH_AUREUS_INF_UP | GSE29618_BCELL_VS_PDC_UP |  |  |
| GSE9239_CTRL_VS_TNF_INHIBITOR_TREATED_DC_DN | GSE33425_CD8_ALPHAALPHA_VS_ALPHABETA_CD161_HIGH_TCELL_UP | | |
| GSE39110_DAY3_VS_DAY6_POST_IMMUNIZATION_CD8_TCELL_WITH_IL2_TREATMENT_UP | GSE10325_CD4_TCELL_VS_BCELL_DN |  |  |
| GSE30962_ACUTE_VS_CHRONIC_LCMV_SECONDARY_INF_CD8_TCELL_DN | GSE10325_LUPUS_CD4_TCELL_VS_LUPUS_BCELL_DN | |  |
| GSE10239_NAIVE_VS_KLRG1INT_EFF_CD8_TCELL_DN | GSE3982_MEMORY_CD4_TCELL_VS_BCELL_DN |  |  |
| GSE36476_CTRL_VS_TSST_ACT_72H_MEMORY_CD4_TCELL_YOUNG_DN | GSE29618_BCELL_VS_PDC_DAY7_FLU_VACCINE_UP | |  |
| GSE43863_TH1_VS_LY6C_INT_CXCR5POS_EFFECTOR_CD4_TCELL_UP | GSE29618_BCELL_VS_MDC_UP |  |  |
| GSE22229_RENAL_TRANSPLANT_VS_HEALTHY_PBMC_UP | GSE7509_UNSTIM_VS_FCGRIIB_STIM_DC_DN |  |  |
| GSE9509_LPS_VS_LPS_AND_IL10_STIM_IL10_KO_MACROPHAGE_20MIN_DN | GSE29618_BCELL_VS_MDC_DAY7_FLU_VACCINE_UP | |  |
| GSE10239_NAIVE_VS_KLRG1HIGH_EFF_CD8_TCELL_DN | GSE4748_CYANOBACTERIUM_LPSLIKE_VS_LPS_AND_CYANOBACTERIUM_LPSLIKE_STIM_DC_3H_UP | | |
| GSE17301_ACD3_ACD28_VS_ACD3_ACD28_AND_IFNA5_STIM_CD8_TCELL_UP | GSE22886_NAIVE_BCELL_VS_MONOCYTE_UP |  |  |
| GSE39110_UNTREATED_VS_IL2_TREATED_CD8_TCELL_DAY3_POST_IMMUNIZATION_DN | GSE16266_LPS_VS_HEATSHOCK_AND_LPS_STIM_MEF_UP | |  |
| GSE28408_LY6G_POS_VS_NEG_DC_UP | GSE22886_NAIVE_CD8_TCELL_VS_MONOCYTE_UP | |  |
| GSE36476_CTRL_VS_TSST_ACT_40H_MEMORY_CD4_TCELL_OLD_DN | GSE29618_BCELL_VS_MONOCYTE_UP |  |  |
| GSE35543_IN_VITRO_ITREG_VS_CONVERTED_EX_ITREG_UP | GSE10325_BCELL_VS_MYELOID_UP |  |  |
| GSE33292_WT_VS_TCF1_KO_DN3_THYMOCYTE_DN | GSE4984_UNTREATED_VS_GALECTIN1_TREATED_DC_DN | |  |
| GSE45365_HEALTHY_VS_MCMV_INFECTION_CD11B_DC_IFNAR_KO_DN | |  |  |
| GSE3039_CD4_TCELL_VS_ALPHAALPHA_CD8_TCELL_DN | |  |  |
| GSE36476_CTRL_VS_TSST_ACT_40H_MEMORY_CD4_TCELL_YOUNG_DN | |  |  |
| GSE3982_DC_VS_TH1_DN |  |  |  |
| GSE19941_LPS_VS_LPS_AND_IL10_STIM_IL10_KO_MACROPHAGE_UP | |  |  |
| GSE30962_PRIMARY_VS_SECONDARY_ACUTE_LCMV_INF_CD8_TCELL_UP | |  |  |
| GSE13547_2H_VS_12_H_ANTI_IGM_STIM_BCELL_UP | |  |  |
| GSE3982_MEMORY_CD4_TCELL_VS_TH1_DN | |  |  |
| GSE5679_CTRL_VS_RARA_AGONIST_AM580_TREATED_DC_UP | |  |  |
| GSE45365_WT_VS_IFNAR_KO_CD11B_DC_MCMV_INFECTION_DN | |  |  |
| GSE21063_WT_VS_NFATC1_KO_8H_ANTI_IGM_STIM_BCELL_UP | |  |  |
| GSE40068_BCL6_POS_VS_NEG_CXCR5_POS_TFH_UP | |  |  |
| GSE20727_DNFB_ALLERGEN_VS_ROS_INH_AND_DNFB_ALLERGEN_TREATED_DC_DN | | |  |
| GSE25088_WT_VS_STAT6_KO_MACROPHAGE_IL4_STIM_DN | |  |  |
| GSE19941_UNSTIM_VS_LPS_AND_IL10_STIM_IL10_KO_MACROPHAGE_DN | |  |  |
| GSE2405_HEAT_KILLED_LYSATE_VS_LIVE_A_PHAGOCYTOPHILUM_STIM_NEUTROPHIL_9H_UP | | |  |
| GSE29614_CTRL_VS_TIV_FLU_VACCINE_PBMC_2007_DN | |  |  |
| GSE12963_ENV_NEF_VS_ENV_NEF_AND_VPR_DEFICIENT_HIV1_INF_CD4_TCELL_DN | | |  |
| GSE33162_UNTREATED_VS_4H_LPS_STIM_HDAC3_KO_MACROPHAGE_UP | |  |  |
| GSE32164_ALTERNATIVELY_ACT_M2_VS_CMYC_INHIBITED_MACROPHAGE_DN | |  |  |
| GSE3982_NKCELL_VS_TH1_DN |  |  |  |
| GSE23505_IL6_IL1_VS_IL6_IL1_IL23_TREATED_CD4_TCELL_UP | |  |  |
| GSE11961_UNSTIM_VS_ANTI_IGM_AND_CD40_STIM_6H_FOLLICULAR_BCELL_UP | |  |  |
| GSE5589_IL6_KO_VS_IL10_KO_LPS_AND_IL6_STIM_MACROPHAGE_45MIN_UP | |  |  |
| GSE5679_CTRL_VS_PPARG_LIGAND_ROSIGLITAZONE_TREATED_DC_UP | |  |  |
| GSE39556_UNTREATED_VS_3H_POLYIC_INJ_MOUSE_NK_CELL_UP | |  |  |
| GSE40274_CTRL_VS_FOXP3_TRANSDUCED_ACTIVATED_CD4_TCELL_UP | |  |  |
| GSE22886_UNSTIM_VS_IL2_STIM_NKCELL_DN | |  |  |
| GSE37532_WT_VS_PPARG_KO_VISCERAL_ADIPOSE_TISSUE_TREG_UP | |  |  |
| GSE12366_GC_VS_MEMORY_BCELL_UP | |  |  |
| GSE15930_NAIVE_VS_72H_IN_VITRO_STIM_TRICHOSTATINA_CD8_TCELL_DN | |  |  |
| GSE29614_CTRL_VS_DAY7_TIV_FLU_VACCINE_PBMC_DN | |  |  |
| GSE3720_UNSTIM_VS_PMA_STIM_VD2_GAMMADELTA_TCELL_DN | |  |  |
| GSE24634_TEFF_VS_TCONV_DAY7_IN_CULTURE_UP | |  |  |
| GSE3982_MAST_CELL_VS_TH1_DN | |  |  |
| GSE24634_TREG_VS_TCONV_POST_DAY3_IL4_CONVERSION_UP | |  |  |
| GSE36476_CTRL_VS_TSST_ACT_72H_MEMORY_CD4_TCELL_OLD_DN | |  |  |
| GSE7568_IL4_VS_IL4_AND_DEXAMETHASONE_TREATED_MACROPHAGE_UP | |  |  |
| GSE2128_C57BL6_VS_NOD_THYMOCYTE_UP | |  |  |
| GSE39556_CD8A_DC_VS_NK_CELL_MOUSE_3H_POST_POLYIC_INJ_UP | |  |  |
| GSE21927_SPLEEN_C57BL6_VS_EL4_TUMOR_BALBC_MONOCYTES_UP | |  |  |
| GSE24574_BCL6_HIGH_VS_LOW_TFH_CD4_TCELL_DN | |  |  |
| GSE26030_TH1_VS_TH17_RESTIMULATED_DAY15_POST_POLARIZATION_UP | |  |  |
| GSE24634_TEFF_VS_TCONV_DAY3_IN_CULTURE_UP | |  |  |
| GSE5589_WT_VS_IL10_KO_LPS_STIM_MACROPHAGE_45MIN_UP | |  |  |
| GSE15930_NAIVE_VS_72H_IN_VITRO_STIM_IFNAB_CD8_TCELL_DN | |  |  |
| HALLMARK_E2F_TARGETS |  |  |  |
| GSE3720_VD1_VS_VD2_GAMMADELTA_TCELL_UP | |  |  |
| GSE21927_SPLENIC_VS_TUMOR_MONOCYTES_FROM_C26GM_TUMOROUS_MICE_BALBC_DN | | |  |
| GSE32986_UNSTIM_VS_CURDLAN_HIGHDOSE_STIM_DC_UP | |  |  |
| GSE22313_HEALTHY_VS_SLE_MOUSE_CD4_TCELL_DN | |  |  |
| GSE2770_IL12_VS_IL4_TREATED_ACT_CD4_TCELL_48H_UP | |  |  |
| GSE5589_WT_VS_IL6_KO_LPS_AND_IL10_STIM_MACROPHAGE_45MIN_UP | |  |  |
| GSE28408_LY6G_POS_VS_NEG_DC_DN | |  |  |
| GSE3982_EFF_MEMORY_CD4_TCELL_VS_TH1_DN | |  |  |
| GSE36888_STAT5_AB_KNOCKIN_VS_WT_TCELL_IL2_TREATED_6H_DN | |  |  |
| GSE45365_WT_VS_IFNAR_KO_BCELL_DN | |  |  |
| GSE33425_CD8_ALPHAALPHA_VS_ALPHABETA_CD161_HIGH_TCELL_DN | |  |  |
| GSE14415_INDUCED_TREG_VS_FAILED_INDUCED_TREG_UP | |  |  |
| GSE22601_DOUBLE_POSITIVE_VS_CD8_SINGLE_POSITIVE_THYMOCYTE_DN | |  |  |
| GSE369_SOCS3_KO_VS_IFNG_KO_LIVER_DN | |  |  |
| GSE24634_NAIVE_CD4_TCELL_VS_DAY7_IL4_CONV_TREG_DN | |  |  |
| GSE22886_NAIVE_CD4_TCELL_VS_48H_ACT_TH1_UP | |  |  |
| GSE3982_CENT_MEMORY_CD4_TCELL_VS_TH2_DN | |  |  |
| GSE32901_NAIVE_VS_TH17_NEG_CD4_TCELL_UP | |  |  |
| GSE29164_CD8_TCELL_VS_CD8_TCELL_AND_IL12_TREATED_MELANOMA_DAY7_DN | | |  |
| GSE9650_EFFECTOR_VS_MEMORY_CD8_TCELL_UP | |  |  |
| GSE3982_MEMORY_CD4_TCELL_VS_TH2_DN | |  |  |
| GSE17186_NAIVE_VS_CD21LOW_TRANSITIONAL_BCELL_CORD_BLOOD_DN | |  |  |
| GSE26669_CTRL_VS_COSTIM_BLOCK_MLR_CD8_TCELL_UP | |  |  |
| GSE16450_IMMATURE_VS_MATURE_NEURON_CELL_LINE_DN | |  |  |
| HALLMARK_G2M_CHECKPOINT |  |  |  |
| GSE8921_UNSTIM_0H_VS_TLR1_2_STIM_MONOCYTE_12H_UP | |  |  |
| GSE17974_CTRL_VS_ACT_IL4_AND_ANTI_IL12_72H_CD4_TCELL_DN | |  |  |
| GSE17974_0H_VS_24H_IN_VITRO_ACT_CD4_TCELL_DN | |  |  |
| GSE37532_WT_VS_PPARG_KO_LN_TCONV_DN | |  |  |
| GSE12366_GC_VS_NAIVE_BCELL_UP | |  |  |
| GSE3982_DC_VS_TH2_DN |  |  |  |
| GSE3982_MAST_CELL_VS_TH2_DN | |  |  |
| GSE33424_CD161_HIGH_VS_NEG_CD8_TCELL_DN | |  |  |
| GSE21670_IL6_VS_TGFB_AND_IL6_TREATED_CD4_TCELL_DN | |  |  |
| GSE22443_IL2_VS_IL12_TREATED_ACT_CD8_TCELL_DN | |  |  |
| GSE21063_CTRL_VS_ANTI_IGM_STIM_BCELL_16H_UP | |  |  |
| GSE21063_3H_VS_16H_ANTI_IGM_STIM_NFATC1_KOBCELL_DN | |  |  |
| GSE18203_CTRL_VS_INTRATUMORAL_CPG_INJ_MC38_TUMOR_DN | |  |  |
| GSE20715_0H_VS_48H_OZONE_LUNG_DN | |  |  |
| GSE15930_NAIVE_VS_24H_IN_VITRO_STIM_INFAB_CD8_TCELL_DN | |  |  |
| GSE17812_WT_VS_THPOK_KO_MEMORY_CD8_TCELL_DN | |  |  |
| GSE15930_NAIVE_VS_24H_IN_VITRO_STIM_IL12_CD8_TCELL_DN | |  |  |
| KAECH_DAY8_EFF_VS_MEMORY_CD8_TCELL_UP | |  |  |
| GSE40274_FOXP3_VS_FOXP3_AND_EOS_TRANSDUCED_ACTIVATED_CD4_TCELL_DN | | |  |
| GSE6259_FLT3L_INDUCED_33D1_POS_DC_VS_CD4_TCELL_DN | |  |  |
| GSE17301_ACD3_ACD28_VS_ACD3_ACD28_AND_IFNA2_STIM_CD8_TCELL_UP | |  |  |
| GSE32986_UNSTIM_VS_GMCSF_AND_CURDLAN_LOWDOSE_STIM_DC_UP | |  |  |
| GSE8921_UNSTIM_VS_TLR1_2_STIM_MONOCYTE_3H_UP | |  |  |
| GSE41087_WT_VS_FOXP3_MUT_ANTI_CD3_CD28_STIM_CD4_TCELL_DN | |  |  |
| GSE33162_UNTREATED_VS_4H_LPS_STIM_HDAC3_KO_MACROPHAGE_DN | |  |  |
| GSE9878_CTRL_VS_EBF_TRANSDUCED_PAX5_KO_PRO_BCELL_DN | |  |  |
| GSE26351_WNT_VS_BMP_PATHWAY_STIM_HEMATOPOIETIC_PROGENITORS_UP | |  |  |
| GSE21360_PRIMARY_VS_QUATERNARY_MEMORY_CD8_TCELL_DN | |  |  |
| GSE3982_NKCELL_VS_TH2_DN |  |  |  |
| GSE24634_TEFF_VS_TCONV_DAY5_IN_CULTURE_UP | |  |  |
| GSE12845_NAIVE_VS_PRE_GC_TONSIL_BCELL_DN | |  |  |
| GSE25085_FETAL_LIVER_VS_ADULT_BM_SP4_THYMIC_IMPLANT_DN | |  |  |
| GSE5142_HTERT_TRANSDUCED_VS_CTRL_CD8_TCELL_LATE_PASSAGE_CLONE_UP | | |  |
| GSE17301_CTRL_VS_48H_ACD3_ACD28_IFNA2_STIM_CD8_TCELL_DN | |  |  |
| GSE22886_UNSTIM_VS_IL15_STIM_NKCELL_DN | |  |  |
| GSE27241_WT_VS_RORGT_KO_TH17_POLARIZED_CD4_TCELL_UP | |  |  |
| GSE1460_DP_THYMOCYTE_VS_NAIVE_CD4_TCELL_ADULT_BLOOD_UP | |  |  |
| GSE15330_HSC_VS_LYMPHOID_PRIMED_MULTIPOTENT_PROGENITOR_DN | |  |  |
| GSE22601_DOUBLE_POSITIVE_VS_CD4_SINGLE_POSITIVE_THYMOCYTE_DN | |  |  |
| GSE10240_CTRL_VS_IL17_AND_IL22_STIM_PRIMARY_BRONCHIAL_EPITHELIAL_CELLS_UP | | |  |
| GSE7852_LN_VS_THYMUS_TCONV_DN | |  |  |
| GSE15930_NAIVE_VS_24H_IN_VITRO_STIM_CD8_TCELL_DN | |  |  |
| GSE1460_INTRATHYMIC_T_PROGENITOR_VS_NAIVE_CD4_TCELL_ADULT_BLOOD_UP | | |  |
| KAECH_DAY8_EFF_VS_DAY15_EFF_CD8_TCELL_UP | |  |  |
| GSE17186_CD21LOW_VS_CD21HIGH_TRANSITIONAL_BCELL_UP | |  |  |
| GSE37563_WT_VS_CTLA4_KO_CD4_TCELL_D4_POST_IMMUNIZATION_UP | |  |  |
| GSE2770_IL12_AND_TGFB_VS_IL4_TREATED_ACT_CD4_TCELL_6H_UP | |  |  |
| GSE10273_LOW_IL7_VS_HIGH_IL7_AND_IRF4_IN_IRF4_8_NULL_PRE_BCELL_UP | |  |  |
| GSE25087_FETAL_VS_ADULT_TCONV_DN | |  |  |
| GSE27786_LIN_NEG_VS_NKTCELL_UP | |  |  |
| GSE3982_BASOPHIL_VS_TH1_DN |  |  |  |
| GSE13547_WT_VS_ZFX_KO_BCELL_ANTI_IGM_STIM_2H_UP | |  |  |
| GSE13547_WT_VS_ZFX_KO_BCELL_ANTI_IGM_STIM_12H_DN | |  |  |
| GSE10273_HIGH_IL7_VS_HIGH_IL7_AND_IRF4_IN_IRF4_8_NULL_PRE_BCELL_DN | |  |  |
| GSE34205_HEALTHY_VS_RSV_INF_INFANT_PBMC_DN | |  |  |
| GSE22432_CONVENTIONAL_CDC_VS_PLASMACYTOID_PDC_UP | |  |  |
| GSE24634_NAIVE_CD4_TCELL_VS_DAY3_IL4_CONV_TREG_DN | |  |  |
| GSE17974_CTRL_VS_ACT_IL4_AND_ANTI_IL12_24H_CD4_TCELL_DN | |  |  |
| GSE22103_UNSTIM_VS_GMCSF_AND_IFNG_STIM_NEUTROPHIL_DN | |  |  |
| GSE21927_GMCSF_IL6_VS_GMCSF_GCSF_TREATED_BONE_MARROW_DN | |  |  |
| GSE12845_IGD_NEG_BLOOD_VS_PRE_GC_TONSIL_BCELL_DN | |  |  |
| GSE6259_33D1_POS_DC_VS_CD4_TCELL_UP | |  |  |
| GSE3982_EFF_MEMORY_CD4_TCELL_VS_TH2_DN | |  |  |
| GSE5542_IFNA_VS_IFNA_AND_IFNG_TREATED_EPITHELIAL_CELLS_24H_DN | |  |  |
| GSE21927_C26GM_VS_4T1_TUMOR_MONOCYTE_BALBC_DN | |  |  |
| GSE29614_DAY3_VS_DAY7_TIV_FLU_VACCINE_PBMC_DN | |  |  |
| GSE2405_0H_VS_12H_A_PHAGOCYTOPHILUM_STIM_NEUTROPHIL_DN | |  |  |
| GSE24634_TREG_VS_TCONV_POST_DAY10_IL4_CONVERSION_UP | |  |  |
| GSE33425_CD161_HIGH_VS_INT_CD8_TCELL_DN | |  |  |
| GSE3982_EOSINOPHIL_VS_TH2_DN | |  |  |
| GSE3691_IFN_PRODUCING_KILLER_DC_VS_PLASMACYTOID_DC_SPLEEN_DN | |  |  |
| GSE6674_CPG_VS_CPG_AND_ANTI_IGM_STIM_BCELL_UP | |  |  |
| GSE40225_WT_VS_RIP_B7X_DIABETIC_MOUSE_PANCREATIC_CD8_TCELL_DN | |  |  |
| GSE411_WT_VS_SOCS3_KO_MACROPHAGE_IL6_STIM_100MIN_UP | |  |  |
| GSE20727_CTRL_VS_ROS_INH_AND_DNFB_ALLERGEN_TREATED_DC_DN | |  |  |
| GSE24574_BCL6_LOW_TFH_VS_TCONV_CD4_TCELL_DN | |  |  |
| GSE32986_UNSTIM_VS_GMCSF_AND_CURDLAN_HIGHDOSE_STIM_DC_DN | |  |  |
| GSE33424_CD161_HIGH_VS_INT_CD8_TCELL_DN | |  |  |
| GSE18893_TCONV_VS_TREG_24H_TNF_STIM_UP | |  |  |
| GSE12963_UNINF_VS_ENV_AND_NEF_DEFICIENT_HIV1_INF_CD4_TCELL_DN | |  |  |
| GSE3982_MAC_VS_TH1_DN |  |  |  |
| GSE15930_NAIVE_VS_72H_IN_VITRO_STIM_CD8_TCELL_DN | |  |  |
| GSE3982_EOSINOPHIL_VS_TH1_DN | |  |  |
| GSE13411_IGM_MEMORY_BCELL_VS_PLASMA_CELL_DN | |  |  |
| GSE12707_AT16L1_HYPOMORPH_VS_WT_THYMUS_UP | |  |  |
| GSE21670_UNTREATED_VS_TGFB_IL6_TREATED_CD4_TCELL_UP | |  |  |
| GSE32901_TH1_VS_TH17_NEG_CD4_TCELL_DN | |  |  |
| GSE4142_NAIVE_VS_GC_BCELL_DN | |  |  |
| KAECH_NAIVE_VS_DAY8_EFF_CD8_TCELL_DN | |  |  |
| GSE13411_NAIVE_BCELL_VS_PLASMA_CELL_DN | |  |  |
| GSE7460_CTRL_VS_TGFB_TREATED_ACT_TREG_UP | |  |  |
| GSE22432_MULTIPOTENT_PROGENITOR_VS_CDC_DN | |  |  |
| GSE7764_IL15_TREATED_VS_CTRL_NK_CELL_24H_UP | |  |  |
| GSE1460_INTRATHYMIC_T_PROGENITOR_VS_NAIVE_CD4_TCELL_CORD_BLOOD_UP | | |  |
| GSE9509_LPS_VS_LPS_AND_IL10_STIM_IL10_KO_MACROPHAGE_30MIN_DN | |  |  |
| GSE25146_UNSTIM_VS_HELIOBACTER_PYLORI_LPS_STIM_AGS_CELL_UP | |  |  |
| GSE21927_BALBC_VS_C57BL6_MONOCYTE_TUMOR_UP | |  |  |
| GSE22886_NAIVE_CD4_TCELL_VS_12H_ACT_TH2_UP | |  |  |
| GSE21063_CTRL_VS_ANTI_IGM_STIM_BCELL_NFATC1_KO_3H_DN | |  |  |
| GSE21670_IL6_VS_TGFB_AND_IL6_TREATED_STAT3_KO_CD4_TCELL_UP | |  |  |
| GSE22045_TREG_VS_TCONV_UP |  |  |  |
| GSE360_L_DONOVANI_VS_M_TUBERCULOSIS_DC_DN | |  |  |
| GSE2770_UNTREATED_VS_IL4_TREATED_ACT_CD4_TCELL_48H_DN | |  |  |
| GSE1448_CTRL_VS_ANTI_VALPHA2_DP_THYMOCYTE_UP | |  |  |
| GSE17974_0H_VS_72H_IN_VITRO_ACT_CD4_TCELL_DN | |  |  |
| GSE15330_MEGAKARYOCYTE_ERYTHROID_PROGENITOR_VS_PRO_BCELL_DN | |  |  |
| GSE19941_UNSTIM_VS_LPS_STIM_IL10_KO_NFKBP50_KO_MACROPHAGE_UP | |  |  |
| GSE22886_NAIVE_CD4_TCELL_VS_48H_ACT_TH1_DN | |  |  |
| GSE14415_ACT_VS_CTRL_NATURAL_TREG_UP | |  |  |
| GSE22886_UNSTIM_VS_IL15_STIM_NKCELL_UP | |  |  |
| GSE25085_FETAL_LIVER_VS_FETAL_BM_SP4_THYMIC_IMPLANT_UP | |  |  |
| GSE40274_CTRL_VS_FOXP3_AND_XBP1_TRANSDUCED_ACTIVATED_CD4_TCELL_UP | | |  |
| GSE24634_TREG_VS_TCONV_POST_DAY5_IL4_CONVERSION_UP | |  |  |
| GSE21063_CTRL_VS_ANTI_IGM_STIM_BCELL_NFATC1_KO_8H_DN | |  |  |
| GSE14386_UNTREATED_VS_IFNA_TREATED_ACT_PBMC_MS_PATIENT_DN | |  |  |
| GSE13484_12H_UNSTIM_VS_YF17D_VACCINE_STIM_PBMC_DN | |  |  |
| GSE22886_NAIVE_BCELL_VS_BLOOD_PLASMA_CELL_DN | |  |  |
| GSE14350_TREG_VS_TEFF_IN_IL2RB_KO_DN | |  |  |
| GSE10325_MYELOID_VS_LUPUS_MYELOID_DN | |  |  |
| GSE3920_UNTREATED_VS_IFNB_TREATED_ENDOTHELIAL_CELL_DN | |  |  |
| GSE43955_1H_VS_10H_ACT_CD4_TCELL_WITH_TGFB_IL6_DN | |  |  |
| GSE24574_BCL6_HIGH_TFH_VS_TCONV_CD4_TCELL_DN | |  |  |
| GSE21670_STAT3_KO_VS_WT_CD4_TCELL_TGFB_TREATED_DN | |  |  |
| HALLMARK_SPERMATOGENESIS |  |  |  |
| GSE17974_CTRL_VS_ACT_IL4_AND_ANTI_IL12_6H_CD4_TCELL_DN | |  |  |
| GSE6259_FLT3L_INDUCED_DEC205_POS_DC_VS_BCELL_DN | |  |  |
| GSE360_DC_VS_MAC_M_TUBERCULOSIS_UP | |  |  |
| GSE8685_IL2_STARVED_VS_IL2_ACT_IL2_STARVED_CD4_TCELL_DN | |  |  |
| GSE39022_LN_VS_SPLEEN_DC_UP | |  |  |
| GSE3982_MEMORY_CD4_TCELL_VS_TH1_UP | |  |  |
| GSE17974_CTRL_VS_ACT_IL4_AND_ANTI_IL12_48H_CD4_TCELL_DN | |  |  |
| GSE22601_CD4_SINGLE_POSITIVE_VS_CD8_SINGLE_POSITIVE_THYMOCYTE_UP | |  |  |
| GSE22886_NAIVE_CD4_TCELL_VS_12H_ACT_TH1_UP | |  |  |
| GSE15735_CTRL_VS_HDAC_INHIBITOR_TREATED_CD4_TCELL_2H_DN | |  |  |
| GSE28237_FOLLICULAR_VS_EARLY_GC_BCELL_DN | |  |  |
| GSE15930_NAIVE_VS_48H_IN_VITRO_STIM_IL12_CD8_TCELL_DN | |  |  |
| GSE41176_UNSTIM_VS_ANTI_IGM_STIM_TAK1_KO_BCELL_24H_DN | |  |  |
| GSE24634_IL4_VS_CTRL_TREATED_NAIVE_CD4_TCELL_DAY10_DN | |  |  |
| GSE14415_INDUCED_TREG_VS_FOXP3_KO_INDUCED_TREG_IL2_CULTURE_UP | |  |  |
| GSE19923_E2A_KO_VS_HEB_AND_E2A_KO_DP_THYMOCYTE_DN | |  |  |
| GSE360_L_DONOVANI_VS_B_MALAYI_LOW_DOSE_DC_UP | |  |  |
| GSE12507_PDC_CELL_LINE_VS_IMMATUE_T_CELL_LINE_UP | |  |  |
| GSE26030_TH1_VS_TH17_DAY15_POST_POLARIZATION_DN | |  |  |
| GSE19888_CTRL_VS_A3R_INHIBITOR_TREATED_MAST_CELL_UP | |  |  |
| GSE37534_UNTREATED_VS_GW1929_TREATED_CD4_TCELL_PPARG1_AND_FOXP3_TRASDUCED_DN | | | |
| GSE2585_CD80_HIGH_VS_LOW_AIRE_KO_MTEC_DN | |  |  |
| GSE25088_IL4_VS_IL4_AND_ROSIGLITAZONE_STIM_STAT6_KO_MACROPHAGE_DAY10_DN | | |  |
| GSE3982_CTRL_VS_LPS_4H_MAC_DN | |  |  |
| GSE14000_UNSTIM_VS_16H_LPS_DC_TRANSLATED_RNA_DN | |  |  |
| GSE13762_CTRL_VS_125_VITAMIND_DAY12_DC_DN | |  |  |
| GSE15930_NAIVE_VS_72H_IN_VITRO_STIM_IL12_CD8_TCELL_DN | |  |  |
| GSE360_HIGH_DOSE_B_MALAYI_VS_M_TUBERCULOSIS_DC_DN | |  |  |
| GSE22140_GERMFREE_VS_SPF_ARTHRITIC_MOUSE_CD4_TCELL_UP | |  |  |
| GSE21546_UNSTIM_VS_ANTI_CD3_STIM_DP_THYMOCYTES_DN | |  |  |
| GSE14000_UNSTIM_VS_4H_LPS_DC_TRANSLATED_RNA_DN | |  |  |
| GSE360_CTRL_VS_M_TUBERCULOSIS_DC_DN | |  |  |
| GSE19923_WT_VS_HEB_AND_E2A_KO_DP_THYMOCYTE_UP | |  |  |
| GSE2770_UNTREATED_VS_TGFB_AND_IL12_TREATED_ACT_CD4_TCELL_2H_DN | |  |  |
| GSE24142_ADULT_VS_FETAL_EARLY_THYMIC_PROGENITOR_DN | |  |  |
| GSE32901_NAIVE_VS_TH17_NEG_CD4_TCELL_DN | |  |  |
| GSE25123_ROSIGLITAZONE_VS_IL4_AND_ROSIGLITAZONE_STIM_PPARG_KO_MACROPHAGE_DAY10_UP | | | |
| GSE43863_TH1_VS_LY6C_INT_CXCR5POS_EFFECTOR_CD4_TCELL_DN | |  |  |
| GSE21546_ELK1_KO_VS_SAP1A_KO_AND_ELK1_KO_DP_THYMOCYTES_DN | |  |  |
| GSE43863_TH1_VS_TFH_EFFECTOR_CD4_TCELL_DN | |  |  |
| GSE360_DC_VS_MAC_B_MALAYI_HIGH_DOSE_DN | |  |  |
| HALLMARK_MITOTIC_SPINDLE |  |  |  |
| GSE5589_LPS_VS_LPS_AND_IL10_STIM_IL10_KO_MACROPHAGE_45MIN_DN | |  |  |
| GSE15733_BM_VS_SPLEEN_MEMORY_CD4_TCELL_UP | |  |  |
| GSE20715_0H_VS_48H_OZONE_TLR4_KO_LUNG_DN | |  |  |
| GSE22589_SIV_VS_HIV_AND_SIV_INFECTED_DC_UP | |  |  |
| GSE19941_IL10_KO_VS_IL10_KO_AND_NFKBP50_KO_LPS_AND_IL10_STIM_MACROPHAGE_DN | | |  |
| GSE3039_CD4_TCELL_VS_NKT_CELL_DN | |  |  |
| GSE34156_TLR1_TLR2_LIGAND_VS_NOD2_AND_TLR1_TLR2_LIGAND_24H_TREATED_MONOCYTE_UP | | | |
| GSE45739_UNSTIM_VS_ACD3_ACD28_STIM_NRAS_KO_CD4_TCELL_UP | |  |  |
| GSE21927_SPLEEN_VS_4T1_TUMOR_MONOCYTE_BALBC_DN | |  |  |
| GSE22886_NAIVE_BCELL_VS_NEUTROPHIL_DN | |  |  |
| GSE22140_HEALTHY_VS_ARTHRITIC_GERMFREE_MOUSE_CD4_TCELL_DN | |  |  |
| GSE2706_R848_VS_R848_AND_LPS_2H_STIM_DC_DN | |  |  |
| GSE45365_WT_VS_IFNAR_KO_CD11B_DC_DN | |  |  |
| GSE2706_UNSTIM_VS_2H_R848_DC_DN | |  |  |
| GSE10325_CD4_TCELL_VS_LUPUS_CD4_TCELL_DN | |  |  |
| GSE9960_HEALTHY_VS_GRAM_POS_SEPSIS_PBMC_DN | |  |  |
| GSE17974_0.5H_VS_72H_IL4_AND_ANTI_IL12_ACT_CD4_TCELL_DN | |  |  |
| GSE360_HIGH_DOSE_B_MALAYI_VS_M_TUBERCULOSIS_MAC_DN | |  |  |
| GSE36476_CTRL_VS_TSST_ACT_16H_MEMORY_CD4_TCELL_YOUNG_DN | |  |  |
| GSE17186_CD21LOW_VS_CD21HIGH_TRANSITIONAL_BCELL_DN | |  |  |
| GSE43863_NAIVE_VS_MEMORY_TH1_CD4_TCELL_D150_LCMV_UP | |  |  |
| GSE32533_MIR17_KO_VS_MIR17_OVEREXPRESS_ACT_CD4_TCELL_UP | |  |  |
| HALLMARK_INFLAMMATORY_RESPONSE | |  |  |
| GSE21927_SPLEEN_C57BL6_VS_EL4_TUMOR_BALBC_MONOCYTES_DN | |  |  |
| GSE14908_ATOPIC_VS_NONATOPIC_PATIENT_HDM_STIM_CD4_TCELL_DN | |  |  |
| GSE24026_PD1_LIGATION_VS_CTRL_IN_ACT_TCELL_LINE_UP | |  |  |
| GSE25085_FETAL_BM_VS_ADULT_BM_SP4_THYMIC_IMPLANT_UP | |  |  |
| GSE32986_GMCSF_VS_GMCSF_AND_CURDLAN_LOWDOSE_STIM_DC_DN | |  |  |
| GSE6269_HEALTHY_VS_FLU_INF_PBMC_DN | |  |  |
| HALLMARK_IL6_JAK_STAT3_SIGNALING | |  |  |
| GSE20754_WT_VS_TCF1_KO_MEMORY_CD8_TCELL_DN | |  |  |
| GSE11961_MARGINAL_ZONE_BCELL_VS_MEMORY_BCELL_DAY40_UP | |  |  |
| GSE360_LOW_DOSE_B_MALAYI_VS_M_TUBERCULOSIS_DC_DN | |  |  |
| GSE32986_UNSTIM_VS_CURDLAN_LOWDOSE_STIM_DC_DN | |  |  |
| GSE14000_UNSTIM_VS_4H_LPS_DC_DN | |  |  |
| GSE6090_UNSTIM_VS_DC_SIGN_STIM_DC_DN | |  |  |
| GSE20727_CTRL_VS_ROS_INHIBITOR_TREATED_DC_UP | |  |  |
| GSE2706_UNSTIM_VS_2H_LPS_AND_R848_DC_DN | |  |  |
| GSE30962_PRIMARY_VS_SECONDARY_CHRONIC_LCMV_INF_CD8_TCELL_UP | |  |  |
| GSE36078_WT_VS_IL1R_KO_LUNG_DC_AFTER_AD5_INF_UP | |  |  |
| HALLMARK_MTORC1_SIGNALING | |  |  |
| GSE19888_ADENOSINE_A3R_INH_VS_ACT_WITH_INHIBITOR_PRETREATMENT_IN_MAST_CELL_UP | | | |
| GSE3039_NKT_CELL_VS_ALPHABETA_CD8_TCELL_UP | |  |  |
| GSE22886_NAIVE_CD4_TCELL_VS_48H_ACT_TH2_DN | |  |  |
| GSE27859_DC_VS_CD11C_INT_F480_INT_DC_DN | |  |  |
| GSE17974_IL4_AND_ANTI_IL12_VS_UNTREATED_24H_ACT_CD4_TCELL_DN | |  |  |
| GSE22886_DAY0_VS_DAY1_MONOCYTE_IN_CULTURE_DN | |  |  |
| GSE18791_UNSTIM_VS_NEWCATSLE_VIRUS_DC_18H_DN | |  |  |
| GSE2706_2H_VS_8H_R848_AND_LPS_STIM_DC_DN | |  |  |
| GSE21063_WT_VS_NFATC1_KO_BCELL_DN | |  |  |
| GSE3039_NKT_CELL_VS_ALPHAALPHA_CD8_TCELL_DN | |  |  |
| GSE39556_UNTREATED_VS_3H_POLYIC_INJ_MOUSE_CD8A_DC_UP | |  |  |
| GSE24026_PD1_LIGATION_VS_CTRL_IN_ACT_TCELL_LINE_DN | |  |  |
| GSE18281_SUBCAPSULAR_VS_CENTRAL_CORTICAL_REGION_OF_THYMUS_DN | |  |  |
| GSE18791_CTRL_VS_NEWCASTLE_VIRUS_DC_12H_DN | |  |  |
| GSE1791_CTRL_VS_NEUROMEDINU_IN_T_CELL_LINE_3H_UP | |  |  |
| GSE18791_CTRL_VS_NEWCASTLE_VIRUS_DC_16H_DN | |  |  |
| GSE17974_0.5H_VS_72H_UNTREATED_IN_VITRO_CD4_TCELL_DN | |  |  |
| HALLMARK_ALLOGRAFT_REJECTION | |  |  |
| GSE19888_ADENOSINE_A3R_INH_PRETREAT_AND_ACT_BY_A3R_VS_TCELL_MEMBRANES_ACT_MAST_CELL_UP | | | |
| GSE38681_WT_VS_LYL1_KO_LYMPHOID_PRIMED_MULTIPOTENT_PROGENITOR_DN | | |  |
| GSE36888_UNTREATED_VS_IL2_TREATED_TCELL_17H_DN | |  |  |
| GSE7218_IGM_VS_IGG_SIGNAL_THGOUGH_ANTIGEN_BCELL_DN | |  |  |
| GSE360_L_DONOVANI_VS_T_GONDII_DC_DN | |  |  |
| GSE22886_UNSTIM_VS_STIM_MEMORY_TCELL_DN | |  |  |
| GSE37301_MULTIPOTENT_PROGENITOR_VS_GRAN_MONO_PROGENITOR_DN | |  |  |
| GSE9960_GRAM_NEG_VS_GRAM_POS_SEPSIS_PBMC_UP | |  |  |
| GSE6269_E_COLI_VS_STREP_PNEUMO_INF_PBMC_DN | |  |  |
| GSE13485_CTRL_VS_DAY3_YF17D_VACCINE_PBMC_DN | |  |  |
| GSE40274_CTRL_VS_FOXP3_AND_HELIOS_TRANSDUCED_ACTIVATED_CD4_TCELL_DN | | |  |
| GOLDRATH_EFF_VS_MEMORY_CD8_TCELL_DN | |  |  |
| GSE8835_CD4_VS_CD8_TCELL_CLL_PATIENT_UP | |  |  |
| GSE18791_CTRL_VS_NEWCASTLE_VIRUS_DC_18H_DN | |  |  |
| GSE1112_HY_CD8AB_VS_HY_CD8AA_THYMOCYTE_RTOC_CULTURE_UP | |  |  |
| GSE7509_UNSTIM_VS_IFNA_STIM_IMMATURE_DC_UP | |  |  |
| GSE15930_NAIVE_VS_48H_IN_VITRO_STIM_CD8_TCELL_DN | |  |  |
| GSE15750_DAY6_VS_DAY10_TRAF6KO_EFF_CD8_TCELL_DN | |  |  |
| GSE16755_CTRL_VS_IFNA_TREATED_MAC_DN | |  |  |
| GSE39110_UNTREATED_VS_IL2_TREATED_CD8_TCELL_DAY6_POST_IMMUNIZATION_UP | | |  |
| GSE9946_IMMATURE_VS_PROSTAGLANDINE2_TREATED_MATURE_DC_DN | |  |  |
| GSE22886_CD8_TCELL_VS_BCELL_NAIVE_UP | |  |  |
| GSE2706_UNSTIM_VS_2H_LPS_DC_DN | |  |  |
| GSE5589_LPS_AND_IL10_VS_LPS_AND_IL6_STIM_IL6_KO_MACROPHAGE_45MIN_UP | | |  |
| GSE42021_CD24INT_VS_CD24LOW_TREG_THYMUS_DN | |  |  |
| GSE42724_NAIVE_BCELL_VS_PLASMABLAST_UP | |  |  |
| GSE1740_UNSTIM_VS_IFNA_STIMULATED_MCSF_DERIVED_MACROPHAGE_DN | |  |  |
| GSE22886_CTRL_VS_LPS_24H_DC_DN | |  |  |
| GSE1432_CTRL_VS_IFNG_6H_MICROGLIA_DN | |  |  |
| GSE18791_CTRL_VS_NEWCASTLE_VIRUS_DC_10H_DN | |  |  |
| GSE1460_DP_THYMOCYTE_VS_NAIVE_CD4_TCELL_CORD_BLOOD_UP | |  |  |
| GSE1432_CTRL_VS_IFNG_24H_MICROGLIA_DN | |  |  |
| GSE18791_CTRL_VS_NEWCASTLE_VIRUS_DC_4H_DN | |  |  |
| GSE17974_0H_VS_48H_IN_VITRO_ACT_CD4_TCELL_DN | |  |  |
| GSE17974_IL4_AND_ANTI_IL12_VS_UNTREATED_72H_ACT_CD4_TCELL_DN | |  |  |
| GSE21546_WT_VS_ELK1_KO_ANTI_CD3_STIM_DP_THYMOCYTES_DN | |  |  |
| GSE18791_UNSTIM_VS_NEWCATSLE_VIRUS_DC_10H_DN | |  |  |
| GSE13485_PRE_VS_POST_YF17D_VACCINATION_PBMC_DN | |  |  |
| GSE24634_NAIVE_CD4_TCELL_VS_DAY10_IL4_CONV_TREG_DN | |  |  |
| GSE46606_UNSTIM_VS_CD40L_IL2_IL5_DAY1_STIMULATED_BCELL_DN | |  |  |
| GSE17974_0H_VS_12H_IN_VITRO_ACT_CD4_TCELL_DN | |  |  |
| GSE19941_LPS_VS_LPS_AND_IL10_STIM_IL10_KO_MACROPHAGE_DN | |  |  |
| GSE7509_DC_VS_MONOCYTE_WITH_FCGRIIB_STIM_DN | |  |  |
| GSE2770_IL12_AND_TGFB_ACT_VS_ACT_CD4_TCELL_48H_DN | |  |  |
| GSE18791_CTRL_VS_NEWCASTLE_VIRUS_DC_6H_DN | |  |  |
| GSE19888_ADENOSINE_A3R_ACT_VS_A3R_ACT_WITH_A3R_INH_PRETREATMENT_IN_MAST_CELL_UP | | | |
| GSE37534_UNTREATED_VS_PIOGLITAZONE_TREATED_CD4_TCELL_PPARG1_AND_FOXP3_TRASDUCED_DN | | | |
| GSE18791_CTRL_VS_NEWCASTLE_VIRUS_DC_8H_DN | |  |  |
| GSE7218_UNSTIM_VS_ANTIGEN_STIM_THROUGH_IGG_BCELL_UP | |  |  |
| GSE22886_NAIVE_CD4_TCELL_VS_12H_ACT_TH1_DN | |  |  |
| GSE37532_TREG_VS_TCONV_PPARG_KO_CD4_TCELL_FROM_LN_UP | |  |  |
| GSE42724_MEMORY_VS_B1_BCELL_DN | |  |  |
| GSE18281_CORTICAL_VS_MEDULLARY_THYMOCYTE_UP | |  |  |
| GSE13547_CTRL_VS_ANTI_IGM_STIM_ZFX_KO_BCELL_12H_DN | |  |  |
| GSE36888_UNTREATED_VS_IL2_TREATED_STAT5_AB_KNOCKIN_TCELL_2H_UP | |  |  |
| GSE42021_TREG_PLN_VS_CD24LO_TREG_THYMUS_DN | |  |  |
| GSE24634_NAIVE_CD4_TCELL_VS_DAY7_IL4_CONV_TREG_UP | |  |  |
| HALLMARK_INTERFERON_ALPHA_RESPONSE | |  |  |
| GSE7548_NAIVE_VS_DAY7_PCC_IMMUNIZATION_CD4_TCELL_DN | |  |  |
| GSE2770_UNTREATED_VS_IL4_TREATED_ACT_CD4_TCELL_2H_DN | |  |  |
| GSE37532_TREG_VS_TCONV_CD4_TCELL_FROM_VISCERAL_ADIPOSE_TISSUE_DN | |  |  |
| GSE37532_VISCERAL_ADIPOSE_TISSUE_VS_LN_DERIVED_PPARG_KO_TCONV_CD4_TCELL_UP | | |  |
| GSE19888_CTRL_VS_TCELL_MEMBRANES_ACT_MAST_CELL_PRETREAT_A3R_INH_DN | | |  |
| GSE3982_CENT_MEMORY_CD4_TCELL_VS_TH2_UP | |  |  |
| GSE10240_CTRL_VS_IL17_AND_IL22_STIM_PRIMARY_BRONCHIAL_EPITHELIAL_CELLS_DN | | |  |
| GSE18791_UNSTIM_VS_NEWCATSLE_VIRUS_DC_6H_DN | |  |  |
| GSE19888_ADENOSINE_A3R_INH_VS_TCELL_MEMBRANES_ACT_MAST_CELL_UP | |  |  |
| GSE33424_CD161_INT_VS_NEG_CD8_TCELL_UP | |  |  |
| GSE29614_CTRL_VS_DAY3_TIV_FLU_VACCINE_PBMC_DN | |  |  |
| GSE10239_MEMORY_VS_KLRG1HIGH_EFF_CD8_TCELL_DN | |  |  |
| GSE27241_CTRL_VS_DIGOXIN_TREATED_RORGT_KO_CD4_TCELL_IN_TH17_POLARIZING_CONDITIONS_UP | | | |
| HALLMARK_TGF_BETA_SIGNALING | |  |  |
| GSE18893_TCONV_VS_TREG_2H_TNF_STIM_DN | |  |  |
| GSE15330_LYMPHOID_MULTIPOTENT_VS_MEGAKARYOCYTE_ERYTHROID_PROGENITOR_IKAROS_KO_UP | | | |
| GSE15930_NAIVE_VS_48H_IN_VITRO_STIM_IFNAB_CD8_TCELL_DN | |  |  |
| GSE36476_CTRL_VS_TSST_ACT_40H_MEMORY_CD4_TCELL_YOUNG_UP | |  |  |
| GSE7460_CD8_TCELL_VS_TREG_ACT_DN | |  |  |
| GSE26030_TH1_VS_TH17_DAY5_POST_POLARIZATION_UP | |  |  |
| GSE21360_SECONDARY_VS_TERTIARY_MEMORY_CD8_TCELL_UP | |  |  |
| GSE25123_CTRL_VS_ROSIGLITAZONE_STIM_PPARG_KO_MACROPHAGE_UP | |  |  |
| GSE16451_IMMATURE_VS_MATURE_NEURON_CELL_LINE_WEST_EQUINE_ENC_VIRUS_DN | | |  |
| GSE17974_0H_VS_48H_IN_VITRO_ACT_CD4_TCELL_UP | |  |  |
| GSE17974_0H_VS_12H_IN_VITRO_ACT_CD4_TCELL_UP | |  |  |
| GSE24142_EARLY_THYMIC_PROGENITOR_VS_DN2_THYMOCYTE_UP | |  |  |
| GSE21670_STAT3_KO_VS_WT_CD4_TCELL_TGFB_IL6_TREATED_DN | |  |  |
| GSE24142_EARLY_THYMIC_PROGENITOR_VS_DN2_THYMOCYTE_ADULT_UP | |  |  |
| GSE43863_TH1_VS_LY6C_INT_CXCR5POS_MEMORY_CD4_TCELL_DN | |  |  |
| GSE39556_UNTREATED_VS_3H_POLYIC_INJ_MOUSE_CD8A_DC_DN | |  |  |
| GSE16450_IMMATURE_VS_MATURE_NEURON_CELL_LINE_UP | |  |  |
| GSE37532_VISCERAL_ADIPOSE_TISSUE_VS_LN_DERIVED_TCONV_CD4_TCELL_DN | |  |  |
| GSE39556_CD8A_DC_VS_NK_CELL_DN | |  |  |
| GSE19198_1H_VS_24H_IL21_TREATED_TCELL_UP | |  |  |
| GSE24142_EARLY_THYMIC_PROGENITOR_VS_DN3_THYMOCYTE_FETAL_UP | |  |  |
| GSE17974_CTRL_VS_ACT_IL4_AND_ANTI_IL12_48H_CD4_TCELL_UP | |  |  |
| GSE36826_WT_VS_IL1R_KO_SKIN_UP | |  |  |
| GSE42088_2H_VS_24H_LEISHMANIA_INF_DC_UP | |  |  |
| GSE1460_DP_VS_CD4_THYMOCYTE_DN | |  |  |
| GSE36891_UNSTIM_VS_POLYIC_TLR3_STIM_PERITONEAL_MACROPHAGE_UP | |  |  |
| GSE29618_BCELL_VS_PDC_UP |  |  |  |
| GSE1460_INTRATHYMIC_T_PROGENITOR_VS_CD4_THYMOCYTE_DN | |  |  |
| GSE14769_UNSTIM_VS_80MIN_LPS_BMDM_DN | |  |  |
| GSE36891_POLYIC_TLR3_VS_PAM_TLR2_STIM_PERITONEAL_MACROPHAGE_UP | |  |  |
