# Supplementary material for: NXPH4 Used as a New Prognostic and Immunotherapeutic Marker for Muscle-Invasive Bladder Cancer
Source: J Oncol. 2022 Oct 4;2022:4271409. doi: 10.1155/2022/4271409 (PMC9553512; doi:10.1155/2022/4271409)
Supplement: Supplementary Materials — Figure s1: article roadmap of the whole research. Figure s2: (A) GSVA results heatmap of invasive bladder cancer in TCGA database (normal =19, tumor =404); Wayne diagram of differential pathways between clusters. (B) Wayne diagram in TCGA clusters (n = 65). (C) Wayne diagram in GEO clusters (n = 463). (D) Wayne diagram in TCGA clusters and GEO clusters (n = 6). Figure s3: (A) the 28 prognostic key pathway genes (P <0.01). Risk model for patients with muscle invasive bladder cancer (MIBC) based on 12 genes (SLC7A2, MST1R, CDK6, NXPH4, GRIK2, TRIB3, PBK, ABCA4, FBN2, SCG2, ELN, and INCENP). (B) LASSO regression with 10-fold crossvalidation was used to obtain 12 prognostic genes with an error within one standard error of the minimum (lambda.1se). (C) LASSO coefficient profiles of 28 key pathway genes. Supplement Table 1: clinical characteristics such as N, M, T, tumor grade, and stage, including age among the three groups (TCGA). Supplement Table 2: survival and prognosis information of three groups based on GEO. Supplement Table 3: 65 differential pathways were obtained from the molecular subtypes of TCGA queue. Supplement Table 4: 6 common differential pathways were obtained based on 65 TCGA, differential pathways, and 463 GEO, differential pathways. Supplement Table 5: 6 common differential pathways with prognosis. Supplement Table 6: the risk model based on the 12 prognostic genes in TCGA and GEO databases. Supplement Table 7: immune landscape between the high- and low-risk patients with muscle invasive bladder cancer (MIBC). Supplement Table 8: evaluation of immune response to CTLA4 and PD1 immunosuppressants in MIBC patients. [file 4271409.f1.zip › supplement table5.docx]

**Supplement Table5** 6 common differential pathways with prognosis

| GEO-6 commom dif with survival | | | | | | | | | TCGA-STAT3 | | | | GEO-STAT3 | | | |
| --- | --- | --- | --- | --- | --- | --- | --- | --- | --- | --- | --- | --- | --- | --- | --- | --- |
| id | futime | fustat | GSE21670_STAT3_KO_VS_WT_CD4_TCELL_TGFB_IL6_TREATED_DN | GSE1460_INTRATHYMIC_T_PROGENITOR_VS_CD4_THYMOCYTE_DN | GSE17974_IL4_AND_ANTI_IL12_VS_UNTREATED_72H_ACT_CD4_TCELL_DN | GSE39556_UNTREATED_VS_3H_POLYIC_INJ_MOUSE_CD8A_DC_UP | GSE42088_2H_VS_24H_LEISHMANIA_INF_DC_UP | GSE29618_BCELL_VS_PDC_UP | Cluster | ID | STAT3 | average-cluster | geneNames | STAT3 | cluster |  |
| GSM814052 | 9.1917808 | 0 | 0.1 | 0.02 | 0.1 | 0 | 0.2 | 0.1 | C1 | TCGA-2F-A9KO | 32.087 | 23 | GSM340613 | 3.44 | C1 | average-cluster |
| GSM814056 | 3.03 | 1 | 0.2 | 0.05 | 0.1 | 0.1 | 0.27 | 0.2 | C1 | TCGA-4Z-AA7N | 30.999 | 28 | GSM340618 | 4.65 | C1 | 3.929 |
| GSM814058 | 5.3260274 | 0 | 0.1 | 0.04 | 0.1 | -0 | 0.24 | 0.2 | C1 | TCGA-4Z-AA7O | 18.41 | 15 | GSM340628 | 4.81 | C1 | 4.748 |
| GSM814060 | 0.4493151 | 1 | 0.2 | 0.16 | 0.2 | 0.1 | 0.33 | 0.2 | C1 | TCGA-4Z-AA7Q | 29.804 |  | GSM340653 | 4.58 | C1 | 5.202 |
| GSM814061 | 1.0684932 | 0 | 0.1 | 0.09 | 0.1 | 0 | 0.21 | 0.1 | C1 | TCGA-4Z-AA7W | 20.855 |  | GSM340654 | 4.58 | C1 |  |
| GSM814064 | 6.3315068 | 0 | 0.2 | 0.13 | 0.2 | 0.1 | 0.32 | 0.2 | C1 | TCGA-4Z-AA81 | 23.156 |  | GSM340673 | 4.7 | C1 |  |
| GSM814065 | 8.6493151 | 0 | 0.1 | 0.08 | 0.1 | 0 | 0.24 | 0.2 | C1 | TCGA-4Z-AA83 | 24.14 |  | GSM340677 | 5.59 | C1 |  |
| GSM814066 | 5.5205479 | 0 | 0.1 | 0.1 | 0 | 0 | 0.25 | 0.1 | C1 | TCGA-4Z-AA87 | 7.3258 |  | GSM340678 | 4.17 | C1 |  |
| GSM814068 | 8.0328767 | 0 | 0.4 | 0.24 | 0.2 | 0.1 | 0.44 | 0.3 | C1 | TCGA-4Z-AA89 | 19.767 |  | GSM340693 | 5.1 | C1 |  |
| GSM814069 | 6.169863 | 0 | 0.1 | 0.1 | 0.1 | 0 | 0.25 | 0.2 | C1 | TCGA-5N-A9KM | 28.394 |  | GSM340696 | 5.78 | C1 |  |
| GSM814070 | 2.1315068 | 0 | 0.3 | 0.22 | 0.2 | 0.1 | 0.38 | 0.2 | C1 | TCGA-BT-A20W | 15.38 |  | GSM340701 | 5.22 | C1 |  |
| GSM814074 | 5.2493151 | 0 | 0.1 | 0.13 | 0.1 | 0 | 0.31 | 0.2 | C1 | TCGA-BT-A42E | 27.588 |  | GSM340704 | 4.98 | C1 |  |
| GSM814076 | 1.4931507 | 1 | 0.2 | 0.16 | 0.2 | 0.1 | 0.34 | 0.2 | C1 | TCGA-BT-A42F | 27.632 |  | GSM340709 | 4.13 | C1 |  |
| GSM814084 | 5.5589041 | 0 | 0.2 | 0.15 | 0.2 | 0 | 0.29 | 0.2 | C1 | TCGA-CF-A1HR | 25.379 |  | GSM340721 | 4.61 | C1 |  |
| GSM814085 | 8.1424658 | 0 | 0.2 | 0.1 | 0.1 | 0.1 | 0.29 | 0.2 | C1 | TCGA-CF-A27C | 11.522 |  | GSM340725 | 5.06 | C1 |  |
| GSM814086 | 5.7041096 | 0 | 0.2 | 0.13 | 0.1 | 0.1 | 0.3 | 0.2 | C1 | TCGA-CF-A3MF | 3.9565 |  | GSM340728 | 5.24 | C1 |  |
| GSM814087 | 6.8273973 | 1 | 0.2 | 0.11 | 0.1 | 0 | 0.27 | 0.2 | C1 | TCGA-CF-A3MH | 10.728 |  | GSM340731 | 5.15 | C1 |  |
| GSM814090 | 6.5369863 | 0 | 0.2 | 0.16 | 0.2 | 0.1 | 0.32 | 0.2 | C1 | TCGA-CF-A3MI | 8.8118 |  | GSM340732 | 4.58 | C1 |  |
| GSM814091 | 2.9780822 | 0 | 0.1 | 0.11 | 0.1 | 0 | 0.24 | 0.2 | C1 | TCGA-CF-A47S | 12.8 |  | GSM340745 | 5.38 | C1 |  |
| GSM814092 | 2.2109589 | 1 | 0.2 | 0.17 | 0.2 | 0.1 | 0.33 | 0.2 | C1 | TCGA-CF-A47V | 11.134 |  | GSM340757 | 4.29 | C1 |  |
| GSM814094 | 2.4575342 | 1 | 0.2 | 0.15 | 0.2 | 0.1 | 0.28 | 0.2 | C1 | TCGA-CF-A47W | 10.682 |  | GSM340760 | 4.91 | C1 |  |
| GSM814095 | 7.1178082 | 0 | 0.2 | 0.12 | 0.2 | 0 | 0.28 | 0.2 | C1 | TCGA-CF-A47X | 21.204 |  | GSM340766 | 4.71 | C1 |  |
| GSM814098 | 6.1561644 | 0 | 0.2 | 0.16 | 0.2 | 0.1 | 0.33 | 0.3 | C1 | TCGA-CF-A47Y | 10.585 |  | GSM786491 | 4.19 | C1 |  |
| GSM814100 | 7.3835616 | 0 | 0.2 | 0.11 | 0.2 | 0 | 0.27 | 0.2 | C1 | TCGA-CF-A5U8 | 12.307 |  | GSM786494 | 5.32 | C1 |  |
| GSM814102 | 6.0164384 | 0 | 0.2 | 0.17 | 0.2 | 0.1 | 0.33 | 0.3 | C1 | TCGA-CF-A7I0 | 11.974 |  | GSM786500 | 4.79 | C1 |  |
| GSM814108 | 1.7589041 | 0 | 0.2 | 0.16 | 0.2 | 0.1 | 0.33 | 0.2 | C1 | TCGA-CF-A8HX | 5.5674 |  | GSM786503 | 5.52 | C1 |  |
| GSM814109 | 5.7917808 | 0 | 0.3 | 0.31 | 0.3 | 0.2 | 0.41 | 0.4 | C1 | TCGA-CF-A9FF | 9.5813 |  | GSM786504 | 5 | C1 |  |
| GSM814110 | 4.5287671 | 0 | 0.1 | 0.09 | 0.1 | -0 | 0.26 | 0.1 | C1 | TCGA-CF-A9FH | 22.092 |  | GSM786506 | 5.1 | C1 |  |
| GSM814112 | 7.0767123 | 0 | 0.1 | 0.09 | 0.1 | -0 | 0.26 | 0.2 | C1 | TCGA-CU-A3YL | 27.728 |  | GSM786509 | 5.81 | C1 |  |
| GSM814118 | 4.5260274 | 0 | 0.1 | 0.08 | 0.1 | -0 | 0.28 | 0.2 | C1 | TCGA-DK-A1A6 | 12.875 |  | GSM786511 | 5.46 | C1 |  |
| GSM814119 | 7.3452055 | 0 | 0.1 | 0.14 | 0.1 | 0.1 | 0.26 | 0.2 | C1 | TCGA-DK-A1A7 | 13.947 |  | GSM786512 | 5.34 | C1 |  |
| GSM814122 | 6.9041096 | 0 | 0.2 | 0.12 | 0.1 | 0 | 0.27 | 0.2 | C1 | TCGA-DK-A1AA | 24.023 |  | GSM786513 | 4.1 | C1 |  |
| GSM814126 | 7.2027397 | 0 | 0.2 | 0.12 | 0.2 | 0.1 | 0.27 | 0.2 | C1 | TCGA-DK-A1AC | 18.103 |  | GSM786514 | 5.47 | C1 |  |
| GSM814132 | 0.5557078 | 0 | 0.2 | 0.23 | 0.3 | 0.1 | 0.33 | 0.3 | C1 | TCGA-DK-A1AD | 17.238 |  | GSM786519 | 5.57 | C1 |  |
| GSM814133 | 4.1123288 | 0 | 0.2 | 0.15 | 0.2 | 0 | 0.29 | 0.2 | C1 | TCGA-DK-A1AG | 21.714 |  | GSM786520 | 4.98 | C1 |  |
| GSM814134 | 2.9972603 | 0 | 0.1 | 0.12 | 0.1 | -0 | 0.27 | 0.2 | C1 | TCGA-DK-A3IK | 12.235 |  | GSM786521 | 3.81 | C1 |  |
| GSM814135 | 6.0465753 | 0 | 0.3 | 0.21 | 0.2 | 0.1 | 0.34 | 0.3 | C1 | TCGA-DK-A3IV | 32.587 |  | GSM786522 | 4.84 | C1 |  |
| GSM814136 | 1.0356164 | 0 | 0.1 | 0.04 | 0.1 | 0 | 0.31 | 0.1 | C1 | TCGA-DK-A3WW | 20.924 |  | GSM786525 | 4.65 | C1 |  |
| GSM814138 | 0.5150685 | 1 | 0.2 | 0.16 | 0.2 | 0.1 | 0.33 | 0.3 | C1 | TCGA-DK-A3WY | 26.995 |  | GSM786528 | 5.3 | C1 |  |
| GSM814142 | 0.6273973 | 1 | 0.1 | 0 | 0 | 0 | 0.23 | 0.1 | C1 | TCGA-DK-A6AW | 16.622 |  | GSM786532 | 4.79 | C1 |  |
| GSM814149 | 0.8410959 | 0 | 0.1 | 0.1 | 0.1 | 0 | 0.26 | 0.2 | C1 | TCGA-DK-A6B0 | 16.903 |  | GSM786543 | 5.34 | C1 |  |
| GSM814150 | 3.2986301 | 0 | 0.1 | 0.09 | 0.1 | 0 | 0.29 | 0.2 | C1 | TCGA-DK-A6B6 | 22.775 |  | GSM786550 | 4.57 | C1 |  |
| GSM814153 | 3.7780822 | 0 | 0.2 | 0.07 | 0.1 | 0 | 0.27 | 0.2 | C1 | TCGA-DK-AA6L | 29.416 |  | GSM786553 | 5.08 | C1 |  |
| GSM814155 | 5.9835616 | 0 | 0.1 | 0.09 | 0.2 | -0 | 0.24 | 0.2 | C1 | TCGA-DK-AA6T | 10.944 |  | GSM786557 | 5.61 | C1 |  |
| GSM814156 | 6.5534247 | 0 | 0.3 | 0.24 | 0.2 | 0.1 | 0.36 | 0.3 | C1 | TCGA-DK-AA6X | 35.833 |  | GSM786560 | 5.46 | C1 |  |
| GSM814160 | 5.9232877 | 0 | 0.2 | 0.11 | 0.1 | 0 | 0.29 | 0.3 | C1 | TCGA-DK-AA77 | 25.197 |  | GSM786562 | 5.53 | C1 |  |
| GSM814162 | 5.7917808 | 0 | 0.1 | 0.09 | 0.1 | -0 | 0.25 | 0.2 | C1 | TCGA-E5-A4U1 | 7.5938 |  | GSM786564 | 4.15 | C1 |  |
| GSM814164 | 5.8054795 | 0 | 0.1 | 0.07 | 0.1 | -0 | 0.24 | 0.1 | C1 | TCGA-E7-A3X6 | 40.419 |  | GSM786580 | 4.08 | C1 |  |
| GSM814167 | 3.5917808 | 0 | 0.2 | 0.11 | 0.1 | 0 | 0.27 | 0.2 | C1 | TCGA-E7-A3Y1 | 11.367 |  | GSM786582 | 4.81 | C1 |  |
| GSM814172 | 1.7178082 | 1 | 0.1 | 0.12 | 0.2 | 0.1 | 0.29 | 0.2 | C1 | TCGA-E7-A4IJ | 33.593 |  | GSM814052 | 4.12 | C1 |  |
| GSM814173 | 2.4986301 | 0 | 0.3 | 0.18 | 0.3 | 0.2 | 0.4 | 0.3 | C1 | TCGA-E7-A519 | 9.7815 |  | GSM814056 | 5.21 | C1 |  |
| GSM814174 | 1.2246575 | 0 | 0.1 | 0.09 | 0.1 | 0 | 0.27 | 0.2 | C1 | TCGA-E7-A541 | 16.013 |  | GSM814060 | 5.42 | C1 |  |
| GSM814175 | 2.8 | 0 | 0.2 | 0.2 | 0.2 | 0.2 | 0.34 | 0.3 | C1 | TCGA-E7-A5KF | 22.633 |  | GSM814064 | 4.37 | C1 |  |
| GSM814176 | 5.8273973 | 0 | 0.2 | 0.17 | 0.2 | 0.1 | 0.35 | 0.3 | C1 | TCGA-E7-A677 | 26.948 |  | GSM814070 | 5.07 | C1 |  |
| GSM814179 | 1.4739726 | 1 | 0.2 | 0.16 | 0.2 | 0.1 | 0.35 | 0.2 | C1 | TCGA-E7-A678 | 13.142 |  | GSM814076 | 5.29 | C1 |  |
| GSM814180 | 1.1671233 | 0 | 0.2 | 0.12 | 0.2 | 0 | 0.31 | 0.2 | C1 | TCGA-E7-A6MD | 25.427 |  | GSM814085 | 4.76 | C1 |  |
| GSM814182 | 0.5726027 | 1 | 0.2 | 0.15 | 0.2 | 0.1 | 0.35 | 0.2 | C1 | TCGA-E7-A6MF | 26.614 |  | GSM814090 | 4.91 | C1 |  |
| GSM814185 | 2.569863 | 0 | 0.1 | 0.09 | 0.1 | 0 | 0.26 | 0.2 | C1 | TCGA-E7-A7DU | 12.85 |  | GSM814092 | 5.15 | C1 |  |
| GSM814189 | 4.7972603 | 0 | 0.3 | 0.25 | 0.2 | 0.2 | 0.4 | 0.3 | C1 | TCGA-E7-A7PW | 10.102 |  | GSM814094 | 4.54 | C1 |  |
| GSM814190 | 4.7123288 | 0 | 0.1 | 0.07 | 0.1 | 0 | 0.27 | 0.2 | C1 | TCGA-E7-A7XN | 32.735 |  | GSM814108 | 5.88 | C1 |  |
| GSM814191 | 5.3863014 | 0 | 0.1 | 0.09 | 0.1 | -0 | 0.29 | 0.2 | C1 | TCGA-E7-A85H | 10.081 |  | GSM814119 | 4.17 | C1 |  |
| GSM814192 | 5.3671233 | 0 | 0.1 | 0.11 | 0.1 | 0 | 0.28 | 0.2 | C1 | TCGA-E7-A8O7 | 20.846 |  | GSM814126 | 4.67 | C1 |  |
| GSM814193 | 4.8356164 | 0 | 0.2 | 0.12 | 0.1 | 0 | 0.29 | 0.2 | C1 | TCGA-E7-A8O8 | 5.6751 |  | GSM814132 | 5.45 | C1 |  |
| GSM814194 | 4.5342466 | 0 | 0.1 | 0.09 | 0.1 | -0 | 0.25 | 0.2 | C1 | TCGA-E7-A97Q | 11.84 |  | GSM814136 | 4.18 | C1 |  |
| GSM814195 | 4.8356164 | 0 | 0.1 | 0.07 | 0.1 | 0 | 0.23 | 0.2 | C1 | TCGA-FD-A43P | 21.596 |  | GSM814138 | 5.38 | C1 |  |
| GSM814196 | 4.2246575 | 0 | 0.1 | 0.09 | 0.1 | 0 | 0.25 | 0.2 | C1 | TCGA-FD-A43S | 21.246 |  | GSM814172 | 5.98 | C1 |  |
| GSM814197 | 3.0273973 | 0 | 0.2 | 0.22 | 0.1 | 0.2 | 0.37 | 0.2 | C1 | TCGA-FD-A43U | 15.057 |  | GSM814173 | 5.33 | C1 |  |
| GSM814200 | 1.1424658 | 0 | 0.2 | 0.15 | 0.2 | 0.1 | 0.31 | 0.2 | C1 | TCGA-FD-A43X | 21.274 |  | GSM814175 | 4.81 | C1 |  |
| GSM814201 | 5.0876712 | 0 | 0.1 | 0.1 | 0.1 | 0 | 0.26 | 0.2 | C1 | TCGA-FD-A5BS | 19.706 |  | GSM814182 | 4.33 | C1 |  |
| GSM814202 | 0.5315068 | 1 | 0.1 | 0.04 | 0.1 | 0 | 0.2 | 0.1 | C1 | TCGA-FD-A6TE | 26.275 |  | GSM814197 | 5.06 | C1 |  |
| GSM814203 | 5.4438356 | 0 | 0.1 | 0.11 | 0.2 | 0 | 0.25 | 0.2 | C1 | TCGA-FJ-A3ZF | 6.6314 |  | GSM814202 | 4.66 | C1 |  |
| GSM814204 | 2.0164384 | 1 | 0.3 | 0.21 | 0.2 | 0.1 | 0.36 | 0.2 | C1 | TCGA-G2-A2EF | 32.953 |  | GSM814204 | 5.14 | C1 |  |
| GSM814205 | 5.3232877 | 0 | 0.2 | 0.12 | 0.2 | -0 | 0.29 | 0.2 | C1 | TCGA-G2-A2EK | 21.157 |  | GSM814218 | 5.62 | C1 |  |
| GSM814208 | 5.3315068 | 0 | 0.1 | 0.06 | 0.1 | -0 | 0.24 | 0.1 | C1 | TCGA-G2-A3IE | 12.933 |  | GSM814225 | 5.04 | C1 |  |
| GSM814210 | 5.1424658 | 0 | 0.1 | 0.06 | 0 | -0 | 0.23 | 0.2 | C1 | TCGA-G2-AA3B | 12.225 |  | GSM814228 | 4.59 | C1 |  |
| GSM814211 | 2.3808219 | 0 | 0.1 | 0.05 | 0.1 | -0 | 0.26 | 0.1 | C1 | TCGA-G2-AA3D | 17.567 |  | GSM814230 | 3.97 | C1 |  |
| GSM814212 | 5.2109589 | 0 | 0.2 | 0.09 | 0.1 | 0 | 0.33 | 0.2 | C1 | TCGA-GC-A3BM | 31.775 |  | GSM814238 | 4.72 | C1 |  |
| GSM814213 | 1.690411 | 0 | 0.1 | 0.09 | 0.1 | 0 | 0.29 | 0.2 | C1 | TCGA-GC-A3WC | 31.85 |  | GSM814240 | 4.36 | C1 |  |
| GSM814215 | 4.7232877 | 0 | 0.2 | 0.1 | 0.1 | 0 | 0.31 | 0.2 | C1 | TCGA-GC-A6I1 | 22.924 |  | GSM814241 | 5.64 | C1 |  |
| GSM814217 | 4.9671233 | 0 | 0.2 | 0.2 | 0.1 | 0.1 | 0.34 | 0.2 | C1 | TCGA-GC-A6I3 | 12.494 |  | GSM814246 | 6.08 | C1 |  |
| GSM814218 | 2.8986301 | 1 | 0.2 | 0.13 | 0.2 | 0.1 | 0.3 | 0.2 | C1 | TCGA-GD-A76B | 27.904 |  | GSM814248 | 4.41 | C1 |  |
| GSM814221 | 0.2684932 | 0 | 0.1 | 0.09 | 0.1 | -0 | 0.28 | 0.1 | C1 | TCGA-GU-A763 | 11.316 |  | GSM814251 | 4.79 | C1 |  |
| GSM814224 | 4.1561644 | 0 | 0.2 | 0.09 | 0.1 | 0 | 0.27 | 0.2 | C1 | TCGA-GV-A3JV | 15.948 |  | GSM814254 | 5.13 | C1 |  |
| GSM814225 | 1.1178082 | 1 | 0.2 | 0.12 | 0.2 | 0.1 | 0.27 | 0.2 | C1 | TCGA-GV-A3QK | 20.511 |  | GSM814264 | 4.98 | C1 |  |
| GSM814226 | 1.7643836 | 0 | 0.2 | 0.12 | 0.1 | 0 | 0.31 | 0.2 | C1 | TCGA-GV-A6ZA | 15.455 |  | GSM814269 | 4.86 | C1 |  |
| GSM814228 | 4.5287671 | 0 | 0.1 | 0.12 | 0.2 | 0.1 | 0.26 | 0.2 | C1 | TCGA-H4-A2HO | 8.7735 |  | GSM814271 | 4.83 | C1 |  |
| GSM814229 | 4.8 | 0 | 0.1 | 0.07 | 0.1 | -0 | 0.25 | 0.2 | C1 | TCGA-K4-A5RJ | 45.217 |  | GSM814275 | 4.63 | C1 |  |
| GSM814230 | 4.2383562 | 0 | 0.1 | 0.06 | 0.1 | 0 | 0.27 | 0.2 | C1 | TCGA-K4-A83P | 27.485 |  | GSM814282 | 4.87 | C1 |  |
| GSM814231 | 3.9342466 | 0 | 0.1 | 0.09 | 0.1 | -0 | 0.22 | 0.2 | C1 | TCGA-KQ-A41O | 6.6095 |  | GSM814284 | 4.47 | C1 |  |
| GSM814234 | 4.5863014 | 0 | 0.2 | 0.15 | 0.2 | 0.1 | 0.31 | 0.2 | C1 | TCGA-KQ-A41R | 6.8169 |  | GSM814287 | 5.38 | C1 |  |
| GSM814238 | 0.3068493 | 1 | 0.3 | 0.16 | 0.2 | 0.1 | 0.37 | 0.2 | C1 | TCGA-LT-A5Z6 | 10.912 |  | GSM814289 | 5.77 | C1 |  |
| GSM814240 | 4.6849315 | 0 | 0.2 | 0.14 | 0.1 | 0.1 | 0.29 | 0.2 | C1 | TCGA-LT-A8JT | 14.383 |  | GSM814291 | 4.32 | C1 |  |
| GSM814241 | 0.539726 | 1 | 0.3 | 0.22 | 0.3 | 0.1 | 0.45 | 0.3 | C1 | TCGA-S5-A6DX | 9.0013 |  | GSM814295 | 5.02 | C1 |  |
| GSM814246 | 4.4767123 | 0 | 0.2 | 0.13 | 0.2 | 0.1 | 0.34 | 0.2 | C1 | TCGA-S5-AA26 | 7.0706 |  | GSM814298 | 5.22 | C1 |  |
| GSM814248 | 3.4219178 | 0 | 0.1 | 0.06 | 0.1 | 0.1 | 0.29 | 0.1 | C1 | TCGA-UY-A78K | 17.807 |  | GSM814300 | 4.29 | C1 |  |
| GSM814250 | 1.309589 | 0 | 0.2 | 0.12 | 0.1 | 0 | 0.33 | 0.2 | C1 | TCGA-UY-A78O | 11.166 |  | GSM814303 | 5.04 | C1 |  |
| GSM814251 | 3.3917808 | 0 | 0.3 | 0.14 | 0.2 | 0.1 | 0.38 | 0.2 | C1 | TCGA-UY-A9PA | 31.551 |  | GSM814307 | 4.81 | C1 |  |
| GSM814252 | 3.0876712 | 0 | 0.1 | 0.07 | 0.1 | -0 | 0.25 | 0.2 | C1 | TCGA-UY-A9PH | 18.202 |  | GSM814313 | 5.36 | C1 |  |
| GSM814253 | 3.1479452 | 0 | 0.1 | 0.06 | 0.1 | -0 | 0.26 | 0.2 | C1 | TCGA-XF-A8HD | 28.224 |  | GSM814317 | 5.66 | C1 |  |
| GSM814254 | 2.7424658 | 0 | 0.2 | 0.1 | 0.2 | 0.1 | 0.33 | 0.2 | C1 | TCGA-XF-A8HG | 16.445 |  | GSM814322 | 5.61 | C1 |  |
| GSM814256 | 3.7726027 | 0 | 0.1 | 0.08 | 0.1 | 0 | 0.27 | 0.2 | C1 | TCGA-XF-A8HI | 18.575 |  | GSM814325 | 4.62 | C1 |  |
| GSM814261 | 3.509589 | 0 | 0.1 | 0.11 | 0.1 | 0 | 0.27 | 0.2 | C1 | TCGA-XF-A9SH | 12.5 |  | GSM814328 | 4.66 | C1 |  |
| GSM814263 | 2.2520548 | 0 | 0.2 | 0.1 | 0.1 | 0 | 0.27 | 0.2 | C1 | TCGA-XF-A9SI | 26.14 |  | GSM814330 | 4.41 | C1 |  |
| GSM814264 | 3.7287671 | 0 | 0.3 | 0.24 | 0.3 | 0.2 | 0.4 | 0.4 | C1 | TCGA-XF-A9T5 | 23.499 |  | GSM814331 | 5.82 | C1 |  |
| GSM814266 | 3.4383562 | 0 | 0.2 | 0.16 | 0.1 | 0 | 0.33 | 0.2 | C1 | TCGA-XF-AAML | 18.668 |  | GSM814333 | 5.42 | C1 |  |
| GSM814267 | 3.6712329 | 0 | 0.1 | 0.09 | 0.1 | 0 | 0.25 | 0.2 | C1 | TCGA-XF-AAMQ | 29.423 |  | GSM814334 | 5.03 | C1 |  |
| GSM814269 | 1.2986301 | 1 | 0.2 | 0.1 | 0.1 | 0 | 0.26 | 0.2 | C1 | TCGA-XF-AAMZ | 11.449 |  | GSM814347 | 4.07 | C1 |  |
| GSM814271 | 3.4027397 | 0 | 0.1 | 0.14 | 0.1 | 0 | 0.31 | 0.2 | C1 | TCGA-XF-AAN0 | 42.626 |  | GSM814349 | 5.92 | C1 |  |
| GSM814273 | 2.6931507 | 0 | 0.2 | 0.11 | 0.2 | -0 | 0.32 | 0.2 | C1 | TCGA-XF-AAN1 | 17.422 |  | GSM814353 | 5.54 | C1 |  |
| GSM814274 | 3.3260274 | 0 | 0.2 | 0.11 | 0.1 | 0.1 | 0.29 | 0.2 | C1 | TCGA-XF-AAN2 | 35.277 |  | GSM814354 | 5.31 | C1 |  |
| GSM814275 | 3.1835616 | 0 | 0.1 | 0.08 | 0.2 | 0 | 0.27 | 0.1 | C1 | TCGA-YF-AA3L | 12.562 |  | GSM814356 | 5.04 | C1 |  |
| GSM814276 | 3.3808219 | 0 | 0.2 | 0.12 | 0.1 | 0 | 0.3 | 0.2 | C1 | TCGA-ZF-A9R0 | 13.209 |  | GSM814357 | 4.42 | C1 |  |
| GSM814281 | 2.109589 | 0 | 0.1 | 0.13 | 0.1 | 0 | 0.29 | 0.2 | C1 | TCGA-ZF-A9R3 | 19.562 |  | GSM340614 | 4.76 | C2 |  |
| GSM814282 | 2.6273973 | 0 | 0.2 | 0.13 | 0.1 | 0 | 0.35 | 0.1 | C1 | TCGA-ZF-A9R4 | 29.787 |  | GSM340620 | 4.56 | C2 |  |
| GSM814284 | 1.8082192 | 0 | 0.1 | 0.05 | 0.1 | 0 | 0.24 | 0.1 | C1 | TCGA-ZF-A9R5 | 25.903 |  | GSM340623 | 4.39 | C2 |  |
| GSM814287 | 2.8082192 | 0 | 0.3 | 0.25 | 0.3 | 0.2 | 0.4 | 0.3 | C1 | TCGA-ZF-A9R7 | 19.389 |  | GSM340633 | 5.14 | C2 |  |
| GSM814288 | 2.0630137 | 0 | 0.1 | 0.03 | 0 | 0 | 0.25 | 0.2 | C1 | TCGA-ZF-A9RL | 18.27 |  | GSM340634 | 4.68 | C2 |  |
| GSM814289 | 0.6849315 | 1 | 0.2 | 0.1 | 0.2 | 0.1 | 0.31 | 0.2 | C1 | TCGA-ZF-A9RN | 39.24 |  | GSM340636 | 5.19 | C2 |  |
| GSM814290 | 0.9890411 | 0 | 0.1 | 0.09 | 0 | 0 | 0.24 | 0.1 | C1 | TCGA-ZF-AA4X | 21.238 |  | GSM340672 | 4.4 | C2 |  |
| GSM814291 | 1.5205479 | 1 | 0.1 | 0.02 | 0.1 | 0 | 0.26 | 0.1 | C1 | TCGA-ZF-AA51 | 13.612 |  | GSM340684 | 4.74 | C2 |  |
| GSM814295 | 2.8383562 | 0 | 0.2 | 0.1 | 0.1 | 0.1 | 0.31 | 0.2 | C2 | TCGA-4Z-AA82 | 26.987 |  | GSM340694 | 5.63 | C2 |  |
| GSM814297 | 2.6109589 | 0 | 0.1 | 0.1 | 0.1 | 0 | 0.28 | 0.2 | C2 | TCGA-4Z-AA86 | 23.386 |  | GSM340700 | 5.01 | C2 |  |
| GSM814298 | 2.5315068 | 0 | 0.3 | 0.2 | 0.3 | 0.1 | 0.37 | 0.3 | C2 | TCGA-5N-A9KI | 22.231 |  | GSM340702 | 5.85 | C2 |  |
| GSM814300 | 0.8191781 | 1 | 0.1 | 0.06 | 0.1 | 0 | 0.24 | 0.1 | C2 | TCGA-BL-A13I | 38.081 |  | GSM340706 | 5.09 | C2 |  |
| GSM814301 | 2.7780822 | 0 | 0.3 | 0.19 | 0.2 | 0.1 | 0.38 | 0.3 | C2 | TCGA-BL-A13J | 31.1 |  | GSM340712 | 4.71 | C2 |  |
| GSM814303 | 2.5671233 | 0 | 0.2 | 0.15 | 0.2 | 0.1 | 0.29 | 0.2 | C2 | TCGA-BL-A5ZZ | 19.049 |  | GSM340715 | 4.6 | C2 |  |
| GSM814304 | 2.1917808 | 0 | 0.1 | 0.08 | 0.1 | -0 | 0.27 | 0.1 | C2 | TCGA-BT-A0S7 | 21.427 |  | GSM340723 | 4.69 | C2 |  |
| GSM814305 | 2.4931507 | 0 | 0.1 | 0.08 | 0.2 | 0 | 0.32 | 0.2 | C2 | TCGA-BT-A20J | 30.716 |  | GSM340729 | 4.71 | C2 |  |
| GSM814306 | 2.4575342 | 0 | 0.1 | 0.07 | 0.1 | -0 | 0.26 | 0.2 | C2 | TCGA-BT-A20O | 27.025 |  | GSM340763 | 4.77 | C2 |  |
| GSM814307 | 2.2054795 | 0 | 0.1 | 0.08 | 0.1 | 0 | 0.26 | 0.1 | C2 | TCGA-BT-A20Q | 49.691 |  | GSM340764 | 4.37 | C2 |  |
| GSM814308 | 2.3369863 | 0 | 0.3 | 0.25 | 0.3 | 0.1 | 0.41 | 0.3 | C2 | TCGA-BT-A20R | 25.792 |  | GSM340765 | 4.31 | C2 |  |
| GSM814309 | 2.2219178 | 0 | 0.1 | 0.05 | 0.1 | 0 | 0.24 | 0.2 | C2 | TCGA-BT-A20T | 22.872 |  | GSM340767 | 4.42 | C2 |  |
| GSM814312 | 2.1205479 | 0 | 0.3 | 0.2 | 0.2 | 0.1 | 0.36 | 0.2 | C2 | TCGA-BT-A20U | 50.979 |  | GSM786493 | 4.43 | C2 |  |
| GSM814313 | 1.7972603 | 0 | 0.3 | 0.28 | 0.3 | 0.2 | 0.4 | 0.3 | C2 | TCGA-BT-A20X | 30.216 |  | GSM786495 | 5.18 | C2 |  |
| GSM814317 | 1.7506849 | 0 | 0.3 | 0.29 | 0.3 | 0.2 | 0.39 | 0.3 | C2 | TCGA-BT-A2LB | 20.244 |  | GSM786496 | 4.43 | C2 |  |
| GSM814318 | 1.9753425 | 0 | 0.2 | 0.12 | 0.1 | 0 | 0.33 | 0.1 | C2 | TCGA-BT-A3PJ | 25.686 |  | GSM786497 | 5.02 | C2 |  |
| GSM814321 | 1.9835616 | 0 | 0.1 | 0.05 | 0.1 | -0 | 0.24 | 0.2 | C2 | TCGA-BT-A3PK | 33.774 |  | GSM786499 | 4.4 | C2 |  |
| GSM814322 | 1.2575342 | 1 | 0.1 | 0.1 | 0.1 | 0 | 0.29 | 0.1 | C2 | TCGA-C4-A0F0 | 10.671 |  | GSM786508 | 5.07 | C2 |  |
| GSM814323 | 1.7013699 | 0 | 0.1 | 0.09 | 0 | 0 | 0.28 | 0.2 | C2 | TCGA-C4-A0F1 | 32.691 |  | GSM786510 | 5.96 | C2 |  |
| GSM814324 | 1.7123288 | 0 | 0.1 | 0.06 | 0.1 | 0 | 0.25 | 0.2 | C2 | TCGA-C4-A0F7 | 31.095 |  | GSM786515 | 5.56 | C2 |  |
| GSM814325 | 1.1178082 | 1 | 0.2 | 0.09 | 0.1 | 0.1 | 0.25 | 0.2 | C2 | TCGA-CF-A9FL | 13.44 |  | GSM786526 | 4.82 | C2 |  |
| GSM814326 | 1.1369863 | 0 | 0.2 | 0.19 | 0.1 | 0.1 | 0.37 | 0.2 | C2 | TCGA-CU-A0YN | 38.297 |  | GSM786530 | 4.99 | C2 |  |
| GSM814328 | 1.6575342 | 0 | 0.1 | 0.03 | 0.1 | -0 | 0.27 | 0.1 | C2 | TCGA-CU-A0YO | 25.189 |  | GSM786533 | 5.09 | C2 |  |
| GSM814330 | 1.6219178 | 0 | 0.1 | 0.09 | 0.1 | -0 | 0.25 | 0.1 | C2 | TCGA-CU-A0YR | 14.822 |  | GSM786535 | 4.56 | C2 |  |
| GSM814331 | 1.4739726 | 0 | 0.2 | 0.17 | 0.2 | 0.1 | 0.35 | 0.2 | C2 | TCGA-DK-A1A3 | 17.715 |  | GSM786536 | 5.26 | C2 |  |
| GSM814333 | 1.4739726 | 0 | 0.1 | 0.06 | 0.1 | 0 | 0.26 | 0.2 | C2 | TCGA-DK-A1A5 | 27.061 |  | GSM786537 | 5.58 | C2 |  |
| GSM814334 | 0.6461187 | 0 | 0.3 | 0.26 | 0.3 | 0.2 | 0.4 | 0.3 | C2 | TCGA-DK-A1AB | 28.786 |  | GSM786540 | 4.39 | C2 |  |
| GSM814336 | 1.3534247 | 0 | 0.1 | 0.1 | 0.1 | 0 | 0.32 | 0.2 | C2 | TCGA-DK-A1AF | 32.356 |  | GSM786542 | 4.74 | C2 |  |
| GSM814337 | 1.4383562 | 0 | 0.1 | 0.09 | 0.1 | 0 | 0.28 | 0.2 | C2 | TCGA-DK-A2HX | 35.037 |  | GSM786546 | 4.34 | C2 |  |
| GSM814338 | 1.0794521 | 0 | 0.2 | 0.08 | 0.1 | -0 | 0.29 | 0.2 | C2 | TCGA-DK-A2I1 | 11.641 |  | GSM786547 | 4.41 | C2 |  |
| GSM814342 | 0.7150685 | 0 | 0.1 | 0.13 | 0.1 | 0 | 0.32 | 0.2 | C2 | TCGA-DK-A2I2 | 24.61 |  | GSM786551 | 4.64 | C2 |  |
| GSM814345 | 0.5479452 | 0 | 0.1 | 0.07 | 0.1 | -0 | 0.27 | 0.2 | C2 | TCGA-DK-A2I4 | 32.727 |  | GSM786559 | 4.43 | C2 |  |
| GSM814347 | 0.830137 | 0 | 0.1 | 0.11 | 0.2 | 0.1 | 0.28 | 0.1 | C2 | TCGA-DK-A3IN | 22.845 |  | GSM786563 | 4.73 | C2 |  |
| GSM814348 | 0.7643836 | 0 | 0.2 | 0.05 | 0.1 | -0 | 0.26 | 0.2 | C2 | TCGA-DK-A3IQ | 20.645 |  | GSM786568 | 5.01 | C2 |  |
| GSM814349 | 0.0416667 | 0 | 0.2 | 0.12 | 0.2 | 0.1 | 0.31 | 0.1 | C2 | TCGA-DK-A3IT | 26.298 |  | GSM786570 | 4.3 | C2 |  |
| GSM814351 | 0.4657534 | 0 | 0.1 | 0.1 | 0 | -0 | 0.28 | 0.2 | C2 | TCGA-DK-A3IU | 30.615 |  | GSM786571 | 4.78 | C2 |  |
| GSM814353 | 0.9123288 | 1 | 0.3 | 0.18 | 0.2 | 0.1 | 0.35 | 0.2 | C2 | TCGA-DK-A3WX | 51.677 |  | GSM786572 | 4.35 | C2 |  |
| GSM814354 | 1.4219178 | 0 | 0.2 | 0.16 | 0.3 | 0.1 | 0.3 | 0.2 | C2 | TCGA-DK-A6B2 | 19.078 |  | GSM786576 | 4.19 | C2 |  |
| GSM814355 | 0.5643836 | 0 | 0.1 | 0.07 | 0.1 | -0 | 0.25 | 0.2 | C2 | TCGA-DK-AA6M | 19.645 |  | GSM786579 | 4.29 | C2 |  |
| GSM814356 | 1.3643836 | 0 | 0.1 | 0.13 | 0.2 | 0.1 | 0.25 | 0.1 | C2 | TCGA-DK-AA6R | 31.038 |  | GSM786581 | 5.09 | C2 |  |
| GSM814357 | 1.6246575 | 0 | 0.1 | 0.01 | 0.1 | -0 | 0.2 | 0.1 | C2 | TCGA-DK-AA6S | 31.465 |  | GSM786583 | 4.25 | C2 |  |
| GSM340612 | 10.8275 | 0 | 0.2 | 0.14 | 0.1 | 0.1 | 0.34 | 0.2 | C2 | TCGA-DK-AA74 | 21.522 |  | GSM814058 | 4.26 | C2 |  |
| GSM340613 | 1.0475 | 1 | 0.1 | 0.09 | 0 | 0 | 0.31 | 0.1 | C2 | TCGA-E7-A97P | 30.93 |  | GSM814061 | 3.91 | C2 |  |
| GSM340614 | 10.7475 | 0 | 0.1 | 0.06 | 0.1 | -0 | 0.26 | 0.1 | C2 | TCGA-FD-A3B3 | 41.263 |  | GSM814065 | 4.91 | C2 |  |
| GSM340618 | 0.0858333 | 1 | 0.1 | 0.06 | 0.1 | -0 | 0.21 | 0.2 | C2 | TCGA-FD-A3B4 | 28.556 |  | GSM814066 | 4.97 | C2 |  |
| GSM340620 | 1.3891667 | 1 | 0.1 | 0.07 | 0 | 0 | 0.29 | 0.1 | C2 | TCGA-FD-A3B5 | 40.362 |  | GSM814069 | 3.74 | C2 |  |
| GSM340623 | 0.2608333 | 1 | 0.1 | 0.12 | 0.1 | -0 | 0.29 | 0.1 | C2 | TCGA-FD-A3B6 | 34.803 |  | GSM814074 | 4.74 | C2 |  |
| GSM340628 | 10.041667 | 0 | 0.1 | 0.08 | 0.2 | 0 | 0.23 | 0.1 | C2 | TCGA-FD-A3B7 | 30.32 |  | GSM814086 | 4.74 | C2 |  |
| GSM340630 | 5.525 | 1 | 0.1 | 0.06 | 0 | -0 | 0.26 | 0.1 | C2 | TCGA-FD-A3B8 | 46.625 |  | GSM814087 | 5.17 | C2 |  |
| GSM340633 | 1.2141667 | 1 | 0.1 | 0.07 | 0.1 | -0 | 0.25 | 0.1 | C2 | TCGA-FD-A3N5 | 39.11 |  | GSM814091 | 4.35 | C2 |  |
| GSM340634 | 9.6525 | 0 | 0.1 | 0.06 | 0.1 | 0 | 0.23 | 0.2 | C2 | TCGA-FD-A3NA | 22.234 |  | GSM814100 | 4.74 | C2 |  |
| GSM340636 | 10.1 | 0 | 0.1 | 0.1 | 0.1 | 0 | 0.27 | 0.1 | C2 | TCGA-FD-A3SL | 20.151 |  | GSM814110 | 5.51 | C2 |  |
| GSM340648 | 7.5608333 | 0 | 0.1 | 0.11 | 0.1 | -0 | 0.27 | 0.2 | C2 | TCGA-FD-A3SM | 36.404 |  | GSM814122 | 5.18 | C2 |  |
| GSM340653 | 2.1525 | 1 | 0.1 | 0.08 | 0.1 | 0 | 0.26 | 0.2 | C2 | TCGA-FD-A3SN | 13 |  | GSM814135 | 4.42 | C2 |  |
| GSM340654 | 0.8558333 | 1 | 0.2 | 0.14 | 0.1 | 0.1 | 0.29 | 0.1 | C2 | TCGA-FD-A3SO | 40.738 |  | GSM814142 | 4.13 | C2 |  |
| GSM340660 | 6.8025 | 0 | 0.2 | 0.13 | 0.1 | 0.1 | 0.33 | 0.2 | C2 | TCGA-FD-A3SP | 39.317 |  | GSM814149 | 5.07 | C2 |  |
| GSM340665 | 0.725 | 1 | 0.2 | 0.1 | 0.1 | 0 | 0.29 | 0.2 | C2 | TCGA-FD-A3SQ | 34.867 |  | GSM814153 | 4.87 | C2 |  |
| GSM340672 | 1.2583333 | 1 | 0.2 | 0.11 | 0.1 | 0 | 0.3 | 0.2 | C2 | TCGA-FD-A3SR | 22.095 |  | GSM814155 | 5.41 | C2 |  |
| GSM340673 | 0.9358333 | 1 | 0.1 | 0.08 | 0.1 | 0 | 0.25 | 0.2 | C2 | TCGA-FD-A43N | 13.424 |  | GSM814163 | 4.73 | C2 |  |
| GSM340674 | 5.7858333 | 0 | 0.3 | 0.15 | 0.1 | 0.1 | 0.35 | 0.2 | C2 | TCGA-FD-A43Y | 18.942 |  | GSM814167 | 4.42 | C2 |  |
| GSM340677 | 5.4358333 | 0 | 0.2 | 0.06 | 0.2 | 0.1 | 0.28 | 0.1 | C2 | TCGA-FD-A5BR | 27.178 |  | GSM814179 | 4.56 | C2 |  |
| GSM340678 | 0.9975 | 1 | 0.1 | 0.06 | 0.1 | 0 | 0.27 | 0.1 | C2 | TCGA-FD-A5BT | 14.195 |  | GSM814180 | 5.4 | C2 |  |
| GSM340684 | 0.8666667 | 1 | 0.2 | 0.12 | 0.1 | 0.1 | 0.27 | 0.2 | C2 | TCGA-FD-A5BU | 34.711 |  | GSM814185 | 4.95 | C2 |  |
| GSM340693 | 4.7058333 | 0 | 0.2 | 0.1 | 0.2 | 0.1 | 0.29 | 0.2 | C2 | TCGA-FD-A5BX | 15.175 |  | GSM814190 | 4.51 | C2 |  |
| GSM340694 | 4.625 | 0 | 0.2 | 0.12 | 0.2 | 0.1 | 0.29 | 0.2 | C2 | TCGA-FD-A5BY | 32.932 |  | GSM814193 | 5.36 | C2 |  |
| GSM340696 | 0.9583333 | 1 | 0.2 | 0.15 | 0.2 | 0.1 | 0.31 | 0.2 | C2 | TCGA-FD-A5BZ | 18.654 |  | GSM814194 | 4.84 | C2 |  |
| GSM340697 | 1.4891667 | 1 | 0.2 | 0.21 | 0.2 | 0.1 | 0.35 | 0.3 | C2 | TCGA-FD-A5C0 | 19.956 |  | GSM814195 | 4.37 | C2 |  |
| GSM340700 | 1.2833333 | 1 | 0.1 | 0.09 | 0.1 | -0 | 0.27 | 0.2 | C2 | TCGA-FD-A5C1 | 28.712 |  | GSM814196 | 4.2 | C2 |  |
| GSM340701 | 4.0358333 | 0 | 0.1 | -0 | 0 | 0.1 | 0.17 | 0.1 | C2 | TCGA-FD-A62N | 24.199 |  | GSM814201 | 4.59 | C2 |  |
| GSM340702 | 0.375 | 1 | 0.2 | 0.15 | 0.2 | 0.1 | 0.28 | 0.2 | C2 | TCGA-FD-A62P | 20.504 |  | GSM814203 | 4.91 | C2 |  |
| GSM340704 | 0.4358333 | 1 | 0 | -0 | 0.1 | 0.1 | 0.16 | 0.1 | C2 | TCGA-FD-A62S | 24.906 |  | GSM814208 | 4.99 | C2 |  |
| GSM340705 | 3.8 | 0 | 0.3 | 0.26 | 0.2 | 0.2 | 0.43 | 0.3 | C2 | TCGA-FD-A6TA | 30.957 |  | GSM814211 | 5.34 | C2 |  |
| GSM340706 | 3.7608333 | 0 | 0.1 | 0.05 | 0 | 0 | 0.26 | 0.1 | C2 | TCGA-FD-A6TB | 56.726 |  | GSM814212 | 3.95 | C2 |  |
| GSM340709 | 0.4941667 | 1 | 0.2 | 0.11 | 0.2 | 0 | 0.23 | 0.2 | C2 | TCGA-FD-A6TC | 20.789 |  | GSM814213 | 4.74 | C2 |  |
| GSM340712 | 0.5416667 | 1 | 0.1 | 0.05 | 0.1 | 0 | 0.25 | 0.1 | C2 | TCGA-FD-A6TD | 25.217 |  | GSM814221 | 4.19 | C2 |  |
| GSM340714 | 3.3141667 | 0 | 0.2 | 0.12 | 0.1 | 0 | 0.31 | 0.2 | C2 | TCGA-FD-A6TF | 17.539 |  | GSM814229 | 4.94 | C2 |  |
| GSM340715 | 3.2475 | 0 | 0.2 | 0.17 | 0.1 | 0.1 | 0.32 | 0.2 | C2 | TCGA-FD-A6TG | 20.839 |  | GSM814231 | 3.65 | C2 |  |
| GSM340718 | 3.0691667 | 0 | 0.2 | 0.14 | 0.1 | 0 | 0.32 | 0.2 | C2 | TCGA-FD-A6TI | 24.897 |  | GSM814252 | 4.87 | C2 |  |
| GSM340720 | 2.3416667 | 0 | 0.3 | 0.17 | 0.1 | 0.1 | 0.42 | 0.2 | C2 | TCGA-FD-A6TK | 24.171 |  | GSM814253 | 4.96 | C2 |  |
| GSM340721 | 0.3275 | 1 | 0.1 | 0.03 | 0.1 | 0 | 0.24 | 0.1 | C2 | TCGA-FJ-A3Z7 | 11.783 |  | GSM814256 | 4.76 | C2 |  |
| GSM340723 | 1.2583333 | 1 | 0.1 | 0.11 | 0.1 | 0 | 0.28 | 0.2 | C2 | TCGA-FT-A61P | 26.233 |  | GSM814267 | 4.53 | C2 |  |
| GSM340725 | 2.8525 | 0 | 0.1 | 0.12 | 0.1 | 0 | 0.27 | 0.2 | C2 | TCGA-G2-A2EC | 19.661 |  | GSM814273 | 5.09 | C2 |  |
| GSM340727 | 0.9225 | 1 | 0.2 | 0.16 | 0.2 | 0.1 | 0.31 | 0.2 | C2 | TCGA-G2-A2EO | 15.058 |  | GSM814274 | 4.01 | C2 |  |
| GSM340728 | 0.4808333 | 1 | 0.1 | 0.11 | 0.2 | 0.1 | 0.29 | 0.2 | C2 | TCGA-G2-A2ES | 26.825 |  | GSM814288 | 5.63 | C2 |  |
| GSM340729 | 2.7558333 | 0 | 0.1 | 0.03 | 0.1 | 0 | 0.22 | 0.1 | C2 | TCGA-GC-A3I6 | 32.428 |  | GSM814290 | 5.02 | C2 |  |
| GSM340731 | 0.7691667 | 1 | 0.2 | 0.09 | 0.1 | 0 | 0.26 | 0.2 | C2 | TCGA-GC-A3OO | 35.784 |  | GSM814304 | 5.3 | C2 |  |
| GSM340732 | 0.5916667 | 1 | 0.2 | 0.14 | 0.2 | 0.1 | 0.33 | 0.2 | C2 | TCGA-GC-A3RC | 33.362 |  | GSM814305 | 5.38 | C2 |  |
| GSM340733 | 1.4275 | 1 | 0.3 | 0.21 | 0.2 | 0.1 | 0.37 | 0.3 | C2 | TCGA-GC-A3YS | 25.39 |  | GSM814306 | 5.11 | C2 |  |
| GSM340745 | 0.1775 | 1 | 0.1 | 0.12 | 0.2 | 0 | 0.29 | 0.2 | C2 | TCGA-GD-A3OQ | 24.5 |  | GSM814309 | 4.98 | C2 |  |
| GSM340746 | 1.8558333 | 0 | 0.3 | 0.19 | 0.2 | 0.1 | 0.38 | 0.3 | C2 | TCGA-GD-A3OS | 26.32 |  | GSM814321 | 4.61 | C2 |  |
| GSM340747 | 0.5725 | 1 | 0.1 | 0.09 | 0.1 | -0 | 0.25 | 0.2 | C2 | TCGA-GU-A762 | 20.339 |  | GSM814323 | 4.53 | C2 |  |
| GSM340757 | 6.6083333 | 0 | 0.1 | 0.04 | 0.1 | -0 | 0.24 | 0.1 | C2 | TCGA-GU-A764 | 16.553 |  | GSM814324 | 4.35 | C2 |  |
| GSM340758 | 1.2891667 | 1 | 0.2 | 0.17 | 0.2 | 0.1 | 0.32 | 0.2 | C2 | TCGA-GU-A766 | 30.792 |  | GSM814337 | 4.74 | C2 |  |
| GSM340759 | 2.2025 | 1 | 0.3 | 0.17 | 0.1 | 0.1 | 0.37 | 0.2 | C2 | TCGA-GU-AATP | 15.519 |  | GSM340612 | 5.33 | C3 |  |
| GSM340760 | 1.1058333 | 1 | 0.1 | 0.03 | 0 | 0 | 0.24 | 0.1 | C2 | TCGA-GV-A3QG | 21.963 |  | GSM340630 | 3.76 | C3 |  |
| GSM340761 | 0.5333333 | 1 | 0.2 | 0.15 | 0.1 | 0.1 | 0.33 | 0.2 | C2 | TCGA-HQ-A5NE | 39.968 |  | GSM340648 | 4.76 | C3 |  |
| GSM340762 | 0.8891667 | 1 | 0.2 | 0.15 | 0.2 | 0 | 0.29 | 0.3 | C2 | TCGA-K4-A3WS | 22.632 |  | GSM340660 | 5.18 | C3 |  |
| GSM340763 | 1.5108333 | 0 | 0.2 | 0.18 | 0.2 | 0.1 | 0.3 | 0.2 | C2 | TCGA-K4-A3WU | 25.492 |  | GSM340665 | 4.63 | C3 |  |
| GSM340764 | 1.2808333 | 0 | 0.1 | 0.1 | 0.1 | 0 | 0.22 | 0.2 | C2 | TCGA-K4-A4AB | 34.82 |  | GSM340674 | 5.3 | C3 |  |
| GSM340765 | 0.9833333 | 0 | 0.2 | 0.1 | 0.1 | 0 | 0.26 | 0.2 | C2 | TCGA-K4-A54R | 32.674 |  | GSM340697 | 5.94 | C3 |  |
| GSM340766 | 0.9058333 | 0 | 0.3 | 0.19 | 0.3 | 0.1 | 0.37 | 0.3 | C2 | TCGA-K4-A5RH | 20.138 |  | GSM340705 | 4.98 | C3 |  |
| GSM340767 | 0.8358333 | 0 | 0.1 | 0.08 | 0.1 | 0 | 0.28 | 0.2 | C2 | TCGA-K4-A6FZ | 40.81 |  | GSM340714 | 5.68 | C3 |  |
| GSM340769 | 0.4416667 | 0 | 0.3 | 0.21 | 0.3 | 0.2 | 0.35 | 0.3 | C2 | TCGA-K4-AAQO | 19.022 |  | GSM340718 | 5.59 | C3 |  |
| GSM786491 | 8.6488667 | 0 | 0.1 | 0.03 | 0.1 | 0.1 | 0.26 | 0.2 | C2 | TCGA-KQ-A41S | 24.061 |  | GSM340720 | 5.52 | C3 |  |
| GSM786492 | 1.0978783 | 1 | 0.3 | 0.22 | 0.2 | 0.1 | 0.38 | 0.2 | C2 | TCGA-LC-A66R | 21.614 |  | GSM340727 | 5.2 | C3 |  |
| GSM786493 | 1.6481858 | 0 | 0.1 | 0.1 | 0.1 | 0 | 0.25 | 0.1 | C2 | TCGA-R3-A69X | 42.727 |  | GSM340733 | 5.25 | C3 |  |
| GSM786494 | 1.368925 | 1 | 0.2 | 0.09 | 0.2 | 0.1 | 0.35 | 0.2 | C2 | TCGA-SY-A9G0 | 28.472 |  | GSM340746 | 5.81 | C3 |  |
| GSM786495 | 1.09514 | 0 | 0.1 | 0.05 | 0.1 | -0 | 0.25 | 0.2 | C2 | TCGA-SY-A9G5 | 34.502 |  | GSM340747 | 4.83 | C3 |  |
| GSM786496 | 0.3696098 | 1 | 0.1 | 0.02 | 0.1 | -0 | 0.23 | 0.1 | C2 | TCGA-UY-A78P | 27.333 |  | GSM340758 | 4.87 | C3 |  |
| GSM786497 | 7.3210133 | 0 | 0.3 | 0.3 | 0.3 | 0.2 | 0.41 | 0.4 | C2 | TCGA-UY-A8OC | 21.167 |  | GSM340759 | 5.48 | C3 |  |
| GSM786499 | 9.0376417 | 1 | 0.1 | 0.04 | 0.1 | -0 | 0.25 | 0.1 | C2 | TCGA-UY-A8OD | 14.998 |  | GSM340761 | 5.19 | C3 |  |
| GSM786500 | 14.6256 | 0 | 0.3 | 0.21 | 0.3 | 0.1 | 0.36 | 0.3 | C2 | TCGA-UY-A9PB | 30.315 |  | GSM340762 | 4.45 | C3 |  |
| GSM786503 | 0.0328542 | 0 | 0.2 | 0.08 | 0.2 | 0 | 0.26 | 0.2 | C2 | TCGA-UY-A9PF | 11.523 |  | GSM340769 | 4.89 | C3 |  |
| GSM786504 | 0.9034908 | 1 | 0.2 | 0.12 | 0.2 | 0.1 | 0.32 | 0.2 | C2 | TCGA-XF-A8HE | 48.736 |  | GSM786492 | 5.44 | C3 |  |
| GSM786506 | 0.2792608 | 1 | 0.1 | 0.02 | 0.1 | 0 | 0.22 | 0.1 | C2 | TCGA-XF-A8HH | 16.696 |  | GSM786516 | 5.59 | C3 |  |
| GSM786508 | 0.9774125 | 1 | 0.2 | 0.09 | 0.1 | 0 | 0.29 | 0.2 | C2 | TCGA-XF-A9SJ | 21.987 |  | GSM786518 | 5.97 | C3 |  |
| GSM786509 | 2.6091717 | 0 | 0.2 | 0.16 | 0.2 | 0.1 | 0.36 | 0.2 | C2 | TCGA-XF-A9SK | 28.047 |  | GSM786523 | 5.2 | C3 |  |
| GSM786510 | 0.6844627 | 1 | 0.1 | 0.05 | 0.1 | -0 | 0.25 | 0.1 | C2 | TCGA-XF-A9SL | 22.091 |  | GSM786531 | 4.16 | C3 |  |
| GSM786511 | 2.0561258 | 0 | 0.3 | 0.3 | 0.3 | 0.2 | 0.45 | 0.3 | C2 | TCGA-XF-A9SM | 28.496 |  | GSM786539 | 4.83 | C3 |  |
| GSM786512 | 1.1088292 | 1 | 0.1 | 0.08 | 0.1 | 0 | 0.26 | 0.2 | C2 | TCGA-XF-A9SP | 16.439 |  | GSM786541 | 4.95 | C3 |  |
| GSM786513 | 0.0903491 | 0 | 0.2 | 0.09 | 0.2 | 0.1 | 0.3 | 0.2 | C2 | TCGA-XF-A9SU | 13.005 |  | GSM786544 | 4.95 | C3 |  |
| GSM786514 | 0.6105408 | 1 | 0.2 | 0.15 | 0.2 | 0.1 | 0.31 | 0.2 | C2 | TCGA-XF-A9SV | 13.34 |  | GSM786545 | 4.94 | C3 |  |
| GSM786515 | 0.2737851 | 0 | 0.1 | 0.07 | 0.1 | -0 | 0.23 | 0.1 | C2 | TCGA-XF-A9SW | 28.17 |  | GSM786549 | 5.35 | C3 |  |
| GSM786516 | 1.2019167 | 1 | 0.2 | 0.17 | 0.1 | 0.1 | 0.35 | 0.3 | C2 | TCGA-XF-A9SX | 19.512 |  | GSM786552 | 5.12 | C3 |  |
| GSM786518 | 4.859685 | 1 | 0.4 | 0.27 | 0.3 | 0.2 | 0.45 | 0.3 | C2 | TCGA-XF-A9SY | 28.309 |  | GSM786554 | 4.67 | C3 |  |
| GSM786519 | 0.4134155 | 1 | 0.2 | 0.17 | 0.2 | 0.1 | 0.37 | 0.3 | C2 | TCGA-XF-A9SZ | 15.705 |  | GSM786556 | 4.82 | C3 |  |
| GSM786520 | 0.7748118 | 1 | 0.2 | 0.13 | 0.2 | 0.1 | 0.31 | 0.2 | C2 | TCGA-XF-A9T3 | 28.696 |  | GSM786558 | 4.43 | C3 |  |
| GSM786521 | 1.0540725 | 1 | 0 | -0.1 | -0 | -0 | 0.13 | 0 | C2 | TCGA-XF-A9T4 | 26.929 |  | GSM786561 | 5.21 | C3 |  |
| GSM786522 | 6.0451742 | 0 | 0.2 | 0.16 | 0.2 | 0.1 | 0.32 | 0.3 | C2 | TCGA-XF-A9T8 | 42.953 |  | GSM786565 | 4.25 | C3 |  |
| GSM786523 | 7.509925 | 0 | 0.3 | 0.18 | 0.2 | 0.1 | 0.34 | 0.2 | C2 | TCGA-XF-AAME | 29.62 |  | GSM786573 | 5.29 | C3 |  |
| GSM786525 | 0.4435318 | 0 | 0.1 | 0.03 | 0.1 | 0 | 0.2 | 0.2 | C2 | TCGA-XF-AAMJ | 16.381 |  | GSM786577 | 5.02 | C3 |  |
| GSM786526 | 0.2819987 | 0 | 0.2 | 0.09 | 0.2 | 0.1 | 0.26 | 0.2 | C2 | TCGA-XF-AAMR | 44.605 |  | GSM814068 | 5.39 | C3 |  |
| GSM786528 | 0.2354552 | 0 | 0.2 | 0.09 | 0.1 | 0 | 0.27 | 0.2 | C2 | TCGA-XF-AAMT | 36.499 |  | GSM814084 | 4.87 | C3 |  |
| GSM786530 | 0.8268309 | 1 | 0.3 | 0.23 | 0.3 | 0.1 | 0.41 | 0.3 | C2 | TCGA-XF-AAMW | 34.793 |  | GSM814095 | 4.86 | C3 |  |
| GSM786531 | 8.55305 | 0 | 0.2 | 0.15 | 0.1 | 0 | 0.32 | 0.2 | C2 | TCGA-XF-AAMY | 19.685 |  | GSM814098 | 4.84 | C3 |  |
| GSM786532 | 0.2765229 | 1 | 0.2 | 0.12 | 0.2 | 0.1 | 0.27 | 0.2 | C2 | TCGA-XF-AAN3 | 40.171 |  | GSM814102 | 5.57 | C3 |  |
| GSM786533 | 9.177275 | 0 | 0.2 | 0.1 | 0.1 | 0.1 | 0.33 | 0.2 | C2 | TCGA-XF-AAN4 | 44.471 |  | GSM814109 | 5.29 | C3 |  |
| GSM786535 | 0.4654347 | 0 | 0.1 | 0.06 | 0.1 | 0 | 0.27 | 0.2 | C2 | TCGA-XF-AAN5 | 29.947 |  | GSM814112 | 3.92 | C3 |  |
| GSM786536 | 0.6379193 | 1 | 0.2 | 0.12 | 0.1 | 0.1 | 0.32 | 0.2 | C2 | TCGA-XF-AAN7 | 22.518 |  | GSM814118 | 5.31 | C3 |  |
| GSM786537 | 1.4182067 | 1 | 0.3 | 0.31 | 0.3 | 0.2 | 0.42 | 0.3 | C2 | TCGA-XF-AAN8 | 39.797 |  | GSM814133 | 5.99 | C3 |  |
| GSM786539 | 10.064342 | 0 | 0.1 | 0.15 | 0.1 | 0 | 0.28 | 0.2 | C2 | TCGA-YC-A8S6 | 25.459 |  | GSM814134 | 4.87 | C3 |  |
| GSM786540 | 3.7234767 | 0 | 0.1 | 0.09 | 0.1 | 0 | 0.24 | 0.2 | C2 | TCGA-ZF-A9R9 | 13.822 |  | GSM814150 | 4.92 | C3 |  |
| GSM786541 | 7.6386033 | 0 | 0.1 | 0.12 | 0.1 | -0 | 0.27 | 0.2 | C2 | TCGA-ZF-A9RD | 28.349 |  | GSM814156 | 5.78 | C3 |  |
| GSM786542 | 0.3696098 | 1 | 0.2 | 0.09 | 0.1 | 0.1 | 0.28 | 0.2 | C2 | TCGA-ZF-AA4N | 18.843 |  | GSM814160 | 4.6 | C3 |  |
| GSM786543 | 6.6201233 | 0 | 0.3 | 0.21 | 0.2 | 0.1 | 0.42 | 0.3 | C2 | TCGA-ZF-AA4R | 17.893 |  | GSM814162 | 5.11 | C3 |  |
| GSM786544 | 1.1389458 | 1 | 0.2 | 0.18 | 0.1 | 0.1 | 0.34 | 0.2 | C2 | TCGA-ZF-AA52 | 18.06 |  | GSM814174 | 4.44 | C3 |  |
| GSM786545 | 5.4401092 | 0 | 0.3 | 0.17 | 0.2 | 0.1 | 0.4 | 0.2 | C2 | TCGA-ZF-AA53 | 22.659 |  | GSM814176 | 5.22 | C3 |  |
| GSM786546 | 0.0657084 | 0 | 0.1 | 0.09 | 0.1 | 0 | 0.25 | 0.2 | C2 | TCGA-ZF-AA54 | 18.029 |  | GSM814189 | 4.67 | C3 |  |
| GSM786547 | 1.1225192 | 1 | 0.2 | 0.11 | 0.1 | 0 | 0.28 | 0.2 | C2 | TCGA-ZF-AA56 | 40.685 |  | GSM814191 | 4.54 | C3 |  |
| GSM786549 | 9.2867917 | 1 | 0.2 | 0.17 | 0.2 | 0.1 | 0.32 | 0.2 | C2 | TCGA-ZF-AA58 | 22.511 |  | GSM814192 | 4.36 | C3 |  |
| GSM786550 | 0.8186174 | 1 | 0.1 | 0.02 | -0 | -0 | 0.18 | 0.1 | C2 | TCGA-ZF-AA5H | 24.22 |  | GSM814200 | 6.17 | C3 |  |
| GSM786551 | 8.5475667 | 0 | 0.2 | 0.16 | 0.3 | 0.1 | 0.29 | 0.2 | C2 | TCGA-ZF-AA5P | 29.992 |  | GSM814205 | 5.08 | C3 |  |
| GSM786552 | 0.6488707 | 0 | 0.2 | 0.11 | 0.1 | 0 | 0.26 | 0.2 | C3 | TCGA-2F-A9KP | 17.33 |  | GSM814210 | 4.95 | C3 |  |
| GSM786553 | 8.490075 | 0 | 0.1 | 0.08 | 0.2 | 0 | 0.25 | 0.2 | C3 | TCGA-2F-A9KQ | 15.902 |  | GSM814215 | 4.73 | C3 |  |
| GSM786554 | 2.7186858 | 0 | 0.1 | 0.08 | 0.1 | -0 | 0.27 | 0.2 | C3 | TCGA-2F-A9KR | 44.171 |  | GSM814217 | 5.22 | C3 |  |
| GSM786556 | 6.63655 | 0 | 0.1 | 0.17 | 0.1 | 0 | 0.3 | 0.2 | C3 | TCGA-2F-A9KT | 15.48 |  | GSM814224 | 4.4 | C3 |  |
| GSM786557 | 8.65435 | 0 | 0.2 | 0.11 | 0.2 | 0.1 | 0.25 | 0.2 | C3 | TCGA-2F-A9KW | 10.354 |  | GSM814226 | 5.43 | C3 |  |
| GSM786558 | 8.41615 | 0 | 0.2 | 0.11 | 0.1 | 0 | 0.27 | 0.2 | C3 | TCGA-4Z-AA7M | 15.341 |  | GSM814234 | 5.1 | C3 |  |
| GSM786559 | 7.6221767 | 0 | 0.1 | 0.11 | 0.2 | 0 | 0.29 | 0.2 | C3 | TCGA-4Z-AA7R | 19.214 |  | GSM814250 | 5.38 | C3 |  |
| GSM786560 | 8.1806983 | 0 | 0.3 | 0.16 | 0.2 | 0.1 | 0.35 | 0.2 | C3 | TCGA-4Z-AA7S | 10.741 |  | GSM814261 | 5 | C3 |  |
| GSM786561 | 1.2566733 | 0 | 0.3 | 0.22 | 0.3 | 0.1 | 0.38 | 0.3 | C3 | TCGA-4Z-AA7Y | 24.202 |  | GSM814263 | 5 | C3 |  |
| GSM786562 | 0.5530458 | 0 | 0.1 | 0.07 | 0.1 | 0 | 0.27 | 0.2 | C3 | TCGA-4Z-AA80 | 21.916 |  | GSM814266 | 5.41 | C3 |  |
| GSM786563 | 1.0595483 | 1 | 0.1 | 0.07 | 0.1 | 0 | 0.27 | 0.2 | C3 | TCGA-4Z-AA84 | 14.133 |  | GSM814276 | 4.6 | C3 |  |
| GSM786564 | 5.6043808 | 0 | 0 | 0.01 | 0.1 | -0 | 0.19 | 0.2 | C3 | TCGA-BL-A0C8 | 22.412 |  | GSM814281 | 4.55 | C3 |  |
| GSM786565 | 3.572895 | 0 | 0.1 | 0.05 | 0 | -0 | 0.21 | 0.2 | C3 | TCGA-BL-A3JM | 27.2 |  | GSM814297 | 4.95 | C3 |  |
| GSM786568 | 7.0609175 | 0 | 0.2 | 0.13 | 0.2 | 0 | 0.29 | 0.2 | C3 | TCGA-BT-A0YX | 40.993 |  | GSM814301 | 4.7 | C3 |  |
| GSM786570 | 1.4674883 | 0 | 0.1 | 0.02 | 0 | 0 | 0.19 | 0 | C3 | TCGA-BT-A20N | 14.955 |  | GSM814308 | 5.88 | C3 |  |
| GSM786571 | 7.1622175 | 0 | 0.2 | 0.12 | 0.1 | 0.1 | 0.35 | 0.2 | C3 | TCGA-BT-A20P | 25.133 |  | GSM814312 | 4.86 | C3 |  |
| GSM786572 | 7.4250517 | 0 | 0.1 | 0.08 | 0.1 | 0 | 0.26 | 0.1 | C3 | TCGA-BT-A20V | 46.921 |  | GSM814318 | 5.66 | C3 |  |
| GSM786573 | 6.7597533 | 0 | 0.2 | 0.22 | 0.2 | 0.1 | 0.34 | 0.3 | C3 | TCGA-BT-A2LA | 7.6185 |  | GSM814326 | 5.25 | C3 |  |
| GSM786576 | 0.9719367 | 1 | 0.1 | 0.02 | 0 | -0 | 0.21 | 0.1 | C3 | TCGA-BT-A2LD | 22.605 |  | GSM814336 | 5.25 | C3 |  |
| GSM786577 | 2.7378508 | 1 | 0.3 | 0.29 | 0.3 | 0.2 | 0.43 | 0.3 | C3 | TCGA-BT-A3PH | 16.489 |  | GSM814338 | 4.87 | C3 |  |
| GSM786579 | 4.29295 | 1 | 0.1 | 0.11 | 0.1 | 0.1 | 0.28 | 0.2 | C3 | TCGA-BT-A42C | 23.343 |  | GSM814342 | 5.37 | C3 |  |
| GSM786580 | 3.8521558 | 0 | 0.1 | -0 | 0 | 0 | 0.18 | 0.1 | C3 | TCGA-C4-A0EZ | 10.594 |  | GSM814345 | 4.54 | C3 |  |
| GSM786581 | 5.98768 | 0 | 0.1 | 0.08 | 0.1 | 0 | 0.29 | 0.2 | C3 | TCGA-C4-A0F6 | 16.647 |  | GSM814348 | 4.52 | C3 |  |
| GSM786582 | 0.476386 | 1 | 0.2 | 0.24 | 0.3 | 0.1 | 0.38 | 0.3 | C3 | TCGA-CF-A1HS | 20.079 |  | GSM814351 | 4.55 | C3 |  |
| GSM786583 | 5.3798767 | 0 | 0.2 | 0.16 | 0.2 | 0.1 | 0.33 | 0.2 | C3 | TCGA-CF-A3MG | 15.471 |  | GSM814355 | 5.07 | C3 |  |
|  |  |  |  |  |  |  |  |  | C3 | TCGA-CF-A47T | 6.4464 |  |  |  |  |  |
|  |  |  |  |  |  |  |  |  | C3 | TCGA-CF-A5UA | 8.6947 |  |  |  |  |  |
| TCGA-6 commom dif with survival | | | | |  |  |  |  | C3 | TCGA-CF-A8HY | 8.3923 |  |  |  |  |  |
| id | futime | fustat | N_GSE21670_STAT3_KO_VS_WT_CD4_TCELL_TGFB_IL6_TREATED_DN | N_GSE1460_INTRATHYMIC_T_PROGENITOR_VS_CD4_THYMOCYTE_DN | N_GSE17974_IL4_AND_ANTI_IL12_VS_UNTREATED_72H_ACT_CD4_TCELL_DN | N_GSE39556_UNTREATED_VS_3H_POLYIC_INJ_MOUSE_CD8A_DC_UP | N_GSE42088_2H_VS_24H_LEISHMANIA_INF_DC_UP | N_GSE29618_BCELL_VS_PDC_UP | C3 | TCGA-CU-A3KJ | 37.774 |  |  |  |  |  |
| TCGA-FD-A6TF | 0.1890411 | 1 | 0.7 | 0.7 | 0.7 | 0.7 | 0.86 | 0.7 | C3 | TCGA-CU-A3QU | 17.529 |  |  |  |  |  |
| TCGA-XF-AAMR | 7.6438356 | 0 | 0.8 | 0.73 | 0.7 | 0.7 | 0.86 | 0.7 | C3 | TCGA-CU-A5W6 | 23.785 |  |  |  |  |  |
| TCGA-XF-A8HF | 8.0931507 | 1 | 0.7 | 0.68 | 0.6 | 0.6 | 0.84 | 0.7 | C3 | TCGA-CU-A72E | 32.771 |  |  |  |  |  |
| TCGA-ZF-AA4U | 0.7178082 | 1 | 0.7 | 0.67 | 0.6 | 0.6 | 0.81 | 0.7 | C3 | TCGA-DK-A1AE | 25.927 |  |  |  |  |  |
| TCGA-ZF-AA54 | 1.6164384 | 1 | 0.8 | 0.73 | 0.7 | 0.7 | 0.88 | 0.8 | C3 | TCGA-DK-A2I6 | 14.527 |  |  |  |  |  |
| TCGA-GU-A766 | 1.3150685 | 0 | 0.8 | 0.75 | 0.7 | 0.7 | 0.88 | 0.8 | C3 | TCGA-DK-A3IL | 12.481 |  |  |  |  |  |
| TCGA-BL-A13I | 0.6109589 | 1 | 0.8 | 0.73 | 0.7 | 0.6 | 0.86 | 0.7 | C3 | TCGA-DK-A3IM | 22.508 |  |  |  |  |  |
| TCGA-XF-AAML | 0.6356164 | 1 | 0.7 | 0.68 | 0.7 | 0.6 | 0.82 | 0.7 | C3 | TCGA-DK-A3IS | 18.604 |  |  |  |  |  |
| TCGA-ZF-AA58 | 4.5178082 | 0 | 0.8 | 0.75 | 0.7 | 0.7 | 0.88 | 0.8 | C3 | TCGA-DK-A3X1 | 11.036 |  |  |  |  |  |
| TCGA-G2-AA3B | 5.5013699 | 0 | 0.7 | 0.66 | 0.6 | 0.6 | 0.81 | 0.7 | C3 | TCGA-DK-A3X2 | 30.07 |  |  |  |  |  |
| TCGA-K4-A54R | 2.3068493 | 0 | 0.8 | 0.72 | 0.7 | 0.7 | 0.86 | 0.7 | C3 | TCGA-DK-A6AV | 8.5691 |  |  |  |  |  |
| TCGA-DK-A1AA | 1.5835616 | 0 | 0.7 | 0.69 | 0.7 | 0.6 | 0.84 | 0.7 | C3 | TCGA-DK-A6B1 | 17.204 |  |  |  |  |  |
| TCGA-CU-A5W6 | 0.1534247 | 1 | 0.7 | 0.68 | 0.7 | 0.6 | 0.83 | 0.7 | C3 | TCGA-DK-A6B5 | 42.386 |  |  |  |  |  |
| TCGA-DK-AA6R | 13.810959 | 0 | 0.7 | 0.69 | 0.7 | 0.6 | 0.82 | 0.7 | C3 | TCGA-DK-AA6P | 26.066 |  |  |  |  |  |
| TCGA-BT-A3PH | 0.3890411 | 1 | 0.7 | 0.67 | 0.6 | 0.6 | 0.82 | 0.7 | C3 | TCGA-DK-AA6Q | 23.04 |  |  |  |  |  |
| TCGA-DK-A3IQ | 1.4767123 | 1 | 0.8 | 0.72 | 0.6 | 0.6 | 0.86 | 0.7 | C3 | TCGA-DK-AA6U | 21.252 |  |  |  |  |  |
| TCGA-E7-A4XJ | 0.1863014 | 1 | 0.7 | 0.64 | 0.6 | 0.6 | 0.8 | 0.7 | C3 | TCGA-DK-AA6W | 16.947 |  |  |  |  |  |
| TCGA-XF-A9T6 | 0.1753425 | 0 | 0.7 | 0.67 | 0.7 | 0.6 | 0.83 | 0.7 | C3 | TCGA-DK-AA71 | 24.832 |  |  |  |  |  |
| TCGA-ZF-AA5H | 2.4575342 | 0 | 0.8 | 0.72 | 0.7 | 0.7 | 0.84 | 0.8 | C3 | TCGA-DK-AA75 | 10.84 |  |  |  |  |  |
| TCGA-DK-A3WX | 0.8794521 | 1 | 0.8 | 0.71 | 0.7 | 0.7 | 0.85 | 0.8 | C3 | TCGA-DK-AA76 | 14.066 |  |  |  |  |  |
| TCGA-YF-AA3L | 0.9972603 | 0 | 0.7 | 0.68 | 0.6 | 0.6 | 0.83 | 0.7 | C3 | TCGA-E5-A2PC | 37.066 |  |  |  |  |  |
| TCGA-BT-A2LA | 1.430137 | 0 | 0.6 | 0.63 | 0.6 | 0.6 | 0.75 | 0.6 | C3 | TCGA-E5-A4TZ | 43.352 |  |  |  |  |  |
| TCGA-4Z-AA87 | 3.9835616 | 0 | 0.7 | 0.66 | 0.6 | 0.6 | 0.82 | 0.7 | C3 | TCGA-E7-A4XJ | 6.372 |  |  |  |  |  |
| TCGA-BT-A2LB | 1.3479452 | 1 | 0.8 | 0.73 | 0.7 | 0.7 | 0.87 | 0.8 | C3 | TCGA-E7-A5KE | 11.244 |  |  |  |  |  |
| TCGA-XF-A8HB | 3.7534247 | 0 | 0.7 | 0.66 | 0.6 | 0.6 | 0.83 | 0.7 | C3 | TCGA-E7-A6ME | 18.769 |  |  |  |  |  |
| TCGA-DK-A2I6 | 7.2767123 | 0 | 0.7 | 0.67 | 0.6 | 0.6 | 0.81 | 0.7 | C3 | TCGA-E7-A7DV | 20.573 |  |  |  |  |  |
| TCGA-ZF-AA4X | 5.6 | 0 | 0.7 | 0.65 | 0.6 | 0.6 | 0.8 | 0.7 | C3 | TCGA-FD-A3N6 | 25.41 |  |  |  |  |  |
| TCGA-BT-A20P | 1.490411 | 1 | 0.7 | 0.67 | 0.6 | 0.6 | 0.81 | 0.7 | C3 | TCGA-FD-A3SJ | 34.217 |  |  |  |  |  |
| TCGA-CF-A3MF | 1.0493151 | 0 | 0.7 | 0.64 | 0.6 | 0.6 | 0.79 | 0.6 | C3 | TCGA-FD-A3SS | 13.583 |  |  |  |  |  |
| TCGA-CF-A47T | 1.0547945 | 1 | 0.7 | 0.64 | 0.6 | 0.6 | 0.8 | 0.6 | C3 | TCGA-FD-A5BV | 12.151 |  |  |  |  |  |
| TCGA-YC-A8S6 | 0.8027397 | 0 | 0.8 | 0.76 | 0.7 | 0.7 | 0.88 | 0.8 | C3 | TCGA-FD-A62O | 13.312 |  |  |  |  |  |
| TCGA-DK-A3IS | 4.1890411 | 0 | 0.7 | 0.66 | 0.6 | 0.6 | 0.81 | 0.7 | C3 | TCGA-FD-A6TH | 16.077 |  |  |  |  |  |
| TCGA-XF-A9SJ | 0.2684932 | 1 | 0.8 | 0.71 | 0.7 | 0.7 | 0.86 | 0.8 | C3 | TCGA-FJ-A3Z9 | 8.718 |  |  |  |  |  |
| TCGA-DK-AA6T | 1.5671233 | 0 | 0.8 | 0.75 | 0.7 | 0.7 | 0.87 | 0.8 | C3 | TCGA-FJ-A3ZE | 14.918 |  |  |  |  |  |
| TCGA-E7-A5KE | 0.0465753 | 0 | 0.6 | 0.61 | 0.6 | 0.6 | 0.77 | 0.6 | C3 | TCGA-FJ-A871 | 11.547 |  |  |  |  |  |
| TCGA-ZF-AA4V | 4.9479452 | 0 | 0.7 | 0.7 | 0.7 | 0.6 | 0.85 | 0.7 | C3 | TCGA-FT-A3EE | 23.132 |  |  |  |  |  |
| TCGA-ZF-AA53 | 4.8246575 | 0 | 0.8 | 0.73 | 0.7 | 0.6 | 0.87 | 0.8 | C3 | TCGA-G2-A2EJ | 32.959 |  |  |  |  |  |
| TCGA-CU-A72E | 1.1315068 | 1 | 0.7 | 0.69 | 0.6 | 0.6 | 0.85 | 0.7 | C3 | TCGA-G2-A2EL | 22.751 |  |  |  |  |  |
| TCGA-FD-A62O | 0.5917808 | 1 | 0.7 | 0.68 | 0.6 | 0.6 | 0.82 | 0.7 | C3 | TCGA-G2-A3IB | 28.285 |  |  |  |  |  |
| TCGA-BL-A3JM | 0.5616438 | 1 | 0.7 | 0.68 | 0.7 | 0.6 | 0.82 | 0.7 | C3 | TCGA-G2-A3VY | 12.152 |  |  |  |  |  |
| TCGA-FJ-A3ZF | 1.4356164 | 0 | 0.6 | 0.61 | 0.6 | 0.5 | 0.76 | 0.6 | C3 | TCGA-G2-AA3C | 22.227 |  |  |  |  |  |
| TCGA-CU-A0YO | 0.4082192 | 1 | 0.8 | 0.75 | 0.7 | 0.7 | 0.87 | 0.8 | C3 | TCGA-G2-AA3F | 13.281 |  |  |  |  |  |
| TCGA-CF-A5UA | 1 | 0 | 0.7 | 0.67 | 0.6 | 0.6 | 0.83 | 0.7 | C3 | TCGA-GC-A3RB | 25.845 |  |  |  |  |  |
| TCGA-K4-AAQO | 0.9835616 | 0 | 0.8 | 0.74 | 0.7 | 0.7 | 0.87 | 0.8 | C3 | TCGA-GC-A3RD | 20.848 |  |  |  |  |  |
| TCGA-ZF-A9R7 | 1.8219178 | 0 | 0.7 | 0.72 | 0.7 | 0.6 | 0.85 | 0.8 | C3 | TCGA-GC-A4ZW | 27.699 |  |  |  |  |  |
| TCGA-FD-A3SO | 0.460274 | 1 | 0.8 | 0.72 | 0.7 | 0.7 | 0.85 | 0.8 | C3 | TCGA-GD-A2C5 | 15.767 |  |  |  |  |  |
| TCGA-E7-A97P | 1.1972603 | 1 | 0.8 | 0.75 | 0.7 | 0.7 | 0.86 | 0.8 | C3 | TCGA-GD-A6C6 | 7.7459 |  |  |  |  |  |
| TCGA-GU-A767 | 0.3945205 | 1 | 0.7 | 0.69 | 0.6 | 0.6 | 0.84 | 0.7 | C3 | TCGA-GU-A42P | 39.195 |  |  |  |  |  |
| TCGA-4Z-AA81 | 3.4794521 | 1 | 0.7 | 0.69 | 0.7 | 0.6 | 0.83 | 0.7 | C3 | TCGA-GU-A42Q | 29.455 |  |  |  |  |  |
| TCGA-2F-A9KQ | 7.9068493 | 0 | 0.7 | 0.68 | 0.6 | 0.6 | 0.82 | 0.7 | C3 | TCGA-GU-A42R | 25.273 |  |  |  |  |  |
| TCGA-SY-A9G0 | 2.7616438 | 1 | 0.8 | 0.76 | 0.7 | 0.7 | 0.87 | 0.8 | C3 | TCGA-GU-A767 | 11.512 |  |  |  |  |  |
| TCGA-GV-A40G | 1.5890411 | 0 | 0.7 | 0.64 | 0.6 | 0.6 | 0.8 | 0.6 | C3 | TCGA-GU-AATO | 33.46 |  |  |  |  |  |
| TCGA-XF-A9SL | 5.5342466 | 1 | 0.8 | 0.77 | 0.7 | 0.7 | 0.9 | 0.8 | C3 | TCGA-GU-AATQ | 28.414 |  |  |  |  |  |
| TCGA-XF-AAN2 | 5.1205479 | 1 | 0.7 | 0.7 | 0.7 | 0.6 | 0.85 | 0.7 | C3 | TCGA-GV-A3JW | 10.819 |  |  |  |  |  |
| TCGA-FD-A6TD | 1.0575342 | 1 | 0.8 | 0.74 | 0.7 | 0.7 | 0.87 | 0.8 | C3 | TCGA-GV-A3JX | 36.732 |  |  |  |  |  |
| TCGA-G2-AA3F | 2.4465753 | 0 | 0.7 | 0.66 | 0.6 | 0.6 | 0.8 | 0.7 | C3 | TCGA-GV-A3JZ | 27.829 |  |  |  |  |  |
| TCGA-FJ-A871 | 0.7452055 | 1 | 0.7 | 0.65 | 0.6 | 0.6 | 0.8 | 0.7 | C3 | TCGA-GV-A3QF | 18.802 |  |  |  |  |  |
| TCGA-ZF-A9RN | 1.6849315 | 1 | 0.7 | 0.7 | 0.7 | 0.6 | 0.84 | 0.7 | C3 | TCGA-GV-A3QH | 19.549 |  |  |  |  |  |
| TCGA-GC-A3RB | 1.5945205 | 0 | 0.7 | 0.69 | 0.7 | 0.6 | 0.82 | 0.7 | C3 | TCGA-GV-A3QI | 15.536 |  |  |  |  |  |
| TCGA-UY-A9PF | 0.3205479 | 0 | 0.8 | 0.72 | 0.7 | 0.7 | 0.85 | 0.8 | C3 | TCGA-GV-A40E | 21.905 |  |  |  |  |  |
| TCGA-YC-A89H | 1.569863 | 0 | 0.7 | 0.68 | 0.6 | 0.6 | 0.84 | 0.7 | C3 | TCGA-GV-A40G | 22.114 |  |  |  |  |  |
| TCGA-XF-AAMQ | 5.9643836 | 0 | 0.8 | 0.74 | 0.7 | 0.7 | 0.86 | 0.8 | C3 | TCGA-H4-A2HQ | 40.694 |  |  |  |  |  |
| TCGA-CF-A1HR | 1.0657534 | 0 | 0.7 | 0.7 | 0.7 | 0.6 | 0.85 | 0.7 | C3 | TCGA-HQ-A2OE | 30.751 |  |  |  |  |  |
| TCGA-E7-A3X6 | 2.4767123 | 1 | 0.8 | 0.72 | 0.7 | 0.6 | 0.84 | 0.7 | C3 | TCGA-HQ-A2OF | 16.665 |  |  |  |  |  |
| TCGA-E7-A97Q | 0.6739726 | 1 | 0.7 | 0.69 | 0.6 | 0.6 | 0.83 | 0.7 | C3 | TCGA-HQ-A5ND | 30.174 |  |  |  |  |  |
| TCGA-E7-A7DV | 0.1013699 | 0 | 0.8 | 0.71 | 0.7 | 0.6 | 0.89 | 0.7 | C3 | TCGA-K4-A3WV | 42.109 |  |  |  |  |  |
| TCGA-HQ-A2OF | 5.3342466 | 0 | 0.7 | 0.65 | 0.6 | 0.6 | 0.81 | 0.6 | C3 | TCGA-K4-A4AC | 24.643 |  |  |  |  |  |
| TCGA-XF-AAN1 | 2.5780822 | 1 | 0.7 | 0.68 | 0.6 | 0.6 | 0.81 | 0.7 | C3 | TCGA-K4-A5RI | 31.736 |  |  |  |  |  |
| TCGA-DK-AA6P | 1.2520548 | 0 | 0.7 | 0.67 | 0.6 | 0.6 | 0.8 | 0.7 | C3 | TCGA-K4-A6MB | 14.133 |  |  |  |  |  |
| TCGA-CF-A47Y | 1.0219178 | 0 | 0.7 | 0.65 | 0.6 | 0.5 | 0.81 | 0.7 | C3 | TCGA-KQ-A41N | 9.0543 |  |  |  |  |  |
| TCGA-CF-A3MI | 1.0136986 | 0 | 0.7 | 0.68 | 0.6 | 0.6 | 0.83 | 0.7 | C3 | TCGA-KQ-A41P | 12.688 |  |  |  |  |  |
| TCGA-G2-A2EL | 2.2438356 | 1 | 0.6 | 0.63 | 0.6 | 0.6 | 0.74 | 0.6 | C3 | TCGA-KQ-A41Q | 16.242 |  |  |  |  |  |
| TCGA-XF-AAMZ | 3.6931507 | 1 | 0.7 | 0.7 | 0.7 | 0.7 | 0.84 | 0.8 | C3 | TCGA-MV-A51V | 15.232 |  |  |  |  |  |
| TCGA-DK-AA6L | 3.1863014 | 1 | 0.7 | 0.7 | 0.6 | 0.6 | 0.84 | 0.7 | C3 | TCGA-PQ-A6FI | 37.621 |  |  |  |  |  |
| TCGA-XF-A9T4 | 1.3561644 | 1 | 0.8 | 0.72 | 0.7 | 0.7 | 0.84 | 0.8 | C3 | TCGA-PQ-A6FN | 14.442 |  |  |  |  |  |
| TCGA-K4-A6MB | 1.2849315 | 0 | 0.7 | 0.68 | 0.6 | 0.6 | 0.83 | 0.7 | C3 | TCGA-UY-A78L | 32.442 |  |  |  |  |  |
| TCGA-UY-A8OB | 5.7780822 | 0 | 0.7 | 0.68 | 0.7 | 0.6 | 0.81 | 0.7 | C3 | TCGA-UY-A78M | 11.593 |  |  |  |  |  |
| TCGA-ZF-A9R5 | 2.9863014 | 0 | 0.7 | 0.68 | 0.6 | 0.6 | 0.81 | 0.7 | C3 | TCGA-UY-A78N | 12.827 |  |  |  |  |  |
| TCGA-CU-A3KJ | 1.539726 | 0 | 0.7 | 0.7 | 0.7 | 0.6 | 0.82 | 0.7 | C3 | TCGA-UY-A8OB | 21.339 |  |  |  |  |  |
| TCGA-DK-AA6S | 13.835616 | 0 | 0.8 | 0.74 | 0.7 | 0.7 | 0.86 | 0.7 | C3 | TCGA-UY-A9PD | 9.7957 |  |  |  |  |  |
| TCGA-E7-A85H | 1.0794521 | 0 | 0.7 | 0.68 | 0.7 | 0.6 | 0.84 | 0.7 | C3 | TCGA-UY-A9PE | 13.714 |  |  |  |  |  |
| TCGA-G2-AA3C | 0.5780822 | 1 | 0.8 | 0.71 | 0.7 | 0.6 | 0.89 | 0.7 | C3 | TCGA-XF-A8HB | 16.358 |  |  |  |  |  |
| TCGA-E7-A8O8 | 0.0356164 | 0 | 0.7 | 0.65 | 0.6 | 0.6 | 0.82 | 0.7 | C3 | TCGA-XF-A8HC | 17.573 |  |  |  |  |  |
| TCGA-XF-A9SM | 2.8712329 | 0 | 0.8 | 0.76 | 0.7 | 0.7 | 0.88 | 0.8 | C3 | TCGA-XF-A8HF | 11.409 |  |  |  |  |  |
| TCGA-DK-A3IN | 0.6849315 | 1 | 0.8 | 0.72 | 0.7 | 0.7 | 0.85 | 0.7 | C3 | TCGA-XF-A9ST | 4.5895 |  |  |  |  |  |
| TCGA-CF-A47W | 1.0082192 | 0 | 0.7 | 0.64 | 0.6 | 0.6 | 0.82 | 0.6 | C3 | TCGA-XF-A9T0 | 14.294 |  |  |  |  |  |
| TCGA-GC-A3I6 | 1.7260274 | 1 | 0.8 | 0.71 | 0.7 | 0.6 | 0.85 | 0.7 | C3 | TCGA-XF-A9T2 | 12.654 |  |  |  |  |  |
| TCGA-FD-A6TA | 5.2383562 | 0 | 0.8 | 0.73 | 0.7 | 0.7 | 0.86 | 0.8 | C3 | TCGA-XF-A9T6 | 12.244 |  |  |  |  |  |
| TCGA-KQ-A41P | 2.9972603 | 0 | 0.7 | 0.69 | 0.6 | 0.6 | 0.86 | 0.7 | C3 | TCGA-XF-AAMG | 27.78 |  |  |  |  |  |
| TCGA-UY-A9PH | 4.2767123 | 0 | 0.8 | 0.74 | 0.7 | 0.7 | 0.88 | 0.8 | C3 | TCGA-XF-AAMH | 12.943 |  |  |  |  |  |
| TCGA-FD-A5BY | 0.6876712 | 0 | 0.7 | 0.69 | 0.7 | 0.6 | 0.84 | 0.7 | C3 | TCGA-XF-AAMX | 10.74 |  |  |  |  |  |
| TCGA-K4-A3WU | 0.2876712 | 0 | 0.8 | 0.76 | 0.7 | 0.7 | 0.87 | 0.8 | C3 | TCGA-YC-A89H | 9.5457 |  |  |  |  |  |
| TCGA-FD-A3SJ | 2.0246575 | 1 | 0.7 | 0.7 | 0.7 | 0.6 | 0.84 | 0.7 | C3 | TCGA-YC-A9TC | 11.481 |  |  |  |  |  |
| TCGA-HQ-A5NE | 1.0136986 | 1 | 0.8 | 0.72 | 0.7 | 0.7 | 0.85 | 0.8 | C3 | TCGA-YF-AA3M | 25.996 |  |  |  |  |  |
| TCGA-XF-AAMT | 0.2465753 | 1 | 0.8 | 0.73 | 0.7 | 0.7 | 0.86 | 0.7 | C3 | TCGA-ZF-A9R1 | 22.772 |  |  |  |  |  |
| TCGA-XF-A9SU | 0.4986301 | 1 | 0.7 | 0.71 | 0.7 | 0.7 | 0.86 | 0.7 | C3 | TCGA-ZF-A9R2 | 25.665 |  |  |  |  |  |
| TCGA-CF-A47S | 0.9123288 | 0 | 0.7 | 0.69 | 0.6 | 0.6 | 0.83 | 0.7 | C3 | TCGA-ZF-A9RC | 18.131 |  |  |  |  |  |
| TCGA-FD-A6TK | 0.9041096 | 0 | 0.8 | 0.75 | 0.7 | 0.7 | 0.87 | 0.8 | C3 | TCGA-ZF-A9RE | 38.03 |  |  |  |  |  |
| TCGA-E7-A5KF | 0.0547945 | 0 | 0.7 | 0.65 | 0.6 | 0.6 | 0.81 | 0.7 | C3 | TCGA-ZF-AA4T | 16.591 |  |  |  |  |  |
| TCGA-UY-A78L | 3.0876712 | 0 | 0.7 | 0.68 | 0.6 | 0.6 | 0.84 | 0.7 | C3 | TCGA-ZF-AA4U | 17.671 |  |  |  |  |  |
| TCGA-S5-A6DX | 0.1534247 | 1 | 0.8 | 0.7 | 0.7 | 0.7 | 0.87 | 0.7 | C3 | TCGA-ZF-AA4V | 30.388 |  |  |  |  |  |
| TCGA-XF-A8HI | 1.490411 | 1 | 0.7 | 0.69 | 0.6 | 0.6 | 0.83 | 0.7 | C3 | TCGA-ZF-AA4W | 26.302 |  |  |  |  |  |
| TCGA-XF-A9T5 | 5.5534247 | 0 | 0.8 | 0.74 | 0.7 | 0.7 | 0.88 | 0.8 | C3 | TCGA-ZF-AA5N | 11.882 |  |  |  |  |  |
| TCGA-XF-A9SX | 1.969863 | 1 | 0.8 | 0.75 | 0.7 | 0.7 | 0.88 | 0.8 |  |  |  |  |  |  |  |  |
| TCGA-G2-A2EK | 1.3287671 | 0 | 0.7 | 0.72 | 0.7 | 0.7 | 0.84 | 0.8 |  |  |  |  |  |  |  |  |
| TCGA-DK-A6AW | 4.4410959 | 0 | 0.7 | 0.68 | 0.6 | 0.6 | 0.83 | 0.7 |  |  |  |  |  |  |  |  |
| TCGA-GC-A3BM | 1.7835616 | 1 | 0.7 | 0.69 | 0.7 | 0.6 | 0.82 | 0.7 |  |  |  |  |  |  |  |  |
| TCGA-GV-A6ZA | 1.8931507 | 0 | 0.7 | 0.67 | 0.6 | 0.6 | 0.82 | 0.7 |  |  |  |  |  |  |  |  |
| TCGA-G2-A2EJ | 4 | 0 | 0.7 | 0.69 | 0.7 | 0.6 | 0.81 | 0.7 |  |  |  |  |  |  |  |  |
| TCGA-FD-A43N | 1.9150685 | 0 | 0.7 | 0.7 | 0.7 | 0.6 | 0.84 | 0.7 |  |  |  |  |  |  |  |  |
| TCGA-GV-A3QH | 0.7068493 | 1 | 0.7 | 0.65 | 0.6 | 0.6 | 0.79 | 0.7 |  |  |  |  |  |  |  |  |
| TCGA-BT-A0YX | 1.0958904 | 1 | 0.8 | 0.72 | 0.7 | 0.7 | 0.85 | 0.8 |  |  |  |  |  |  |  |  |
| TCGA-FT-A3EE | 0.2712329 | 1 | 0.7 | 0.7 | 0.7 | 0.6 | 0.83 | 0.7 |  |  |  |  |  |  |  |  |
| TCGA-ZF-A9RL | 7.4054795 | 0 | 0.7 | 0.64 | 0.6 | 0.5 | 0.78 | 0.6 |  |  |  |  |  |  |  |  |
| TCGA-ZF-A9R9 | 2.3671233 | 1 | 0.7 | 0.72 | 0.7 | 0.6 | 0.86 | 0.7 |  |  |  |  |  |  |  |  |
| TCGA-FD-A5BX | 0.4739726 | 1 | 0.8 | 0.74 | 0.7 | 0.7 | 0.88 | 0.7 |  |  |  |  |  |  |  |  |
| TCGA-BL-A13J | 0.2219178 | 1 | 0.7 | 0.68 | 0.6 | 0.6 | 0.79 | 0.7 |  |  |  |  |  |  |  |  |
| TCGA-FT-A61P | 0.9232877 | 0 | 0.8 | 0.74 | 0.7 | 0.7 | 0.87 | 0.8 |  |  |  |  |  |  |  |  |
| TCGA-DK-AA77 | 1.6931507 | 0 | 0.7 | 0.7 | 0.7 | 0.6 | 0.84 | 0.7 |  |  |  |  |  |  |  |  |
| TCGA-ZF-AA5N | 0.460274 | 1 | 0.7 | 0.65 | 0.6 | 0.6 | 0.81 | 0.7 |  |  |  |  |  |  |  |  |
| TCGA-DK-AA6U | 1.5835616 | 0 | 0.7 | 0.67 | 0.6 | 0.6 | 0.8 | 0.7 |  |  |  |  |  |  |  |  |
| TCGA-BT-A20O | 1.0136986 | 1 | 0.8 | 0.79 | 0.8 | 0.7 | 0.9 | 0.8 |  |  |  |  |  |  |  |  |
| TCGA-GD-A3OS | 1.7479452 | 0 | 0.7 | 0.72 | 0.6 | 0.6 | 0.83 | 0.7 |  |  |  |  |  |  |  |  |
| TCGA-XF-A9SZ | 2.3534247 | 1 | 0.7 | 0.7 | 0.7 | 0.6 | 0.86 | 0.7 |  |  |  |  |  |  |  |  |
| TCGA-4Z-AA82 | 4.2630137 | 1 | 0.7 | 0.7 | 0.7 | 0.6 | 0.84 | 0.7 |  |  |  |  |  |  |  |  |
| TCGA-DK-AA76 | 1.0027397 | 0 | 0.7 | 0.64 | 0.6 | 0.6 | 0.77 | 0.6 |  |  |  |  |  |  |  |  |
| TCGA-DK-A3IL | 1.1315068 | 1 | 0.7 | 0.67 | 0.6 | 0.6 | 0.82 | 0.7 |  |  |  |  |  |  |  |  |
| TCGA-E7-A678 | 2.1863014 | 0 | 0.7 | 0.67 | 0.6 | 0.6 | 0.83 | 0.7 |  |  |  |  |  |  |  |  |
| TCGA-ZF-A9R1 | 2.1178082 | 0 | 0.7 | 0.69 | 0.6 | 0.6 | 0.82 | 0.7 |  |  |  |  |  |  |  |  |
| TCGA-XF-A9SH | 5.4 | 1 | 0.7 | 0.69 | 0.6 | 0.6 | 0.85 | 0.7 |  |  |  |  |  |  |  |  |
| TCGA-FD-A5BU | 1.6109589 | 0 | 0.8 | 0.74 | 0.7 | 0.7 | 0.86 | 0.8 |  |  |  |  |  |  |  |  |
| TCGA-C4-A0F7 | 0.169863 | 1 | 0.7 | 0.67 | 0.7 | 0.6 | 0.82 | 0.7 |  |  |  |  |  |  |  |  |
| TCGA-C4-A0F0 | 0.1616438 | 0 | 0.7 | 0.7 | 0.7 | 0.7 | 0.84 | 0.7 |  |  |  |  |  |  |  |  |
| TCGA-FD-A3N5 | 1.8767123 | 1 | 0.7 | 0.7 | 0.7 | 0.6 | 0.82 | 0.7 |  |  |  |  |  |  |  |  |
| TCGA-4Z-AA7S | 2.9150685 | 1 | 0.7 | 0.67 | 0.6 | 0.6 | 0.81 | 0.7 |  |  |  |  |  |  |  |  |
| TCGA-CF-A3MH | 1.090411 | 0 | 0.7 | 0.66 | 0.6 | 0.6 | 0.83 | 0.7 |  |  |  |  |  |  |  |  |
| TCGA-DK-A6B2 | 1.3068493 | 0 | 0.8 | 0.74 | 0.7 | 0.7 | 0.87 | 0.8 |  |  |  |  |  |  |  |  |
| TCGA-4Z-AA86 | 0.8520548 | 1 | 0.8 | 0.75 | 0.7 | 0.7 | 0.86 | 0.8 |  |  |  |  |  |  |  |  |
| TCGA-5N-A9KI | 0.2082192 | 1 | 0.8 | 0.72 | 0.7 | 0.7 | 0.87 | 0.7 |  |  |  |  |  |  |  |  |
| TCGA-GU-AATO | 0.8876712 | 1 | 0.7 | 0.71 | 0.7 | 0.7 | 0.87 | 0.8 |  |  |  |  |  |  |  |  |
| TCGA-CF-A9FH | -0.175342 | 0 | 0.7 | 0.67 | 0.6 | 0.6 | 0.81 | 0.7 |  |  |  |  |  |  |  |  |
| TCGA-FD-A6TG | 0.2547945 | 1 | 0.8 | 0.73 | 0.7 | 0.7 | 0.86 | 0.8 |  |  |  |  |  |  |  |  |
| TCGA-4Z-AA7O | 1.4027397 | 0 | 0.7 | 0.7 | 0.7 | 0.6 | 0.84 | 0.8 |  |  |  |  |  |  |  |  |
| TCGA-GV-A3QI | 3.0410959 | 0 | 0.7 | 0.65 | 0.6 | 0.6 | 0.78 | 0.6 |  |  |  |  |  |  |  |  |
| TCGA-DK-A6AV | 5.3479452 | 0 | 0.7 | 0.71 | 0.7 | 0.7 | 0.83 | 0.7 |  |  |  |  |  |  |  |  |
| TCGA-DK-A1A3 | 1.8219178 | 1 | 0.8 | 0.72 | 0.7 | 0.7 | 0.86 | 0.8 |  |  |  |  |  |  |  |  |
| TCGA-GD-A6C6 | 0.1835616 | 0 | 0.7 | 0.62 | 0.6 | 0.6 | 0.81 | 0.7 |  |  |  |  |  |  |  |  |
| TCGA-GC-A3RD | 1.1726027 | 0 | 0.7 | 0.67 | 0.6 | 0.6 | 0.83 | 0.7 |  |  |  |  |  |  |  |  |
| TCGA-BT-A20R | 0.4219178 | 1 | 0.8 | 0.71 | 0.7 | 0.6 | 0.87 | 0.7 |  |  |  |  |  |  |  |  |
| TCGA-CF-A8HX | 0.9452055 | 0 | 0.7 | 0.66 | 0.6 | 0.6 | 0.81 | 0.6 |  |  |  |  |  |  |  |  |
| TCGA-PQ-A6FI | 1.0191781 | 0 | 0.7 | 0.68 | 0.7 | 0.6 | 0.83 | 0.7 |  |  |  |  |  |  |  |  |
| TCGA-FJ-A3Z7 | 2.5890411 | 0 | 0.7 | 0.71 | 0.7 | 0.6 | 0.82 | 0.7 |  |  |  |  |  |  |  |  |
| TCGA-FD-A3SP | 2.1452055 | 0 | 0.8 | 0.76 | 0.7 | 0.7 | 0.88 | 0.8 |  |  |  |  |  |  |  |  |
| TCGA-CF-A1HS | 1.0465753 | 0 | 0.7 | 0.71 | 0.7 | 0.6 | 0.84 | 0.8 |  |  |  |  |  |  |  |  |
| TCGA-DK-AA71 | 1.1369863 | 0 | 0.7 | 0.67 | 0.6 | 0.6 | 0.83 | 0.7 |  |  |  |  |  |  |  |  |
| TCGA-KQ-A41O | 4.2136986 | 0 | 0.6 | 0.58 | 0.5 | 0.5 | 0.73 | 0.6 |  |  |  |  |  |  |  |  |
| TCGA-E7-A677 | 2.2465753 | 0 | 0.7 | 0.72 | 0.7 | 0.6 | 0.82 | 0.7 |  |  |  |  |  |  |  |  |
| TCGA-DK-A1A5 | 0.1780822 | 1 | 0.8 | 0.72 | 0.7 | 0.6 | 0.84 | 0.7 |  |  |  |  |  |  |  |  |
| TCGA-FD-A3B4 | 1.3972603 | 1 | 0.8 | 0.73 | 0.7 | 0.7 | 0.85 | 0.8 |  |  |  |  |  |  |  |  |
| TCGA-DK-A1AB | 1.3917808 | 1 | 0.8 | 0.73 | 0.7 | 0.7 | 0.86 | 0.8 |  |  |  |  |  |  |  |  |
| TCGA-ZF-AA4N | 0.2410959 | 1 | 0.8 | 0.71 | 0.7 | 0.6 | 0.85 | 0.7 |  |  |  |  |  |  |  |  |
| TCGA-BT-A20X | 0.6876712 | 1 | 0.7 | 0.69 | 0.7 | 0.6 | 0.83 | 0.7 |  |  |  |  |  |  |  |  |
| TCGA-GV-A3JW | 1.7780822 | 0 | 0.7 | 0.61 | 0.6 | 0.5 | 0.79 | 0.6 |  |  |  |  |  |  |  |  |
| TCGA-UY-A78P | 6.5205479 | 0 | 0.8 | 0.75 | 0.7 | 0.7 | 0.88 | 0.8 |  |  |  |  |  |  |  |  |
| TCGA-BL-A0C8 | 3.339726 | 0 | 0.7 | 0.67 | 0.6 | 0.6 | 0.81 | 0.6 |  |  |  |  |  |  |  |  |
| TCGA-BT-A42C | 2.3917808 | 0 | 0.7 | 0.67 | 0.6 | 0.6 | 0.81 | 0.7 |  |  |  |  |  |  |  |  |
| TCGA-G2-A3IB | 0.6027397 | 1 | 0.7 | 0.66 | 0.6 | 0.6 | 0.82 | 0.7 |  |  |  |  |  |  |  |  |
| TCGA-2F-A9KP | 0.9972603 | 1 | 0.7 | 0.68 | 0.6 | 0.6 | 0.8 | 0.7 |  |  |  |  |  |  |  |  |
| TCGA-DK-A3IT | 1.7753425 | 0 | 0.8 | 0.71 | 0.7 | 0.7 | 0.84 | 0.8 |  |  |  |  |  |  |  |  |
| TCGA-FD-A43P | 2.2794521 | 0 | 0.8 | 0.74 | 0.7 | 0.7 | 0.86 | 0.8 |  |  |  |  |  |  |  |  |
| TCGA-DK-A3IV | 0.8054795 | 1 | 0.8 | 0.74 | 0.7 | 0.7 | 0.84 | 0.8 |  |  |  |  |  |  |  |  |
| TCGA-2F-A9KW | 0.6958904 | 1 | 0.7 | 0.7 | 0.7 | 0.6 | 0.85 | 0.7 |  |  |  |  |  |  |  |  |
| TCGA-GU-A762 | 0.6356164 | 1 | 0.8 | 0.75 | 0.7 | 0.7 | 0.87 | 0.8 |  |  |  |  |  |  |  |  |
| TCGA-XF-AAME | 7.7479452 | 1 | 0.8 | 0.78 | 0.7 | 0.7 | 0.9 | 0.8 |  |  |  |  |  |  |  |  |
| TCGA-XF-AAN0 | 4.7068493 | 1 | 0.8 | 0.72 | 0.7 | 0.7 | 0.86 | 0.8 |  |  |  |  |  |  |  |  |
| TCGA-DK-A6B1 | 5.6136986 | 0 | 0.7 | 0.68 | 0.6 | 0.6 | 0.82 | 0.7 |  |  |  |  |  |  |  |  |
| TCGA-CF-A5U8 | 1.0931507 | 0 | 0.7 | 0.65 | 0.6 | 0.6 | 0.81 | 0.7 |  |  |  |  |  |  |  |  |
| TCGA-XF-AAMW | 0.6931507 | 1 | 0.8 | 0.73 | 0.7 | 0.7 | 0.84 | 0.8 |  |  |  |  |  |  |  |  |
| TCGA-BT-A3PJ | 2.1616438 | 0 | 0.7 | 0.72 | 0.7 | 0.6 | 0.83 | 0.7 |  |  |  |  |  |  |  |  |
| TCGA-YC-A9TC | 0.0547945 | 1 | 0.7 | 0.66 | 0.6 | 0.6 | 0.8 | 0.7 |  |  |  |  |  |  |  |  |
| TCGA-GV-A3QG | 0 | 1 | 0.8 | 0.79 | 0.7 | 0.8 | 0.89 | 0.9 |  |  |  |  |  |  |  |  |
| TCGA-K4-A6FZ | 0.1506849 | 0 | 0.8 | 0.73 | 0.7 | 0.7 | 0.86 | 0.8 |  |  |  |  |  |  |  |  |
| TCGA-KQ-A41R | 3.6986301 | 0 | 0.7 | 0.68 | 0.6 | 0.6 | 0.8 | 0.7 |  |  |  |  |  |  |  |  |
| TCGA-DK-AA6M | 4.3342466 | 0 | 0.8 | 0.73 | 0.7 | 0.7 | 0.86 | 0.8 |  |  |  |  |  |  |  |  |
| TCGA-ZF-AA52 | 2.9506849 | 1 | 0.8 | 0.72 | 0.7 | 0.7 | 0.88 | 0.7 |  |  |  |  |  |  |  |  |
| TCGA-K4-A5RJ | 1.4767123 | 0 | 0.8 | 0.76 | 0.7 | 0.7 | 0.89 | 0.8 |  |  |  |  |  |  |  |  |
| TCGA-HQ-A5ND | 0.7506849 | 1 | 0.7 | 0.66 | 0.7 | 0.6 | 0.8 | 0.7 |  |  |  |  |  |  |  |  |
| TCGA-BT-A3PK | 0.830137 | 1 | 0.8 | 0.73 | 0.7 | 0.7 | 0.86 | 0.7 |  |  |  |  |  |  |  |  |
| TCGA-FD-A3N6 | 2.3315068 | 0 | 0.7 | 0.68 | 0.7 | 0.6 | 0.81 | 0.7 |  |  |  |  |  |  |  |  |
| TCGA-CF-A47X | 1.0520548 | 0 | 0.7 | 0.66 | 0.6 | 0.6 | 0.79 | 0.7 |  |  |  |  |  |  |  |  |
| TCGA-ZF-AA51 | 4.6958904 | 0 | 0.8 | 0.74 | 0.7 | 0.7 | 0.87 | 0.8 |  |  |  |  |  |  |  |  |
| TCGA-UY-A78O | 6.3342466 | 0 | 0.7 | 0.67 | 0.6 | 0.6 | 0.82 | 0.7 |  |  |  |  |  |  |  |  |
| TCGA-GC-A3OO | 1.3178082 | 0 | 0.8 | 0.71 | 0.7 | 0.7 | 0.86 | 0.8 |  |  |  |  |  |  |  |  |
| TCGA-FD-A43Y | 1.2986301 | 1 | 0.7 | 0.71 | 0.7 | 0.6 | 0.84 | 0.7 |  |  |  |  |  |  |  |  |
| TCGA-S5-AA26 | 1.3780822 | 0 | 0.7 | 0.63 | 0.6 | 0.5 | 0.79 | 0.7 |  |  |  |  |  |  |  |  |
| TCGA-BT-A42E | 3.0356164 | 0 | 0.8 | 0.72 | 0.7 | 0.6 | 0.85 | 0.8 |  |  |  |  |  |  |  |  |
| TCGA-FD-A43X | 0.3013699 | 0 | 0.7 | 0.65 | 0.6 | 0.5 | 0.8 | 0.7 |  |  |  |  |  |  |  |  |
| TCGA-ZF-AA4R | 2.8383562 | 1 | 0.7 | 0.7 | 0.7 | 0.6 | 0.82 | 0.7 |  |  |  |  |  |  |  |  |
| TCGA-BT-A20T | 1.2410959 | 1 | 0.7 | 0.72 | 0.7 | 0.6 | 0.85 | 0.7 |  |  |  |  |  |  |  |  |
| TCGA-E7-A7DU | 0.0767123 | 0 | 0.7 | 0.67 | 0.6 | 0.6 | 0.82 | 0.7 |  |  |  |  |  |  |  |  |
| TCGA-DK-A3WY | 13.608219 | 0 | 0.8 | 0.8 | 0.7 | 0.8 | 0.9 | 0.9 |  |  |  |  |  |  |  |  |
| TCGA-G2-A2EF | 5.1616438 | 0 | 0.8 | 0.72 | 0.7 | 0.7 | 0.84 | 0.8 |  |  |  |  |  |  |  |  |
| TCGA-C4-A0F6 | 1.9178082 | 0 | 0.7 | 0.68 | 0.6 | 0.6 | 0.84 | 0.7 |  |  |  |  |  |  |  |  |
| TCGA-CF-A7I0 | 1.0082192 | 0 | 0.7 | 0.7 | 0.6 | 0.6 | 0.84 | 0.7 |  |  |  |  |  |  |  |  |
| TCGA-FD-A3B6 | 2.7534247 | 1 | 0.8 | 0.72 | 0.7 | 0.7 | 0.86 | 0.8 |  |  |  |  |  |  |  |  |
| TCGA-GC-A3YS | 2.0767123 | 0 | 0.7 | 0.72 | 0.7 | 0.6 | 0.85 | 0.7 |  |  |  |  |  |  |  |  |
| TCGA-XF-A9T2 | 1.5753425 | 1 | 0.7 | 0.69 | 0.6 | 0.6 | 0.83 | 0.7 |  |  |  |  |  |  |  |  |
| TCGA-XF-A9T0 | 2.1890411 | 0 | 0.7 | 0.68 | 0.6 | 0.6 | 0.84 | 0.7 |  |  |  |  |  |  |  |  |
| TCGA-YF-AA3M | 1.1369863 | 0 | 0.7 | 0.67 | 0.7 | 0.6 | 0.83 | 0.7 |  |  |  |  |  |  |  |  |
| TCGA-4Z-AA7Y | 4.169863 | 0 | 0.7 | 0.68 | 0.6 | 0.6 | 0.81 | 0.7 |  |  |  |  |  |  |  |  |
| TCGA-E7-A6ME | 2.0027397 | 0 | 0.7 | 0.7 | 0.7 | 0.6 | 0.83 | 0.7 |  |  |  |  |  |  |  |  |
| TCGA-XF-AAN5 | 6.2821918 | 0 | 0.8 | 0.73 | 0.7 | 0.7 | 0.86 | 0.8 |  |  |  |  |  |  |  |  |
| TCGA-FD-A3SN | 2.430137 | 0 | 0.7 | 0.68 | 0.7 | 0.6 | 0.84 | 0.7 |  |  |  |  |  |  |  |  |
| TCGA-ZF-A9R2 | 1.7589041 | 0 | 0.7 | 0.65 | 0.6 | 0.6 | 0.79 | 0.7 |  |  |  |  |  |  |  |  |
| TCGA-ZF-AA4T | 1.6410959 | 1 | 0.7 | 0.67 | 0.6 | 0.6 | 0.79 | 0.7 |  |  |  |  |  |  |  |  |
| TCGA-E7-A4IJ | 1.8465753 | 1 | 0.7 | 0.69 | 0.7 | 0.6 | 0.83 | 0.7 |  |  |  |  |  |  |  |  |
| TCGA-FD-A6TE | 1.030137 | 0 | 0.7 | 0.67 | 0.6 | 0.6 | 0.82 | 0.7 |  |  |  |  |  |  |  |  |
| TCGA-H4-A2HO | 0.1260274 | 0 | 0.7 | 0.7 | 0.7 | 0.6 | 0.85 | 0.7 |  |  |  |  |  |  |  |  |
| TCGA-FD-A6TB | 1.5671233 | 0 | 0.8 | 0.77 | 0.7 | 0.7 | 0.88 | 0.8 |  |  |  |  |  |  |  |  |
| TCGA-XF-AAN3 | 7.1917808 | 0 | 0.8 | 0.71 | 0.7 | 0.6 | 0.86 | 0.7 |  |  |  |  |  |  |  |  |
| TCGA-XF-AAMH | 0.9424658 | 1 | 0.7 | 0.66 | 0.6 | 0.6 | 0.81 | 0.7 |  |  |  |  |  |  |  |  |
| TCGA-FD-A3B8 | 1.0520548 | 0 | 0.8 | 0.8 | 0.8 | 0.7 | 0.89 | 0.8 |  |  |  |  |  |  |  |  |
| TCGA-XF-A9SY | 1.7534247 | 0 | 0.8 | 0.75 | 0.7 | 0.7 | 0.86 | 0.8 |  |  |  |  |  |  |  |  |
| TCGA-CF-A9FF | 0.9890411 | 0 | 0.7 | 0.71 | 0.7 | 0.7 | 0.84 | 0.8 |  |  |  |  |  |  |  |  |
| TCGA-XF-A9ST | 0.3506849 | 1 | 0.7 | 0.64 | 0.6 | 0.6 | 0.79 | 0.6 |  |  |  |  |  |  |  |  |
| TCGA-DK-A6B5 | 4.2246575 | 0 | 0.7 | 0.7 | 0.6 | 0.6 | 0.85 | 0.7 |  |  |  |  |  |  |  |  |
| TCGA-GC-A3RC | 1.3260274 | 0 | 0.8 | 0.73 | 0.7 | 0.7 | 0.85 | 0.8 |  |  |  |  |  |  |  |  |
| TCGA-GV-A3QK | 2.2794521 | 0 | 0.7 | 0.72 | 0.7 | 0.6 | 0.84 | 0.7 |  |  |  |  |  |  |  |  |
| TCGA-GU-A764 | 1.6712329 | 0 | 0.8 | 0.73 | 0.7 | 0.7 | 0.86 | 0.8 |  |  |  |  |  |  |  |  |
| TCGA-GU-A42R | 1.5808219 | 1 | 0.7 | 0.65 | 0.6 | 0.6 | 0.79 | 0.7 |  |  |  |  |  |  |  |  |
| TCGA-DK-A6B6 | 3.0547945 | 0 | 0.7 | 0.71 | 0.7 | 0.6 | 0.83 | 0.7 |  |  |  |  |  |  |  |  |
| TCGA-R3-A69X | 1.1863014 | 0 | 0.8 | 0.75 | 0.7 | 0.7 | 0.87 | 0.8 |  |  |  |  |  |  |  |  |
| TCGA-5N-A9KM | 1.4520548 | 1 | 0.8 | 0.71 | 0.7 | 0.6 | 0.86 | 0.7 |  |  |  |  |  |  |  |  |
| TCGA-4Z-AA7M | 1.3561644 | 0 | 0.7 | 0.65 | 0.6 | 0.6 | 0.81 | 0.7 |  |  |  |  |  |  |  |  |
| TCGA-ZF-AA56 | 0.709589 | 1 | 0.8 | 0.74 | 0.7 | 0.7 | 0.87 | 0.8 |  |  |  |  |  |  |  |  |
| TCGA-XF-A8HH | 0.1561644 | 1 | 0.7 | 0.71 | 0.7 | 0.6 | 0.85 | 0.7 |  |  |  |  |  |  |  |  |
| TCGA-C4-A0EZ | 0.7479452 | 1 | 0.6 | 0.64 | 0.6 | 0.6 | 0.77 | 0.6 |  |  |  |  |  |  |  |  |
| TCGA-UY-A8OD | 9.4027397 | 0 | 0.8 | 0.73 | 0.7 | 0.7 | 0.86 | 0.8 |  |  |  |  |  |  |  |  |
| TCGA-2F-A9KT | 6.4438356 | 0 | 0.7 | 0.66 | 0.6 | 0.6 | 0.82 | 0.7 |  |  |  |  |  |  |  |  |
| TCGA-BT-A20Q | 1.6246575 | 1 | 0.8 | 0.74 | 0.7 | 0.7 | 0.85 | 0.8 |  |  |  |  |  |  |  |  |
| TCGA-CU-A0YR | 1.260274 | 1 | 0.8 | 0.73 | 0.7 | 0.7 | 0.86 | 0.8 |  |  |  |  |  |  |  |  |
| TCGA-SY-A9G5 | 3.2493151 | 0 | 0.8 | 0.74 | 0.7 | 0.6 | 0.87 | 0.8 |  |  |  |  |  |  |  |  |
| TCGA-4Z-AA84 | 1.260274 | 0 | 0.7 | 0.67 | 0.6 | 0.6 | 0.81 | 0.7 |  |  |  |  |  |  |  |  |
| TCGA-4Z-AA7Q | 1.3972603 | 1 | 0.8 | 0.72 | 0.7 | 0.7 | 0.87 | 0.8 |  |  |  |  |  |  |  |  |
| TCGA-FD-A5BT | 0.8986301 | 1 | 0.8 | 0.74 | 0.7 | 0.7 | 0.89 | 0.8 |  |  |  |  |  |  |  |  |
| TCGA-CF-A27C | 1.1643836 | 0 | 0.7 | 0.64 | 0.6 | 0.5 | 0.79 | 0.7 |  |  |  |  |  |  |  |  |
| TCGA-E7-A6MF | 2.0547945 | 0 | 0.7 | 0.7 | 0.6 | 0.6 | 0.83 | 0.7 |  |  |  |  |  |  |  |  |
| TCGA-DK-A3WW | 1.7342466 | 0 | 0.8 | 0.73 | 0.7 | 0.7 | 0.87 | 0.8 |  |  |  |  |  |  |  |  |
| TCGA-XF-AAMY | 8.2493151 | 0 | 0.7 | 0.7 | 0.7 | 0.6 | 0.85 | 0.7 |  |  |  |  |  |  |  |  |
| TCGA-K4-A3WS | 2.0849315 | 0 | 0.8 | 0.75 | 0.7 | 0.7 | 0.87 | 0.8 |  |  |  |  |  |  |  |  |
| TCGA-UY-A9PA | 2.9369863 | 0 | 0.8 | 0.72 | 0.7 | 0.6 | 0.85 | 0.7 |  |  |  |  |  |  |  |  |
| TCGA-2F-A9KR | 8.7205479 | 1 | 0.7 | 0.67 | 0.6 | 0.6 | 0.81 | 0.7 |  |  |  |  |  |  |  |  |
| TCGA-E5-A4U1 | 3.2356164 | 0 | 0.6 | 0.61 | 0.6 | 0.5 | 0.77 | 0.6 |  |  |  |  |  |  |  |  |
| TCGA-E7-A3Y1 | 0.4465753 | 0 | 0.7 | 0.66 | 0.6 | 0.6 | 0.82 | 0.7 |  |  |  |  |  |  |  |  |
| TCGA-FD-A6TC | 0.5123288 | 0 | 0.7 | 0.69 | 0.6 | 0.6 | 0.84 | 0.7 |  |  |  |  |  |  |  |  |
| TCGA-FD-A5BV | 0.4465753 | 1 | 0.7 | 0.68 | 0.6 | 0.6 | 0.82 | 0.7 |  |  |  |  |  |  |  |  |
| TCGA-DK-A2I4 | 10.506849 | 0 | 0.8 | 0.78 | 0.8 | 0.7 | 0.9 | 0.8 |  |  |  |  |  |  |  |  |
| TCGA-UY-A78M | 1.890411 | 1 | 0.7 | 0.65 | 0.6 | 0.6 | 0.81 | 0.7 |  |  |  |  |  |  |  |  |
| TCGA-4Z-AA7R | 1.430137 | 1 | 0.7 | 0.66 | 0.6 | 0.6 | 0.8 | 0.7 |  |  |  |  |  |  |  |  |
| TCGA-FD-A62P | 0.5232877 | 1 | 0.8 | 0.73 | 0.7 | 0.6 | 0.87 | 0.7 |  |  |  |  |  |  |  |  |
| TCGA-CF-A47V | 1.0383562 | 0 | 0.7 | 0.7 | 0.6 | 0.6 | 0.83 | 0.7 |  |  |  |  |  |  |  |  |
| TCGA-XF-A9SI | 6.6383562 | 0 | 0.8 | 0.79 | 0.7 | 0.7 | 0.9 | 0.8 |  |  |  |  |  |  |  |  |
| TCGA-GU-A42Q | 0.9424658 | 1 | 0.7 | 0.65 | 0.6 | 0.6 | 0.79 | 0.7 |  |  |  |  |  |  |  |  |
| TCGA-BT-A42F | 2.3671233 | 0 | 0.8 | 0.72 | 0.7 | 0.7 | 0.87 | 0.8 |  |  |  |  |  |  |  |  |
| TCGA-FD-A3B5 | 0.7452055 | 1 | 0.7 | 0.68 | 0.6 | 0.6 | 0.82 | 0.7 |  |  |  |  |  |  |  |  |
| TCGA-DK-A1A7 | 1.5342466 | 0 | 0.7 | 0.69 | 0.6 | 0.6 | 0.82 | 0.7 |  |  |  |  |  |  |  |  |
| TCGA-FD-A3SR | 1.6493151 | 1 | 0.8 | 0.74 | 0.7 | 0.7 | 0.86 | 0.8 |  |  |  |  |  |  |  |  |
| TCGA-GU-AATP | 2.7479452 | 0 | 0.7 | 0.7 | 0.7 | 0.6 | 0.84 | 0.7 |  |  |  |  |  |  |  |  |
| TCGA-DK-AA6X | 1.2794521 | 0 | 0.8 | 0.72 | 0.7 | 0.7 | 0.86 | 0.8 |  |  |  |  |  |  |  |  |
| TCGA-XF-A9T3 | 0.1863014 | 0 | 0.8 | 0.73 | 0.7 | 0.7 | 0.85 | 0.8 |  |  |  |  |  |  |  |  |
| TCGA-PQ-A6FN | 1.3890411 | 0 | 0.7 | 0.68 | 0.6 | 0.7 | 0.84 | 0.7 |  |  |  |  |  |  |  |  |
| TCGA-E7-A519 | 1.3917808 | 0 | 0.7 | 0.7 | 0.6 | 0.6 | 0.86 | 0.7 |  |  |  |  |  |  |  |  |
| TCGA-DK-A3IU | 1.9342466 | 1 | 0.8 | 0.78 | 0.7 | 0.7 | 0.9 | 0.8 |  |  |  |  |  |  |  |  |
| TCGA-FD-A3SM | 1.4986301 | 1 | 0.8 | 0.7 | 0.7 | 0.6 | 0.84 | 0.7 |  |  |  |  |  |  |  |  |
| TCGA-BL-A5ZZ | 1.0328767 | 0 | 0.8 | 0.75 | 0.7 | 0.7 | 0.88 | 0.7 |  |  |  |  |  |  |  |  |
| TCGA-FD-A62N | 0.2246575 | 0 | 0.8 | 0.78 | 0.7 | 0.7 | 0.9 | 0.8 |  |  |  |  |  |  |  |  |
| TCGA-BT-A20J | 1.5863014 | 1 | 0.8 | 0.73 | 0.7 | 0.7 | 0.87 | 0.8 |  |  |  |  |  |  |  |  |
| TCGA-K4-A3WV | 1.769863 | 0 | 0.7 | 0.65 | 0.6 | 0.6 | 0.79 | 0.7 |  |  |  |  |  |  |  |  |
| TCGA-GU-AATQ | 0.5835616 | 1 | 0.7 | 0.68 | 0.7 | 0.6 | 0.84 | 0.7 |  |  |  |  |  |  |  |  |
| TCGA-G2-A3VY | 1.4684932 | 0 | 0.7 | 0.64 | 0.6 | 0.5 | 0.79 | 0.7 |  |  |  |  |  |  |  |  |
| TCGA-XF-AAN8 | 0.3232877 | 1 | 0.8 | 0.78 | 0.7 | 0.7 | 0.91 | 0.8 |  |  |  |  |  |  |  |  |
| TCGA-CU-A3QU | 0.4328767 | 0 | 0.7 | 0.65 | 0.6 | 0.6 | 0.81 | 0.7 |  |  |  |  |  |  |  |  |
| TCGA-KQ-A41Q | 0.9890411 | 0 | 0.7 | 0.65 | 0.6 | 0.6 | 0.8 | 0.7 |  |  |  |  |  |  |  |  |
| TCGA-DK-A1AF | 1.4684932 | 0 | 0.8 | 0.77 | 0.7 | 0.7 | 0.89 | 0.8 |  |  |  |  |  |  |  |  |
| TCGA-LT-A5Z6 | 1.2986301 | 0 | 0.7 | 0.65 | 0.6 | 0.6 | 0.82 | 0.7 |  |  |  |  |  |  |  |  |
| TCGA-GC-A4ZW | 0.0410959 | 0 | 0.7 | 0.67 | 0.6 | 0.6 | 0.8 | 0.7 |  |  |  |  |  |  |  |  |
| TCGA-K4-A4AB | 0.2082192 | 0 | 0.8 | 0.72 | 0.7 | 0.6 | 0.85 | 0.7 |  |  |  |  |  |  |  |  |
| TCGA-DK-AA74 | 4.6794521 | 0 | 0.8 | 0.76 | 0.7 | 0.7 | 0.89 | 0.8 |  |  |  |  |  |  |  |  |
| TCGA-G2-A2EO | 4.9424658 | 1 | 0.8 | 0.72 | 0.7 | 0.7 | 0.86 | 0.7 |  |  |  |  |  |  |  |  |
| TCGA-DK-A1AG | 1.3013699 | 0 | 0.7 | 0.68 | 0.6 | 0.6 | 0.83 | 0.7 |  |  |  |  |  |  |  |  |
| TCGA-GV-A3JZ | 1.6520548 | 0 | 0.7 | 0.68 | 0.7 | 0.6 | 0.83 | 0.7 |  |  |  |  |  |  |  |  |
| TCGA-XF-A9SV | 1.0630137 | 1 | 0.7 | 0.69 | 0.6 | 0.6 | 0.83 | 0.7 |  |  |  |  |  |  |  |  |
| TCGA-UY-A78K | 1.4684932 | 1 | 0.8 | 0.74 | 0.7 | 0.7 | 0.86 | 0.8 |  |  |  |  |  |  |  |  |
| TCGA-FD-A6TH | 0.3589041 | 1 | 0.7 | 0.7 | 0.7 | 0.6 | 0.84 | 0.7 |  |  |  |  |  |  |  |  |
| TCGA-DK-AA6W | 1.1369863 | 1 | 0.7 | 0.69 | 0.6 | 0.6 | 0.81 | 0.7 |  |  |  |  |  |  |  |  |
| TCGA-CF-A3MG | 1.0109589 | 0 | 0.7 | 0.65 | 0.6 | 0.6 | 0.8 | 0.7 |  |  |  |  |  |  |  |  |
| TCGA-DK-A3X1 | 5.5041096 | 0 | 0.7 | 0.68 | 0.7 | 0.6 | 0.82 | 0.7 |  |  |  |  |  |  |  |  |
| TCGA-DK-A1A6 | 5.5342466 | 0 | 0.7 | 0.7 | 0.7 | 0.6 | 0.84 | 0.7 |  |  |  |  |  |  |  |  |
| TCGA-XF-AAMG | 9.2164384 | 0 | 0.7 | 0.68 | 0.6 | 0.6 | 0.81 | 0.7 |  |  |  |  |  |  |  |  |
| TCGA-E7-A541 | 2.1315068 | 1 | 0.8 | 0.72 | 0.7 | 0.7 | 0.85 | 0.8 |  |  |  |  |  |  |  |  |
| TCGA-XF-A8HE | 10.457534 | 0 | 0.8 | 0.73 | 0.7 | 0.7 | 0.85 | 0.8 |  |  |  |  |  |  |  |  |
| TCGA-DK-AA6Q | 1.1315068 | 1 | 0.8 | 0.71 | 0.7 | 0.6 | 0.84 | 0.7 |  |  |  |  |  |  |  |  |
| TCGA-UY-A78N | 7.2356164 | 1 | 0.7 | 0.65 | 0.6 | 0.6 | 0.8 | 0.6 |  |  |  |  |  |  |  |  |
| TCGA-DK-AA75 | 0.9315068 | 1 | 0.6 | 0.62 | 0.6 | 0.6 | 0.78 | 0.6 |  |  |  |  |  |  |  |  |
| TCGA-ZF-AA5P | 1.0191781 | 0 | 0.8 | 0.75 | 0.7 | 0.7 | 0.87 | 0.8 |  |  |  |  |  |  |  |  |
| TCGA-GC-A3WC | 1.4794521 | 0 | 0.8 | 0.74 | 0.7 | 0.7 | 0.86 | 0.8 |  |  |  |  |  |  |  |  |
| TCGA-K4-A4AC | 0.7616438 | 1 | 0.7 | 0.7 | 0.7 | 0.6 | 0.84 | 0.7 |  |  |  |  |  |  |  |  |
| TCGA-FD-A3SL | 1.9506849 | 1 | 0.8 | 0.74 | 0.7 | 0.7 | 0.86 | 0.8 |  |  |  |  |  |  |  |  |
| TCGA-K4-A5RH | 0.7561644 | 0 | 0.8 | 0.77 | 0.7 | 0.7 | 0.89 | 0.8 |  |  |  |  |  |  |  |  |
| TCGA-FD-A5C1 | 4.909589 | 0 | 0.8 | 0.74 | 0.7 | 0.7 | 0.87 | 0.8 |  |  |  |  |  |  |  |  |
| TCGA-XF-AAN7 | 1.5479452 | 1 | 0.7 | 0.69 | 0.7 | 0.6 | 0.84 | 0.7 |  |  |  |  |  |  |  |  |
| TCGA-2F-A9KO | 2.0109589 | 1 | 0.8 | 0.75 | 0.7 | 0.7 | 0.87 | 0.8 |  |  |  |  |  |  |  |  |
| TCGA-DK-A3IM | 0.6794521 | 1 | 0.7 | 0.65 | 0.6 | 0.6 | 0.8 | 0.7 |  |  |  |  |  |  |  |  |
| TCGA-FD-A3SS | 1.0712329 | 1 | 0.7 | 0.65 | 0.6 | 0.6 | 0.81 | 0.7 |  |  |  |  |  |  |  |  |
| TCGA-UY-A9PB | 2.4630137 | 0 | 0.8 | 0.75 | 0.7 | 0.7 | 0.87 | 0.8 |  |  |  |  |  |  |  |  |
| TCGA-FD-A6TI | 0.8054795 | 1 | 0.8 | 0.7 | 0.7 | 0.6 | 0.85 | 0.7 |  |  |  |  |  |  |  |  |
| TCGA-XF-AAMJ | 4.5753425 | 1 | 0.7 | 0.73 | 0.7 | 0.6 | 0.86 | 0.8 |  |  |  |  |  |  |  |  |
| TCGA-GU-A763 | 2.7315068 | 0 | 0.7 | 0.64 | 0.6 | 0.6 | 0.82 | 0.7 |  |  |  |  |  |  |  |  |
| TCGA-G2-A2EC | 1.9068493 | 1 | 0.8 | 0.74 | 0.7 | 0.6 | 0.86 | 0.8 |  |  |  |  |  |  |  |  |
| TCGA-FD-A3B7 | 0.3342466 | 1 | 0.8 | 0.76 | 0.7 | 0.7 | 0.88 | 0.8 |  |  |  |  |  |  |  |  |
| TCGA-FD-A5C0 | 1.5068493 | 1 | 0.8 | 0.71 | 0.7 | 0.6 | 0.84 | 0.7 |  |  |  |  |  |  |  |  |
| TCGA-FD-A5BS | 4.490411 | 0 | 0.8 | 0.78 | 0.7 | 0.7 | 0.91 | 0.8 |  |  |  |  |  |  |  |  |
| TCGA-CU-A0YN | 1.0767123 | 1 | 0.8 | 0.74 | 0.7 | 0.7 | 0.84 | 0.8 |  |  |  |  |  |  |  |  |
| TCGA-FD-A5BZ | 2.2876712 | 1 | 0.8 | 0.73 | 0.7 | 0.7 | 0.85 | 0.7 |  |  |  |  |  |  |  |  |
| TCGA-UY-A9PE | 0.5178082 | 0 | 0.7 | 0.68 | 0.6 | 0.6 | 0.82 | 0.7 |  |  |  |  |  |  |  |  |
| TCGA-GV-A40E | 0.7150685 | 1 | 0.7 | 0.69 | 0.7 | 0.6 | 0.83 | 0.7 |  |  |  |  |  |  |  |  |
| TCGA-BT-A2LD | 1.7068493 | 1 | 0.7 | 0.69 | 0.7 | 0.6 | 0.84 | 0.7 |  |  |  |  |  |  |  |  |
| TCGA-UY-A9PD | 1.4849315 | 0 | 0.7 | 0.68 | 0.6 | 0.6 | 0.83 | 0.7 |  |  |  |  |  |  |  |  |
| TCGA-KQ-A41N | 4.3945205 | 0 | 0.7 | 0.65 | 0.6 | 0.6 | 0.78 | 0.6 |  |  |  |  |  |  |  |  |
| TCGA-GD-A3OQ | 0.260274 | 0 | 0.7 | 0.7 | 0.7 | 0.6 | 0.83 | 0.7 |  |  |  |  |  |  |  |  |
| TCGA-BT-A20U | 1.2465753 | 1 | 0.7 | 0.69 | 0.7 | 0.6 | 0.83 | 0.7 |  |  |  |  |  |  |  |  |
| TCGA-XF-A9SW | 0.9917808 | 1 | 0.8 | 0.75 | 0.7 | 0.7 | 0.88 | 0.8 |  |  |  |  |  |  |  |  |
| TCGA-FD-A3B3 | 2.6684932 | 1 | 0.8 | 0.76 | 0.7 | 0.7 | 0.87 | 0.8 |  |  |  |  |  |  |  |  |
| TCGA-ZF-A9RC | 7.8575342 | 0 | 0.7 | 0.69 | 0.6 | 0.6 | 0.82 | 0.7 |  |  |  |  |  |  |  |  |
| TCGA-E7-A6MD | 0.3534247 | 0 | 0.8 | 0.74 | 0.7 | 0.7 | 0.86 | 0.8 |  |  |  |  |  |  |  |  |
| TCGA-E5-A4TZ | 1.2794521 | 1 | 0.7 | 0.65 | 0.6 | 0.6 | 0.8 | 0.7 |  |  |  |  |  |  |  |  |
| TCGA-ZF-A9R4 | 2.5232877 | 0 | 0.7 | 0.68 | 0.7 | 0.6 | 0.82 | 0.7 |  |  |  |  |  |  |  |  |
| TCGA-ZF-A9RE | 0.290411 | 1 | 0.7 | 0.66 | 0.6 | 0.6 | 0.81 | 0.7 |  |  |  |  |  |  |  |  |
| TCGA-BT-A20W | 0.6958904 | 1 | 0.7 | 0.73 | 0.7 | 0.7 | 0.84 | 0.8 |  |  |  |  |  |  |  |  |
| TCGA-GC-A6I3 | 0 | 0 | 0.7 | 0.7 | 0.7 | 0.6 | 0.86 | 0.7 |  |  |  |  |  |  |  |  |
| TCGA-4Z-AA80 | 0.0520548 | 1 | 0.7 | 0.67 | 0.6 | 0.6 | 0.79 | 0.7 |  |  |  |  |  |  |  |  |
| TCGA-G2-A3IE | 1.6767123 | 1 | 0.7 | 0.67 | 0.6 | 0.6 | 0.82 | 0.7 |  |  |  |  |  |  |  |  |
| TCGA-ZF-A9R0 | 1.8630137 | 1 | 0.7 | 0.7 | 0.7 | 0.6 | 0.83 | 0.7 |  |  |  |  |  |  |  |  |
| TCGA-FD-A43S | 1.2465753 | 0 | 0.8 | 0.77 | 0.7 | 0.7 | 0.9 | 0.8 |  |  |  |  |  |  |  |  |
| TCGA-XF-A8HG | 1.2794521 | 1 | 0.7 | 0.63 | 0.6 | 0.6 | 0.8 | 0.6 |  |  |  |  |  |  |  |  |
| TCGA-K4-A83P | 1.3561644 | 0 | 0.8 | 0.79 | 0.7 | 0.7 | 0.9 | 0.9 |  |  |  |  |  |  |  |  |
| TCGA-GD-A2C5 | 2.2246575 | 0 | 0.7 | 0.69 | 0.7 | 0.6 | 0.84 | 0.7 |  |  |  |  |  |  |  |  |
| TCGA-XF-AAMX | 0.5643836 | 1 | 0.7 | 0.67 | 0.6 | 0.6 | 0.81 | 0.7 |  |  |  |  |  |  |  |  |
| TCGA-DK-A1AE | 1.3452055 | 0 | 0.7 | 0.67 | 0.7 | 0.6 | 0.8 | 0.7 |  |  |  |  |  |  |  |  |
| TCGA-FJ-A3ZE | 0.8876712 | 1 | 0.6 | 0.61 | 0.6 | 0.5 | 0.77 | 0.6 |  |  |  |  |  |  |  |  |
| TCGA-DK-A2I1 | 1.4958904 | 0 | 0.8 | 0.71 | 0.7 | 0.7 | 0.86 | 0.8 |  |  |  |  |  |  |  |  |
| TCGA-DK-A6B0 | 6.3835616 | 0 | 0.7 | 0.66 | 0.6 | 0.6 | 0.83 | 0.7 |  |  |  |  |  |  |  |  |
| TCGA-FD-A5BR | 2.2273973 | 0 | 0.8 | 0.74 | 0.7 | 0.7 | 0.86 | 0.8 |  |  |  |  |  |  |  |  |
| TCGA-E7-A7PW | 1.139726 | 0 | 0.7 | 0.62 | 0.6 | 0.5 | 0.81 | 0.7 |  |  |  |  |  |  |  |  |
| TCGA-LT-A8JT | 1.7561644 | 0 | 0.7 | 0.69 | 0.6 | 0.6 | 0.83 | 0.7 |  |  |  |  |  |  |  |  |
| TCGA-FJ-A3Z9 | 1.0547945 | 1 | 0.7 | 0.62 | 0.6 | 0.6 | 0.79 | 0.7 |  |  |  |  |  |  |  |  |
| TCGA-DK-A1AC | 10.906849 | 0 | 0.7 | 0.71 | 0.7 | 0.7 | 0.84 | 0.7 |  |  |  |  |  |  |  |  |
| TCGA-C4-A0F1 | 0.2438356 | 0 | 0.7 | 0.69 | 0.7 | 0.6 | 0.83 | 0.7 |  |  |  |  |  |  |  |  |
| TCGA-G2-A2ES | 2.7506849 | 1 | 0.7 | 0.7 | 0.7 | 0.6 | 0.85 | 0.7 |  |  |  |  |  |  |  |  |
| TCGA-XF-AAN4 | 2.2547945 | 1 | 0.8 | 0.78 | 0.7 | 0.7 | 0.89 | 0.8 |  |  |  |  |  |  |  |  |
| TCGA-4Z-AA83 | 5.5452055 | 0 | 0.7 | 0.72 | 0.6 | 0.6 | 0.84 | 0.7 |  |  |  |  |  |  |  |  |
| TCGA-BT-A20V | 0.4219178 | 1 | 0.7 | 0.69 | 0.7 | 0.6 | 0.83 | 0.7 |  |  |  |  |  |  |  |  |
| TCGA-XF-A8HD | 8.1205479 | 0 | 0.7 | 0.71 | 0.7 | 0.6 | 0.85 | 0.8 |  |  |  |  |  |  |  |  |
| TCGA-GV-A3JX | 1.5917808 | 0 | 0.7 | 0.72 | 0.7 | 0.7 | 0.84 | 0.7 |  |  |  |  |  |  |  |  |
| TCGA-H4-A2HQ | 1.6164384 | 0 | 0.7 | 0.67 | 0.6 | 0.6 | 0.8 | 0.7 |  |  |  |  |  |  |  |  |
| TCGA-XF-A9SP | 1.2438356 | 1 | 0.8 | 0.72 | 0.7 | 0.7 | 0.87 | 0.7 |  |  |  |  |  |  |  |  |
| TCGA-DK-A3IK | 0.4 | 1 | 0.7 | 0.68 | 0.6 | 0.6 | 0.83 | 0.7 |  |  |  |  |  |  |  |  |
| TCGA-4Z-AA7N | 3.7452055 | 1 | 0.8 | 0.82 | 0.7 | 0.8 | 0.9 | 0.9 |  |  |  |  |  |  |  |  |
| TCGA-HQ-A2OE | 3.2164384 | 0 | 0.7 | 0.68 | 0.6 | 0.6 | 0.81 | 0.7 |  |  |  |  |  |  |  |  |
| TCGA-UY-A8OC | 0 | 1 | 0.7 | 0.7 | 0.7 | 0.6 | 0.84 | 0.7 |  |  |  |  |  |  |  |  |
| TCGA-GV-A3JV | 1.1890411 | 1 | 0.8 | 0.72 | 0.7 | 0.7 | 0.84 | 0.7 |  |  |  |  |  |  |  |  |
| TCGA-XF-A9T8 | 1.1452055 | 1 | 0.8 | 0.75 | 0.7 | 0.7 | 0.88 | 0.8 |  |  |  |  |  |  |  |  |
| TCGA-KQ-A41S | 0.0958904 | 0 | 0.8 | 0.72 | 0.7 | 0.6 | 0.87 | 0.7 |  |  |  |  |  |  |  |  |
| TCGA-CU-A3YL | 2.4821918 | 0 | 0.7 | 0.68 | 0.6 | 0.6 | 0.82 | 0.7 |  |  |  |  |  |  |  |  |
| TCGA-E7-A7XN | 1.1726027 | 0 | 0.8 | 0.72 | 0.7 | 0.7 | 0.86 | 0.7 |  |  |  |  |  |  |  |  |
| TCGA-DK-A2HX | 3.890411 | 1 | 0.8 | 0.73 | 0.7 | 0.7 | 0.86 | 0.7 |  |  |  |  |  |  |  |  |
| TCGA-CF-A8HY | 0.9452055 | 0 | 0.7 | 0.67 | 0.6 | 0.6 | 0.82 | 0.7 |  |  |  |  |  |  |  |  |
| TCGA-4Z-AA7W | 2.3013699 | 0 | 0.8 | 0.74 | 0.7 | 0.7 | 0.88 | 0.8 |  |  |  |  |  |  |  |  |
| TCGA-FD-A43U | 1.7424658 | 0 | 0.8 | 0.73 | 0.7 | 0.7 | 0.89 | 0.8 |  |  |  |  |  |  |  |  |
| TCGA-BT-A0S7 | 0.5479452 | 1 | 0.7 | 0.7 | 0.6 | 0.6 | 0.84 | 0.7 |  |  |  |  |  |  |  |  |
| TCGA-ZF-A9R3 | 2.6 | 1 | 0.7 | 0.71 | 0.7 | 0.6 | 0.83 | 0.7 |  |  |  |  |  |  |  |  |
| TCGA-4Z-AA89 | 2.8191781 | 0 | 0.7 | 0.66 | 0.6 | 0.6 | 0.82 | 0.7 |  |  |  |  |  |  |  |  |
| TCGA-GU-A42P | 0.909589 | 1 | 0.7 | 0.64 | 0.6 | 0.6 | 0.78 | 0.6 |  |  |  |  |  |  |  |  |
| TCGA-FD-A3NA | 5.0547945 | 0 | 0.8 | 0.72 | 0.7 | 0.6 | 0.85 | 0.7 |  |  |  |  |  |  |  |  |
| TCGA-BT-A20N | 2.1780822 | 1 | 0.7 | 0.66 | 0.6 | 0.6 | 0.8 | 0.7 |  |  |  |  |  |  |  |  |
| TCGA-XF-A8HC | 0.5479452 | 1 | 0.6 | 0.6 | 0.6 | 0.6 | 0.77 | 0.6 |  |  |  |  |  |  |  |  |
| TCGA-ZF-AA4W | 5.0136986 | 0 | 0.7 | 0.67 | 0.7 | 0.6 | 0.81 | 0.7 |  |  |  |  |  |  |  |  |
| TCGA-LC-A66R | 1.2767123 | 0 | 0.8 | 0.73 | 0.7 | 0.7 | 0.86 | 0.8 |  |  |  |  |  |  |  |  |
| TCGA-E7-A8O7 | 1.2767123 | 0 | 0.7 | 0.73 | 0.7 | 0.7 | 0.84 | 0.8 |  |  |  |  |  |  |  |  |
| TCGA-CF-A9FL | 1.5479452 | 1 | 0.7 | 0.69 | 0.6 | 0.6 | 0.83 | 0.7 |  |  |  |  |  |  |  |  |
| TCGA-ZF-A9RD | 1.1178082 | 1 | 0.8 | 0.74 | 0.7 | 0.7 | 0.87 | 0.8 |  |  |  |  |  |  |  |  |
| TCGA-G2-AA3D | 5.860274 | 0 | 0.7 | 0.67 | 0.6 | 0.6 | 0.82 | 0.7 |  |  |  |  |  |  |  |  |
| TCGA-DK-A2I2 | 0.6493151 | 1 | 0.8 | 0.72 | 0.7 | 0.7 | 0.86 | 0.7 |  |  |  |  |  |  |  |  |
| TCGA-FD-A62S | 1.1123288 | 1 | 0.8 | 0.76 | 0.7 | 0.7 | 0.87 | 0.8 |  |  |  |  |  |  |  |  |
| TCGA-MV-A51V | 1.1232877 | 0 | 0.7 | 0.68 | 0.6 | 0.6 | 0.82 | 0.7 |  |  |  |  |  |  |  |  |
| TCGA-E5-A2PC | 3.6328767 | 0 | 0.7 | 0.67 | 0.7 | 0.6 | 0.82 | 0.7 |  |  |  |  |  |  |  |  |
| TCGA-DK-A3X2 | 1.4986301 | 1 | 0.7 | 0.63 | 0.6 | 0.6 | 0.8 | 0.7 |  |  |  |  |  |  |  |  |
| TCGA-GD-A76B | 0.6136986 | 0 | 0.8 | 0.73 | 0.7 | 0.7 | 0.86 | 0.8 |  |  |  |  |  |  |  |  |
| TCGA-GC-A6I1 | 0 | 0 | 0.8 | 0.73 | 0.7 | 0.7 | 0.88 | 0.8 |  |  |  |  |  |  |  |  |
| TCGA-XF-A9SK | 1.3315068 | 1 | 0.8 | 0.73 | 0.7 | 0.6 | 0.86 | 0.7 |  |  |  |  |  |  |  |  |
| TCGA-DK-A1AD | 9.369863 | 0 | 0.7 | 0.69 | 0.7 | 0.6 | 0.83 | 0.7 |  |  |  |  |  |  |  |  |
| TCGA-K4-A5RI | 0.9753425 | 1 | 0.7 | 0.7 | 0.7 | 0.6 | 0.84 | 0.7 |  |  |  |  |  |  |  |  |
| TCGA-FD-A3SQ | 3.8986301 | 1 | 0.8 | 0.74 | 0.7 | 0.7 | 0.86 | 0.8 |  |  |  |  |  |  |  |  |
| TCGA-GV-A3QF | 1.690411 | 1 | 0.7 | 0.63 | 0.6 |  |  |  |  |  |  |  |  |  |  |  |
