# Supplementary material for: NXPH4 Used as a New Prognostic and Immunotherapeutic Marker for Muscle-Invasive Bladder Cancer
Source: J Oncol. 2022 Oct 4;2022:4271409. doi: 10.1155/2022/4271409 (PMC9553512; doi:10.1155/2022/4271409)
Supplement: Supplementary Materials — Figure s1: article roadmap of the whole research. Figure s2: (A) GSVA results heatmap of invasive bladder cancer in TCGA database (normal =19, tumor =404); Wayne diagram of differential pathways between clusters. (B) Wayne diagram in TCGA clusters (n = 65). (C) Wayne diagram in GEO clusters (n = 463). (D) Wayne diagram in TCGA clusters and GEO clusters (n = 6). Figure s3: (A) the 28 prognostic key pathway genes (P <0.01). Risk model for patients with muscle invasive bladder cancer (MIBC) based on 12 genes (SLC7A2, MST1R, CDK6, NXPH4, GRIK2, TRIB3, PBK, ABCA4, FBN2, SCG2, ELN, and INCENP). (B) LASSO regression with 10-fold crossvalidation was used to obtain 12 prognostic genes with an error within one standard error of the minimum (lambda.1se). (C) LASSO coefficient profiles of 28 key pathway genes. Supplement Table 1: clinical characteristics such as N, M, T, tumor grade, and stage, including age among the three groups (TCGA). Supplement Table 2: survival and prognosis information of three groups based on GEO. Supplement Table 3: 65 differential pathways were obtained from the molecular subtypes of TCGA queue. Supplement Table 4: 6 common differential pathways were obtained based on 65 TCGA, differential pathways, and 463 GEO, differential pathways. Supplement Table 5: 6 common differential pathways with prognosis. Supplement Table 6: the risk model based on the 12 prognostic genes in TCGA and GEO databases. Supplement Table 7: immune landscape between the high- and low-risk patients with muscle invasive bladder cancer (MIBC). Supplement Table 8: evaluation of immune response to CTLA4 and PD1 immunosuppressants in MIBC patients. [file 4271409.f1.zip › supplement table6.docx]

**Supplement Table6** The risk model based on the 12 prognostic genes in TCGA and GEO database.

| GSE21670_STAT3_KO_VS_WT_CD4_TCELL_TGFB_IL6_TREATED_DN | 128 gene-exptime | | | | |
| --- | --- | --- | --- | --- | --- |
| http://www.gsea-msigdb.org/gsea/msigdb/cards/GSE21670_STAT3_KO_VS_WT_CD4_TCELL_TGFB_IL6_TREATED_DN | ID | futime | fustat | riskScore | risk |
| PTPRJ | GSM340612 | 10.8275 | 0 | 3.418363 | low |
| RAP2B | GSM340613 | 1.0475 | 1 | 6.715079 | high |
| TSC22D3 | GSM340614 | 10.7475 | 0 | 11.85662 | high |
| AQP3 | GSM340618 | 0.085833 | 1 | 10.76884 | high |
| UTRN | GSM340620 | 1.389167 | 1 | 2.84265 | low |
| PLBD1 | GSM340623 | 0.260833 | 1 | 6.45223 | high |
| ME1 | GSM340628 | 10.04167 | 0 | 6.429943 | high |
| ID3 | GSM340630 | 5.525 | 1 | 9.925962 | high |
| SEPTIN9 | GSM340633 | 1.214167 | 1 | 5.597476 | low |
| ADA2 | GSM340634 | 9.6525 | 0 | 7.477625 | high |
| APBB1IP | GSM340636 | 10.1 | 0 | 3.785834 | low |
| TRAV8-3 | GSM340648 | 7.560833 | 0 | 4.510541 | low |
| TMEM63A | GSM340653 | 2.1525 | 1 | 9.169005 | high |
| SPARC | GSM340654 | 0.855833 | 1 | 6.518539 | high |
| ADAM28 | GSM340660 | 6.8025 | 0 | 6.110384 | low |
| TPP2 | GSM340665 | 0.725 | 1 | 9.100397 | high |
| RCSD1 | GSM340672 | 1.258333 | 1 | 3.888587 | low |
| RRM2B | GSM340673 | 0.935833 | 1 | 5.784309 | low |
| RCBTB2 | GSM340674 | 5.785833 | 0 | 13.26615 | high |
| CTDSP2 | GSM340677 | 5.435833 | 0 | 4.220878 | low |
| SUN2 | GSM340678 | 0.9975 | 1 | 8.923809 | high |
| TNFRSF1A | GSM340684 | 0.866667 | 1 | 3.21185 | low |
| ACP5 | GSM340693 | 4.705833 | 0 | 6.164815 | low |
| SERPING1 | GSM340694 | 4.625 | 0 | 4.898786 | low |
| ITGB7 | GSM340696 | 0.958333 | 1 | 6.009235 | low |
| LITAF | GSM340697 | 1.489167 | 1 | 4.345273 | low |
| SLC44A1 | GSM340700 | 1.283333 | 1 | 2.556332 | low |
| CD27 | GSM340701 | 4.035833 | 0 | 4.206758 | low |
| SORBS3 | GSM340702 | 0.375 | 1 | 9.090622 | high |
| IL36A | GSM340704 | 0.435833 | 1 | 5.329854 | low |
| RGS2 | GSM340705 | 3.8 | 0 | 4.988907 | low |
| CD52 | GSM340706 | 3.760833 | 0 | 4.7216 | low |
| ARRDC2 | GSM340709 | 0.494167 | 1 | 19.58431 | high |
| NLRP3 | GSM340712 | 0.541667 | 1 | 5.86944 | low |
| LBH | GSM340714 | 3.314167 | 0 | 8.302565 | high |
| CLEC4E | GSM340715 | 3.2475 | 0 | 4.770628 | low |
| FMNL1 | GSM340718 | 3.069167 | 0 | 5.151231 | low |
| GASK1B | GSM340720 | 2.341667 | 0 | 6.840765 | high |
| SIRPG | GSM340721 | 0.3275 | 1 | 8.146043 | high |
| GCNT4 | GSM340723 | 1.258333 | 1 | 12.98999 | high |
| PARP4 | GSM340725 | 2.8525 | 0 | 7.145596 | high |
| ASAH1 | GSM340727 | 0.9225 | 1 | 8.08555 | high |
| ITM2B | GSM340728 | 0.480833 | 1 | 16.40486 | high |
| IQSEC1 | GSM340729 | 2.755833 | 0 | 10.03445 | high |
| PMFBP1 | GSM340731 | 0.769167 | 1 | 13.37652 | high |
| STAB1 | GSM340732 | 0.591667 | 1 | 3.591418 | low |
| MITF | GSM340733 | 1.4275 | 1 | 4.076412 | low |
| PLD3 | GSM340745 | 0.1775 | 1 | 6.470762 | high |
| RNASE1 | GSM340746 | 1.855833 | 0 | 4.658311 | low |
| MSN | GSM340747 | 0.5725 | 1 | 5.197739 | low |
| SDCBP | GSM340757 | 6.608333 | 0 | 6.302201 | low |
| TTYH2 | GSM340758 | 1.289167 | 1 | 6.645016 | high |
| ADAMDEC1 | GSM340759 | 2.2025 | 1 | 12.99675 | high |
| MSR1 | GSM340760 | 1.105833 | 1 | 4.380874 | low |
| RASGRP2 | GSM340761 | 0.533333 | 1 | 9.30217 | high |
| MADD | GSM340762 | 0.889167 | 1 | 15.65291 | high |
| GCLC | GSM340763 | 1.510833 | 0 | 6.078779 | low |
| LFNG | GSM340764 | 1.280833 | 0 | 4.874173 | low |
| SSBP2 | GSM340765 | 0.983333 | 0 | 6.423538 | high |
| FAM78A | GSM340766 | 0.905833 | 0 | 7.872456 | high |
| NCAM1 | GSM340767 | 0.835833 | 0 | 8.246406 | high |
| SMC4 | GSM340769 | 0.441667 | 0 | 11.16174 | high |
| IGFLR1 | GSM786491 | 8.648867 | 0 | 10.31172 | high |
| APOBEC3A | GSM786492 | 1.097878 | 1 | 8.956393 | high |
| AVPI1 | GSM786493 | 1.648186 | 0 | 9.956449 | high |
| ARRB2 | GSM786494 | 1.368925 | 1 | 9.562249 | high |
| ELF1 | GSM786495 | 1.09514 | 0 | 5.177777 | low |
| CTSS | GSM786496 | 0.36961 | 1 | 5.233653 | low |
| NMT2 | GSM786497 | 7.321013 | 0 | 7.060399 | high |
| PIP4P2 | GSM786499 | 9.037642 | 1 | 4.449831 | low |
| CREBL2 | GSM786500 | 14.6256 | 0 | 6.258412 | low |
| CLEC6A | GSM786503 | 0.032854 | 0 | 14.2325 | high |
| CD9 | GSM786504 | 0.903491 | 1 | 3.099817 | low |
| CITED2 | GSM786506 | 0.279261 | 1 | 26.97071 | high |
| CD4 | GSM786508 | 0.977413 | 1 | 9.841191 | high |
| CCPG1 | GSM786509 | 2.609172 | 0 | 4.342535 | low |
| SORL1 | GSM786510 | 0.684463 | 1 | 3.848023 | low |
| GLG1 | GSM786511 | 2.056126 | 0 | 6.512109 | high |
| RGCC | GSM786512 | 1.108829 | 1 | 6.666744 | high |
| GABBR1 | GSM786513 | 0.090349 | 0 | 4.823078 | low |
| TPP1 | GSM786514 | 0.610541 | 1 | 5.366739 | low |
| DGKZ | GSM786515 | 0.273785 | 0 | 12.58152 | high |
| ZFP36L2 | GSM786516 | 1.201917 | 1 | 9.079661 | high |
| MPP1 | GSM786518 | 4.859685 | 1 | 4.122326 | low |
| ZNF831 | GSM786519 | 0.413416 | 1 | 11.85111 | high |
| AHNAK | GSM786520 | 0.774812 | 1 | 4.835589 | low |
| TGFBI | GSM786521 | 1.054073 | 1 | 5.660118 | low |
| CREG1 | GSM786522 | 6.045174 | 0 | 3.775431 | low |
| LYZ | GSM786523 | 7.509925 | 0 | 3.26962 | low |
| GPNMB | GSM786525 | 0.443532 | 0 | 4.209366 | low |
| PRCP | GSM786526 | 0.281999 | 0 | 8.227544 | high |
| PSAP | GSM786528 | 0.235455 | 0 | 12.00258 | high |
| MARCHF1 | GSM786530 | 0.826831 | 1 | 9.993249 | high |
| CD40LG | GSM786531 | 8.55305 | 0 | 5.682038 | low |
| VSIR | GSM786532 | 0.276523 | 1 | 4.450149 | low |
| RNASE6 | GSM786533 | 9.177275 | 0 | 6.422211 | high |
| SIGLEC1 | GSM786535 | 0.465435 | 0 | 8.422592 | high |
| RHBDD2 | GSM786536 | 0.637919 | 1 | 6.323831 | low |
| FGL2 | GSM786537 | 1.418207 | 1 | 3.732636 | low |
| CXCL10 | GSM786539 | 10.06434 | 0 | 3.938407 | low |
| DIAPH1 | GSM786540 | 3.723477 | 0 | 5.465202 | low |
| ARRB1 | GSM786541 | 7.638603 | 0 | 5.251972 | low |
| PDK4 | GSM786542 | 0.36961 | 1 | 4.30284 | low |
| ITGB2 | GSM786543 | 6.620123 | 0 | 11.1456 | high |
| CEMIP2 | GSM786544 | 1.138946 | 1 | 5.646082 | low |
| KLHL3 | GSM786545 | 5.440109 | 0 | 6.113682 | low |
| NPL | GSM786546 | 0.065708 | 0 | 14.65302 | high |
| MDS2 | GSM786547 | 1.122519 | 1 | 11.47125 | high |
| LAIR1 | GSM786549 | 9.286792 | 1 | 6.725276 | high |
| MCTP1 | GSM786550 | 0.818617 | 1 | 11.09325 | high |
| YPEL4 | GSM786551 | 8.547567 | 0 | 20.85279 | high |
| ZFYVE16 | GSM786552 | 0.648871 | 0 | 18.0673 | high |
| ARHGAP21 | GSM786553 | 8.490075 | 0 | 9.428344 | high |
| DPEP2 | GSM786554 | 2.718686 | 0 | 4.571759 | low |
| SMIM14 | GSM786556 | 6.63655 | 0 | 4.624757 | low |
| TLK1 | GSM786557 | 8.65435 | 0 | 4.717561 | low |
| C5 | GSM786558 | 8.41615 | 0 | 4.713146 | low |
| GPX4 | GSM786559 | 7.622177 | 0 | 10.13463 | high |
| CPED1 | GSM786560 | 8.180698 | 0 | 4.802066 | low |
| TIMD4 | GSM786561 | 1.256673 | 0 | 6.935732 | high |
| BMPR2 | GSM786562 | 0.553046 | 0 | 4.556054 | low |
| ADGRE5 | GSM786563 | 1.059548 | 1 | 6.209038 | low |
| FUCA1 | GSM786564 | 5.604381 | 0 | 7.795083 | high |
| NEK7 | GSM786565 | 3.572895 | 0 | 5.423513 | low |
| PAQR8 | GSM786568 | 7.060918 | 0 | 6.916001 | high |
| GNS | GSM786570 | 1.467488 | 0 | 13.13456 | high |
| SLC16A5 | GSM786571 | 7.162218 | 0 | 5.068987 | low |
| TIMP2 | GSM786572 | 7.425052 | 0 | 11.08386 | high |
| TXLNB | GSM786573 | 6.759753 | 0 | 4.435742 | low |
| SLC37A2 | GSM786576 | 0.971937 | 1 | 5.182828 | low |
| RTN1 | GSM786577 | 2.737851 | 1 | 6.443857 | high |
| RIPOR2 | GSM786579 | 4.29295 | 1 | 10.46901 | high |
| EPHX1 | GSM786580 | 3.852156 | 0 | 3.614897 | low |
| FAM111A | GSM786581 | 5.98768 | 0 | 11.21527 | high |
| ITGA5 | GSM786582 | 0.476386 | 1 | 4.666709 | low |
| WNT7A | GSM786583 | 5.379877 | 0 | 5.478479 | low |
| MANBA | GSM814052 | 9.191781 | 0 | 8.052977 | high |
| LRP1 | GSM814056 | 3.03 | 1 | 3.560755 | low |
| NCEH1 | GSM814058 | 5.326027 | 0 | 5.24871 | low |
| ATP6AP2 | GSM814060 | 0.449315 | 1 | 14.55065 | high |
| SLC1A3 | GSM814061 | 1.068493 | 0 | 4.093716 | low |
| ASRGL1 | GSM814064 | 6.331507 | 0 | 6.599701 | high |
| GRN | GSM814065 | 8.649315 | 0 | 4.780196 | low |
| BNIP3L | GSM814066 | 5.520548 | 0 | 7.608276 | high |
| DHRS9 | GSM814068 | 8.032877 | 0 | 10.36693 | high |
| CD5 | GSM814069 | 6.169863 | 0 | 5.152588 | low |
| ZYX | GSM814070 | 2.131507 | 0 | 6.882148 | high |
| CPVL | GSM814074 | 5.249315 | 0 | 5.334428 | low |
| SLC12A6 | GSM814076 | 1.493151 | 1 | 12.41391 | high |
| IL13RA1 | GSM814084 | 5.558904 | 0 | 4.998528 | low |
| CCM2 | GSM814085 | 8.142466 | 0 | 5.621179 | low |
| CCDC88A | GSM814086 | 5.70411 | 0 | 2.460561 | low |
| LRRK2 | GSM814087 | 6.827397 | 1 | 11.31585 | high |
| IPCEF1 | GSM814090 | 6.536986 | 0 | 5.897143 | low |
| CYBRD1 | GSM814091 | 2.978082 | 0 | 12.88344 | high |
| GPAT3 | GSM814092 | 2.210959 | 1 | 8.617456 | high |
| ANXA11 | GSM814094 | 2.457534 | 1 | 25.00068 | high |
| ACP3 | GSM814095 | 7.117808 | 0 | 10.96833 | high |
| DAPK1 | GSM814098 | 6.156164 | 0 | 13.15355 | high |
| ALDH1A1 | GSM814100 | 7.383562 | 0 | 20.87973 | high |
| ATP10D | GSM814102 | 6.016438 | 0 | 3.830865 | low |
| SIRPB2 | GSM814108 | 1.758904 | 0 | 12.75061 | high |
| ARHGAP9 | GSM814109 | 5.791781 | 0 | 6.917899 | high |
| CLEC2D | GSM814110 | 4.528767 | 0 | 10.38941 | high |
| RASSF3 | GSM814112 | 7.076712 | 0 | 4.038639 | low |
| CA11 | GSM814118 | 4.526027 | 0 | 4.827917 | low |
| STK38 | GSM814119 | 7.345205 | 0 | 10.47903 | high |
| TEP1 | GSM814122 | 6.90411 | 0 | 6.799207 | high |
| CA2 | GSM814126 | 7.20274 | 0 | 11.3072 | high |
| CAMSAP2 | GSM814132 | 0.555708 | 0 | 7.037206 | high |
| DUSP10 | GSM814133 | 4.112329 | 0 | 8.2494 | high |
| TBL1X | GSM814134 | 2.99726 | 0 | 5.098655 | low |
| NAB2 | GSM814135 | 6.046575 | 0 | 6.087911 | low |
| NFE2L2 | GSM814136 | 1.035616 | 0 | 4.169152 | low |
| ESYT1 | GSM814138 | 0.515068 | 1 | 1.93744 | low |
| ACTN1 | GSM814142 | 0.627397 | 1 | 4.61385 | low |
| RGS10 | GSM814149 | 0.841096 | 0 | 2.383997 | low |
| WIPF1 | GSM814150 | 3.29863 | 0 | 3.433935 | low |
| CD180 | GSM814153 | 3.778082 | 0 | 2.430792 | low |
| MNDA | GSM814155 | 5.983562 | 0 | 2.039045 | low |
| ANXA4 | GSM814156 | 6.553425 | 0 | 3.172528 | low |
| ZMIZ1 | GSM814160 | 5.923288 | 0 | 4.279949 | low |
| METTL7A | GSM814162 | 5.791781 | 0 | 9.239392 | high |
| SLC17A5 | GSM814163 | 5.805479 | 0 | 22.01046 | high |
| PDGFC | GSM814167 | 3.591781 | 0 | 5.325679 | low |
| MRAS | GSM814172 | 1.717808 | 1 | 4.993304 | low |
| KIAA0930 | GSM814173 | 2.49863 | 0 | 5.117058 | low |
| MYADM | GSM814174 | 1.224658 | 0 | 9.820351 | high |
| XCR1 | GSM814175 | 2.8 | 0 | 11.48406 | high |
| TLR4 | GSM814176 | 5.827397 | 0 | 22.83856 | high |
| SPTBN1 | GSM814179 | 1.473973 | 1 | 4.098947 | low |
| SLAMF7 | GSM814180 | 1.167123 | 0 | 6.412436 | high |
| SLC36A1 | GSM814182 | 0.572603 | 1 | 4.912016 | low |
| DSC1 | GSM814185 | 2.569863 | 0 | 4.689912 | low |
| PDE7A | GSM814189 | 4.79726 | 0 | 3.405997 | low |
| CTSB | GSM814190 | 4.712329 | 0 | 6.592403 | high |
| TMBIM1 | GSM814191 | 5.386301 | 0 | 5.519016 | low |
| CLEC4D | GSM814192 | 5.367123 | 0 | 2.771146 | low |
| TXNIP | GSM814193 | 4.835616 | 0 | 4.259543 | low |
| IL16 | GSM814194 | 4.534247 | 0 | 2.423173 | low |
|  | GSM814195 | 4.835616 | 0 | 10.35953 | high |
|  | GSM814196 | 4.224658 | 0 | 10.01724 | high |
|  | GSM814197 | 3.027397 | 0 | 5.019788 | low |
|  | GSM814200 | 1.142466 | 0 | 23.60776 | high |
|  | GSM814201 | 5.087671 | 0 | 4.928682 | low |
|  | GSM814202 | 0.531507 | 1 | 9.856373 | high |
|  | GSM814203 | 5.443836 | 0 | 4.073759 | low |
|  | GSM814204 | 2.016438 | 1 | 9.967828 | high |
|  | GSM814205 | 5.323288 | 0 | 8.434997 | high |
|  | GSM814208 | 5.331507 | 0 | 5.128239 | low |
|  | GSM814210 | 5.142466 | 0 | 2.439719 | low |
|  | GSM814211 | 2.380822 | 0 | 6.068339 | low |
|  | GSM814212 | 5.210959 | 0 | 2.592253 | low |
|  | GSM814213 | 1.690411 | 0 | 2.656451 | low |
|  | GSM814215 | 4.723288 | 0 | 4.467359 | low |
|  | GSM814217 | 4.967123 | 0 | 3.479813 | low |
|  | GSM814218 | 2.89863 | 1 | 11.94759 | high |
|  | GSM814221 | 0.268493 | 0 | 4.288146 | low |
|  | GSM814224 | 4.156164 | 0 | 7.972414 | high |
|  | GSM814225 | 1.117808 | 1 | 2.822085 | low |
|  | GSM814226 | 1.764384 | 0 | 7.072325 | high |
|  | GSM814228 | 4.528767 | 0 | 12.84688 | high |
|  | GSM814229 | 4.8 | 0 | 6.098324 | low |
|  | GSM814230 | 4.238356 | 0 | 19.53614 | high |
|  | GSM814231 | 3.934247 | 0 | 9.03815 | high |
|  | GSM814234 | 4.586301 | 0 | 8.043271 | high |
|  | GSM814238 | 0.306849 | 1 | 6.784371 | high |
|  | GSM814240 | 4.684932 | 0 | 8.656431 | high |
|  | GSM814241 | 0.539726 | 1 | 4.086237 | low |
|  | GSM814246 | 4.476712 | 0 | 2.553749 | low |
|  | GSM814248 | 3.421918 | 0 | 15.69171 | high |
|  | GSM814250 | 1.309589 | 0 | 6.05462 | low |
|  | GSM814251 | 3.391781 | 0 | 9.574188 | high |
|  | GSM814252 | 3.087671 | 0 | 4.759056 | low |
|  | GSM814253 | 3.147945 | 0 | 3.694236 | low |
|  | GSM814254 | 2.742466 | 0 | 4.841871 | low |
|  | GSM814256 | 3.772603 | 0 | 8.641207 | high |
|  | GSM814261 | 3.509589 | 0 | 11.00537 | high |
|  | GSM814263 | 2.252055 | 0 | 5.817878 | low |
|  | GSM814264 | 3.728767 | 0 | 4.859199 | low |
|  | GSM814266 | 3.438356 | 0 | 10.1 | high |
|  | GSM814267 | 3.671233 | 0 | 13.79089 | high |
|  | GSM814269 | 1.29863 | 1 | 4.996054 | low |
|  | GSM814271 | 3.40274 | 0 | 7.564249 | high |
|  | GSM814273 | 2.693151 | 0 | 5.986759 | low |
|  | GSM814274 | 3.326027 | 0 | 4.399015 | low |
|  | GSM814275 | 3.183562 | 0 | 7.722198 | high |
|  | GSM814276 | 3.380822 | 0 | 23.25615 | high |
|  | GSM814281 | 2.109589 | 0 | 3.998678 | low |
|  | GSM814282 | 2.627397 | 0 | 8.669203 | high |
|  | GSM814284 | 1.808219 | 0 | 5.270358 | low |
|  | GSM814287 | 2.808219 | 0 | 8.876787 | high |
|  | GSM814288 | 2.063014 | 0 | 6.026005 | low |
|  | GSM814289 | 0.684932 | 1 | 6.666542 | high |
|  | GSM814290 | 0.989041 | 0 | 11.62517 | high |
|  | GSM814291 | 1.520548 | 1 | 10.61046 | high |
|  | GSM814295 | 2.838356 | 0 | 7.604148 | high |
|  | GSM814297 | 2.610959 | 0 | 5.307518 | low |
|  | GSM814298 | 2.531507 | 0 | 6.147256 | low |
|  | GSM814300 | 0.819178 | 1 | 8.146493 | high |
|  | GSM814301 | 2.778082 | 0 | 5.175245 | low |
|  | GSM814303 | 2.567123 | 0 | 8.294916 | high |
|  | GSM814304 | 2.191781 | 0 | 4.120719 | low |
|  | GSM814305 | 2.493151 | 0 | 5.065621 | low |
|  | GSM814306 | 2.457534 | 0 | 2.274801 | low |
|  | GSM814307 | 2.205479 | 0 | 6.419972 | high |
|  | GSM814308 | 2.336986 | 0 | 5.950264 | low |
|  | GSM814309 | 2.221918 | 0 | 11.43388 | high |
|  | GSM814312 | 2.120548 | 0 | 7.907959 | high |
|  | GSM814313 | 1.79726 | 0 | 5.328046 | low |
|  | GSM814317 | 1.750685 | 0 | 9.276031 | high |
|  | GSM814318 | 1.975342 | 0 | 3.631706 | low |
|  | GSM814321 | 1.983562 | 0 | 9.999568 | high |
|  | GSM814322 | 1.257534 | 1 | 9.012881 | high |
|  | GSM814323 | 1.70137 | 0 | 6.988534 | high |
|  | GSM814324 | 1.712329 | 0 | 6.117504 | low |
|  | GSM814325 | 1.117808 | 1 | 3.679647 | low |
|  | GSM814326 | 1.136986 | 0 | 17.1408 | high |
|  | GSM814328 | 1.657534 | 0 | 5.566239 | low |
|  | GSM814330 | 1.621918 | 0 | 7.696061 | high |
|  | GSM814331 | 1.473973 | 0 | 5.274793 | low |
|  | GSM814333 | 1.473973 | 0 | 24.98968 | high |
|  | GSM814334 | 0.646119 | 0 | 10.51702 | high |
|  | GSM814336 | 1.353425 | 0 | 5.828702 | low |
|  | GSM814337 | 1.438356 | 0 | 9.67318 | high |
|  | GSM814338 | 1.079452 | 0 | 7.584465 | high |
|  | GSM814342 | 0.715068 | 0 | 7.672042 | high |
|  | GSM814345 | 0.547945 | 0 | 3.265119 | low |
|  | GSM814347 | 0.830137 | 0 | 2.349955 | low |
|  | GSM814348 | 0.764384 | 0 | 6.262754 | low |
|  | GSM814349 | 0.041667 | 0 | 5.094676 | low |
|  | GSM814351 | 0.465753 | 0 | 3.346199 | low |
|  | GSM814353 | 0.912329 | 1 | 9.327886 | high |
|  | GSM814354 | 1.421918 | 0 | 6.267703 | low |
|  | GSM814355 | 0.564384 | 0 | 7.559927 | high |
|  | GSM814356 | 1.364384 | 0 | 4.489337 | low |
|  | GSM814357 | 1.624658 | 0 | 7.635087 | high |
|  | TCGA-2F-A9KO | 2.010959 | 1 | 5.541795 | low |
|  | TCGA-2F-A9KP | 0.99726 | 1 | 4.154542 | low |
|  | TCGA-2F-A9KQ | 7.906849 | 0 | 3.065501 | low |
|  | TCGA-2F-A9KR | 8.720548 | 1 | 6.255364 | low |
|  | TCGA-2F-A9KT | 6.443836 | 0 | 6.242282 | low |
|  | TCGA-2F-A9KW | 0.69589 | 1 | 9.077719 | high |
|  | TCGA-4Z-AA7M | 1.356164 | 0 | 3.760835 | low |
|  | TCGA-4Z-AA7N | 3.745205 | 1 | 3.64645 | low |
|  | TCGA-4Z-AA7O | 1.40274 | 0 | 9.008398 | high |
|  | TCGA-4Z-AA7Q | 1.39726 | 1 | 5.469798 | low |
|  | TCGA-4Z-AA7R | 1.430137 | 1 | 14.25292 | high |
|  | TCGA-4Z-AA7S | 2.915068 | 1 | 3.418598 | low |
|  | TCGA-4Z-AA7W | 2.30137 | 0 | 3.132437 | low |
|  | TCGA-4Z-AA7Y | 4.169863 | 0 | 10.4037 | high |
|  | TCGA-4Z-AA80 | 0.052055 | 1 | 6.78222 | high |
|  | TCGA-4Z-AA81 | 3.479452 | 1 | 8.669888 | high |
|  | TCGA-4Z-AA82 | 4.263014 | 1 | 5.944398 | low |
|  | TCGA-4Z-AA83 | 5.545205 | 0 | 7.28195 | high |
|  | TCGA-4Z-AA84 | 1.260274 | 0 | 9.460307 | high |
|  | TCGA-4Z-AA86 | 0.852055 | 1 | 19.15301 | high |
|  | TCGA-4Z-AA87 | 3.983562 | 0 | 5.223796 | low |
|  | TCGA-4Z-AA89 | 2.819178 | 0 | 10.15028 | high |
|  | TCGA-5N-A9KI | 0.208219 | 1 | 9.853354 | high |
|  | TCGA-5N-A9KM | 1.452055 | 1 | 8.262629 | high |
|  | TCGA-BL-A13I | 0.610959 | 1 | 9.929502 | high |
|  | TCGA-BL-A13J | 0.221918 | 1 | 15.76392 | high |
|  | TCGA-BL-A3JM | 0.561644 | 1 | 4.230659 | low |
|  | TCGA-BL-A5ZZ | 1.032877 | 0 | 5.156195 | low |
|  | TCGA-BT-A0S7 | 0.547945 | 1 | 5.16466 | low |
|  | TCGA-BT-A0YX | 1.09589 | 1 | 15.53704 | high |
|  | TCGA-BT-A20J | 1.586301 | 1 | 9.829021 | high |
|  | TCGA-BT-A20N | 2.178082 | 1 | 6.005659 | low |
|  | TCGA-BT-A20O | 1.013699 | 1 | 3.322326 | low |
|  | TCGA-BT-A20P | 1.490411 | 1 | 4.016715 | low |
|  | TCGA-BT-A20Q | 1.624658 | 1 | 7.00318 | high |
|  | TCGA-BT-A20R | 0.421918 | 1 | 24.73229 | high |
|  | TCGA-BT-A20T | 1.241096 | 1 | 6.873079 | high |
|  | TCGA-BT-A20U | 1.246575 | 1 | 5.489561 | low |
|  | TCGA-BT-A20V | 0.421918 | 1 | 5.393047 | low |
|  | TCGA-BT-A20W | 0.69589 | 1 | 5.325287 | low |
|  | TCGA-BT-A20X | 0.687671 | 1 | 3.701208 | low |
|  | TCGA-BT-A2LA | 1.430137 | 0 | 5.797577 | low |
|  | TCGA-BT-A2LB | 1.347945 | 1 | 2.171455 | low |
|  | TCGA-BT-A2LD | 1.706849 | 1 | 3.220018 | low |
|  | TCGA-BT-A3PH | 0.389041 | 1 | 12.99058 | high |
|  | TCGA-BT-A3PJ | 2.161644 | 0 | 10.18094 | high |
|  | TCGA-BT-A3PK | 0.830137 | 1 | 3.722124 | low |
|  | TCGA-BT-A42C | 2.391781 | 0 | 10.29515 | high |
|  | TCGA-BT-A42E | 3.035616 | 0 | 6.963782 | high |
|  | TCGA-BT-A42F | 2.367123 | 0 | 5.270306 | low |
|  | TCGA-C4-A0EZ | 0.747945 | 1 | 8.248489 | high |
|  | TCGA-C4-A0F0 | 0.161644 | 0 | 6.756984 | high |
|  | TCGA-C4-A0F1 | 0.243836 | 0 | 6.569916 | high |
|  | TCGA-C4-A0F6 | 1.917808 | 0 |  |  |
|  | TCGA-C4-A0F7 | 0.169863 | 1 |  |  |
|  | TCGA-CF-A1HR | 1.065753 | 0 |  |  |
|  | TCGA-CF-A1HS | 1.046575 | 0 |  |  |
|  | TCGA-CF-A27C | 1.164384 | 0 |  |  |
|  | TCGA-CF-A3MF | 1.049315 | 0 |  |  |
|  | TCGA-CF-A3MG | 1.010959 | 0 |  |  |
|  | TCGA-CF-A3MH | 1.090411 | 0 |  |  |
|  | TCGA-CF-A3MI | 1.013699 | 0 |  |  |
|  | TCGA-CF-A47S | 0.912329 | 0 |  |  |
|  | TCGA-CF-A47T | 1.054795 | 1 |  |  |
|  | TCGA-CF-A47V | 1.038356 | 0 |  |  |
|  | TCGA-CF-A47W | 1.008219 | 0 |  |  |
|  | TCGA-CF-A47X | 1.052055 | 0 |  |  |
|  | TCGA-CF-A47Y | 1.021918 | 0 |  |  |
|  | TCGA-CF-A5U8 | 1.093151 | 0 |  |  |
|  | TCGA-CF-A5UA | 1 | 0 |  |  |
|  | TCGA-CF-A7I0 | 1.008219 | 0 |  |  |
|  | TCGA-CF-A8HX | 0.945205 | 0 |  |  |
|  | TCGA-CF-A8HY | 0.945205 | 0 |  |  |
|  | TCGA-CF-A9FF | 0.989041 | 0 |  |  |
|  | TCGA-CF-A9FH | 0.003 | 0 |  |  |
|  | TCGA-CF-A9FL | 1.547945 | 1 |  |  |
|  | TCGA-CU-A0YN | 1.076712 | 1 |  |  |
|  | TCGA-CU-A0YO | 0.408219 | 1 |  |  |
|  | TCGA-CU-A0YR | 1.260274 | 1 |  |  |
|  | TCGA-CU-A3KJ | 1.539726 | 0 |  |  |
|  | TCGA-CU-A3QU | 0.432877 | 0 |  |  |
|  | TCGA-CU-A3YL | 2.482192 | 0 |  |  |
|  | TCGA-CU-A5W6 | 0.153425 | 1 |  |  |
|  | TCGA-CU-A72E | 1.131507 | 1 |  |  |
|  | TCGA-DK-A1A3 | 1.821918 | 1 |  |  |
|  | TCGA-DK-A1A5 | 0.178082 | 1 |  |  |
|  | TCGA-DK-A1A6 | 5.534247 | 0 |  |  |
|  | TCGA-DK-A1A7 | 1.534247 | 0 |  |  |
|  | TCGA-DK-A1AA | 1.583562 | 0 |  |  |
|  | TCGA-DK-A1AB | 1.391781 | 1 |  |  |
|  | TCGA-DK-A1AC | 10.90685 | 0 |  |  |
|  | TCGA-DK-A1AD | 9.369863 | 0 |  |  |
|  | TCGA-DK-A1AE | 1.345205 | 0 |  |  |
|  | TCGA-DK-A1AF | 1.468493 | 0 |  |  |
|  | TCGA-DK-A1AG | 1.30137 | 0 |  |  |
|  | TCGA-DK-A2HX | 3.890411 | 1 |  |  |
|  | TCGA-DK-A2I1 | 1.49589 | 0 |  |  |
|  | TCGA-DK-A2I2 | 0.649315 | 1 |  |  |
|  | TCGA-DK-A2I4 | 10.50685 | 0 |  |  |
|  | TCGA-DK-A2I6 | 7.276712 | 0 |  |  |
|  | TCGA-DK-A3IK | 0.4 | 1 |  |  |
|  | TCGA-DK-A3IL | 1.131507 | 1 |  |  |
|  | TCGA-DK-A3IM | 0.679452 | 1 |  |  |
|  | TCGA-DK-A3IN | 0.684932 | 1 |  |  |
|  | TCGA-DK-A3IQ | 1.476712 | 1 |  |  |
|  | TCGA-DK-A3IS | 4.189041 | 0 |  |  |
|  | TCGA-DK-A3IT | 1.775342 | 0 |  |  |
|  | TCGA-DK-A3IU | 1.934247 | 1 |  |  |
|  | TCGA-DK-A3IV | 0.805479 | 1 |  |  |
|  | TCGA-DK-A3WW | 1.734247 | 0 |  |  |
|  | TCGA-DK-A3WX | 0.879452 | 1 |  |  |
|  | TCGA-DK-A3WY | 13.60822 | 0 |  |  |
|  | TCGA-DK-A3X1 | 5.50411 | 0 |  |  |
|  | TCGA-DK-A3X2 | 1.49863 | 1 |  |  |
|  | TCGA-DK-A6AV | 5.347945 | 0 |  |  |
|  | TCGA-DK-A6AW | 4.441096 | 0 |  |  |
|  | TCGA-DK-A6B0 | 6.383562 | 0 |  |  |
|  | TCGA-DK-A6B1 | 5.613699 | 0 |  |  |
|  | TCGA-DK-A6B2 | 1.306849 | 0 |  |  |
|  | TCGA-DK-A6B5 | 4.224658 | 0 |  |  |
|  | TCGA-DK-A6B6 | 3.054795 | 0 |  |  |
|  | TCGA-DK-AA6L | 3.186301 | 1 |  |  |
|  | TCGA-DK-AA6M | 4.334247 | 0 |  |  |
|  | TCGA-DK-AA6P | 1.252055 | 0 |  |  |
|  | TCGA-DK-AA6Q | 1.131507 | 1 |  |  |
|  | TCGA-DK-AA6R | 13.81096 | 0 |  |  |
|  | TCGA-DK-AA6S | 13.83562 | 0 |  |  |
|  | TCGA-DK-AA6T | 1.567123 | 0 |  |  |
|  | TCGA-DK-AA6U | 1.583562 | 0 |  |  |
|  | TCGA-DK-AA6W | 1.136986 | 1 |  |  |
|  | TCGA-DK-AA6X | 1.279452 | 0 |  |  |
|  | TCGA-DK-AA71 | 1.136986 | 0 |  |  |
|  | TCGA-DK-AA74 | 4.679452 | 0 |  |  |
|  | TCGA-DK-AA75 | 0.931507 | 1 |  |  |
|  | TCGA-DK-AA76 | 1.00274 | 0 |  |  |
|  | TCGA-DK-AA77 | 1.693151 | 0 |  |  |
|  | TCGA-E5-A2PC | 3.632877 | 0 |  |  |
|  | TCGA-E5-A4TZ | 1.279452 | 1 |  |  |
|  | TCGA-E5-A4U1 | 3.235616 | 0 |  |  |
|  | TCGA-E7-A3X6 | 2.476712 | 1 |  |  |
|  | TCGA-E7-A3Y1 | 0.446575 | 0 |  |  |
|  | TCGA-E7-A4IJ | 1.846575 | 1 |  |  |
|  | TCGA-E7-A4XJ | 0.186301 | 1 |  |  |
|  | TCGA-E7-A519 | 1.391781 | 0 |  |  |
|  | TCGA-E7-A541 | 2.131507 | 1 |  |  |
|  | TCGA-E7-A5KE | 0.046575 | 0 |  |  |
|  | TCGA-E7-A5KF | 0.054795 | 0 |  |  |
|  | TCGA-E7-A677 | 2.246575 | 0 |  |  |
|  | TCGA-E7-A678 | 2.186301 | 0 |  |  |
|  | TCGA-E7-A6MD | 0.353425 | 0 |  |  |
|  | TCGA-E7-A6ME | 2.00274 | 0 |  |  |
|  | TCGA-E7-A6MF | 2.054795 | 0 |  |  |
|  | TCGA-E7-A7DU | 0.076712 | 0 |  |  |
|  | TCGA-E7-A7DV | 0.10137 | 0 |  |  |
|  | TCGA-E7-A7PW | 1.139726 | 0 |  |  |
|  | TCGA-E7-A7XN | 1.172603 | 0 |  |  |
|  | TCGA-E7-A85H | 1.079452 | 0 |  |  |
|  | TCGA-E7-A8O7 | 1.276712 | 0 |  |  |
|  | TCGA-E7-A8O8 | 0.035616 | 0 |  |  |
|  | TCGA-E7-A97P | 1.19726 | 1 |  |  |
|  | TCGA-E7-A97Q | 0.673973 | 1 |  |  |
|  | TCGA-FD-A3B3 | 2.668493 | 1 |  |  |
|  | TCGA-FD-A3B4 | 1.39726 | 1 |  |  |
|  | TCGA-FD-A3B5 | 0.745205 | 1 |  |  |
|  | TCGA-FD-A3B6 | 2.753425 | 1 |  |  |
|  | TCGA-FD-A3B7 | 0.334247 | 1 |  |  |
|  | TCGA-FD-A3B8 | 1.052055 | 0 |  |  |
|  | TCGA-FD-A3N5 | 1.876712 | 1 |  |  |
|  | TCGA-FD-A3N6 | 2.331507 | 0 |  |  |
|  | TCGA-FD-A3NA | 5.054795 | 0 |  |  |
|  | TCGA-FD-A3SJ | 2.024658 | 1 |  |  |
|  | TCGA-FD-A3SL | 1.950685 | 1 |  |  |
|  | TCGA-FD-A3SM | 1.49863 | 1 |  |  |
|  | TCGA-FD-A3SN | 2.430137 | 0 |  |  |
|  | TCGA-FD-A3SO | 0.460274 | 1 |  |  |
|  | TCGA-FD-A3SP | 2.145205 | 0 |  |  |
|  | TCGA-FD-A3SQ | 3.89863 | 1 |  |  |
|  | TCGA-FD-A3SR | 1.649315 | 1 |  |  |
|  | TCGA-FD-A3SS | 1.071233 | 1 |  |  |
|  | TCGA-FD-A43N | 1.915068 | 0 |  |  |
|  | TCGA-FD-A43P | 2.279452 | 0 |  |  |
|  | TCGA-FD-A43S | 1.246575 | 0 |  |  |
|  | TCGA-FD-A43U | 1.742466 | 0 |  |  |
|  | TCGA-FD-A43X | 0.30137 | 0 |  |  |
|  | TCGA-FD-A43Y | 1.29863 | 1 |  |  |
|  | TCGA-FD-A5BR | 2.227397 | 0 |  |  |
|  | TCGA-FD-A5BS | 4.490411 | 0 |  |  |
|  | TCGA-FD-A5BT | 0.89863 | 1 |  |  |
|  | TCGA-FD-A5BU | 1.610959 | 0 |  |  |
|  | TCGA-FD-A5BV | 0.446575 | 1 |  |  |
|  | TCGA-FD-A5BX | 0.473973 | 1 |  |  |
|  | TCGA-FD-A5BY | 0.687671 | 0 |  |  |
|  | TCGA-FD-A5BZ | 2.287671 | 1 |  |  |
|  | TCGA-FD-A5C0 | 1.506849 | 1 |  |  |
|  | TCGA-FD-A5C1 | 4.909589 | 0 |  |  |
|  | TCGA-FD-A62N | 0.224658 | 0 |  |  |
|  | TCGA-FD-A62O | 0.591781 | 1 |  |  |
|  | TCGA-FD-A62P | 0.523288 | 1 |  |  |
|  | TCGA-FD-A62S | 1.112329 | 1 |  |  |
|  | TCGA-FD-A6TA | 5.238356 | 0 |  |  |
|  | TCGA-FD-A6TB | 1.567123 | 0 |  |  |
|  | TCGA-FD-A6TC | 0.512329 | 0 |  |  |
|  | TCGA-FD-A6TD | 1.057534 | 1 |  |  |
|  | TCGA-FD-A6TE | 1.030137 | 0 |  |  |
|  | TCGA-FD-A6TF | 0.189041 | 1 |  |  |
|  | TCGA-FD-A6TG | 0.254795 | 1 |  |  |
|  | TCGA-FD-A6TH | 0.358904 | 1 |  |  |
|  | TCGA-FD-A6TI | 0.805479 | 1 |  |  |
|  | TCGA-FD-A6TK | 0.90411 | 0 |  |  |
|  | TCGA-FJ-A3Z7 | 2.589041 | 0 |  |  |
|  | TCGA-FJ-A3Z9 | 1.054795 | 1 |  |  |
|  | TCGA-FJ-A3ZE | 0.887671 | 1 |  |  |
|  | TCGA-FJ-A3ZF | 1.435616 | 0 |  |  |
|  | TCGA-FJ-A871 | 0.745205 | 1 |  |  |
|  | TCGA-FT-A3EE | 0.271233 | 1 |  |  |
|  | TCGA-FT-A61P | 0.923288 | 0 |  |  |
|  | TCGA-G2-A2EC | 1.906849 | 1 |  |  |
|  | TCGA-G2-A2EF | 5.161644 | 0 |  |  |
|  | TCGA-G2-A2EJ | 4 | 0 |  |  |
|  | TCGA-G2-A2EK | 1.328767 | 0 |  |  |
|  | TCGA-G2-A2EL | 2.243836 | 1 |  |  |
|  | TCGA-G2-A2EO | 4.942466 | 1 |  |  |
|  | TCGA-G2-A2ES | 2.750685 | 1 |  |  |
|  | TCGA-G2-A3IB | 0.60274 | 1 |  |  |
|  | TCGA-G2-A3IE | 1.676712 | 1 |  |  |
|  | TCGA-G2-A3VY | 1.468493 | 0 |  |  |
|  | TCGA-G2-AA3B | 5.50137 | 0 |  |  |
|  | TCGA-G2-AA3C | 0.578082 | 1 |  |  |
|  | TCGA-G2-AA3D | 5.860274 | 0 |  |  |
|  | TCGA-G2-AA3F | 2.446575 | 0 |  |  |
|  | TCGA-GC-A3BM | 1.783562 | 1 |  |  |
|  | TCGA-GC-A3I6 | 1.726027 | 1 |  |  |
|  | TCGA-GC-A3OO | 1.317808 | 0 |  |  |
|  | TCGA-GC-A3RB | 1.594521 | 0 |  |  |
|  | TCGA-GC-A3RC | 1.326027 | 0 |  |  |
|  | TCGA-GC-A3RD | 1.172603 | 0 |  |  |
|  | TCGA-GC-A3WC | 1.479452 | 0 |  |  |
|  | TCGA-GC-A3YS | 2.076712 | 0 |  |  |
|  | TCGA-GC-A4ZW | 0.041096 | 0 |  |  |
|  | TCGA-GC-A6I1 | 0.003 | 0 |  |  |
|  | TCGA-GC-A6I3 | 0.003 | 0 |  |  |
|  | TCGA-GD-A2C5 | 2.224658 | 0 |  |  |
|  | TCGA-GD-A3OP | 0.175342 | 0 |  |  |
|  | TCGA-GD-A3OQ | 0.260274 | 0 |  |  |
|  | TCGA-GD-A3OS | 1.747945 | 0 |  |  |
|  | TCGA-GD-A6C6 | 0.183562 | 0 |  |  |
|  | TCGA-GD-A76B | 0.613699 | 0 |  |  |
|  | TCGA-GU-A42P | 0.909589 | 1 |  |  |
|  | TCGA-GU-A42Q | 0.942466 | 1 |  |  |
|  | TCGA-GU-A42R | 1.580822 | 1 |  |  |
|  | TCGA-GU-A762 | 0.635616 | 1 |  |  |
|  | TCGA-GU-A763 | 2.731507 | 0 |  |  |
|  | TCGA-GU-A764 | 1.671233 | 0 |  |  |
|  | TCGA-GU-A766 | 1.315068 | 0 |  |  |
|  | TCGA-GU-A767 | 0.394521 | 1 |  |  |
|  | TCGA-GU-AATO | 0.887671 | 1 |  |  |
|  | TCGA-GU-AATP | 2.747945 | 0 |  |  |
|  | TCGA-GU-AATQ | 0.583562 | 1 |  |  |
|  | TCGA-GV-A3JV | 1.189041 | 1 |  |  |
|  | TCGA-GV-A3JW | 1.778082 | 0 |  |  |
|  | TCGA-GV-A3JX | 1.591781 | 0 |  |  |
|  | TCGA-GV-A3JZ | 1.652055 | 0 |  |  |
|  | TCGA-GV-A3QF | 1.690411 | 1 |  |  |
|  | TCGA-GV-A3QG | 0.003 | 1 |  |  |
|  | TCGA-GV-A3QH | 0.706849 | 1 |  |  |
|  | TCGA-GV-A3QI | 3.041096 | 0 |  |  |
|  | TCGA-GV-A3QK | 2.279452 | 0 |  |  |
|  | TCGA-GV-A40E | 0.715068 | 1 |  |  |
|  | TCGA-GV-A40G | 1.589041 | 0 |  |  |
|  | TCGA-GV-A6ZA | 1.893151 | 0 |  |  |
|  | TCGA-H4-A2HO | 0.126027 | 0 |  |  |
|  | TCGA-H4-A2HQ | 1.616438 | 0 |  |  |
|  | TCGA-HQ-A2OE | 3.216438 | 0 |  |  |
|  | TCGA-HQ-A2OF | 5.334247 | 0 |  |  |
|  | TCGA-HQ-A5ND | 0.750685 | 1 |  |  |
|  | TCGA-HQ-A5NE | 1.013699 | 1 |  |  |
|  | TCGA-K4-A3WS | 2.084932 | 0 |  |  |
|  | TCGA-K4-A3WU | 0.287671 | 0 |  |  |
|  | TCGA-K4-A3WV | 1.769863 | 0 |  |  |
|  | TCGA-K4-A4AB | 0.208219 | 0 |  |  |
|  | TCGA-K4-A4AC | 0.761644 | 1 |  |  |
|  | TCGA-K4-A54R | 2.306849 | 0 |  |  |
|  | TCGA-K4-A5RH | 0.756164 | 0 |  |  |
|  | TCGA-K4-A5RI | 0.975342 | 1 |  |  |
|  | TCGA-K4-A5RJ | 1.476712 | 0 |  |  |
|  | TCGA-K4-A6FZ | 0.150685 | 0 |  |  |
|  | TCGA-K4-A6MB | 1.284932 | 0 |  |  |
|  | TCGA-K4-A83P | 1.356164 | 0 |  |  |
|  | TCGA-K4-AAQO | 0.983562 | 0 |  |  |
|  | TCGA-KQ-A41N | 4.394521 | 0 |  |  |
|  | TCGA-KQ-A41O | 4.213699 | 0 |  |  |
|  | TCGA-KQ-A41P | 2.99726 | 0 |  |  |
|  | TCGA-KQ-A41Q | 0.989041 | 0 |  |  |
|  | TCGA-KQ-A41R | 3.69863 | 0 |  |  |
|  | TCGA-KQ-A41S | 0.09589 | 0 |  |  |
|  | TCGA-LC-A66R | 1.276712 | 0 |  |  |
|  | TCGA-LT-A5Z6 | 1.29863 | 0 |  |  |
|  | TCGA-LT-A8JT | 1.756164 | 0 |  |  |
|  | TCGA-MV-A51V | 1.123288 | 0 |  |  |
|  | TCGA-PQ-A6FI | 1.019178 | 0 |  |  |
|  | TCGA-PQ-A6FN | 1.389041 | 0 |  |  |
|  | TCGA-R3-A69X | 1.186301 | 0 |  |  |
|  | TCGA-S5-A6DX | 0.153425 | 1 |  |  |
|  | TCGA-S5-AA26 | 1.378082 | 0 |  |  |
|  | TCGA-SY-A9G0 | 2.761644 | 1 |  |  |
|  | TCGA-SY-A9G5 | 3.249315 | 0 |  |  |
|  | TCGA-UY-A78K | 1.468493 | 1 |  |  |
|  | TCGA-UY-A78L | 3.087671 | 0 |  |  |
|  | TCGA-UY-A78M | 1.890411 | 1 |  |  |
|  | TCGA-UY-A78N | 7.235616 | 1 |  |  |
|  | TCGA-UY-A78O | 6.334247 | 0 |  |  |
|  | TCGA-UY-A78P | 6.520548 | 0 |  |  |
|  | TCGA-UY-A8OB | 5.778082 | 0 |  |  |
|  | TCGA-UY-A8OC | 0.003 | 1 |  |  |
|  | TCGA-UY-A8OD | 9.40274 | 0 |  |  |
|  | TCGA-UY-A9PA | 2.936986 | 0 |  |  |
|  | TCGA-UY-A9PB | 2.463014 | 0 |  |  |
|  | TCGA-UY-A9PD | 1.484932 | 0 |  |  |
|  | TCGA-UY-A9PE | 0.517808 | 0 |  |  |
|  | TCGA-UY-A9PF | 0.320548 | 0 |  |  |
|  | TCGA-UY-A9PH | 4.276712 | 0 |  |  |
|  | TCGA-XF-A8HB | 3.753425 | 0 |  |  |
|  | TCGA-XF-A8HC | 0.547945 | 1 |  |  |
|  | TCGA-XF-A8HD | 8.120548 | 0 |  |  |
|  | TCGA-XF-A8HE | 10.45753 | 0 |  |  |
|  | TCGA-XF-A8HF | 8.093151 | 1 |  |  |
|  | TCGA-XF-A8HG | 1.279452 | 1 |  |  |
|  | TCGA-XF-A8HH | 0.156164 | 1 |  |  |
|  | TCGA-XF-A8HI | 1.490411 | 1 |  |  |
|  | TCGA-XF-A9SH | 5.4 | 1 |  |  |
|  | TCGA-XF-A9SI | 6.638356 | 0 |  |  |
|  | TCGA-XF-A9SJ | 0.268493 | 1 |  |  |
|  | TCGA-XF-A9SK | 1.331507 | 1 |  |  |
|  | TCGA-XF-A9SL | 5.534247 | 1 |  |  |
|  | TCGA-XF-A9SM | 2.871233 | 0 |  |  |
|  | TCGA-XF-A9SP | 1.243836 | 1 |  |  |
|  | TCGA-XF-A9ST | 0.350685 | 1 |  |  |
|  | TCGA-XF-A9SU | 0.49863 | 1 |  |  |
|  | TCGA-XF-A9SV | 1.063014 | 1 |  |  |
|  | TCGA-XF-A9SW | 0.991781 | 1 |  |  |
|  | TCGA-XF-A9SX | 1.969863 | 1 |  |  |
|  | TCGA-XF-A9SY | 1.753425 | 0 |  |  |
|  | TCGA-XF-A9SZ | 2.353425 | 1 |  |  |
|  | TCGA-XF-A9T0 | 2.189041 | 0 |  |  |
|  | TCGA-XF-A9T2 | 1.575342 | 1 |  |  |
|  | TCGA-XF-A9T3 | 0.186301 | 0 |  |  |
|  | TCGA-XF-A9T4 | 1.356164 | 1 |  |  |
|  | TCGA-XF-A9T5 | 5.553425 | 0 |  |  |
|  | TCGA-XF-A9T6 | 0.175342 | 0 |  |  |
|  | TCGA-XF-A9T8 | 1.145205 | 1 |  |  |
|  | TCGA-XF-AAME | 7.747945 | 1 |  |  |
|  | TCGA-XF-AAMG | 9.216438 | 0 |  |  |
|  | TCGA-XF-AAMH | 0.942466 | 1 |  |  |
|  | TCGA-XF-AAMJ | 4.575342 | 1 |  |  |
|  | TCGA-XF-AAML | 0.635616 | 1 |  |  |
|  | TCGA-XF-AAMQ | 5.964384 | 0 |  |  |
|  | TCGA-XF-AAMR | 7.643836 | 0 |  |  |
|  | TCGA-XF-AAMT | 0.246575 | 1 |  |  |
|  | TCGA-XF-AAMW | 0.693151 | 1 |  |  |
|  | TCGA-XF-AAMX | 0.564384 | 1 |  |  |
|  | TCGA-XF-AAMY | 8.249315 | 0 |  |  |
|  | TCGA-XF-AAMZ | 3.693151 | 1 |  |  |
|  | TCGA-XF-AAN0 | 4.706849 | 1 |  |  |
|  | TCGA-XF-AAN1 | 2.578082 | 1 |  |  |
|  | TCGA-XF-AAN2 | 5.120548 | 1 |  |  |
|  | TCGA-XF-AAN3 | 7.191781 | 0 |  |  |
|  | TCGA-XF-AAN4 | 2.254795 | 1 |  |  |
|  | TCGA-XF-AAN5 | 6.282192 | 0 |  |  |
|  | TCGA-XF-AAN7 | 1.547945 | 1 |  |  |
|  | TCGA-XF-AAN8 | 0.323288 | 1 |  |  |
|  | TCGA-YC-A89H | 1.569863 | 0 |  |  |
|  | TCGA-YC-A8S6 | 0.80274 | 0 |  |  |
|  | TCGA-YC-A9TC | 0.054795 | 1 |  |  |
|  | TCGA-YF-AA3L | 0.99726 | 0 |  |  |
|  | TCGA-YF-AA3M | 1.136986 | 0 |  |  |
|  | TCGA-ZF-A9R0 | 1.863014 | 1 |  |  |
|  | TCGA-ZF-A9R1 | 2.117808 | 0 |  |  |
|  | TCGA-ZF-A9R2 | 1.758904 | 0 |  |  |
|  | TCGA-ZF-A9R3 | 2.6 | 1 |  |  |
|  | TCGA-ZF-A9R4 | 2.523288 | 0 |  |  |
|  | TCGA-ZF-A9R5 | 2.986301 | 0 |  |  |
|  | TCGA-ZF-A9R7 | 1.821918 | 0 |  |  |
|  | TCGA-ZF-A9R9 | 2.367123 | 1 |  |  |
|  | TCGA-ZF-A9RC | 7.857534 | 0 |  |  |
|  | TCGA-ZF-A9RD | 1.117808 | 1 |  |  |
|  | TCGA-ZF-A9RE | 0.290411 | 1 |  |  |
|  | TCGA-ZF-A9RL | 7.405479 | 0 |  |  |
|  | TCGA-ZF-A9RN | 1.684932 | 1 |  |  |
|  | TCGA-ZF-AA4N | 0.241096 | 1 |  |  |
|  | TCGA-ZF-AA4R | 2.838356 | 1 |  |  |
|  | TCGA-ZF-AA4T | 1.641096 | 1 |  |  |
|  | TCGA-ZF-AA4U | 0.717808 | 1 |  |  |
|  | TCGA-ZF-AA4V | 4.947945 | 0 |  |  |
|  | TCGA-ZF-AA4W | 5.013699 | 0 |  |  |
|  | TCGA-ZF-AA4X | 5.6 | 0 |  |  |
|  | TCGA-ZF-AA51 | 4.69589 | 0 |  |  |
|  | TCGA-ZF-AA52 | 2.950685 | 1 |  |  |
|  | TCGA-ZF-AA53 | 4.824658 | 0 |  |  |
|  | TCGA-ZF-AA54 | 1.616438 | 1 |  |  |
|  | TCGA-ZF-AA56 | 0.709589 | 1 |  |  |
|  | TCGA-ZF-AA58 | 4.517808 | 0 |  |  |
|  | TCGA-ZF-AA5H | 2.457534 | 0 |  |  |
|  | TCGA-ZF-AA5N | 0.460274 | 1 |  |  |
|  | TCGA-ZF-AA5P | 1.019178 | 0 |  |  |
