# Supplementary material for: NXPH4 Used as a New Prognostic and Immunotherapeutic Marker for Muscle-Invasive Bladder Cancer
Source: J Oncol. 2022 Oct 4;2022:4271409. doi: 10.1155/2022/4271409 (PMC9553512; doi:10.1155/2022/4271409)
Supplement: Supplementary Materials — Figure s1: article roadmap of the whole research. Figure s2: (A) GSVA results heatmap of invasive bladder cancer in TCGA database (normal =19, tumor =404); Wayne diagram of differential pathways between clusters. (B) Wayne diagram in TCGA clusters (n = 65). (C) Wayne diagram in GEO clusters (n = 463). (D) Wayne diagram in TCGA clusters and GEO clusters (n = 6). Figure s3: (A) the 28 prognostic key pathway genes (P <0.01). Risk model for patients with muscle invasive bladder cancer (MIBC) based on 12 genes (SLC7A2, MST1R, CDK6, NXPH4, GRIK2, TRIB3, PBK, ABCA4, FBN2, SCG2, ELN, and INCENP). (B) LASSO regression with 10-fold crossvalidation was used to obtain 12 prognostic genes with an error within one standard error of the minimum (lambda.1se). (C) LASSO coefficient profiles of 28 key pathway genes. Supplement Table 1: clinical characteristics such as N, M, T, tumor grade, and stage, including age among the three groups (TCGA). Supplement Table 2: survival and prognosis information of three groups based on GEO. Supplement Table 3: 65 differential pathways were obtained from the molecular subtypes of TCGA queue. Supplement Table 4: 6 common differential pathways were obtained based on 65 TCGA, differential pathways, and 463 GEO, differential pathways. Supplement Table 5: 6 common differential pathways with prognosis. Supplement Table 6: the risk model based on the 12 prognostic genes in TCGA and GEO databases. Supplement Table 7: immune landscape between the high- and low-risk patients with muscle invasive bladder cancer (MIBC). Supplement Table 8: evaluation of immune response to CTLA4 and PD1 immunosuppressants in MIBC patients. [file 4271409.f1.zip › supplement table7.docx]

**Supplement Table7** Immune landscape between the high- and low-risk patients with muscle invasive bladder cancer (MIBC).

| immuneCor |  |
| --- | --- |
| immune | pvalue |
| T cell CD8+_TIMER | 9.95E-06 |
| Neutrophil_TIMER | 1.16E-05 |
| Macrophage_TIMER | 9.48E-13 |
| Myeloid dendritic cell_TIMER | 6.59E-06 |
| B cell naive_CIBERSORT | 0.00193016 |
| B cell plasma_CIBERSORT | 0.019588178 |
| T cell CD8+_CIBERSORT | 0.006934993 |
| T cell CD4+ naive_CIBERSORT | 0.000477407 |
| T cell follicular helper_CIBERSORT | 1.80E-05 |
| Macrophage M0_CIBERSORT | 1.57E-06 |
| Macrophage M1_CIBERSORT | 0.001041762 |
| Macrophage M2_CIBERSORT | 8.06E-05 |
| Myeloid dendritic cell activated_CIBERSORT | 2.37E-06 |
| B cell naive_CIBERSORT-ABS | 0.0002081 |
| T cell CD4+ naive_CIBERSORT-ABS | 0.000658857 |
| T cell regulatory (Tregs)_CIBERSORT-ABS | 0.030843468 |
| NK cell activated_CIBERSORT-ABS | 0.033178036 |
| Macrophage M0_CIBERSORT-ABS | 2.35E-08 |
| Macrophage M1_CIBERSORT-ABS | 6.83E-05 |
| Macrophage M2_CIBERSORT-ABS | 3.06E-08 |
| Myeloid dendritic cell activated_CIBERSORT-ABS | 0.000630617 |
| B cell_QUANTISEQ | 0.0283507 |
| Macrophage M1_QUANTISEQ | 8.07E-07 |
| Macrophage M2_QUANTISEQ | 0.000519003 |
| Neutrophil_QUANTISEQ | 0.047806674 |
| T cell CD8+_QUANTISEQ | 0.034232761 |
| T cell regulatory (Tregs)_QUANTISEQ | 0.000110145 |
| uncharacterized cell_QUANTISEQ | 0.000159933 |
| T cell CD8+_MCPCOUNTER | 0.044149574 |
| cytotoxicity score_MCPCOUNTER | 0.000136879 |
| NK cell_MCPCOUNTER | 5.49E-05 |
| Monocyte_MCPCOUNTER | 3.48E-09 |
| Macrophage/Monocyte_MCPCOUNTER | 3.48E-09 |
| Myeloid dendritic cell_MCPCOUNTER | 0.000168775 |
| Cancer associated fibroblast_MCPCOUNTER | 1.26E-10 |
| Myeloid dendritic cell activated_XCELL | 0.002035344 |
| T cell CD4+ naive_XCELL | 0.00540374 |
| T cell CD4+ central memory_XCELL | 0.001828964 |
| T cell CD4+ effector memory_XCELL | 0.002395383 |
| T cell CD8+ naive_XCELL | 0.001853555 |
| T cell CD8+_XCELL | 0.001979337 |
| Class-switched memory B cell_XCELL | 0.000349611 |
| Common lymphoid progenitor_XCELL | 0.015972251 |
| Eosinophil_XCELL | 0.000476685 |
| Cancer associated fibroblast_XCELL | 7.63E-05 |
| Granulocyte-monocyte progenitor_XCELL | 0.000814104 |
| Macrophage_XCELL | 1.12E-05 |
| Macrophage M1_XCELL | 7.66E-06 |
| Macrophage M2_XCELL | 0.000121466 |
| Mast cell_XCELL | 0.0114221 |
| Monocyte_XCELL | 0.012892312 |
| B cell naive_XCELL | 0.003721455 |
| NK cell_XCELL | 0.043285879 |
| T cell NK_XCELL | 0.001170383 |
| Plasmacytoid dendritic cell_XCELL | 0.019809166 |
| T cell CD4+ Th2_XCELL | 2.73E-12 |
| immune score_XCELL | 0.028965301 |
| stroma score_XCELL | 0.003679707 |
| microenvironment score_XCELL | 0.001251753 |
| Cancer associated fibroblast_EPIC | 4.18E-08 |
| T cell CD4+_EPIC | 1.71E-08 |
| Macrophage_EPIC | 4.82E-08 |
| NK cell_EPIC | 0.000261378 |
| uncharacterized cell_EPIC | 0.012519527 |
