# Supplementary material for: NXPH4 Used as a New Prognostic and Immunotherapeutic Marker for Muscle-Invasive Bladder Cancer
Source: J Oncol. 2022 Oct 4;2022:4271409. doi: 10.1155/2022/4271409 (PMC9553512; doi:10.1155/2022/4271409)
Supplement: Supplementary Materials — Figure s1: article roadmap of the whole research. Figure s2: (A) GSVA results heatmap of invasive bladder cancer in TCGA database (normal =19, tumor =404); Wayne diagram of differential pathways between clusters. (B) Wayne diagram in TCGA clusters (n = 65). (C) Wayne diagram in GEO clusters (n = 463). (D) Wayne diagram in TCGA clusters and GEO clusters (n = 6). Figure s3: (A) the 28 prognostic key pathway genes (P <0.01). Risk model for patients with muscle invasive bladder cancer (MIBC) based on 12 genes (SLC7A2, MST1R, CDK6, NXPH4, GRIK2, TRIB3, PBK, ABCA4, FBN2, SCG2, ELN, and INCENP). (B) LASSO regression with 10-fold crossvalidation was used to obtain 12 prognostic genes with an error within one standard error of the minimum (lambda.1se). (C) LASSO coefficient profiles of 28 key pathway genes. Supplement Table 1: clinical characteristics such as N, M, T, tumor grade, and stage, including age among the three groups (TCGA). Supplement Table 2: survival and prognosis information of three groups based on GEO. Supplement Table 3: 65 differential pathways were obtained from the molecular subtypes of TCGA queue. Supplement Table 4: 6 common differential pathways were obtained based on 65 TCGA, differential pathways, and 463 GEO, differential pathways. Supplement Table 5: 6 common differential pathways with prognosis. Supplement Table 6: the risk model based on the 12 prognostic genes in TCGA and GEO databases. Supplement Table 7: immune landscape between the high- and low-risk patients with muscle invasive bladder cancer (MIBC). Supplement Table 8: evaluation of immune response to CTLA4 and PD1 immunosuppressants in MIBC patients. [file 4271409.f1.zip › supplement table8.docx]

**Supplement Table8** Evaluation of immune response to CTLA4 and PD1 immunosuppressants in MIBC patients

| barcode | ips_ctla4_neg_pd1_neg | ips_ctla4_neg_pd1_pos | ips_ctla4_pos_pd1_neg | ips_ctla4_pos_pd1_pos | | NXPH4 |
| --- | --- | --- | --- | --- | --- | --- |
| TCGA-ZF-AA5P | 9 | 8 | 9 | 8 |  | 2.273435 |
| TCGA-ZF-AA5N | 8 | 7 | 8 | 6 |  | 2.034078 |
| TCGA-ZF-AA5H | 8 | 9 | 8 | 9 |  | 2.262477 |
| TCGA-ZF-AA58 | 8 | 9 | 8 | 9 |  | 1.910816 |
| TCGA-ZF-AA56 | 9 | 7 | 8 | 7 |  | 5.322623 |
| TCGA-ZF-AA54 | 8 | 8 | 8 | 8 |  | 4.051823 |
| TCGA-ZF-AA53 | 7 | 8 | 7 | 7 |  | 2.244972 |
| TCGA-ZF-AA52 | 8 | 6 | 7 | 6 |  | 2.10756 |
| TCGA-ZF-AA51 | 8 | 7 | 8 | 7 |  | 0.987104 |
| TCGA-ZF-AA4X | 10 | 9 | 10 | 8 |  | 3.419907 |
| TCGA-ZF-AA4W | 9 | 8 | 8 | 7 |  | 5.197333 |
| TCGA-ZF-AA4V | 7 | 7 | 7 | 7 |  | 2.005637 |
| TCGA-ZF-AA4U | 10 | 8 | 10 | 8 |  | 3.734595 |
| TCGA-ZF-AA4T | 10 | 8 | 9 | 8 |  | 1.366906 |
| TCGA-ZF-AA4R | 8 | 7 | 8 | 6 |  | 4.574809 |
| TCGA-ZF-AA4N | 8 | 7 | 7 | 7 |  | 4.624976 |
| TCGA-ZF-A9RN | 8 | 8 | 8 | 8 |  | 1.770988 |
| TCGA-ZF-A9RM | 9 | 7 | 8 | 6 |  | 2.778781 |
| TCGA-ZF-A9RL | 9 | 7 | 8 | 6 |  | 1.588401 |
| TCGA-ZF-A9RF | 9 | 10 | 9 | 10 |  | 1.676619 |
| TCGA-ZF-A9RE | 8 | 6 | 7 | 5 |  | 5.893506 |
| TCGA-ZF-A9RD | 8 | 8 | 8 | 8 |  | 4.335637 |
| TCGA-ZF-A9RC | 7 | 5 | 6 | 4 |  | 1.995393 |
| TCGA-ZF-A9R9 | 9 | 7 | 8 | 7 |  | 1.033982 |
| TCGA-ZF-A9R7 | 10 | 10 | 10 | 10 |  | 1.570024 |
| TCGA-ZF-A9R5 | 10 | 9 | 10 | 8 |  | 0.983424 |
| TCGA-ZF-A9R4 | 10 | 10 | 10 | 9 |  | 2.597379 |
| TCGA-ZF-A9R3 | 9 | 8 | 9 | 8 |  | 0.983458 |
| TCGA-ZF-A9R2 | 8 | 6 | 8 | 6 |  | 4.287209 |
| TCGA-ZF-A9R1 | 8 | 6 | 8 | 6 |  | 2.301736 |
| TCGA-ZF-A9R0 | 10 | 9 | 10 | 8 |  | 1.69742 |
| TCGA-YF-AA3M | 8 | 6 | 7 | 6 |  | 2.674764 |
| TCGA-YF-AA3L | 8 | 6 | 8 | 6 |  | 0.782878 |
| TCGA-YC-A9TC | 8 | 7 | 8 | 7 |  | 6.142111 |
| TCGA-YC-A8S6 | 8 | 7 | 8 | 7 |  | 3.137841 |
| TCGA-YC-A89H | 7 | 5 | 6 | 5 |  | 5.330629 |
| TCGA-XF-AAN8 | 7 | 6 | 7 | 6 |  | 2.243571 |
| TCGA-XF-AAN7 | 8 | 6 | 7 | 6 |  | 4.213864 |
| TCGA-XF-AAN5 | 8 | 8 | 8 | 8 |  | 2.824415 |
| TCGA-XF-AAN4 | 6 | 6 | 6 | 6 |  | 1.151194 |
| TCGA-XF-AAN3 | 8 | 6 | 7 | 6 |  | 4.25634 |
| TCGA-XF-AAN2 | 8 | 9 | 8 | 8 |  | 1.22486 |
| TCGA-XF-AAN1 | 9 | 8 | 9 | 7 |  | 2.245105 |
| TCGA-XF-AAN0 | 8 | 6 | 7 | 6 |  | 1.009333 |
| TCGA-XF-AAMZ | 8 | 6 | 8 | 6 |  | 5.051947 |
| TCGA-XF-AAMY | 7 | 5 | 6 | 5 |  | 4.537479 |
| TCGA-XF-AAMX | 7 | 6 | 7 | 5 |  | 3.444622 |
| TCGA-XF-AAMW | 8 | 7 | 8 | 7 |  | 1.544257 |
| TCGA-XF-AAMT | 8 | 7 | 8 | 7 |  | 3.99291 |
| TCGA-XF-AAMR | 9 | 7 | 8 | 6 |  | 1.45124 |
| TCGA-XF-AAMQ | 8 | 8 | 8 | 7 |  | 3.148434 |
| TCGA-XF-AAML | 10 | 10 | 10 | 10 |  | 2.240625 |
| TCGA-XF-AAMJ | 8 | 6 | 8 | 6 |  | 1.492851 |
| TCGA-XF-AAMH | 6 | 5 | 6 | 5 |  | 1.946465 |
| TCGA-XF-AAMG | 8 | 6 | 7 | 6 |  | 4.627217 |
| TCGA-XF-AAME | 8 | 7 | 8 | 7 |  | 2.021931 |
| TCGA-XF-A9T8 | 7 | 7 | 7 | 7 |  | 5.205141 |
| TCGA-XF-A9T6 | 8 | 7 | 8 | 7 |  | 4.318037 |
| TCGA-XF-A9T5 | 10 | 10 | 10 | 10 |  | 6.409415 |
| TCGA-XF-A9T4 | 8 | 9 | 8 | 8 |  | 2.050034 |
| TCGA-XF-A9T3 | 9 | 9 | 9 | 9 |  | 2.485342 |
| TCGA-XF-A9T2 | 8 | 6 | 7 | 6 |  | 2.918682 |
| TCGA-XF-A9T0 | 7 | 6 | 7 | 6 |  | 4.141227 |
| TCGA-XF-A9SZ | 8 | 7 | 8 | 7 |  | 1.507796 |
| TCGA-XF-A9SY | 7 | 8 | 7 | 8 |  | 1.858738 |
| TCGA-XF-A9SX | 8 | 8 | 8 | 8 |  | 2.783577 |
| TCGA-XF-A9SW | 8 | 6 | 7 | 6 |  | 1.282407 |
| TCGA-XF-A9SV | 7 | 5 | 7 | 5 |  | 3.996072 |
| TCGA-XF-A9SU | 7 | 6 | 7 | 6 |  | 3.693342 |
| TCGA-XF-A9ST | 7 | 6 | 6 | 5 |  | 4.956859 |
| TCGA-XF-A9SP | 9 | 7 | 8 | 7 |  | 1.142705 |
| TCGA-XF-A9SM | 8 | 8 | 8 | 9 |  | 4.128642 |
| TCGA-XF-A9SL | 8 | 7 | 8 | 7 |  | 1.632882 |
| TCGA-XF-A9SK | 8 | 7 | 8 | 7 |  | 3.700566 |
| TCGA-XF-A9SJ | 8 | 8 | 8 | 8 |  | 3.777623 |
| TCGA-XF-A9SI | 8 | 9 | 9 | 9 |  | 2.066592 |
| TCGA-XF-A9SH | 8 | 6 | 7 | 5 |  | 1.103448 |
| TCGA-XF-A8HI | 9 | 7 | 8 | 7 |  | 2.43382 |
| TCGA-XF-A8HH | 8 | 6 | 7 | 6 |  | 6.230908 |
| TCGA-XF-A8HG | 8 | 6 | 7 | 5 |  | 5.289433 |
| TCGA-XF-A8HF | 9 | 7 | 8 | 7 |  | 2.1205 |
| TCGA-XF-A8HE | 7 | 7 | 7 | 7 |  | 4.877219 |
| TCGA-XF-A8HD | 10 | 10 | 10 | 10 |  | 1.123823 |
| TCGA-XF-A8HC | 8 | 6 | 8 | 6 |  | 1.092721 |
| TCGA-XF-A8HB | 9 | 7 | 8 | 6 |  | 5.477796 |
| TCGA-UY-A9PH | 9 | 9 | 9 | 9 |  | 1.235415 |
| TCGA-UY-A9PF | 9 | 7 | 9 | 7 |  | 5.740711 |
| TCGA-UY-A9PE | 9 | 7 | 8 | 6 |  | 2.446026 |
| TCGA-UY-A9PD | 10 | 8 | 10 | 8 |  | 2.083653 |
| TCGA-UY-A9PB | 8 | 8 | 8 | 8 |  | 2.507388 |
| TCGA-UY-A9PA | 10 | 10 | 10 | 10 |  | 1.1998 |
| TCGA-UY-A8OD | 8 | 6 | 8 | 6 |  | 1.638006 |
| TCGA-UY-A8OC | 7 | 6 | 7 | 5 |  | 3.789302 |
| TCGA-UY-A8OB | 10 | 10 | 10 | 10 |  | 2.678532 |
| TCGA-UY-A78P | 8 | 8 | 8 | 8 |  | 2.013912 |
| TCGA-UY-A78O | 8 | 7 | 8 | 6 |  | 4.419675 |
| TCGA-UY-A78N | 7 | 5 | 7 | 5 |  | 3.525343 |
| TCGA-UY-A78M | 7 | 6 | 7 | 5 |  | 6.253482 |
| TCGA-UY-A78L | 7 | 6 | 7 | 6 |  | 1.554855 |
| TCGA-UY-A78K | 8 | 8 | 9 | 8 |  | 3.880841 |
| TCGA-SY-A9G5 | 8 | 8 | 8 | 8 |  | 1.553165 |
| TCGA-SY-A9G0 | 8 | 7 | 8 | 6 |  | 1.720371 |
| TCGA-S5-AA26 | 7 | 5 | 7 | 5 |  | 4.303512 |
| TCGA-S5-A6DX | 7 | 6 | 7 | 7 |  | 2.204837 |
| TCGA-R3-A69X | 8 | 7 | 8 | 7 |  | 1.701684 |
| TCGA-PQ-A6FN | 8 | 6 | 7 | 6 |  | 1.828451 |
| TCGA-PQ-A6FI | 9 | 8 | 9 | 8 |  | 4.446357 |
| TCGA-MV-A51V | 9 | 7 | 8 | 6 |  | 0.814369 |
| TCGA-LT-A8JT | 10 | 8 | 9 | 7 |  | 3.957374 |
| TCGA-LT-A5Z6 | 8 | 7 | 8 | 6 |  | 1.173559 |
| TCGA-LC-A66R | 8 | 9 | 8 | 9 |  | 2.882096 |
| TCGA-KQ-A41S | 9 | 7 | 8 | 6 |  | 0.882884 |
| TCGA-KQ-A41R | 8 | 6 | 8 | 6 |  | 4.055345 |
| TCGA-KQ-A41Q | 9 | 7 | 8 | 6 |  | 2.357192 |
| TCGA-KQ-A41P | 7 | 6 | 7 | 5 |  | 4.085729 |
| TCGA-KQ-A41O | 7 | 5 | 6 | 4 |  | 1.17731 |
| TCGA-KQ-A41N | 8 | 6 | 7 | 5 |  | 1.842403 |
| TCGA-K4-AAQO | 9 | 7 | 8 | 7 |  | 3.612118 |
| TCGA-K4-A83P | 8 | 8 | 8 | 8 |  | 3.112433 |
| TCGA-K4-A6MB | 8 | 6 | 7 | 5 |  | 2.862684 |
| TCGA-K4-A6FZ | 8 | 8 | 8 | 7 |  | 3.46055 |
| TCGA-K4-A5RJ | 8 | 9 | 8 | 9 |  | 5.439952 |
| TCGA-K4-A5RI | 7 | 5 | 6 | 4 |  | 2.826156 |
| TCGA-K4-A5RH | 9 | 10 | 9 | 10 |  | 2.612526 |
| TCGA-K4-A54R | 7 | 7 | 7 | 7 |  | 1.745234 |
| TCGA-K4-A4AC | 8 | 7 | 7 | 6 |  | 2.476476 |
| TCGA-K4-A4AB | 9 | 7 | 8 | 6 |  | 3.94331 |
| TCGA-K4-A3WV | 7 | 6 | 7 | 5 |  | 1.933203 |
| TCGA-K4-A3WU | 8 | 7 | 8 | 7 |  | 5.52685 |
| TCGA-K4-A3WS | 8 | 7 | 8 | 7 |  | 2.250313 |
| TCGA-HQ-A5NE | 8 | 8 | 8 | 7 |  | 1.866018 |
| TCGA-HQ-A5ND | 9 | 8 | 9 | 7 |  | 5.152854 |
| TCGA-HQ-A2OF | 8 | 6 | 7 | 5 |  | 1.065494 |
| TCGA-HQ-A2OE | 7 | 5 | 6 | 5 |  | 2.938116 |
| TCGA-H4-A2HQ | 8 | 6 | 8 | 6 |  | 4.569004 |
| TCGA-H4-A2HO | 9 | 7 | 8 | 6 |  | 2.896242 |
| TCGA-GV-A6ZA | 10 | 9 | 10 | 9 |  | 2.603452 |
| TCGA-GV-A40G | 8 | 6 | 7 | 5 |  | 1.869259 |
| TCGA-GV-A40E | 7 | 7 | 7 | 7 |  | 3.665391 |
| TCGA-GV-A3QK | 10 | 9 | 10 | 9 |  | 2.483595 |
| TCGA-GV-A3QI | 10 | 8 | 9 | 7 |  | 6.238505 |
| TCGA-GV-A3QH | 9 | 8 | 9 | 7 |  | 1.598517 |
| TCGA-GV-A3QG | 8 | 8 | 8 | 8 |  | 3.222717 |
| TCGA-GV-A3QF | 7 | 5 | 6 | 4 |  | 4.630991 |
| TCGA-GV-A3JZ | 7 | 6 | 7 | 6 |  | 3.54882 |
| TCGA-GV-A3JX | 9 | 8 | 9 | 8 |  | 2.278143 |
| TCGA-GV-A3JW | 7 | 5 | 7 | 4 |  | 3.798152 |
| TCGA-GV-A3JV | 9 | 8 | 9 | 8 |  | 1.683042 |
| TCGA-GU-AATQ | 8 | 6 | 8 | 6 |  | 5.491729 |
| TCGA-GU-AATP | 9 | 8 | 9 | 8 |  | 1.733138 |
| TCGA-GU-AATO | 8 | 7 | 8 | 7 |  | 4.225955 |
| TCGA-GU-A767 | 9 | 7 | 8 | 6 |  | 2.124374 |
| TCGA-GU-A766 | 8 | 9 | 8 | 9 |  | 1.947895 |
| TCGA-GU-A764 | 7 | 8 | 6 | 7 |  | 4.214965 |
| TCGA-GU-A763 | 10 | 9 | 10 | 8 |  | 1.592058 |
| TCGA-GU-A762 | 9 | 10 | 9 | 10 |  | 2.475719 |
| TCGA-GU-A42R | 7 | 5 | 7 | 5 |  | 2.031587 |
| TCGA-GU-A42Q | 8 | 6 | 7 | 5 |  | 6.530306 |
| TCGA-GU-A42P | 7 | 5 | 7 | 5 |  | 3.532515 |
| TCGA-GD-A76B | 10 | 10 | 10 | 10 |  | 1.895653 |
| TCGA-GD-A6C6 | 8 | 7 | 8 | 6 |  | 1.825972 |
| TCGA-GD-A3OS | 9 | 8 | 9 | 7 |  | 6.451192 |
| TCGA-GD-A3OQ | 8 | 7 | 7 | 6 |  | 4.284317 |
| TCGA-GD-A3OP | 9 | 7 | 8 | 7 |  | 0.960844 |
| TCGA-GD-A2C5 | 9 | 7 | 8 | 7 |  | 2.375298 |
| TCGA-GC-A6I3 | 10 | 9 | 10 | 9 |  | 2.646924 |
| TCGA-GC-A6I1 | 8 | 8 | 8 | 8 |  | 4.027546 |
| TCGA-GC-A4ZW | 9 | 8 | 9 | 8 |  | 1.85812 |
| TCGA-GC-A3YS | 8 | 7 | 8 | 7 |  | 4.27348 |
| TCGA-GC-A3WC | 9 | 10 | 9 | 10 |  | 3.568457 |
| TCGA-GC-A3RD | 7 | 5 | 7 | 5 |  | 1.393497 |
| TCGA-GC-A3RC | 8 | 8 | 8 | 8 |  | 4.331632 |
| TCGA-GC-A3RB | 9 | 7 | 8 | 6 |  | 4.063493 |
| TCGA-GC-A3OO | 8 | 6 | 7 | 6 |  | 2.178022 |
| TCGA-GC-A3I6 | 9 | 8 | 9 | 8 |  | 4.430992 |
| TCGA-GC-A3BM | 9 | 8 | 9 | 7 |  | 4.515278 |
| TCGA-G2-AA3F | 8 | 6 | 7 | 6 |  | 2.594563 |
| TCGA-G2-AA3D | 9 | 7 | 9 | 7 |  | 2.819846 |
| TCGA-G2-AA3C | 7 | 6 | 6 | 5 |  | 2.188714 |
| TCGA-G2-AA3B | 10 | 9 | 10 | 9 |  | 4.234728 |
| TCGA-G2-A3VY | 9 | 6 | 8 | 6 |  | 4.06258 |
| TCGA-G2-A3IE | 8 | 6 | 8 | 6 |  | 2.765272 |
| TCGA-G2-A3IB | 8 | 7 | 8 | 6 |  | 4.864465 |
| TCGA-G2-A2ES | 7 | 7 | 7 | 7 |  | 4.691656 |
| TCGA-G2-A2EO | 8 | 7 | 8 | 7 |  | 5.617305 |
| TCGA-G2-A2EL | 9 | 7 | 8 | 6 |  | 5.466853 |
| TCGA-G2-A2EK | 9 | 7 | 8 | 6 |  | 1.428185 |
| TCGA-G2-A2EJ | 8 | 8 | 8 | 8 |  | 4.386313 |
| TCGA-G2-A2EF | 6 | 7 | 7 | 7 |  | 2.390535 |
| TCGA-G2-A2EC | 9 | 7 | 9 | 7 |  | 1.106575 |
| TCGA-FT-A61P | 9 | 9 | 8 | 9 |  | 1.217207 |
| TCGA-FT-A3EE | 7 | 6 | 7 | 6 |  | 3.771564 |
| TCGA-FJ-A871 | 9 | 7 | 8 | 7 |  | 3.005555 |
| TCGA-FJ-A3ZF | 8 | 6 | 7 | 5 |  | 2.510617 |
| TCGA-FJ-A3ZE | 7 | 5 | 6 | 4 |  | 5.860969 |
| TCGA-FJ-A3Z9 | 8 | 6 | 7 | 5 |  | 1.639822 |
| TCGA-FJ-A3Z7 | 8 | 7 | 8 | 7 |  | 2.319526 |
| TCGA-FD-A6TK | 8 | 8 | 8 | 8 |  | 1.999967 |
| TCGA-FD-A6TI | 9 | 7 | 8 | 7 |  | 2.519636 |
| TCGA-FD-A6TH | 7 | 7 | 7 | 6 |  | 2.081394 |
| TCGA-FD-A6TG | 8 | 6 | 7 | 6 |  | 5.014919 |
| TCGA-FD-A6TF | 7 | 6 | 6 | 6 |  | 2.225082 |
| TCGA-FD-A6TE | 10 | 9 | 10 | 9 |  | 1.735259 |
| TCGA-FD-A6TD | 8 | 8 | 8 | 8 |  | 3.408142 |
| TCGA-FD-A6TC | 8 | 6 | 7 | 5 |  | 2.792331 |
| TCGA-FD-A6TB | 8 | 7 | 8 | 7 |  | 1.457907 |
| TCGA-FD-A6TA | 8 | 7 | 8 | 7 |  | 1.904457 |
| TCGA-FD-A62S | 7 | 6 | 7 | 6 |  | 3.516555 |
| TCGA-FD-A62P | 7 | 6 | 7 | 6 |  | 2.551338 |
| TCGA-FD-A62O | 7 | 6 | 7 | 5 |  | 0.938761 |
| TCGA-FD-A62N | 7 | 8 | 7 | 8 |  | 2.514228 |
| TCGA-FD-A5C1 | 8 | 8 | 8 | 8 |  | 1.45823 |
| TCGA-FD-A5C0 | 7 | 6 | 7 | 5 |  | 1.206243 |
| TCGA-FD-A5BZ | 8 | 6 | 7 | 6 |  | 4.411021 |
| TCGA-FD-A5BY | 8 | 7 | 8 | 7 |  | 4.752218 |
| TCGA-FD-A5BX | 9 | 8 | 8 | 7 |  | 3.83527 |
| TCGA-FD-A5BV | 8 | 7 | 8 | 6 |  | 2.316682 |
| TCGA-FD-A5BU | 7 | 7 | 7 | 7 |  | 3.252527 |
| TCGA-FD-A5BT | 8 | 8 | 8 | 8 |  | 3.115851 |
| TCGA-FD-A5BS | 8 | 9 | 8 | 9 |  | 1.847008 |
| TCGA-FD-A5BR | 8 | 7 | 8 | 7 |  | 1.823456 |
| TCGA-FD-A43Y | 9 | 8 | 8 | 7 |  | 5.213822 |
| TCGA-FD-A43X | 8 | 6 | 8 | 6 |  | 2.519831 |
| TCGA-FD-A43U | 7 | 6 | 7 | 6 |  | 1.557095 |
| TCGA-FD-A43S | 9 | 8 | 9 | 8 |  | 1.460629 |
| TCGA-FD-A43P | 9 | 8 | 9 | 9 |  | 2.508406 |
| TCGA-FD-A43N | 8 | 7 | 8 | 6 |  | 2.483083 |
| TCGA-FD-A3SS | 8 | 6 | 7 | 6 |  | 4.442266 |
| TCGA-FD-A3SR | 9 | 8 | 9 | 8 |  | 2.970314 |
| TCGA-FD-A3SQ | 8 | 7 | 8 | 7 |  | 1.073749 |
| TCGA-FD-A3SP | 7 | 7 | 7 | 7 |  | 1.791483 |
| TCGA-FD-A3SO | 8 | 7 | 7 | 7 |  | 1.141093 |
| TCGA-FD-A3SN | 8 | 7 | 7 | 6 |  | 5.865972 |
| TCGA-FD-A3SM | 8 | 6 | 7 | 6 |  | 1.830401 |
| TCGA-FD-A3SL | 8 | 7 | 8 | 6 |  | 2.979372 |
| TCGA-FD-A3SJ | 8 | 6 | 8 | 6 |  | 3.835055 |
| TCGA-FD-A3NA | 9 | 8 | 9 | 7 |  | 1.28502 |
| TCGA-FD-A3N6 | 9 | 8 | 9 | 8 |  | 5.675516 |
| TCGA-FD-A3N5 | 9 | 8 | 8 | 8 |  | 5.817028 |
| TCGA-FD-A3B8 | 7 | 8 | 7 | 8 |  | 2.049377 |
| TCGA-FD-A3B7 | 7 | 8 | 7 | 7 |  | 1.88304 |
| TCGA-FD-A3B6 | 7 | 8 | 7 | 8 |  | 1.711617 |
| TCGA-FD-A3B5 | 8 | 6 | 7 | 6 |  | 4.305674 |
| TCGA-FD-A3B4 | 9 | 9 | 9 | 8 |  | 4.714638 |
| TCGA-FD-A3B3 | 8 | 8 | 8 | 8 |  | 4.364477 |
| TCGA-E7-A97Q | 9 | 8 | 9 | 7 |  | 3.651871 |
| TCGA-E7-A97P | 8 | 8 | 8 | 8 |  | 2.191699 |
| TCGA-E7-A8O8 | 9 | 7 | 9 | 7 |  | 1.86313 |
| TCGA-E7-A8O7 | 9 | 8 | 8 | 7 |  | 0.933454 |
| TCGA-E7-A85H | 8 | 7 | 8 | 7 |  | 2.882645 |
| TCGA-E7-A7XN | 8 | 9 | 8 | 9 |  | 3.129584 |
| TCGA-E7-A7PW | 8 | 7 | 8 | 6 |  | 2.31411 |
| TCGA-E7-A7DV | 7 | 7 | 7 | 6 |  | 1.024428 |
| TCGA-E7-A7DU | 8 | 6 | 7 | 5 |  | 3.332387 |
| TCGA-E7-A6MF | 9 | 7 | 8 | 7 |  | 1.715922 |
| TCGA-E7-A6ME | 10 | 10 | 10 | 9 |  | 2.228141 |
| TCGA-E7-A6MD | 8 | 7 | 8 | 7 |  | 1.299095 |
| TCGA-E7-A678 | 10 | 8 | 9 | 7 |  | 1.972839 |
| TCGA-E7-A677 | 9 | 8 | 9 | 8 |  | 2.553618 |
| TCGA-E7-A5KF | 9 | 7 | 8 | 6 |  | 1.025119 |
| TCGA-E7-A5KE | 6 | 4 | 5 | 3 |  | 3.230856 |
| TCGA-E7-A541 | 10 | 9 | 9 | 9 |  | 3.701404 |
| TCGA-E7-A519 | 9 | 7 | 9 | 7 |  | 0.826631 |
| TCGA-E7-A4XJ | 7 | 5 | 6 | 4 |  | 5.082389 |
| TCGA-E7-A4IJ | 10 | 8 | 9 | 8 |  | 1.468882 |
| TCGA-E7-A3Y1 | 9 | 7 | 8 | 6 |  | 2.927681 |
| TCGA-E7-A3X6 | 9 | 9 | 9 | 8 |  | 3.051862 |
| TCGA-E5-A4U1 | 7 | 5 | 7 | 5 |  | 2.837224 |
| TCGA-E5-A4TZ | 7 | 5 | 6 | 4 |  | 5.520463 |
| TCGA-E5-A2PC | 10 | 9 | 10 | 8 |  | 1.30212 |
| TCGA-DK-AA77 | 10 | 9 | 10 | 9 |  | 5.556316 |
| TCGA-DK-AA76 | 8 | 6 | 7 | 5 |  | 2.509604 |
| TCGA-DK-AA75 | 7 | 5 | 7 | 5 |  | 4.394059 |
| TCGA-DK-AA74 | 8 | 8 | 8 | 8 |  | 2.150842 |
| TCGA-DK-AA71 | 8 | 6 | 8 | 6 |  | 4.444137 |
| TCGA-DK-AA6X | 9 | 8 | 9 | 7 |  | 1.919294 |
| TCGA-DK-AA6W | 7 | 5 | 6 | 4 |  | 4.455689 |
| TCGA-DK-AA6U | 9 | 7 | 8 | 6 |  | 1.566211 |
| TCGA-DK-AA6T | 10 | 9 | 9 | 8 |  | 2.820931 |
| TCGA-DK-AA6S | 8 | 7 | 8 | 7 |  | 2.525781 |
| TCGA-DK-AA6R | 6 | 6 | 6 | 6 |  | 1.267201 |
| TCGA-DK-AA6Q | 9 | 9 | 9 | 8 |  | 4.113004 |
| TCGA-DK-AA6P | 8 | 6 | 7 | 5 |  | 2.910342 |
| TCGA-DK-AA6M | 8 | 8 | 8 | 7 |  | 4.315312 |
| TCGA-DK-AA6L | 8 | 7 | 8 | 7 |  | 5.769222 |
| TCGA-DK-A6B6 | 10 | 9 | 10 | 9 |  | 0.88181 |
| TCGA-DK-A6B5 | 8 | 7 | 8 | 6 |  | 4.796742 |
| TCGA-DK-A6B2 | 9 | 8 | 9 | 8 |  | 2.25397 |
| TCGA-DK-A6B1 | 10 | 9 | 10 | 8 |  | 1.462619 |
| TCGA-DK-A6B0 | 8 | 7 | 8 | 6 |  | 2.386911 |
| TCGA-DK-A6AW | 8 | 6 | 7 | 6 |  | 1.034723 |
| TCGA-DK-A6AV | 8 | 6 | 8 | 6 |  | 3.527048 |
| TCGA-DK-A3X2 | 7 | 5 | 6 | 4 |  | 4.447248 |
| TCGA-DK-A3X1 | 9 | 7 | 9 | 7 |  | 3.058561 |
| TCGA-DK-A3WY | 9 | 9 | 9 | 10 |  | 2.521471 |
| TCGA-DK-A3WX | 7 | 7 | 7 | 7 |  | 2.079082 |
| TCGA-DK-A3WW | 8 | 9 | 8 | 9 |  | 4.013085 |
| TCGA-DK-A3IV | 9 | 9 | 9 | 8 |  | 3.110124 |
| TCGA-DK-A3IU | 8 | 9 | 8 | 9 |  | 2.798307 |
| TCGA-DK-A3IT | 8 | 6 | 7 | 6 |  | 3.290003 |
| TCGA-DK-A3IS | 10 | 9 | 10 | 9 |  | 1.410435 |
| TCGA-DK-A3IQ | 7 | 6 | 7 | 5 |  | 1.357687 |
| TCGA-DK-A3IN | 6 | 6 | 6 | 6 |  | 2.815038 |
| TCGA-DK-A3IM | 8 | 6 | 7 | 5 |  | 5.450117 |
| TCGA-DK-A3IL | 7 | 5 | 6 | 5 |  | 5.523246 |
| TCGA-DK-A3IK | 9 | 7 | 8 | 7 |  | 1.572032 |
| TCGA-DK-A2I6 | 7 | 7 | 6 | 6 |  | 1.80567 |
| TCGA-DK-A2I4 | 8 | 9 | 8 | 9 |  | 1.918127 |
| TCGA-DK-A2I2 | 7 | 6 | 7 | 6 |  | 1.426938 |
| TCGA-DK-A2I1 | 8 | 7 | 8 | 7 |  | 3.160514 |
| TCGA-DK-A2HX | 7 | 6 | 7 | 5 |  | 1.118682 |
| TCGA-DK-A1AG | 8 | 6 | 8 | 6 |  | 3.515154 |
| TCGA-DK-A1AF | 8 | 7 | 8 | 7 |  | 1.604777 |
| TCGA-DK-A1AE | 8 | 7 | 8 | 6 |  | 4.186274 |
| TCGA-DK-A1AD | 9 | 8 | 9 | 7 |  | 0.977689 |
| TCGA-DK-A1AC | 7 | 7 | 7 | 7 |  | 2.766123 |
| TCGA-DK-A1AB | 8 | 7 | 8 | 7 |  | 5.229456 |
| TCGA-DK-A1AA | 8 | 6 | 8 | 6 |  | 2.300134 |
| TCGA-DK-A1A7 | 9 | 7 | 8 | 6 |  | 1.865921 |
| TCGA-DK-A1A6 | 8 | 7 | 7 | 7 |  | 3.342215 |
| TCGA-DK-A1A5 | 10 | 9 | 9 | 8 |  | 1.73824 |
| TCGA-DK-A1A3 | 7 | 6 | 7 | 6 |  | 4.367472 |
| TCGA-CU-A72E | 7 | 5 | 6 | 5 |  | 2.320385 |
| TCGA-CU-A5W6 | 8 | 7 | 8 | 6 |  | 1.161822 |
| TCGA-CU-A3YL | 9 | 7 | 9 | 7 |  | 2.353877 |
| TCGA-CU-A3QU | 10 | 9 | 10 | 8 |  | 2.477446 |
| TCGA-CU-A3KJ | 9 | 7 | 8 | 7 |  | 4.283619 |
| TCGA-CU-A0YR | 7 | 7 | 7 | 7 |  | 3.128553 |
| TCGA-CU-A0YO | 10 | 9 | 10 | 8 |  | 3.777906 |
| TCGA-CU-A0YN | 7 | 6 | 7 | 6 |  | 2.466294 |
| TCGA-CF-A9FM | 8 | 7 | 8 | 6 |  | 1.588472 |
| TCGA-CF-A9FL | 8 | 6 | 8 | 6 |  | 2.948133 |
| TCGA-CF-A9FH | 10 | 9 | 10 | 9 |  | 0.971118 |
| TCGA-CF-A9FF | 9 | 8 | 9 | 7 |  | 2.2326 |
| TCGA-CF-A8HY | 7 | 6 | 7 | 5 |  | 2.100521 |
| TCGA-CF-A8HX | 7 | 5 | 6 | 4 |  | 4.318302 |
| TCGA-CF-A7I0 | 8 | 6 | 8 | 6 |  | 4.038004 |
| TCGA-CF-A5UA | 8 | 6 | 8 | 6 |  | 1.172254 |
| TCGA-CF-A5U8 | 8 | 6 | 8 | 6 |  | 0.813138 |
| TCGA-CF-A47Y | 8 | 7 | 8 | 6 |  | 1.61824 |
| TCGA-CF-A47X | 8 | 7 | 8 | 6 |  | 3.301484 |
| TCGA-CF-A47W | 9 | 7 | 8 | 6 |  | 3.127768 |
| TCGA-CF-A47V | 10 | 8 | 9 | 8 |  | 1.231596 |
| TCGA-CF-A47T | 7 | 5 | 6 | 5 |  | 4.088938 |
| TCGA-CF-A47S | 8 | 7 | 8 | 6 |  | 0.827064 |
| TCGA-CF-A3MI | 8 | 6 | 8 | 6 |  | 1.175527 |
| TCGA-CF-A3MH | 9 | 7 | 8 | 6 |  | 3.571937 |
| TCGA-CF-A3MG | 8 | 6 | 7 | 5 |  | 3.054953 |
| TCGA-CF-A3MF | 7 | 5 | 6 | 5 |  | 1.59739 |
| TCGA-CF-A27C | 9 | 7 | 8 | 7 |  | 0.954993 |
| TCGA-CF-A1HS | 7 | 6 | 7 | 6 |  | 3.043062 |
| TCGA-CF-A1HR | 9 | 8 | 9 | 7 |  | 2.255247 |
| TCGA-C4-A0F7 | 7 | 6 | 7 | 5 |  | 4.539794 |
| TCGA-C4-A0F6 | 8 | 6 | 7 | 6 |  | 3.375193 |
| TCGA-C4-A0F1 | 9 | 7 | 8 | 7 |  | 4.149502 |
| TCGA-C4-A0F0 | 8 | 7 | 7 | 7 |  | 1.26839 |
| TCGA-C4-A0EZ | 6 | 4 | 6 | 4 |  | 5.892446 |
| TCGA-BT-A42F | 7 | 7 | 7 | 7 |  | 2.022459 |
| TCGA-BT-A42E | 7 | 7 | 8 | 7 |  | 3.338545 |
| TCGA-BT-A42C | 8 | 6 | 8 | 6 |  | 4.638431 |
| TCGA-BT-A3PK | 7 | 6 | 7 | 6 |  | 2.453848 |
| TCGA-BT-A3PJ | 9 | 9 | 9 | 9 |  | 2.455647 |
| TCGA-BT-A3PH | 7 | 5 | 6 | 4 |  | 2.146949 |
| TCGA-BT-A2LD | 8 | 8 | 8 | 7 |  | 2.539532 |
| TCGA-BT-A2LB | 8 | 7 | 8 | 7 |  | 1.092318 |
| TCGA-BT-A2LA | 8 | 6 | 8 | 5 |  | 4.584981 |
| TCGA-BT-A20X | 8 | 7 | 8 | 6 |  | 5.710267 |
| TCGA-BT-A20W | 9 | 7 | 9 | 7 |  | 3.035871 |
| TCGA-BT-A20V | 8 | 8 | 7 | 8 |  | 7.088623 |
| TCGA-BT-A20U | 8 | 6 | 8 | 6 |  | 4.210062 |
| TCGA-BT-A20T | 10 | 9 | 9 | 8 |  | 1.589038 |
| TCGA-BT-A20R | 8 | 6 | 7 | 6 |  | 1.799557 |
| TCGA-BT-A20Q | 9 | 8 | 9 | 8 |  | 1.165543 |
| TCGA-BT-A20P | 9 | 7 | 8 | 6 |  | 2.331097 |
| TCGA-BT-A20O | 8 | 9 | 8 | 9 |  | 2.285145 |
| TCGA-BT-A20N | 8 | 6 | 7 | 5 |  | 1.678766 |
| TCGA-BT-A20J | 7 | 7 | 7 | 6 |  | 1.280255 |
| TCGA-BT-A0YX | 7 | 6 | 7 | 6 |  | 4.633179 |
| TCGA-BT-A0S7 | 8 | 6 | 8 | 6 |  | 3.641633 |
| TCGA-BL-A5ZZ | 6 | 6 | 6 | 5 |  | 2.220376 |
| TCGA-BL-A3JM | 8 | 7 | 8 | 7 |  | 1.924193 |
| TCGA-BL-A13J | 8 | 6 | 7 | 6 |  | 4.51094 |
| TCGA-BL-A13I | 7 | 7 | 7 | 7 |  | 3.311749 |
| TCGA-BL-A0C8 | 8 | 6 | 7 | 5 |  | 2.754225 |
| TCGA-5N-A9KM | 9 | 7 | 9 | 7 |  | 1.401567 |
| TCGA-5N-A9KI | 10 | 8 | 9 | 8 |  | 3.105995 |
| TCGA-4Z-AA89 | 10 | 8 | 10 | 8 |  | 3.687265 |
| TCGA-4Z-AA87 | 7 | 7 | 7 | 6 |  | 5.116151 |
| TCGA-4Z-AA86 | 7 | 7 | 7 | 8 |  | 3.484024 |
| TCGA-4Z-AA84 | 7 | 6 | 6 | 5 |  | 1.423099 |
| TCGA-4Z-AA83 | 9 | 7 | 8 | 7 |  | 4.133405 |
| TCGA-4Z-AA82 | 8 | 7 | 7 | 6 |  | 4.046123 |
| TCGA-4Z-AA81 | 10 | 10 | 10 | 10 |  | 1.035483 |
| TCGA-4Z-AA80 | 9 | 8 | 9 | 7 |  | 3.697859 |
| TCGA-4Z-AA7Y | 9 | 7 | 9 | 7 |  | 4.131536 |
| TCGA-4Z-AA7W | 9 | 9 | 10 | 10 |  | 2.262685 |
| TCGA-4Z-AA7S | 7 | 5 | 7 | 5 |  | 3.777857 |
| TCGA-4Z-AA7R | 8 | 6 | 7 | 5 |  | 3.337761 |
| TCGA-4Z-AA7Q | 8 | 8 | 8 | 8 |  | 1.079684 |
| TCGA-4Z-AA7O | 10 | 9 | 10 | 8 |  | 1.865364 |
| TCGA-4Z-AA7N | 9 | 9 | 9 | 9 |  | 2.83335 |
| TCGA-4Z-AA7M | 8 | 6 | 7 | 5 |  | 1.483144 |
| TCGA-2F-A9KW | 7 | 6 | 7 | 6 |  | 2.670682 |
| TCGA-2F-A9KT | 7 | 5 | 6 | 4 |  | 2.637077 |
| TCGA-2F-A9KR | 9 | 7 | 9 | 7 |  | 4.202711 |
| TCGA-2F-A9KQ | 8 | 6 | 7 | 5 |  | 4.994514 |
| TCGA-2F-A9KP | 8 | 6 | 7 | 6 |  | 3.589416 |
| TCGA-2F-A9KO | 10 | 9 | 10 | 9 |  | 1.49009 |
